# Supplementary material for: Biomimetically inspired asymmetric total synthesis of (+)-19-dehydroxyl arisandilactone A
Source: Nat Commun. 2017 Jan 31;8:14233. doi: 10.1038/ncomms14233 (PMC5290315; doi:10.1038/ncomms14233)
Supplement: Supplementary Information — Supplementary figures, supplementary table, supplementary methods and supplementary references. [file ncomms14233-s1.pdf]

Supplementary Methods.

General information for experimentation.

All reactions were carried out under a nitrogen atmosphere under anhydrous conditions and all reagents were purchased from commercial suppliers without further purification. Solvent purification was conducted according to Purification of Laboratory Chemicals (Peerrin, D. D.; Armarego, W. L. and Perrins, D. R., Pergamon Press: Oxford, 1980). Yields refer to chromatographically and spectroscopically (^{1}H NMR) homogeneous materials. Reactions were monitored by Thin Layer Chromatography on plates (GF254) supplied by Yantai Chemicals (China) visualized by UV or stained with ethanolic solution of phosphomolybdic acid and cerium sulfate, basic solution of $KMnO_4$, and iodine vapor. If not specially mentioned, flash column chromatography was performed using E. Merck silica gel (60, particle size 0.040–0.063 mm). NMR spectra were recorded on Bruker AV400, Bruker AV500 instruments and calibrated by using residual undeuterated chloroform (δH = 7.26 ppm) and $CDCl_3$ (δC = 77.16 ppm), partially-deuterated methylene chloride (δH= 5.32 ppm) and methylene chloride-*d*2 (δC = 53.84 ppm), partially-deuterated methanol (δH= 3.31 ppm) and methanol-*d*4 (δC = 49.00 ppm) as internal references. The following abbreviations were used to explain the multiplicities: s = singlet, d = doublet, t = triple, q = quartet, br = broad, td = triple doublet, dt = double triplet, dq = double quartet, m = multiplet. Infrared (IR) spectra were recorded on a Thermo Nicolet Avatar 330 FT-IR spectrometer. High-resolution mass spectra (HRMS) were recorded on a Bruker Apex IV FTMS mass spectrometer using ESI (electrospray ionization) as ionization method.

Synthesis of compound 9.

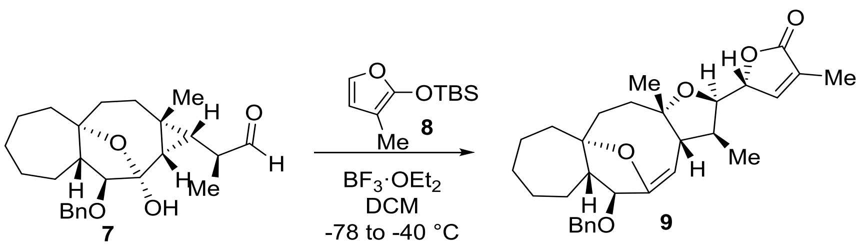

Synthesis of To a solution of **8** (28 mg, 0.13 mmol) and **7** (26 mg, 0.065 mmol) was slowly added a DCM solution of BF_3•OEt_2 (9.2 mg, 0.065 mmol in 0.5 mL DCM) at -78°C. The mixture was stirred at this temperature for 1 h. After that, the reaction was quenched with saturated solution of $NaHCO_3$ (1 mL). The aqueous phase was extracted with EtOAc (5 mL×3) and the combined organic phase was washed by brine (1 mL), dried over Na_2SO_4 and concentrated in vacuo. Purification by silica gel column chromatography (petroleum ether/ethyl acetate 15:1) afforded product **9** (15 mg) in 48% yield. R_f = 0.35 (silica gel, PE/EA = 2:1); ^{1}H-NMR (500 MHz, $CDCl_3$): δ = 7.38-7.28 (m, 5H), 6.97-6.96 (m, 1H), 4.97 (dd, *J* = 9.1, 1.8 Hz, 1H), 4.89 (m, 1H), 4.69 (d, *J* = 11.9 Hz, 1H), 4.58 (d, *J* = 11.9 Hz, 1H), 4.25 (d, *J* = 8.7 Hz, 1H), 3.82 (dd, *J* = 3.6, 1.5 Hz, 1H), 3.40-3.32 (m, 1H), 2.33 (dd, *J* = 12.8, 8.6 Hz, 1H), 1.99-1.95 (m, 1H), 1.94 (t, *J* = 1.6 Hz, 3H), 1.85 (dd, *J* = 13.8, 9.4 Hz, 1H), 1.82-1.76 (m, 1H), 1.71-1.63 (m, 6H), 1.58-1.50 (m, 2H), 1.36-1.26 (m, 3H), 1.23-1.14 (m, 1H), 1.19 (d, *J* = 6.9 Hz, 3H), 1.16 (s, 3H) ppm; ^{13}C-NMR (125 MHz, $CDCl_3$): δ = 175.0, 157.5, 147.0, 138.4, 130.9, 128.6, 127.9, 127.8, 100.3, 88.0, 86.9, 84.2,

81.1, 77.4, 73.1, 54.6, 51.0, 40.6, 34.2, 34.1, 32.5, 31.3, 29.8, 29.6, 26.5, 24.2, 12.9, 11.0 ppm; IR (neat): ν_{max} = 2927, 2847, 2357, 1755, 1565, 1093 cm^{-1}; HRMS (ESI, *m/z*): $[M+H]^+$ calcd for $C_{30}H_{39}O_5$, 479.2792; found 479.2806.

Synthesis of compound 10.

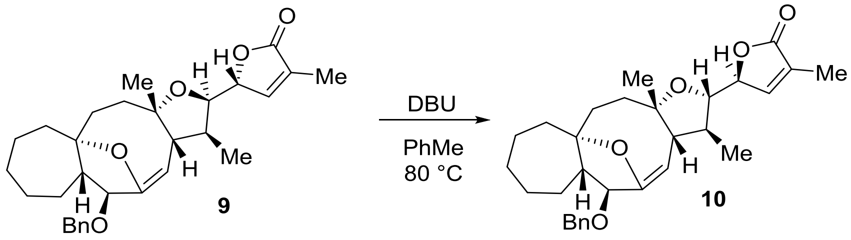

To a solution of **9** (9 mg, 0.0182 mmol in 4.5 mL toluene) was added DBU (194 mg, 1.27 mmol). Then the mixture was heated under 80 °C for 16h. After that, the reaction was quenched with saturated solution of NH_4Cl (1 mL). The aqueous phase was extracted with EtOAc (5 mL×3) and the combined organic phase was washed by brine (1 mL), dried over Na_2SO_4 and concentrated in vacuo. Purification by silica gel column chromatography (petroleum ether/ethyl acetate 20:1) afforded product **10** (6 mg) in 65% yield. R_f = 0.35 (silica gel, PE/EA = 2:1); ^{1}H-NMR (500 MHz, $CDCl_3$): δ = 7.38-7.28 (m, 5H), 7.03 (t, *J* = 1.6 Hz, 1H), 4.95 (dd, *J* = 9.0, 1.8 Hz, 1H), 4.92 (dt, *J* = 3.6, 1.9 Hz, 1H), 4.69 (d, *J* = 11.9 Hz, 1H), 4.59 (d, *J* = 12.0 Hz, 1H), 3.84 (dd, *J* = 4.1, 1.8 Hz, 1H), 3.60 (dd, *J* = 9.6, 3.2 Hz, 1H), 3.09 (ddq, *J* = 16.0, 9.5, 6.5 Hz, 1H), 2.06 (dd, *J* = 14.7, 8.4 Hz, 1H), 2.00 (dd, *J* = 12.3, 8.9 Hz, 1H), 1.93 (t, *J* = 1.8 Hz, 3H), 1.82-1.67 (m, 6H), 1.62-1.52 (m, 3H), 1.36-1.25 (m, 3H), 1.24 (s, 3H), 1.20-1.12 (m, 2H), 1.05 (d, *J* = 6.4 Hz, 3H) ppm; ^{13}C-NMR (125 MHz, $CDCl_3$): δ = 174.3, 158.2, 146.3, 138.4, 130.9, 128.6, 127.9, 127.7, 99.6, 88.2, 87.1, 83.6, 81.0, 77.4, 73.0, 55.1, 54.1, 40.2, 34.3, 33.4, 32.4, 31.4, 29.5, 27.7, 24.2, 15.9, 11.0 ppm; IR (neat): ν_{max} = 2924, 2854, 1759, 1454, 1102, 739 cm^{-1}; HRMS (ESI, *m/z*): $[M+H]^+$ calcd for $C_{30}H_{39}O_5$, 479.2792; found 479.2797. CCDC 1509144 contains the supplementary crystallographic data for compound **10** and is available free of charge from The Cambridge Crystallographic Data Centre via www.ccdc.cam.ac.uk/data_request/cif.

Synthesis of compound S1.

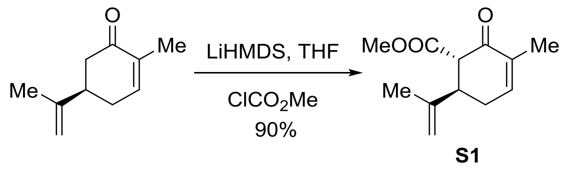

To a LiHMDS solution (1 L, 1.0 mol/L in THF, 1.0 mol) was added *R*-(-)-carvone (71.52 g, 0.48 mol, in 150 mL THF) at -78 °C over 15 min and the mixture was slowly warmed to 0 °C over 1 h. After stirring for another 0.5 h, the mixture was cooled to -78 °C again, and $ClCO_2Me$ (67.50 g, 0.71 mol, in 400 mL THF) was added slowly via cannula over 2 h. After 15 min, the reaction was quenched by NH_4Cl (sat. aq., 400 mL) *in situ*. The aqueous phase was extracted with dichloromethane (500 mL×3) and the combined organic phase was washed by brine (500 mL), dried over Na_2SO_4 and concentrated in vacuo. The residue could directly move to the next step, or be purified by column chromatography (petroleum ether/ethyl acetate, 20:1 to 10:1) to give **S1**

(89.43 g, 90%) as yellowish oil. The characterization data matched with previous report[1].

Synthesis of compound 11.

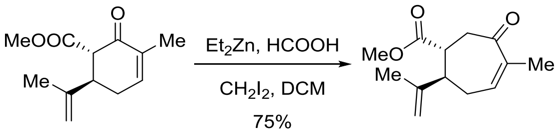

To a Et_2Zn solution (288 mL, 2.0 mol/L in hexane, 0.58 mol) was added HCO_2H (26.47 g, 0.58 mol, in 500 mL dichloromethane) at 0 °C over 2 h. (CAUTION: Mass of gas carrying white smoke was released. Be careful of plugged lines and explosion!) After stirring for another 10 min, CH_2I_2 (154.1 g, 0.58 mol, in 150 mL dichloromethane) was added in a drop-wise manner over 15 min. The zinc reagent was stirred for another 1 h and then **S1** (52.10 g, 0.25 mol, in 150 mL dichloromethane) was added within 10 min (CAUTION: severe exothermic and gas-releasing. STIR VIGOROUSLY and KEEP COOL). The reaction mixture was stirred for another 2 h at 0 °C and quenched by NH_4Cl (sat. aq., 300 mL) *in situ*. The aqueous phase was extracted with dichloromethane (500 mL×3) and the combined organic phase was washed by brine (500 mL), dried over Na_2SO_4 and concentrated in vacuo. The residue could directly move to the next step, or be purified by column chromatography (petroleum ether/ethyl acetate, 20:1 to 8:1) to give **11** (41.78 g, 75%) as colorless oil. R_f = 0.4 (silica gel, petroleum ether/ethyl acetate, 4:1); $[\alpha]_D^{20}$ = + 21.0 (c = 0.96 in $CHCl_3$); ^1H NMR (400 MHz, $CDCl_3$): δ = 6.51 (ddq, J = 8.0, 6.4, 1.2 Hz, 1H), 4.75-4.74 (m, 1H), 4.72 (s br, 1H), 3.63 (s, 3H), 2.95 (td, J = 7.6, 4.8 Hz, 1H), 2.86-2.70 (m, 3H), 2.48-2.35 (m, 2H), 1.78 (d, J = 1.2 Hz, 3H), 1.72 (s br, 3H) ppm; ^{13}C NMR (100 MHz, $CDCl_3$): δ = 201.4, 174.5, 146.6, 140.0, 139.9, 111.7, 52.1, 46.3, 43.3, 43.1, 30.6, 20.7, 18.6 ppm; IR (neat): ν_{max} = 2984, 2897, 1731, 1661, 1437, 1375, 1167, 1059, 898 cm^{-1}; HRMS (ESI): m/z calcd for $C_{13}H_{18}O_3Na$ $[M+Na]^+$: 245.1148, found 245.1150.

Synthesis of compound S2.

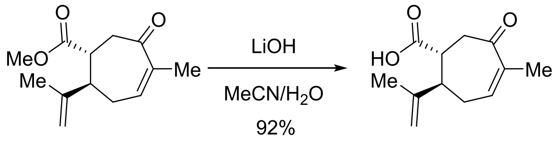

To the solution of **11** (133.21 g, 0.60 mol) in $MeCN/H_2O$ (500mL/500mL) was added LiOH (31.62 g, 1.32 mol), and the mixture was stirred at 25 °C for 24 h. After MeCN was removed under vacuum, the aqueous phase was washed by dichloromethane (50 mL×2), acidified with HCl (12 mol/L in H_2O) to pH ≈ 1 and extracted with dichloromethane (400 mL×3). The dichloromethane solution was washed by brine (300 mL) and dried over Na_2SO_4 and concentrated in vacuo. After filtration by silica gel and concentration, the residue could directly move to the next step, or be purified by column chromatography (dichloromethane/MeOH, 20:1) to give **S2** (115.02 g, 92%) as yellowish oil. R_f = 0.1 (silica gel, petroleum ether/ethyl acetate, 4:1); $[\alpha]_D^{20}$ = + 41.2 (c = 1.0 in $CHCl_3$); ^1H NMR (400 MHz, $CDCl_3$): δ = 10.76 (s br, 1H), 6.52 (t, J = 7.2 Hz, 1H), 4.79 (m, 1H), 4.77 (s br, 1H), 2.95 (q, J = 6.4 Hz, 1H), 2.90-2.84 (m, 2H), 2.81-2.75 (m, 1H), 2.43 (t, J = 6.4 Hz, 2H), 1.80 (s br, 3H), 1.75 (s, 3H) ppm; ^{13}C NMR (100 MHz, $CDCl_3$): δ = 201.8, 179.8, 146.4, 140.1, 140.0, 111.9, 46.1, 43.1, 42.9, 30.6, 20.9, 18.6 ppm; IR (neat): ν_{max} = 2967, 1732, 1704,

1663, 1644, 1455, 1374, 1180, 892 cm^{-1}; HRMS (ESI): *m/z* calcd for $C_{12}H_{16}NaO_3$ $[M+Na]^+$: 231.0992, found 231.0992.

Synthesis of compound 12.

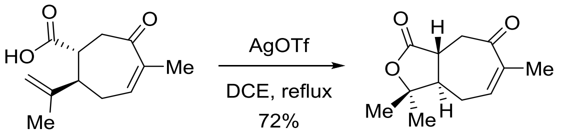

S2 **12**

To the solution of **S2** (98.13 g, 0.47 mol) in DCE (1 L) was added AgOTf (6.052 g, 24 mmol). The mixture was protected by N_2 and heated to reflux for 2 h. After cooled to room temperature, the mixture was filtered with a short pad of silica gel and eluted with petroleum ether/ethyl acetate (2:1). The eluent was removed under vacuum and the desired product could crystallize from petroleum ether/ethyl acetate, or be purified by column chromatography (petroleum ether/ethyl acetate, 10:1 to 2:1) to give **12** (70.849 g, 72%) as white solid. R_f = 0.2 (silica gel, petroleum ether/ethyl acetate, 4:1); $[\alpha]_D^{20}$ = -156.5 (*c* = 0.99 in $CHCl_3$); ^1H NMR (400 MHz, $CDCl_3$): δ = 6.30 (d, *J* = 6.0 Hz, 1H), 3.22 (dd, *J* = 16.4, 4.4 Hz, 1H), 2.81 (td, *J* = 12.0, 4.4 Hz, 1H), 2.63 (dd, *J* = 16.4, 12.0 Hz, 1H), 2.49 (dd, *J* = 14.0, 6.4 Hz, 1H), 2.39-2.33 (m, 2H), 1.90 (s, 3H), 1.49 (s, 3H), 1.30 (s, 3H) ppm; ^{13}C NMR (100 MHz, $CDCl_3$): δ = 200.2, 175.5, 138.3, 135.4, 84. 5, 50.5, 44.6, 40.4, 30.3, 27.5, 22.6, 22.5 ppm; IR (neat): ν_{max} = 2976, 2900, 1764, 1654, 1392, 1377, 1271, 1055, 956 cm^{-1}; HRMS (ESI): *m/z* calcd for $C_{12}H_{17}O_3$ $[M+H]^+$: 209.1172, found 209.1178.

Synthesis of compound S3.

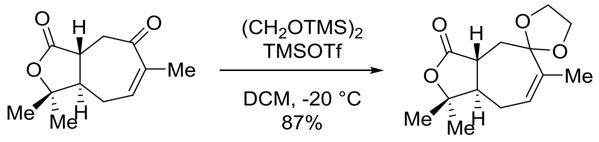

12 **S3**

To the solution of **12** (85.23 g, 0.41 mol) in dichloromethane (410 mL) was added $(CH_2OTMS)_2$ (118.27 g, 0.57 mol) in one portion and TMSOTf (9.096 g, 41 mmol) in a drop-wise manner at -78 ^oC under N_2 protection. The mixture was slowly warmed to -20 ^oC over 3 h, stirred for another 24 h and quenched by $NaHCO_3$ (sat. aq., 300 mL) *in situ*. The aqueous phase was extracted with dichloromethane (100 mL×3) and the combined organic phase was washed by brine (200 mL), dried over Na_2SO_4 and concentrated in vacuo. The residue could directly move to the next step, or be purified by column chromatography (petroleum ether/ethyl acetate, 12:1 to 3:1) to give **S3** (89.67 g, 87%) as white solid. R_f = 0.25 (silica gel, petroleum ether/ethyl acetate, 4:1); $[\alpha]_D^{20}$ = + 65.9 (*c* = 0.92 in $CHCl_3$); ^1H NMR (400 MHz, $CDCl_3$): δ = 5.70 (dq, *J* = 9.1, 1.8 Hz, 1H), 4.05-3.91 (m, 4H), 2.89 (td, *J* = 12.4, 2.8 Hz, 1H), 2.34 (dd, *J* = 13.7, 2.8 Hz, 1H), 2.20 (ddq, *J* = 14.8, 12.4, 2.1 Hz, 1H), 2.04 (ddd. *J* = 14.8, 9.1, 2.4 Hz, 1H), 1.87 (td, *J* = 12.4, 2.4 Hz, 1H), 1.78-1.77 (m, 3H), 1.66 (dd, *J* = 13.7, 12.4 Hz, 1H) 1.42 (s, 3H), 1.25 (s, 3H) ppm; ^{13}C NMR (100 MHz, $CDCl_3$): δ = 177.2, 142.2, 125.1, 108.7, 84.9, 65.1, 64.5, 50.8, 42.5, 36.0, 27.1, 26. 7, 22.2, 21.2 ppm; IR (neat): ν_{max} = 2976, 2884, 1773, 1441, 1371, 1267, 1167, 1126, 1051, 947 cm^{-1}; HRMS (ESI): *m/z* calcd for $C_{14}H_{20}O_4Na$ $[M+Na]^+$: 275.1254, found 275.1255.

Synthesis of compound 13.

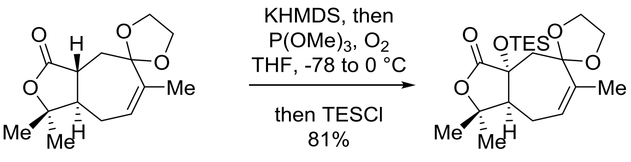

To a solution of compound **S3** (2.80 g, 11.1 mmol) in THF (45 ml) was added potassium bis(trimethylsilyl)amide (1.0 M solution in THF, 22 ml, 22 mmol) in drop-wise manner at -78 °C under nitrogen atmosphere, and the resultant mixture was stirred at the same temperature for 10 min. After warming up to 0 °C, the reaction mixture was stirred at the same temperature for 45 min, and then cooled back to -78 °C. To this solution was added $P(OMe)_3$ (2.1 mL, 17.6 mmol) in one portion, and the resultant mixture was degassed with O_2 for 3 times, stirred at -78 °C for 20 min under oxygen atmosphere, and then stirred 0 °C for 1 h. After changing the reaction atmosphere from oxygen to nitrogen again, the reaction mixture was treated with TESCl (2.6 mL, 15.4 mmol), and the formed mixture was stirred at 0 °C overnight. The reaction mixture was quenched with a saturated solution of $NaHCO_3$, and extracted with EtOAc (3×30 mL). The combined organic layer was washed with brine (100 mL), dried over Na_2SO_4. The solvent was removed under vacuum, and the residue was purified by a flash column chromatography on silica gel (petroleum ether/ethyl acetate = 50:1 to 20:1) to give product **13** (3.44 g, 81% yield) as white solid. R_f = 0.5 (silica gel, petroleum ether/ethyl acetate, 4:1); $[\alpha]_D^{20}$ = + 56.6 (*c* = 1.08 in $CHCl_3$); ^1H NMR (400 MHz, $CDCl_3$): δ = 5.63 (ddd, *J* = 7.4, 6.2, 1.3 Hz, 1H), 4.07-3.89 (m, 4H), 2.41 (d, *J* = 14.8 Hz, 1H), 2.38 (dd, *J* = 10.4, 4.4 Hz, 1H), 2.30 (tdd, *J* = 10.5, 6.1, 1.0 Hz, 1H), 2.23-2.18 (m, 1H), 2.18 (d, *J* = 14.7 Hz, 1H), 1.69 (s, 3H), 1.49 (s, 3H), 1.29 (s, 3H), 0.93 (t, *J* = 7.9 Hz, 9H), 0.69-0.62 (m, 6H) ppm; ^{13}C NMR (100 MHz, $CDCl_3$): δ = 176.8, 140.2, 124.3, 108.3, 84.1, 79.2, 65.3, 64.9, 55.9, 43.8, 30.7, 25.3, 24.4, 19.5, 7.2, 6.2 ppm; IR (neat): ν_{max} = 2947, 2876, 2333, 2358, 1769, 1458, 1371, 1267, 1184, 1110, 1055, 1006, 947, 889 cm^{-1}; HRMS (ESI): *m/z* calcd for $C_{20}H_{35}O_5Si$ $[M+H]^+$: 383.2248, found 383.2243.

Synthesis of compound S4.

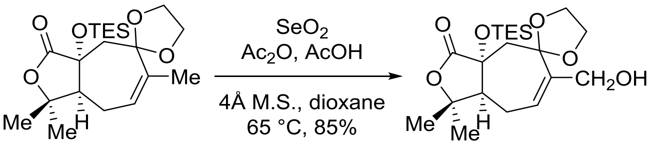

Note: strict anhydrous is crucial for this Riley oxidation.

To a flame-dried flask was added dried 4Å molecular sieves (24.02 g, m_{sub}×1.2) and SeO_2 (17.45 g, 0.16 mol). After protection by N_2, dioxane (420 mL), Ac_2O (5.34 g, 52 mmol) and AcOH (520 mg, 8.7 mmol) were added subsequently via syringe, and the mixture was stirred for 15 min at 65 °C. After cooled to room temperature, **13** (20.043 g, 52 mmol, in 100 mL dioxane) was added via syringe. The mixture was stirred under room temperature for 15 min and warmed to 65 °C for another 24 h. After cooled to room temperature, the mixture was poured into $NaHCO_3/Na_2CO_3$ buffer (aq., 500 mL, pH ≈ 9) and the resultant biphasic suspension was filtered with a pad of celite/silica gel and eluted with ethyl acetate. After separation of the biphasic solution, the aqueous phase was extracted with dichloromethane (150 mL×3) and the combined organic phase was

washed by brine (300 mL), dried over Na_2SO_4 and concentrated in vacuo. The residue was purified by column chromatography (petroleum ether/ethyl acetate, 4:1 to 1:1) to give **S4** (17.682 g, 85%) as yellowish oil. R_f = 0.15 (silica gel, petroleum ether/ethyl acetate, 2:1); $[\alpha]_D^{20}$ = + 53.5 (*c* = 0.96 in $CHCl_3$); ^{1}H NMR (400 MHz, $CDCl_3$): δ = 5.97 (t, *J* = 6.8 Hz, 1H), 4.12-3.92 (m, 6H), 2.44-2.31 (m, 5H), 2.18 (d, *J* = 14.7 Hz, 1H), 1.48 (s, 3H), 1.29 (s, 3H), 0.92 (t, *J* = 7.9 Hz, 9H), 0.68-0.60 (m, 6H) ppm; ^{13}C NMR (100 MHz, $CDCl_3$): δ = 176.5, 142.0, 127.9, 108.7, 84.0, 78.9, 64.8, 64.8, 64.7, 55.6, 43.8, 30.7, 25.3, 24.0, 7.1, 6.1 ppm; IR (neat): ν_{max} = 2958, 2874, 2345, 1771, 1276, 1188, 1093, 1054, 1004 cm^{-1}; HRMS (ESI): *m/z* calcd for $C_{20}H_{35}O_6Si$ $[M+H]^+$: 399.2197, found 399.2202.

Synthesis of compound S5.

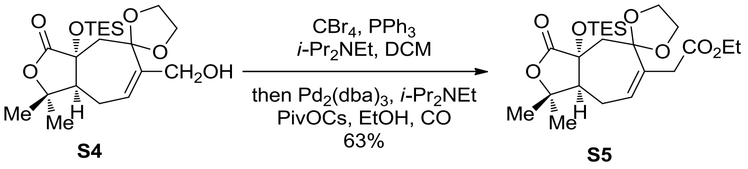

To the solution of **S4** (204 mg, 0.51 mmol) in dichloromethane (5 mL) was added PPh_3 (161 mg, 0.61 mmol) and *i*-Pr_2NEt (26 mg, 0.2 mmol). After cooling the mixture to 0 °C, CBr_4 (204 mg, 0.61 mmol) was added in one portion. The mixture was stirred at 0 °C for 3 h under N_2 protection. After conversion completed, the mixture was added by EtOH (20 mL), PivOCs (60 mg, 0.26 mmol), *i*-Pr_2NEt (52 mg, 0.4 mmol), $Pd_2(dba)_3$ (23 mg, 0.025 mmol), degassed with CO for 3 times and allowed to warm to room temperature slowly. After stirring for another 24 h in dark, the mixture was filtered with a short pad of silica gel eluted with ethyl acetate. The solvent was thoroughly removed under vacuum and the residue could be purified by column chromatography (dichloromethane/actone, 500:1 to 80:1) to give **S5** (147 mg, 63%) as yellowish oil. R_f = 0.45 (silica gel, petroleum ether/ethyl acetate, 2:1); $[\alpha]_D^{20}$ = + 46.5 (*c* = 0.8 in $CHCl_3$); ^{1}H NMR (400 MHz, $CDCl_3$): δ = 5.83-5.81 (m, 1H), 4.09 (q, *J* = 7.1 Hz, 2H), 4.03-3.97 (m, 2H), 3.95-3.86 (m, 2H), 3.03 (d, *J* = 15.0 Hz, 1H), 2.89 (d, *J* = 15.0 Hz, 1H), 2.51-2.43 (m, 2H), 2.35 (d, *J* = 14.7 Hz, 1H), 2.36-2.28 (m, 1H), 2.24 (d, *J* = 14.7 Hz, 1H), 1.48 (s, 3H), 1.33 (s, 3H), 1.21 (t, *J* = 7.1 Hz, 3H), 0.92 (t, *J* = 7.9 Hz, 9H), 0.71-0.56 (m, 6H) ppm; ^{13}C NMR (100 MHz, $CDCl_3$): δ = 176.4, 172.0, 137.2, 129.8, 107.5, 84.4, 79.2, 65.2, 64.6, 60.6, 55.7, 43.2, 39.3, 31.1, 25.4, 24.2, 14.3, 7.1, 6.2 ppm; IR (neat): ν_{max} = 2955, 2868, 1769, 1727, 1267, 1155, 1093, 1014 cm^{-1}; HRMS (ESI): *m/z* calcd for $C_{23}H_{39}O_7Si$ $[M+H]^+$: 455.2460, found 455.2467.

Synthesis of compound 14.

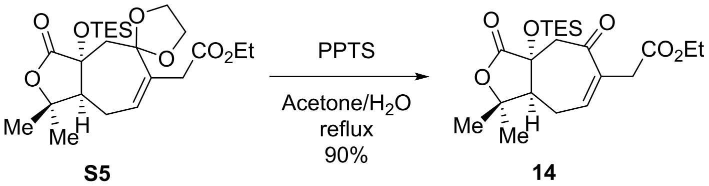

The solution of **S5** (21.032 g, 46 mmol) in acetone/H_2O (400 mL/40 mL) was added by PPTS (1.163 g, 4.6 mmol), stirred under reflux for 6 h and cooled to room temperature. Another 200 mL H_2O was added before the acetone was removed under vacuum, and the resultant aqueous phase was extracted with dichloromethane (100 mL×3). The combined organic phase was washed by

brine (100 mL), dried over Na_2SO_4 and concentrated in vacuo. The desired product could crystallize from petroleum ether/dichloromethane, or be purified by column chromatography (petroleum ether/ethyl acetate, 4:1 to 2:1) to give **14** (17.139 g, 90%) as white solid. R_f = 0.25 (silica gel, petroleum ether/ethyl acetate, 2:1); $[\alpha]_D^{20}$ = + 44.1 (*c* = 1.93 in $CHCl_3$); ^{1}H NMR (400 MHz, $CDCl_3$): δ = 6.78-6.73 (m, 1H), 4.13 (q, *J* = 7.1 Hz, 2H), 3.35 (d, *J* = 16.2 Hz, 1H), 3.19 (d, *J* = 14.6 Hz, 1H), 3.01 (d, *J* = 16.2 Hz, 1H), 2.84 (d, *J* = 14.6 Hz, 1H), 2.62-2.47 (m, 3H), 1.56 (s, 3H), 1.37 (s, 3H), 1.25 (t, *J* = 7.1 Hz, 3H), 0.89 (t, *J* = 7.9 Hz, 9H), 0.66 (q, *J* = 7.8 Hz, 6H) ppm; ^{13}C NMR (100 MHz, $CDCl_3$): δ = 195.1, 174.8, 171.3, 142.3, 138.9, 84.0, 79.3, 61.0, 56.2, 50.0, 38.0, 30.3, 27.1, 25.1, 14.2, 7.0, 5.9 ppm; IR (neat): ν_{max} = 2948, 2874, 1758, 1734, 1663, 1187, 1024 cm^{-1}; HRMS (ESI): *m/z* calcd for $C_{21}H_{34}NaO_6Si$ $[M+Na]^+$: 433.2017, found 433.2008.

Synthesis of compound S6.

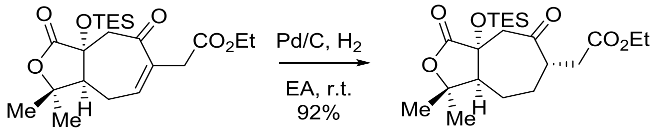

To the solution of **14** (12.583 g, 31 mmol) in ethyl acetate (120 mL) was added Pd/C (5% Pd, 1.26 g, $m_{sub}\times0.1$), and the mixture was degassed with H_2 for 5 times. After stirring for 4 h at 25 ^oC, the suspension was filtered with celite and eluted with ethyl acetate. After the eluent was removed under vacuum, the residue could directly move to the next step or be further purified by chromatography column (petroleum ether/ethyl acetate, 5:1 to 3:1) to give **S6** (11.67 g, 92%) as white solid. R_f = 0.3 (silica gel, petroleum ether/ethyl acetate, 4:1); $[\alpha]_D^{20}$ = -8.5 (*c* = 1.86 in $CHCl_3$); ^{1}H NMR (400 MHz, $CDCl_3$): δ = 4.14 (q, *J* = 7.1 Hz, 2H), 2.91 (d, *J* = 12.3 Hz, 1H), 2.91-2.83 (m, 2H), 2.81 (d, *J* = 12.3 Hz, 1H), 2.45 (dd, *J* = 12.5, 6.0 Hz, 1H), 2.16-2.03 (m, 2H), 1.96 (ddd, *J* = 13.2, 8.4, 4.4 Hz, 1H), 1.86 (q, *J* = 12.7 Hz, 1H), 1.61 (q, *J* = 12.1 Hz, 1H), 1.50 (s, 3H), 1.34 (s, 3H), 1.26 (t, *J* = 7.1 Hz, 3H), 0.91 (t, *J* = 7.8 Hz, 9H), 0.70-0.63 (m, 6H) ppm; ^{13}C NMR (100 MHz, $CDCl_3$): δ = 203.9, 175.6, 172.7, 83.6, 78.1, 60.7, 58.1, 52.2, 49.2, 35.7, 29.9, 27.1, 25.6, 24.7, 14.3, 7.2, 6.1 ppm; IR (neat): ν_{max} = 2947, 2872, 1765, 1731, 1711, 1267, 1184, 1088, 1001 cm^{-1}; HRMS (ESI): *m/z* calcd for $C_{21}H_{36}NaO_6Si$ $[M+Na]^+$: 435.2173, found 435.2182.

Synthesis of compound 15.

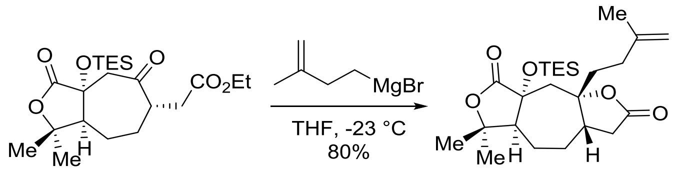

The solution of **S6** (6.933 g, 16.8 mmol) in THF (60 mL) was protected under N_2 and cooled to -23 ^oC. While vigorously stirring, freshly prepared Grignard reagent (50 mL, 1.0 mol/L in Et_2O, 50 mmol) was added within 5 min. After stirring at the same temperature for another 15 min, the reaction was quenched by NH_4Cl (sat. aq., 50 mL). The aqueous phase was extracted with dichloromethane (25 mL×3) and the combined organic phase was washed by brine (50 mL), dried over Na_2SO_4 and concentrated in vacuo. The residue could directly move to the next step.

Alternatively, the desired product could crystallize from petroleum ether/dichloromethane and be purified by column chromatography (petroleum ether/dichloromethane/ethyl acetate, 200:200:1 to 50:50:1) to give **15** (5.881 g, 80%) as white solid. R_f = 0.5 (silica gel, petroleum ether/dichloromethane/ethyl acetate, 20:20:1); $[\alpha]_D^{20}$ = -14.7 (*c* = 1.13 in $CHCl_3$); ^{1}H NMR (400 MHz, $CDCl_3$): δ = 4.72 (s, 1H), 4.66 (s, 1H), 2.86 (dd, *J* = 17.9, 9.0 Hz, 1H), 2.64 (d, *J* = 15.6 Hz, 1H), 2.47-2.40 (m, 2H), 2.25 (dd, *J* = 17.9, 2.8 Hz, 1H), 2.11-2.07 (m, 2H), 2.00-1.91 (m, 2H), 1.81-1.63 (m, 4H), 1.71 (s, 3H), 1.48-1.39 (m, 1H), 1.46 (s, 3H), 1.29 (s, 3H), 0.95 (t, *J* = 7.8 Hz, 9H), 0.78-0.64 (m, 6H) ppm; ^{13}C NMR (100 MHz, $CDCl_3$): δ = 176.6, 175.1, 144.4, 110.5, 88.5, 83.0, 79.8, 57.7, 43.8, 42.0, 39.7, 36.7, 31.6, 29.9, 27.3, 24.4, 23.4, 22.6, 7.2, 6.0 ppm; IR (neat): ν_{max} = 2949, 2936, 2873, 1762, 1649, 1456, 1375, 1275, 1239, 1204, 1143, 1116, 1101, 1010, 942, 911, 884, 728 cm^{-1}; HRMS (ESI): *m/z* calcd for $C_{24}H_{41}O_5Si$ $[M+H]^+$: 437.2716, found 437.2718.

Synthesis of compound S7.

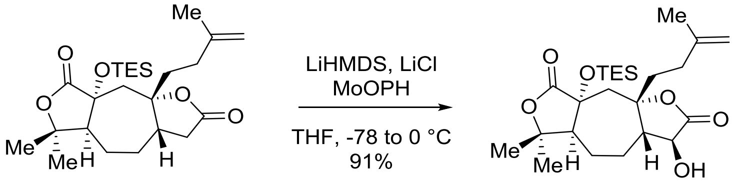

15 **S7**

To the suspension of **15** (4.989 g, 11.4 mmol) and LiCl (965 mg, 22.8 mmol) in THF (110 mL) was added LiHMDS (22.8 mL, 1.0 mol/L in THF, 22.8 mmol) at -78 °C under N_2 protection. The mixture was slowly warmed to 0 °C over 1 h, stirred for another 0.5 h and cooled to -78 °C again. Freshly prepared Vedejs' reagent (7.442 g, 17.1 mmol) was added in one portion, and the resultant mixture was stirred for another 1 h and then quenched by NH_4Cl (sat. aq., 50 mL) and Na_2SO_3 (sat. aq., 10 mL) *in situ*. The aqueous phase was extracted with dichloromethane (20 mL×3) and the combined organic phase was washed by brine (40 mL), dried over Na_2SO_4 and concentrated in vacuo. The residue could directly move to the next step, or be purified by column chromatography (petroleum ether/ethyl acetate, 3:1 to 1:1) to give **S7** (4.705 g, 91%) as white solid. R_f = 0.1 (silica gel, petroleum ether/ethyl acetate, 4:1); $[\alpha]_D^{20}$ = -22.9 (*c* = 0.73 in $CHCl_3$); ^{1}H NMR (400 MHz, $CDCl_3$): δ = 4.75-4.73 (m, 2H), 4.67 (s br, 1H), 2.77-2.75 (m, 1H), 2.55 (ddd, *J* = 9.8, 7.2, 3.4 Hz, 1H), 2.50-2.46 (m, 2H), 2.18-1.84 (m, 7H), 1.79-1.66 (m, 2H), 1.72 (s, 3H), 1.48 (s, 3H), 1.40 (s, 3H), 0.94 (t, *J* = 8.0 Hz, 9H), 0.76-0.60 (m, 6H) ppm; ^{13}C NMR (100 MHz, $CDCl_3$): δ = 176.34, 176.31, 144.3, 110.5, 87.0, 83.9, 80.1, 70.8, 55.0, 48.6, 42.0, 31.0, 30.4, 23.6, 22.7, 22.5, 21.1, 7.0, 5.7 ppm; IR (neat): ν_{max} = 3438, 2950, 2876, 1764, 1457, 1372, 1276, 1239, 1102, 1006 cm^{-1}; HRMS (ESI): *m/z* calcd for $C_{24}H_{40}NaO_6Si$ $[M+Na]^+$: 475.2486, found 475.2485.

Synthesis of compound 16.

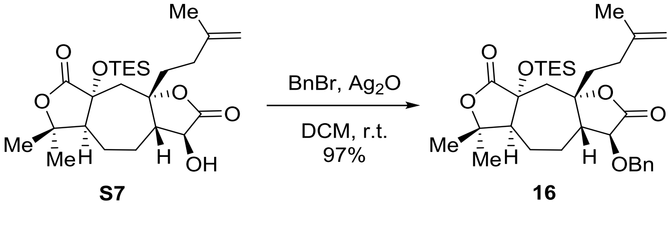

S7 **16**

To the solution of **S7** (2.783 g, 6.2 mmol) in dichloromethane (20 mL) was added BnBr (2.110 g,

12.3 mmol) and Ag_2O (2.848 g, 12.3 mmol). The mixture was protected by N_2 and warmed to 35 °C for 24 h. After cooled to room temperature, the mixture was filtered with a short pad of celite and eluted with ethyl acetate. After concentration in vacuo, the residue was purified by column chromatography (toluene/dichloromethane/ethyl acetate, 100:100:1, to elute Bn_2O and BnBr, and then petroleum ether/ethyl acetate, 50:1 to 20:1) to give **16** (3.233 g, 97%) as white solid. R_f = 0.6 (silica gel, petroleum ether/dichloromethane/ethyl acetate, 8:1:2); $[\alpha]_D^{20}$ = -70.5 (*c* = 0.93 in $CHCl_3$); ^{1}H NMR (400 MHz, $CDCl_3$): δ = 7.40-7.29 (m, 5H), 5.12 (d, *J* = 11.9 Hz, 1H), 4.75 (d, *J* = 11.9 Hz, 1H), 4.72 (s, 1H), 4.66 (s, 1H), 4.37 (d, *J* = 8.6 Hz, 1H), 2.58 (td, *J* = 8.3, 3.1 Hz, 1H), 2.47 (d, *J* = 15.9 Hz, 1H), 2.32 (t, *J* = 6.2 Hz, 1H), 2.10 (t, *J* = 8.3 Hz, 2H), 1.94-1.75 (m, 6H), 1.70 (s, 3H), 1.56-1.46 (m, 1H), 1.42 (s, 3H), 1.34 (s, 3H), 0.95 (t, *J* = 7.8 Hz, 9H), 0.77-0.61 (m, 6H) ppm; ^{13}C NMR (100 MHz, $CDCl_3$): δ = 176.5, 174.5, 144.5, 137.8, 128.6, 128.24, 128.18, 110.6, 86.6, 83.8, 80.2, 77.0, 72.4, 55.5, 48.1, 42.4, 41.8, 31.3, 30.5, 23.9, 22.81, 22.78, 21.7, 7.3, 5.9 ppm; IR (neat): ν_{max} = 2947, 2872, 1765, 1453, 1271, 1238, 1101, 1010, 885 cm^{-1}; HRMS (ESI): *m/z* calcd for $C_{31}H_{47}O_6Si$ $[M+H]^+$: 543.3136, found 543.3130. CCDC 1508989 contains the supplementary crystallographic data for compound **16** and is available free of charge from The Cambridge Crystallographic Data Centre via www.ccdc.cam.ac.uk/data_request/cif.

Synthesis of compound 18.

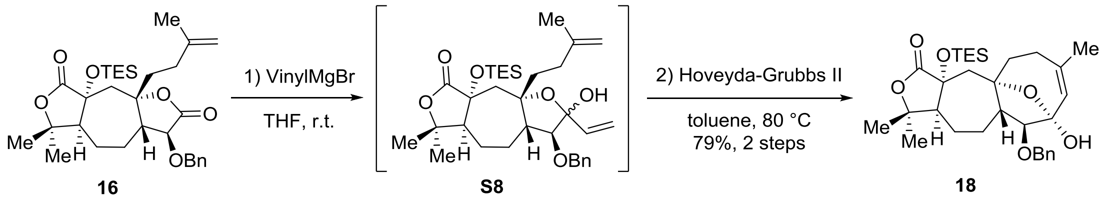

To the solution of **16** (2.797 g, 5.2 mmol) in THF (52 mL) was added vinylmagnesium bromide (7.7 mL, 1.0 mol/L in THF, 7.7 mmol) at 25 °C. After stirring for another 1 h, the reaction was quenched by saturated solution of NH_4Cl (sat. aq., 20 mL). The aqueous phase was extracted with dichloromethane (10 mL×3) and the combined organic phase was washed by brine (20 mL), dried over Na_2SO_4 and concentrated in vacuo. The residue was filtered with a short pad of silica gel and eluted with petroleum ether/ethyl acetate (4:1). The eluent was removed under vacuum and the residue moved to the next step directly without further purification.

The residue above was dissolved in toluene (520 mL) and added by Hoveyda-Grubbs catalyst 2nd generation (161 mg, 0.26 mmol). The mixture was degassed with N_2 and stirred at 80 °C. After 4 h and 8 h, the mixture was cooled, degassed with N_2 and heated again. 18 h later in all, the reaction was stopped by cooling to room temperature, and the mixture was directly purified by column chromatography (petroleum ether/dichloromethane/ethyl acetate, 20:5:1 to 20:5:1.5) to give **18** (2.217 g, 79% for 2 steps) as yellowish oil, with some **S8** recovered in addition. Characterization data for **18**: R_f = 0.35 (silica gel, petroleum ether/dichloromethane/ethyl acetate, 8:8:1); $[\alpha]_D^{20}$ = -59.5 (*c* = 1.1 in CH_3OH); ^{1}H NMR (400 MHz, CD_3OD): δ = 7.34-7.25 (m, 5H), 5.54 (s br, 1H), 4.76 (d, *J* = 11.7 Hz, 1H), 4.55 (d, *J* = 11.7 Hz, 1H), 3.58 (d, *J* = 8.0, 1H), 2.47-2.34 (m, 3H), 2.26-2.15 (m, 3H), 2.04-1.89 (m, 3H), 1.79 (s, 3H), 1.66 (dd, *J* = 14.0, 5.8 Hz, 1H), 1.61 (td, *J* = 12.0, 8.8 Hz, 1H), 1.54 (s, 3H), 1.30 (s, 3H), 1.20 (td, *J* = 13.6, 9.2 Hz, 1H), 0.98 (t, *J* = 8.0 Hz, 9H), 0.73 (q, *J* = 8.0 Hz, 6H) ppm; ^{13}C NMR (100 MHz, CD_3OD): δ = 178.0, 140.6, 139.9, 129.3, 128.9, 128.6, 128.1, 104.7, 92.0, 86.5, 82.4, 80.9, 73.2, 58.6, 51.0, 46.0, 41.3, 31.0, 30.5, 27.3, 26.9, 25.6, 24.8, 7.5, 7.3 ppm; IR (neat): ν_{max} = 3393, 2953, 2933, 2910, 2873, 1767, 1669, 1455, 1392,

1375, 1271, 1169, 1076, 734 cm^{-1}; HRMS (ESI): *m/z* calcd for $C_{31}H_{47}O_6Si$ $[M+H]^+$: 543.3136, found 543.3121.

Synthesis of compound S9.

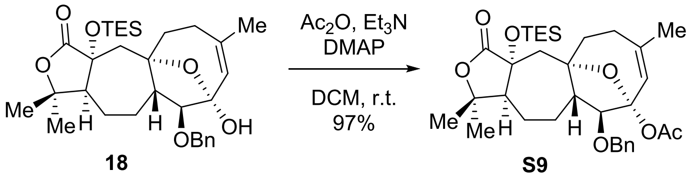

To a solution of **18** (2.34 g, 4.31 mmol) in 43 ml DCM was added by Et_3N (3.0 ml, 21.57 mmol), DMAP (264 mg, 2.16 mmol) and Ac_2O (0.83 ml, 8.64 mmol) subsequently. After stirring for 24 h, the reaction was quenched with NH_4Cl (sat. aq., 10 mL). The mixture was separated and the aqueous layer was extracted with ethyl acetate (20 mL×3). The combined organic layer was washed with a saturated aqueous solution of $NaHCO_3$ (20 mL) and brine (20 mL), dried over Na_2SO_4 and concentrated in vacuo. Purification by silica gel column chromatography (petroleum ether/ethyl acetate 20:1) afforded **S9** (2.44g, 4.18 mmol, 97%) as white solid. R_f = 0.4 (silica gel, petroleum ether/ethyl acetate = 4:1); $[\alpha]_D^{20}$ = –40.7 (*c* = 0.73 in $CHCl_3$); ^{1}H NMR (400 MHz, $CDCl_3$): δ = 7.37-7.27 (m, 5H), 5.63 (s, 1H), 4.72 (d, *J* = 11.8 Hz, 1H), 4.52 (d, *J* = 11.9 Hz, 1H), 3.91 (d, *J* = 5.7 Hz, 1H), 2.50 (d, *J* = 15.3 Hz, 1H), 2.47-2.41 (m, 1H), 2.37 (dd, *J* = 12.2, 5.4 Hz, 1H), 2.20-2.13 (m, 2H), 2.07 (s, 3H), 1.96-1.77 (m, 5H), 1.86 (d, *J* = 15.2 Hz, 1H), 1.80 (s, 3H), 1.47 (s, 3H), 1.32-1.21 (m, 1H), 1.25 (s, 3H), 0.94 (t, *J* = 7.9 Hz, 9H), 0.71 (dd, *J* = 8.5, 7.5 Hz, 6H) ppm; ^{13}C NMR (100 MHz, $CDCl_3$): δ = 176.7, 168.7, 140.3, 138.6, 128.5, 127.9, 127.8, 124.4, 108.3, 91.9, 83.6, 83.4, 80.3, 72.7, 58.3, 51.1, 43.7, 41.9, 30.2, 30.1, 26.8, 26.3, 24.7, 24.4, 22.3, 7.4, 6.2 ppm; IR (neat): ν_{max}= 2947, 2868, 2363, 2333, 1765, 1731, 1449, 1375, 1232, 1163, 1101, 1018, 732 cm^{-1}; HRMS (ESI, *m/z*): $[M+H]^+$ calcd for $C_{33}H_{49}O_9Si$, 585.3242; found 585.3258.

Synthesis of compound 19.

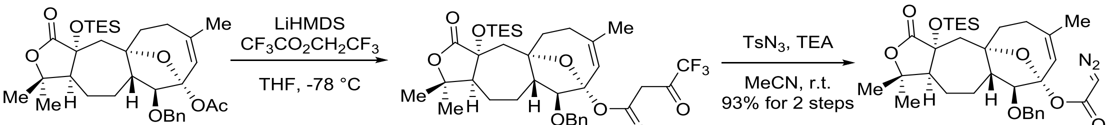

Compound **S9** (1.72g, 2.94mmol) was dissolved into 30mL THF, then cooled down to -78 ^{o}C, stirred for 10 min. Then 7.35 mL LiHMDS (1 M, THF solution) was added slowly into the system and the mixture was stirred at -78 ^{o}C for 1 h. After that, $CF_3CO_2CH_2CF_3$ (0.6 ml, 4.4 mmol) was slowly added at -78 ^{o}C, then the mixture was stirred for additional 30 min at -78 ^{o}C and stirred at 0 ^{o}C for 1 h. When the reaction completed, NH_4Cl (sat. aq., 20 mL) was added to quench the reaction. The mixture was separated and the aqueous layer was extracted with ethyl acetate (20 mL×3). The combined organic layer was washed with brine (10 mL), dried over Na_2SO_4 and concentrated in vacuo. Purification by silica gel column chromatography (petroleum ether/ethyl acetate 20:1) afforded crude **S10**.

The crude **S10** was dissolved into 30 mL MeCN. To the solution was added by Et_3N (1.24 mL, 8.82 mmol) and TsN_3 (0.81g, 4.11 mmol) subsequently. The mixture was stirred for 24 h and concentrated in vacuo. Purification by silica gel column chromatography (petroleum ether/ethyl acetate 30:1 to 10:1) afforded **19** (1.67 g, 2.73 mmol, 93% for 2 steps) as light yellow solid.

Characterization data for **19**: R_f = 0.35 (silica gel, petroleum ether/ethyl acetate = 4:1); $[\alpha]_D^{20}$ = –22.5 (c = 1.08 in $CHCl_3$); ^{1}H-NMR (500 MHz, $CDCl_3$): δ = 7.36-7.27 (m, 5H), 5.66 (s, 1H), 4.78 (br, 1H), 4.70 (d, J = 11.8 Hz, 1H), 4.48 (d, J = 11.8 Hz, 1H), 3.94 (d, J = 6.3 Hz, 1H), 2.51 (d, J = 15.2 Hz, 1H), 2.45 (dt, J = 17.3, 5.7 Hz, 1H), 2.36 (dd, J = 12.1, 6.0 Hz, 1H), 2.23-2.16 (m, 2H), 2.01-1.85 (m, 3H), 1.91 (d, J = 15.3, 1H), 1.82 (d, J = 1.4 Hz, 3H), 1.84-1.80 (m, 1H), 1.78-1.75 (m, 1H), 1.47 (s, 3H), 1.29-1.22 (m, 1H), 1.26 (s, 3H), 0.95 (t, J = 7.9, 9H), 0.70 (q, J = 7.8 Hz, 6H) ppm; ^{13}C-NMR (125 MHz, $CDCl_3$): δ = 176.5, 140.1, 138.4, 128.5, 127.81, 127.80, 124.5, 108.4, 91.8, 83.6, 83.5, 80.3, 72.6, 58.1, 50.3, 47.1, 44.0, 41.9, 30.24, 30.20, 26.8, 26.2, 24.7, 24.2, 7.3, 6.2 ppm; IR (neat): ν_{max} = 2951, 2868, 2110, 1764, 1711, 1368, 1143, 1104, 905, 734 cm^{-1}; HRMS (ESI, *m/z*): $[M+Na]^+$ calcd for $C_{33}H_{46}N_2O_7SiNa$, 633.2967; found 633.2982.

Synthesis of compound 20.

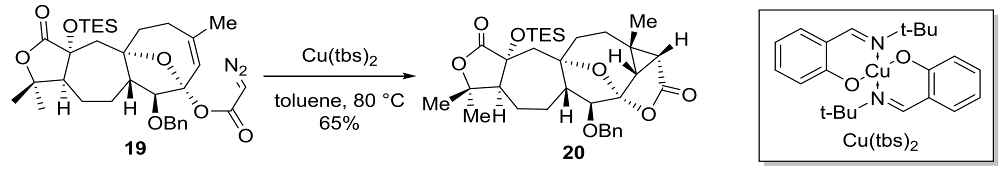

$Cu(tbs)_2$ (20 mg,0.048 mmol) was dissolved in 4 mL toluene and the mixture was heated to 80 °C. The solution of **19** (300 mg, 0.49 mmol) in 9 mL toluene was added in a drop-wise manner over 4 h, then stirred for additional 0.5 h at the same temperature. After cooling to room temperature, the mixture was concentrated in vacuo. Purification by silica gel column chromatography (petroleum ether/ethyl acetate 30:1 to 10:1) afforded **20** (186 mg, 0.32 mmol, 65% yield) as light yellow solid. R_f = 0.3 (silica gel, petroleum ether/ethyl acetate = 4:1); $[\alpha]_D^{20}$ = –13.9 (c = 0.36 in $CHCl_3$); IR (neat): 2952, 2869, 1773, 1453, 1115, 1100, 1063, 1007, 912, 731 cm^{-1}; ^{1}H NMR (500 MHz, $CDCl_3$): δ = 7.39-7.29 (m, 5H), 4.79 (d, J = 11.8 Hz, 1H), 4.56 (d, J = 11.8 Hz, 1H), 3.92 (d, J = 9.5 Hz, 1H), 2.71 (d, J = 7.3 Hz, 1H), 2.72-2.66 (m, 1H), 2.50 (td, J = 9.8, 5.2 Hz, 1H), 2.28 (dd, J = 10.6, 5.9 Hz, 1H), 2.27-2.23 (m, 1H), 2.18-2.06 (m, 3H), 1.91 (d, J = 7.3 Hz, 1H), 1.90-1.81 (m, 2H), 1.66-1.58 (m, 2H), 1.51 (s, 3H), 1.33 (s, 3H), 1.24-1.18 (m, 1H), 1.06 (s, 3H), 0.92 (t, J = 7.9 Hz, 9H), 0.67 (q, J = 7.9 Hz, 6H); ^{13}C NMR (125 MHz, $CDCl_3$): δ = 176.1, 173.3, 137.6, 128.6, 128.2, 128.0, 109.3, 84.7, 80.6, 80.2, 74.4, 72.5, 58.3, 56.9, 44.8, 44.7, 43.5, 36.1, 35.5, 30.4, 28.2, 27.3, 24.7, 24.4, 23.1, 7.2, 6.3 ppm; HRMS (ESI, *m/z*): $[M+H]^+$ calcd for $C_{33}H_{47}O_7Si$, 583.3086; found 583.3082.

Synthesis of compound S11.

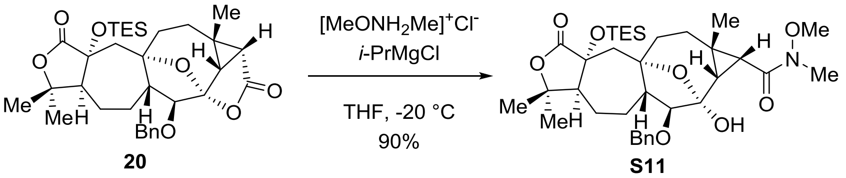

To a mixture of **20** (836 mg, 1.43 mmol), *N, O*-dimethylhydroxylamine hydrochloride (700 mg, 7.18 mmol) in 14 mL THF was added by 7.18 mL *iso*-propylmagnesium chloride (2M, THF solution) at -20 °C. After stirred for 10 min, reaction was quenched by a saturated solution of NH_4Cl (5 mL). The mixture was separated and aqueous layer was extracted with ethyl acetate (10 mL×3). The combined organic layer was washed with brine (5 mL), dried over Na_2SO_4 and

concentrated *in vacuo*. Purification by silica gel column chromatography (petroleum ether/ethyl acetate 20:1) afforded **S11** (811 mg, 1.29 mmol, 90% yield) as light yellow solid. R_f = 0.2 (silica gel, petroleum ether/ethyl acetate = 4:1); $[\alpha]_D^{20}$ = +27.0 (c = 1.25 in $CHCl_3$); IR (neat): ν_{max} = 2942, 2867, 1760, 1632, 1458, 1383, 1097, 1055 cm^{-1}; ^1H NMR (500 MHz, $CDCl_3$): δ = 7.39-7.27 (m, 5H), 7.10 (s, 1H), 4.95 (d, J = 11.9 Hz, 1H), 4.65 (d, J = 12.0 Hz, 1H), 3.72 (s, 3H), 3.54 (d, J = 9.6 Hz, 1H), 3.26 (s, 3H), 2.34 (d, J = 14.3 Hz, 1H), 2.28 (dd, J = 13.0, 6.0 Hz, 1H), 2.23 (d, J = 14.3 Hz, 1H), 2.17-2.06 (m, 3H), 1.96-1.79 (m, 2H), 1.86 (d, J = 8.7 Hz, 1H), 1.69 (d, J = 8.9 Hz, 1H), 1.62-1.42 (m, 2H), 1.54 (s, 3H), 1.46 (d, J = 15.1 Hz, 1H), 1.30 (s, 3H), 1.28-1.23 (m, 1H), 1.21 (s, 3H), 0.93 (t, J = 7.9 Hz, 9H), 0.68 (q, J = 7.7 Hz, 6H); ^{13}C NMR (125 MHz, $CDCl_3$): δ = 176.0, 172.9, 138.9, 128.4, 127.9, 127.6, 104.1, 86.6, 84.8, 81.1, 72.1, 61.3, 57.6, 53.7, 45.5, 37.1, 32.7, 31.3, 30.5, 30.4, 26.8, 26.2, 25.9, 25.4, 24.9, 23.5, 7.2, 6.5 ppm; HRMS (ESI, *m/z*): $[M+H]^+$ calcd for $C_{35}H_{54}NO_8Si$, 644.3613; found 644.3625.

Synthesis of compound S12.

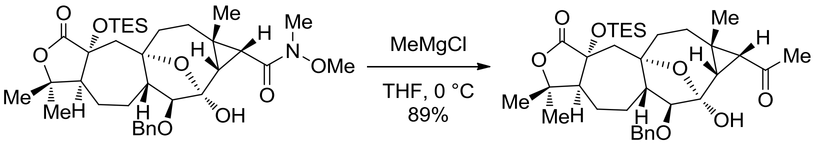

To a solution of **S11** (113 mg, 0.18 mmol) in 2 mL THF was added by methylmagnesium chloride (0.18 mL, 3 M in THF solution) at 0 °C. After stirred for 3h, the reaction mixture was added in drops into a saturated solution of NH_4Cl (2 mL). The mixture was separated and aqueous layer was extracted with ethyl acetate (5 mL×3). The combined organic layer was washed with brine (2 mL), dried over Na_2SO_4 and concentrated in *vacuo*. Purification by silica gel column chromatography (petroleum ether/ethyl acetate 10:1) afforded **S12** (96 mg, 0.16 mmol, 89% yield) as colorless oil. R_f = 0.25 (silica gel, petroleum ether/ethyl acetate = 4:1); $[\alpha]_D^{20}$ = +3.1 (c = 1.0 in $CHCl_3$); IR (neat): ν_{max} = 2945, 2874, 1767, 1690, 1454, 1365, 1107, 1061 cm^{-1}; ^1H NMR (400 MHz, $CDCl_3$): δ = 7.38-7.27 (m, 5H), 6.03 (s, 1H), 4.89 (d, J = 12.0 Hz, 1H), 4.63 (d, J = 12.0 Hz, 1H), 3.58 (d, J = 9.5 Hz, 1H), 2.30 (s, 3H), 2.29-2.25 (m, 1H), 2.22 (s, 2H), 2.15-1.97 (m, 3H), 1.87 (d, J = 8.4 Hz, 1H), 1.86-1.78 (m, 2H), 1.68 (d, J = 8.4 Hz, 1H), 1.65-1.54 (m, 2H), 1.52 (s, 3H), 1.49-1.42 (m, 1H), 1.30 (s, 3H), 1.29-1.26 (m, 1H), 1.25 (s, 3H), 0.93 (t, J = 7.9 Hz, 9H), 0.68 (q, J = 7.9 Hz, 6H) ppm; ^{13}C NMR (100 MHz, $CDCl_3$): δ = 209.3, 176.1, 138.7, 128.4, 127.9, 127.8, 104.1, 86.5, 84.7, 80.9, 78.0, 72.1, 57.4, 53.8, 45.4, 40.4, 37.5, 32.5, 31.3, 30.3, 28.2, 26.3, 25.9, 25.2, 24.8, 23.3, 7.2, 6.4 ppm; HRMS (ESI, *m/z*): $[M+H]^+$ calcd for $C_{34}H_{51}O_7Si$, 599.3399; found 599.3388.

Synthesis of compound 21.

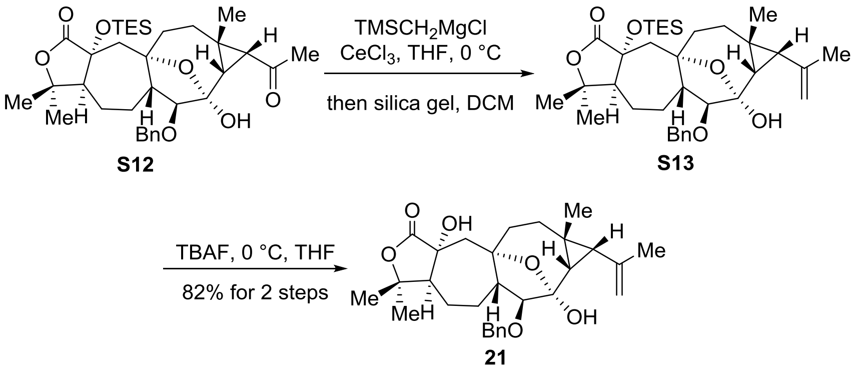

To an anhydrous cerium (III) chloride (216 mg, 0.876 mmol) was added by THF (2 mL) at 0 °C, then the suspension was stirred at room temperature for 3 h. After that, $TMSCH_2MgCl$ (0.88 ml, 1M, Et_2O solution) was added to the suspension at 0 °C and stirred for 1 h at this temperature. A solution of **S12** (105 mg, 0.18 mmol) in 2 mL THF was added and stirred for 3 h. The reaction was quenched by a saturated solution of NH_4Cl (2 mL). The mixture was separated and aqueous layer was extracted with ethyl acetate (5 mL×3). The combined organic layer was washed with brine (2 mL), dried over Na_2SO_4 and concentrated in *vacuo*. The residue was dissolved in 2 mL DCM and 630 mg silica gel was added. The suspension was stirred for 12 hours, filtered and washed with ethyl acetate (5 mL×3). The solution was concentrated in vacuo to afford **S13** as a colorless oil.

The oil was dissolved in 2 mL THF and TBAF•$3H_2O$ (170 mg, 0.54 mmol) was added at 0 °C. After stirred for 10 min, The reaction was quenched by a saturated solution of NH_4Cl (2 mL). The mixture was separated and aqueous layer was extracted with ethyl acetate (5 mL×3). The combined organic layer was washed with brine (2 mL), dried over Na_2SO_4 and concentrated in vacuo. Purification by silica gel column chromatography (petroleum ether/ethyl acetate 5:1) afforded **21** (71 mg, 0.15 mmol, 82% yield for 2 steps) as white solid. Characterization data for **21**: R_f = 0.3 (silica gel, petroleum ether/ethyl acetate = 2:1); $[\alpha]_D^{20}$ = +10.7 (c = 0.73 in $CHCl_3$); IR (neat): ν_{max} = 3403, 2928, 2863, 1766, 1454, 1374, 1269, 1096, 1029, 911, 734 cm^{-1}; ^1H NMR (500 MHz, $CDCl_3$): δ = 7.37-7.27 (m, 5H), 5.88 (s, 1H), 5.05 (s, 1H), 5.03 (s, 1H), 4.69 (d, J = 11.8 Hz, 1H), 4.64 (d, J = 11.8 Hz, 1H), 3.87 (d, J = 4.0 Hz, 1H), 3.34 (s, 1H), 2.49 (dd, J = 9.4, 6.5 Hz, 1H), 2.31 (d, J = 15.5 Hz, 1H), 2.19 (dt, J = 10.6, 4.3 Hz, 1H), 2.10 (ddd, J = 14.1, 12.4, 2.0 Hz, 1H), 1.95-1.78 (m, 4H), 1.86 (s, 3H), 1.73 (d, J = 15.5 Hz, 1H), 1.54-1.44 (m, 2H), 1.42 (s, 3H), 1.40-1.36 (m, 2H), 1.32-1.29 (m, 1H), 1.28 (s, 3H), 1.13 (s, 3H); ^{13}C NMR (125 MHz, $CDCl_3$): δ = 177.5, 140.8, 138.2, 128.5, 127.8, 127.6, 113.9, 106.2, 92.0, 85.8, 83.2, 79.3, 72.9, 55.3, 53.3, 40.3, 39.0, 35.2, 33.7, 30.0, 28.1, 26.3, 26.1, 25.3, 24.5, 23.8, 23.5 ppm; HRMS (ESI, m/z): $[M+H]^+$ calcd for $C_{29}H_{39}O_6$, 483.2741; found 483.2746. CCDC 1509314 contains the supplementary crystallographic data for compound **21** and is available free of charge from The Cambridge Crystallographic Data Centre via www.ccdc.cam.ac.uk/data_request/cif.

Synthesis of compound S14.

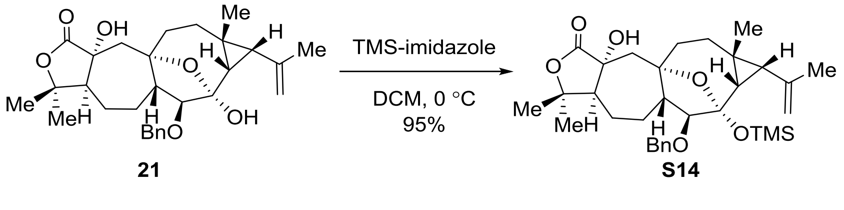

To a solution of **21** (72 mg, 0.15 mmol) in 1.5 mL DCM was added by TMS-imidazole (0.22 mL, 1.49 mmol) at 0 °C. Then the mixture was stirred for 24 h, the reaction was quenched by a saturated solution of NH_4Cl (2 mL). The mixture was separated and aqueous layer was extracted with ethyl acetate (5 mL×3). The combined organic layer was washed with brine (2 mL), dried over Na_2SO_4 and concentrated in vacuo. Purification by silica gel column chromatography (petroleum ether/ethyl acetate 15:1) afforded **S14** (79 mg, 0.14 mmol, 95% yield) as colorless oil. R_f = 0.65 (silica gel, petroleum ether/ethyl acetate = 2:1); $[\alpha]_D^{20}$ = +28.1 (*c* = 0.95 in $CHCl_3$); IR (neat): ν_{max} = 3411, 2938, 2863, 1769, 1449, 1371, 1250, 1100, 836 cm^{-1}; ^1H NMR (500 MHz, $CDCl_3$): δ = 7.37-7.29 (m, 5H), 6.36 (s, 1H), 5.19 (s, 1H), 5.05 (s, 1H), 4.63 (d, *J* = 11.3 Hz, 1H), 4.47 (d, *J* = 11.2 Hz, 1H), 3.69 (s, 1H), 2.55 (dd, *J* = 12.4, 6.1 Hz, 1H), 2.36 (d, *J* = 15.4 Hz, 1H), 2.15 (dd, *J* = 16.5, 12.9 Hz, 1H), 2.15 (d, *J* = 13.1 Hz, 1H), 1.99-1.92 (m, 2H), 1.80 (ddd, *J* = 14.5, 9.5, 5.0 Hz, 1H), 1.76 (s, 3H), 1.71 (t, *J* = 13.2 Hz, 1H), 1.62 (d, *J* = 15.3 Hz, 1H), 1.56-1.47 (m, 1H), 1.45 (s, 3H), 1.34-1.26(m, 3H), 1.24 (s, 3H), 1.22 (d, *J* = 8.6 Hz, 1H), 1.05 (s, 3H), 0.16 (s, 9H); ^{13}C NMR (125 MHz, $CDCl_3$): δ = 177.4, 139.1, 138.0, 128.5, 127.9, 127.6, 115.8, 107.6, 97.9, 86.3, 82.7, 79.2, 72.8, 57.0, 53.8, 39.2, 37.5, 37.0, 34.9, 30.1, 28.3, 28.2, 25.3, 25.1, 24.92, 24.90, 24.4, 1.6 ppm; HRMS (ESI, *m/z*): $[M+H]^+$ calcd for $C_{32}H_{47}O_6Si$, 555.3136; found 555.3252.

Synthesis of compound 22.

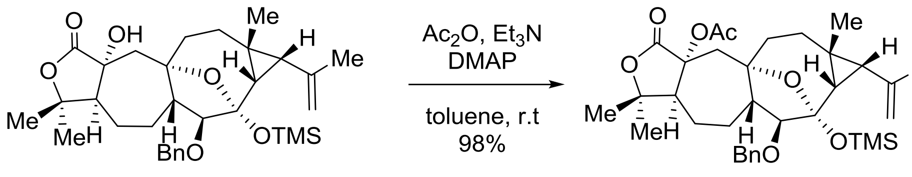

To a solution of **S14** (140 mg, 0.252 mmol) in 7 mL toluene was added by Et_3N (0.54 ml, 3.78 mmol), DMAP (31 mg, 0.25 mmol) and Ac_2O (0.36 ml, 3.78 mmol) subsequently. After stirring for 12h, the reaction was quenched with a saturated solution of NH_4Cl (5 mL). The mixture was separated and the aqueous layer was extracted with ethyl acetate (10 mL×3). The combined organic layer was washed with a saturated aqueous solution of $NaHCO_3$ (2 mL) and brine (2 mL), dried over Na_2SO_4 and concentrated in vacuo. Purification by silica gel column chromatography (petroleum ether/ethyl acetate 15:1) afforded **22** (147 mg, 0.247 mmol, 98%) as colorless oil. R_f = 0.3 (silica gel, petroleum ether/ethyl acetate = 4:1); $[\alpha]_D^{20}$ = +44.4 (*c* = 0.8 in $CHCl_3$); IR (neat): ν_{max} = 2946, 2855, 1772, 1735, 1366, 1242, 1105, 1039, 889, 840 cm^{-1}; ^1H NMR (500 MHz, $CDCl_3$): δ = 7.36-7.27 (m, 5H), 5.35 (s, 1H), 4.97 (s, 1H), 4.63 (d, *J* = 11.4 Hz, 1H), 4.48 (d, *J* = 11.4 Hz, 1H), 3.63 (s, 1H), 2.83 (dd, *J* = 12.7, 6.4 Hz, 1H), 2.43 (d, *J* = 15.3 Hz, 1H), 2.10-2.05 (m, 2H), 2.03 (s, 3H), 1.91-1.85 (m, 1H), 1.77 (s, 3H), 1.75-1.69 (m, 3H), 1.55-1.50 (m, 2H), 1.52 (s, 3H), 1.36 (dd, *J* = 14.0, 5.5 Hz, 1H), 1.29 (s, 3H), 1.27-1.22 (m, 1H), 1.24 (s, 1H), 1.19 (dd, *J* = 14.0, 6.1 Hz, 1H), 1.06 (s, 3H), 0.14 (s, 9H); ^{13}C NMR (125 MHz, $CDCl_3$): 174.3, 170.5, 139.1, 138.4, 128.4, 127.7, 127.4, 115.6, 106.6, 98.0, 83.5, 83.2, 83.0, 72.7, 54.7, 52.0, 40.8, 38.3, 36.9, 35.2, 29.1, 28.4, 28.1, 25.6, 25.5, 25.4, 25.1, 24.5, 21.0, 1.7 ppm; HRMS (ESI, *m/z*): $[M+H]^+$ calcd for $C_{34}H_{49}O_7Si$, 597.3242; found 597.3259.

Synthesis of compound 23.

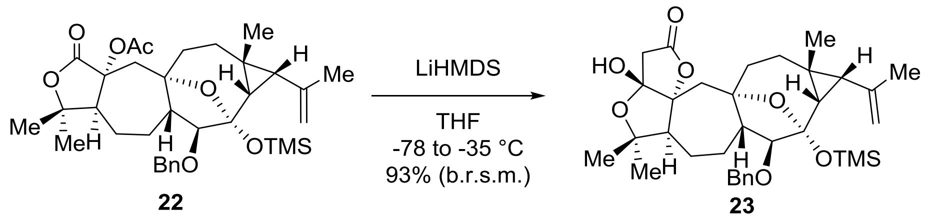

To a solution of **22** (311 mg, 0.521 mmol) in 11 mL THF was added by 1.6 mL LiHMDS (1M, in THF) at -78 °C. After stirring for 1h, the mixture was warmed to -35 °C and stirred for 18 h. The reaction was quenched with a saturated solution of NH_4Cl (5 mL). The mixture was separated and the aqueous layer was extracted with ethyl acetate (10 mL×3). The combined organic layer was washed with a saturated aqueous solution of $NaHCO_3$ (2 mL) and brine (2 mL), dried over Na_2SO_4 and concentrated in vacuo. Purification by silica gel column chromatography (petroleum ether/ethyl acetate 8:1) afforded **22** (40 mg, 0.073 mmol) and **23** (249 mg, 0.417 mmol, 93% b.r.s.m.) as white solid. R_f = 0.3 (silica gel, petroleum ether/ethyl acetate = 2:1); $[\alpha]_D^{20}$ = +19.3 (*c* = 0.97 in $CHCl_3$); IR (neat): ν_{max} = 3415, 2934, 2863, 1752, 1453, 1366, 1250, 1067, 840 cm^{-1}; ^1H NMR (500 MHz, $CDCl_3$): δ = 7.37-7.27 (m, 5H), 5.33 (s, 1H), 4.99 (s, 1H), 4.62 (d, *J* = 11.4 Hz, 1H), 4.49 (d, *J* = 11.4 Hz, 1H), 3.60 (s, 1H), 2.85 (d, *J* = 17.5 Hz, 1H), 2.69 (d, *J* = 17.4 Hz, 1H), 2.65 (s, 1H), 2.38 (dd, *J* = 13.0, 4.0 Hz, 1H), 2.21 (t, *J* = 13.0 Hz, 1H), 2.11 (d, *J* = 16.1 Hz, 1H), 2.06 (dd, *J* = 12.8, 5.7 Hz, 1H), 1.86-1.81 (m, 1H), 1.86 (d, *J* = 16.4 Hz, 1H), 1.75 (s, 3H), 1.74-1.60 (m, 3H), 1.50-1.42 (m, 1H), 1.34 (dd, *J* = 13.6, 5.9 Hz, 1H), 1.29 (s, 3H), 1.27-1.25 (m, 1H), 1.24 (s, 3H), 1.23 (s, 1H), 1.16 (dd, *J* = 13.8, 5.9 Hz, 1H), 1.07 (s, 3H), 0.17 (s, 9H); ^{13}C NMR (125 MHz, $CDCl_3$): δ = 171.9, 138.6, 138.4, 128.4, 127.6, 127.5, 116.3, 108.8, 106.9, 98.1, 97.7, 86.1, 82.7, 72.6, 60.4, 51.7, 42.6, 40.6, 37.4, 36.8, 35.4, 30.9, 30.1, 28.7, 25.8, 25.7, 25.0, 24.0, 1.9 ppm; HRMS (ESI, *m/z*): $[M+Na]^+$ calcd for $C_{34}H_{48}NaO_7Si$, 619.3062; found 619.3062.

Synthesis of compound 24.

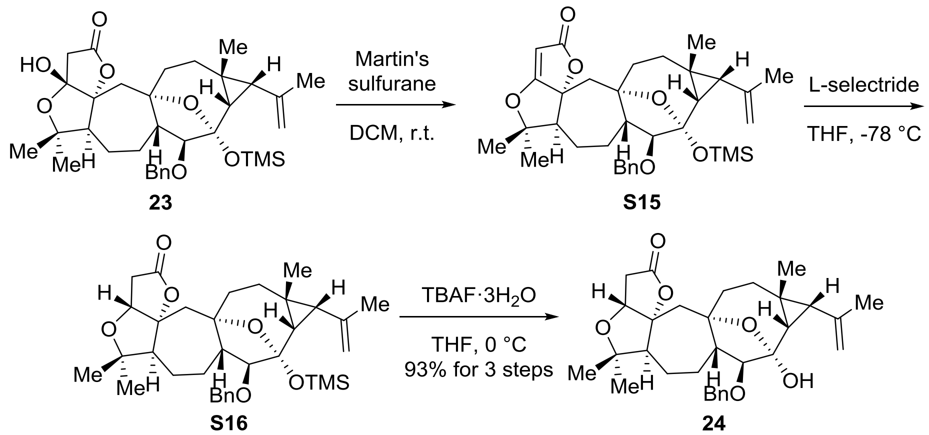

To a solution of **23** (82 mg, 0.137 mmol) in 2.5 mL DCM was added by Martin's sulfurane (111 mg, 0.165 mmol) at r.t. After stirring for 1h, the reaction was quenched with a saturated solution of $NaHCO_3$ (2 mL). The mixture was separated and the aqueous layer was extracted with ethyl acetate (5 mL×3). The combined organic layer was washed with brine (1 mL), dried over Na_2SO_4 and concentrated in vacuo to give crude **S15**. The crude product was dissolved into 2.5 mL THF, cooled to -78 °C, and added by L-selectride (0.42 mL, 1M, THF solution). The mixture was stirred for 1 h

and quenched with saturated solution of NH_4Cl (2 mL). The mixture was separated and the aqueous layer was extracted with ethyl acetate (5 mL×3). The combined organic layer was washed with brine (1 mL), dried over Na_2SO_4 and concentrated in vacuo to afford crude **S16**. To a solution of crude **S16** in 2.5 ml THF was added TBAF•$3H_2O$ (216 mg, 0.685 mmol) at 0 °C. After stirring for 10 min, the aqueous solution of NH_4Cl (2 mL) was added. The mixture was separated and the aqueous layer was extracted with ethyl acetate (5 mL×3). The combined organic layer was washed with brine (1 mL), dried over Na_2SO_4 and concentrated in vacuo. Purification by silica gel column chromatography (petroleum ether/ethyl acetate 4:1) afforded **24** (65 mg, 0.128 mmol, 93% for 3 steps) as colorless oil. Characterization data for **24**: R_f = 0.25 (silica gel, petroleum ether/ethyl acetate = 2:1); $[\alpha]_D^{20}$ = +20.7 (c = 0.95 in $CHCl_3$); IR (neat): ν_{max} = 3440, 2926, 2859, 1772, 1452, 1370, 1204, 1067, 906, 736 cm^{-1}; ^1H NMR (500 MHz, $CDCl_3$): δ = 7.37-7.27 (m, 5H), 5.20 (s, 1H), 4.98 (s, 1H), 4.70 (d, J = 11.8 Hz, 1H), 4.63 (d, J = 11.8 Hz, 1H), 4.12 (d, J = 5.6 Hz, 1H), 3.69 (d, J = 3.5 Hz, 1H), 2.92 (s, 1H), 2.70 (dd, J = 18.3, 5.7 Hz, 1H), 2.58 (d, J = 18.3 Hz, 1H), 2.20 (dd, J = 13.0, 4.6 Hz, 1H), 2.18-2.09 (m, 2H), 1.97-1.90 (m, 1H), 1.94 (d, J = 15.5 Hz, 1H), 1.83 (s, 3H), 1.81-1.76 (m, 1H), 1.75 (d, J = 15.5 Hz, 1H), 1.72-1.62 (m, 2H), 1.44-1.38 (m, 1H), 1.37 (d, J = 9.0 Hz, 1H), 1.35-1.30 (m, 2H), 1.28 (s, 3H), 1.27-1.22 (m, 1H), 1.13 (s, 3H), 1.07 (s, 3H); ^{13}C NMR (125 MHz, $CDCl_3$): δ = 174.9, 140.5, 138.5, 128.4, 127.7, 127.6, 114.3, 105.6, 96.7, 94.4, 84.2, 82.2, 81.1, 72.9, 60.1, 52.7, 43.3, 38.5, 35.5, 35.3, 33.8, 28.8, 28.6, 28.3, 26.3, 24.9, 24.5, 24.0, 21.9 ppm; HRMS (ESI, m/z): $[M+Na]^+$ calcd for $C_{31}H_{40}NaO_6Si$, 531.2717; found 531.2728.

Synthesis of compound 26.

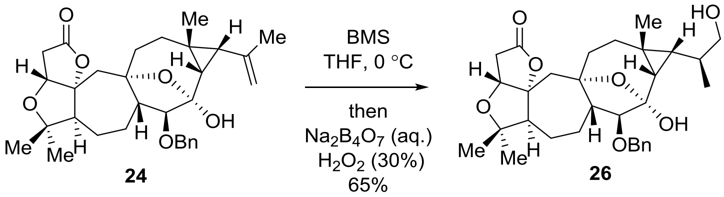

To a solution of **24** (12 mg, 0.0236 mmol) in 1 mL THF was added by 0.1 mL borane-dimethylsulfide complex (2M, THF solution) at 0 °C. After stirring for 40 min, 30% H_2O_2 (3 mL) and a saturated solution of $Na_2B_4O_7$ (3 mL) were added simultaneously. The mixture was warmed to r.t and stirred for 18 h. The mixture was separated and the aqueous layer was extracted with ethyl acetate (5 mL×3). The combined organic layer was washed with a saturated aqueous solution of Na_2CO_3 (5 mL) and brine (1 mL), dried over Na_2SO_4 and concentrated in vacuo. Purification by silica gel column chromatography (petroleum ether/ethyl acetate 4:1 to ethyl acetate) afforded **26** (8 mg, 0.0153 mmol, 65%) as white solid. R_f = 0.15 (silica gel, petroleum ether/ethyl acetate = 1:2); $[\alpha]_D^{20}$ = +14.7 (c = 0.87 in $CHCl_3$); IR (neat): ν_{max}= 3469, 2930, 2860, 1769, 1453, 1209, 1026 cm^{-1}; ^1H NMR (500 MHz, $CDCl_3$): δ = 7.36-7.27 (m, 5H), 4.67 (d, J = 11.9 Hz, 1H), 4.62 (d, J = 11.9 Hz, 1H), 4.08 (d, J = 5.8 Hz, 1H), 3.70 – 3.66 (m, 1H), 3.55 (dd, J = 10.6, 5.2 Hz, 1H), 3.41 (dd, J = 10.6, 7.5 Hz, 1H), 2.84 (s, 1H), 2.74 (dd, J = 18.6, 5.9 Hz, 1H), 2.59 (d, J = 18.5 Hz, 1H), 2.34-2.28 (m, 1H), 2.26 (d, J = 13.1 Hz, 1H), 2.20 (dd, J = 12.9, 4.6 Hz, 1H), 2.10 (ddd, J = 11.6, 5.5, 2.4 Hz, 1H), 1.94-1.88 (m, 1H), 1.89 (d, J = 15.8 Hz, 1H), 1.78 (d, J = 15.7 Hz, 1H), 1.73 (d, J = 13.1 Hz, 1H), 1.70-1.63 (m, 2H), 1.38-1.37 (m, 1H), 1.35-1.26 (m, 3H), 1.29 (s, 3H), 1.20 (d, J = 6.5 Hz, 3H), 1.17 (d, J = 9.2 Hz, 1H), 1.07 (s, 3H), 1.03 (s, 3H), 0.47 (dd, J = 10.8, 9.2 Hz, 1H) ppm; ^{13}C NMR (125 MHz, $CDCl_3$): δ = 175.4, 138.5, 128.5, 127.7,

127.4, 106.7, 96.7, 95.5, 84.3, 83.0, 81.2, 73.0, 69.4, 60.3, 53.4, 43.0, 38.2, 35.4, 32.7, 31.5, 30.4, 29.3, 28.9, 28.4, 24.8, 24.6, 23.2, 21.5, 18.3 ppm; HRMS (ESI, *m/z*): $[M+Na]^+$ calcd for $C_{31}H_{42}NaO_7$, 549.2823; found 549.2833. CCDC 1509138 contains the supplementary crystallographic data for compound **26** and is available free of charge from The Cambridge Crystallographic Data Centre via www.ccdc.cam.ac.uk/data_request/cif. The best single crystal we could get was recrystallized from ether-methanol-water mixed solvents, and it is very clear that there is statically uncertain crystalized solvent in the lattice. The solvent was removed manually during refinement in order to get better data.

Synthesis of compound 27.

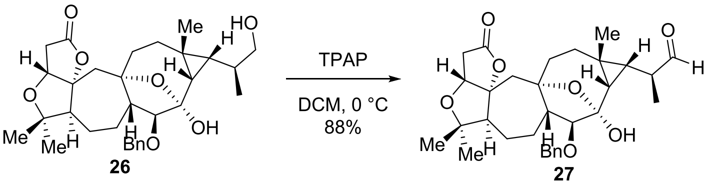

To a solution of **26** (18 mg, 0.0342 mmol) in 2 mL DCM was added by TPAP (16 mg, 0.0444 mmol) at 0 °C. After stirring 10 min, the mixture was filter via a silica pad, eluted with DCM to remove dark green fraction and with ethyl acetate (5 mL) to obtain crude product solution. The solution was concentrated in vacuo. Purification by silica gel column chromatography (petroleum ether/ethyl acetate 4:1) afforded **27** (16 mg, 0.03 mmol, 88%) as white solid. Data for **27**: R_f = 0.65 (silica gel, petroleum ether/ethyl acetate = 1:2); $[\alpha]_D^{20}$ = +38.3 (*c* = 0.47 in $CHCl_3$); IR (neat): ν_{max} = 3469, 2920, 2859, 1775, 1717, 1455, 1373, 1209, 1066 cm^{-1}; ^1H NMR (500 MHz, $CDCl_3$): δ = 9.68 (d, *J* = 1.6 Hz, 1H), 7.38-7.27 (m, 5H), 4.69 (d, *J* = 11.9 Hz, 1H), 4.63 (d, *J* = 11.9 Hz, 1H), 4.07 (d, *J* = 5.8 Hz, 1H), 3.69 (d, *J* = 3.0 Hz, 1H), 3.06 (dqd, *J* = 10.8, 7.0, 1.6 Hz, 1H), 2.78 (s, 1H), 2.73 (dd, *J* = 18.6, 6.0 Hz, 1H), 2.60 (d, *J* = 18.6 Hz, 1H), 2.22 (t, *J* = 12.3 Hz, 1H), 2.17-2.10 (m, 2H), 1.96-1.90 (m, 1H), 1.89 (d, *J* = 15.6 Hz, 1H), 1.77 (d, *J* = 15.6 Hz, 1H), 1.69-1.62 (m, 2H), 1.60-1.55 (m, 1H), 1.40 -1.37 (m, 2H), 1.35 (d, *J* = 7.0 Hz, 3H), 1.32-1.29 (m, 1H), 1.28 (s, 3H), 1.26 (d, *J* = 9.0 Hz, 1H), 1.10 (s, 3H), 1.06 (s, 3H), 0.67 (dd, *J* = 10.7, 8.9 Hz, 1H) ppm; ^{13}C NMR (125 MHz, $CDCl_3$): δ = 205.5, 175.0, 138.4, 128.5, 127.8, 127.5, 106.6, 96.3, 95.1, 84.2, 82.9, 81.3, 73.0, 60.4, 53.2, 43.0, 42.8, 38.6, 35.3, 29.8, 29.7, 29.0, 28.6, 28.4, 25.3, 24.6, 23.5, 21.4, 14.9 ppm; HRMS (ESI, *m/z*): $[M+NH_4]^+$ calcd for $C_{31}H_{44}O_7N$, 542.3112; found 542.3119.

Synthesis of compound 29.

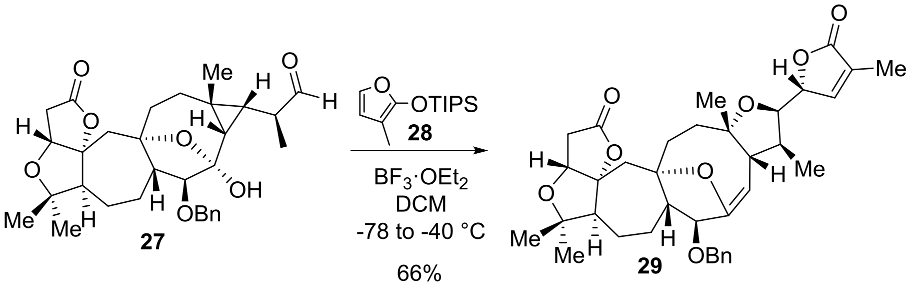

To a mixture of **27** (22.4 mg, 0.043 mmol) and **28** (21.6 mg, 0.085 mmol) in 2 mL DCM was added $BF_3{\bullet}OEt_2$ (6.6 mg dissolved in 0.1 mL DCM) at -78 °C and stirred for 1 h. Another portion of **28** (10.8 mg, 0.042 mmol) and $BF_3{\bullet}OEt_2$ (6.6 mg dissolved in 0.1 mL DCM) was added and after

stirred for one more hour. Then BF_3•OEt_2 (6.6 mg dissolved in 0.1 mL DCM) was added and the mixture was allowed to warm to -35 °C. After stirring for 1 h, the reaction was quenched by saturated solution of $NaHCO_3$ (1 mL). The mixture was separated and the aqueous layer was extracted with EtOAc (5 mL×3). The combined organic layer was washed with brine (1 mL), dried over Na_2SO_4 and concentrated under reduced pressure. Purification by silica gel column chromatography (petroleum ether/EtOAc 4:1 to EtOAc) afforded **29** (14.8 mg, 0.0245 mmol, 66%) as white solid. Data for **29**: R_f = 0.15 (silica gel, petroleum ether/ethyl acetate = 1:2); $[\alpha]_D^{20}$ = -44.2 (c=0.6 in $CHCl_3$); IR (neat): 2967, 2930, 2870, 1754, 1453, 1374, 1211, 1101, 1067, 914, 731 cm^{-1}; ^{1}H-NMR (500 MHz, $CDCl_3$): δ = 7.38-7.28 (m, 5H), 6.95 (s, 1H), 4.99 (dd, *J* = 9.0, 1.7 Hz, 1H), 4.88 (s, 1H), 4.65 (d, *J* = 12.1 Hz, 1H), 4.59 (d, *J* = 12.1 Hz, 1H), 4.24 (d, *J* = 8.7 Hz, 1H), 4.10 (d, *J* = 6.5 Hz, 1H), 3.87 (s, 1H), 3.41-3.32 (m, 1H), 2.79 (dd, *J* = 18.7, 6.6 Hz, 1H), 2.64 (d, *J* = 18.7 Hz, 1H), 2.35 (dd, *J* = 13.0, 9.1 Hz, 1H), 2.22 (d, *J* = 15.8 Hz, 1H), 2.18 – 2.13 (m, 1H), 2.04-1.98 (m, 2H), 1.97-1.91 (m, 1H), 1.93 (s, 3H), 1.70 (ddd, *J* = 14.3, 9.4, 4.9 Hz, 1H), 1.59-1.50 (m, 1H), 1.54 (s, 3H), 1.35 (dd, *J* = 13.7, 10.2 Hz, 1H), 1.29 (s, 3H), 1.26-1.21 (m, 1H), 1.19 (d, *J* = 6.7 Hz, 3H), 1.13 (s, 3H), 1.06 (s, 3H) ppm; ^{13}C-NMR (125 MHz, $CDCl_3$): 175.4, 174.9, 157.7, 147.0, 138.3, 130.9, 128.6, 127.9, 127.5, 101.7, 95.8, 86.4, 84.2, 84.1, 83.8, 81.2, 81.0, 77.6, 72.8, 61.7, 51.9, 51.3, 40.3, 40.1, 36.9, 35.5, 34.5, 30.8, 28.2, 26.3, 23.6, 21.2, 13.1, 11.0 ppm; HRMS (ESI, m/z): $[M+Na]^+$ calcd for $C_{36}H_{44}NaO_8$, 627.2928; found, 627.2935. CCDC 1507976 contains the supplementary crystallographic data for compound **29** and is available free of charge from The Cambridge Crystallographic Data Centre via www.ccdc.cam.ac.uk/data_request/cif.

Synthesis of compound 30.

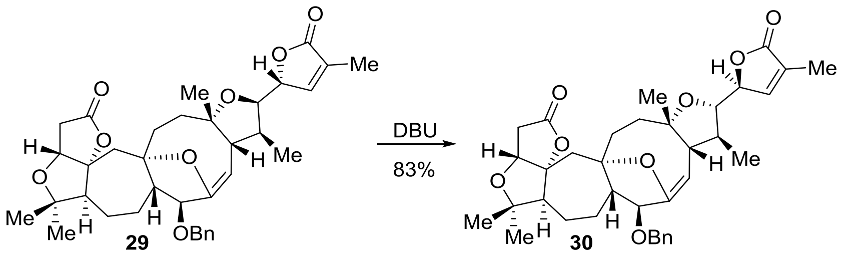

To a solution of **29** (42 mg, 0.069 mmol) in 4 mL toluene was added DBU (158 mg, 1.042 mmol) at r.t. and warmed to 65 °C stirred for 18 h. After that, saturated solution of NH_4Cl (1 mL) was added. The mixture was separated and the aqueous layer was extracted with EtOAc (5 mL×3). The combined organic layer was washed with brine (1 mL), dried over Na_2SO_4 and concentrated under reduced pressure. Purification by silica gel column chromatography (petroleum ether/EtOAc 6:1 to EtOAc) afforded **30** (35 mg, 0.058 mmol, 83%) as white solid. Data for **30**: R_f = 0.45 (silica gel, petroleum ether/ethyl acetate = 1:2); $[\alpha]_D^{20}$ = +2.5 (c=0.4 in $CHCl_3$); IR (neat): 2969, 2924, 2870, 1774, 1746, 1456, 1377, 1209, 1104, 1084, 1072, 920, 735 cm^{-1}; ^{1}H-NMR (500 MHz, $CDCl_3$): δ = 7.37-7.28 (m, 5H), 7.02 (s, 1H), 4.95 (d, *J* = 8.8 Hz, 1H), 4.89 (s, 1H), 4.62 (s, 2H), 4.22 (d, *J* = 6.3 Hz, 1H), 3.89 (s, 1H), 3.68 (d, *J* = 9.3 Hz, 1H), 3.51-3.44 (m, 1H), 3.46 (dd, *J* = 18.5, 6.3 Hz, 1H), 2.64 (d, *J* = 15.9 Hz, 1H), 2.57 (d, *J* = 18.5 Hz, 1H), 2.24 (dd, *J* = 13.7, 9.0 Hz, 1H), 2.16 (dd, *J* = 12.9, 4.5 Hz, 1H), 2.04-1.93 (m, 3H), 1.91 (s, 3H), 1.87 (dd, *J* = 12.8, 5.9 Hz, 1H), 1.76 (d, *J* = 15.9 Hz, 1H), 1.65 (ddd, *J* = 14.0, 9.2, 4.6 Hz, 1H), 1.61-1.56 (m, 1H), 1.29 (s, 3H), 1.27-1.19 (m, 2H), 1.17 (s, 3H), 1.15-1.12 (m, 1H), 1.09 (d, *J* = 6.5 Hz, 3H), 1.05 (s, 3H) ppm; ^{13}C-NMR (125 MHz, $CDCl_3$): δ = 176.2, 175.2, 158.5, 147.4, 138.4, 130.3, 128.6, 127.8, 127.4, 100.9, 95.7, 86.4,

84.7, 84.4, 83.6, 81.8, 80.3, 72.5, 61.9, 55.6, 50.9, 39.8, 38.5, 35.6, 35.0, 34.4, 31.4, 28.2, 27.4, 24.0, 21.1, 16.2, 10.9 ppm; HRMS (ESI, m/z): $[M+H]^+$ calcd for $C_{36}H_{45}O_8$, 605.3109; found, 605.3103.

Synthesis of compound 31.

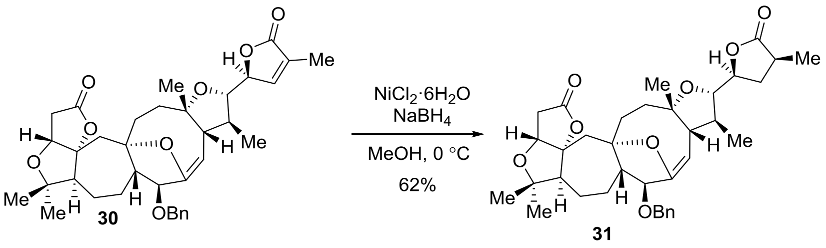

To a solution of **30** (13.2 mg, 0.0218 mmol) and $NiCl_2 \cdot 6H_2O$ (5.2 mg, 0.0218 mmol) in 1 mL MeOH was added $NaBH_4$ (2.5 mg, 0.0655mmol) at 0 °C. After stirred for 0.5 h, saturated solution of NH_4Cl (1 mL) was added to quench the reaction. The mixture was separated and the aqueous layer was extracted with EtOAc (5 mL×3). The combined organic layer was washed with brine (1 mL), dried over Na_2SO_4 and concentrated under reduced pressure. Purification by silica gel column chromatography (petroleum ether/EtOAc 6:1 to EtOAc) afforded **31** (8.2 mg, 0.0135 mmol, 62%) as white solid. Data for **31**: R_f = 0.4 (silica gel, petroleum ether/ethyl acetate = 1:1); $[\alpha]_D^{20}$ = -15.5 (c=0.6 in $CHCl_3$); IR (neat): 2967, 2932, 2868, 1762, 1454, 1373, 1203, 1167,1103, 1018, 913, 728 cm^{-1}; ^{1}H-NMR (500 MHz, $CDCl_3$): δ = 7.37-7.28 (m, 5H), 4.96 (d, J = 8.6 Hz, 1H), 4.62 (s, 2H), 4.42 (t, J = 8.0 Hz, 1H), 4.18 (d, J = 6.3 Hz, 1H), 3.87 (s, 1H), 3.41 (d, J = 9.5 Hz, 1H), 3.37 (dd, J = 18.5, 6.0 Hz, 1H), 3.37-2.29 (m, 1H), 2.71-2.63 (m, 1H), 2.60 (d, J = 16.0 Hz, 1H), 2.53 (d, J = 18.5 Hz, 1H), 2.38-2.29 (m, 2H), 2.14 (dd, J = 12.8, 4.5 Hz, 1H), 2.07-1.95 (m, 4H), 1.87 (dd, J = 12.7, 5.8 Hz, 1H), 1.76 (d, J = 15.8 Hz, 1H), 1.65 (ddd, J = 14.0, 9.1, 4.6 Hz, 1H), 1.60-1.53 (m, 2H), 1.28 (d, J = 8.5, 3H), 1.28 (s, 3H), 1.22 (s, 3H), 1.20-1.18 (m, 2H), 1.05 (s, 3H), 1.03 (d, J = 6.7 Hz, 3H) ppm; ^{13}C-NMR (125 MHz, $CDCl_3$): δ = 180.2, 176.2, 158.3, 138.5, 128.6, 127.8, 127.4, 101.2, 95.7, 86.1, 84.7, 84.6, 84.4, 83.6, 81.7, 76.8, 72.5, 61.9, 56.1, 51.0, 39.4, 38.7, 35.9, 35.6, 34.9, 34.7, 32.1, 31.3, 28.2, 27.5, 23.9, 21.1, 16.1, 15.6 ppm; HRMS (ESI, m/z): $[M+H]^+$ calcd for $C_{36}H_{47}O_8$, 607.3265; found, 607.3260. CCDC 1507977 contains the supplementary crystallographic data for compound **31** and is available free of charge from The Cambridge Crystallographic Data Centre via www.ccdc.cam.ac.uk/data_request/cif. According to http://checkcif.iucr.org/, One B Alert has been raised concerning about the short contact between O163 and O1. The O1 could be assigned to water considering about the surroundings and the number of atoms, but the hydrogens were not added due to insufficient occupancy. Thus the short contact between O163 and O1 actually suggests an intermolecular hydrogen bond.

Synthesis of compound S17.

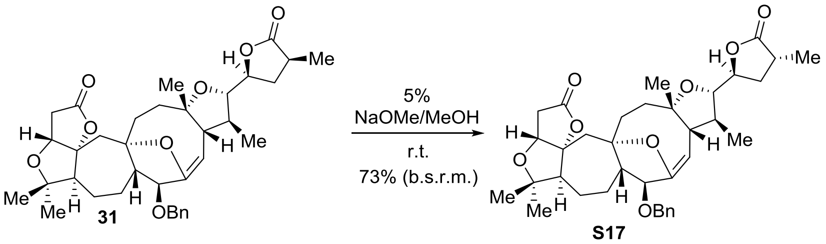

A solution of 5% NaOMe/MeOH (1 mL) was added into **31** (12.8 mg, 0.0138 mmol) at 0 °C and the mixture was allowed to warm to r.t. After stirred for 2 h, the mixture was poured into ice $NaHCO_3$ saturated solution, and the aqueous layer was extracted with EtOAc (5 mL×3). The combined organic layer was washed with brine, dried over Na_2SO_4 and concentrated under reduced pressure. Purification by silica gel column chromatography (petroleum ether/EtOAc 6:1 to EtOAc) afforded **S17** (2.9 mg, 0.0048 mmol, 73% b.r.s.m.) and recovered **31** (8.8 mg, 0.0145 mmol). **S17** was a white solid. R_f = 0.5 (silica gel, petroleum ether/ethyl acetate = 1:2); $[\alpha]_D^{20}$ = -15.9 (c=0.17 in $CHCl_3$); IR (neat): 2967, 2927, 2869, 1773, 1455, 1373, 1203, 1177, 1111, 1065, 914, 733 cm^{-1}; ^{1}H-NMR (500 MHz, $CDCl_3$): δ = 7.37-7.28 (m, 5H), 4.95 (d, *J* = 8.8 Hz, 1H), 4.61 (s, 2H), 4.48 (d, *J* = 9.2 Hz, 1H), 4.17 (d, *J* = 6.2 Hz, 1H), 3.85 (s, 1H), 3.47 (d, *J* = 10.0 Hz, 1H), 3.43 (dd, *J* = 18.5, 6.3 Hz, 1H), 3.32-3.24 (m, 1H), 2.98-2.89 (m, 1H), 2.56 (d, *J* = 18.5 Hz, 1H), 2.54 (d, *J* = 15.8 Hz, 1H), 2.47 – 2.41 (m, 1H), 2.17-2.10 (m, 2H), 2.04-1.94 (m, 4H), 1.85 (dd, *J* = 12.7, 5.7 Hz, 1H), 1.72 (d, *J* = 15.9 Hz, 1H), 1.64 (ddd, *J* = 14.0, 9.1, 4.6 Hz, 1H), 1.60-1.52 (m, 2H), 1.28 (s, 3H), 1.22 (d, *J* = 7.2 Hz, 3H), 1.20 (s, 3H), 1.18-1.12 (m, 2H), 1.04 (s, 3H), 1.02 (d, *J* = 6.4 Hz, 3H) ppm; ^{13}C-NMR (125 MHz, $CDCl_3$): δ = 181.9, 176.1, 158.3, 138.4, 128.6, 127.8, 127.4, 101.2, 95.6, 87.6, 86.1, 84.6, 84.4, 83.6, 81.8, 75.9, 72.4, 61.8, 55.5, 50.7, 38.8, 38.4, 35.5, 35.0, 34.6, 34.2, 33.5, 31.4, 28.2, 27.5, 24.0, 21.1, 16.0, 15.8 ppm; HRMS (ESI, m/z): $[M+H]^+$ calcd for $C_{36}H_{47}O_8$, 607.3265; found, 607.3273.

Synthesis of compound 32.

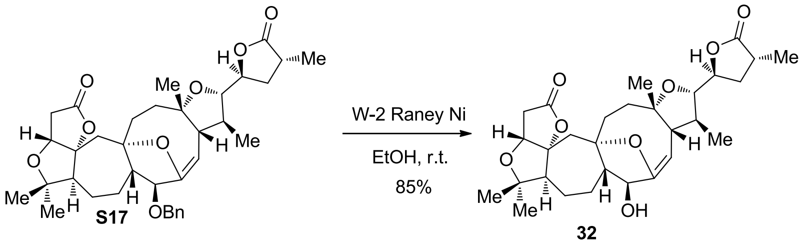

To a solution of **S17** (2.5 mg, 0.0041 mmol) in 1 mL EtOH was added W-2 Raney Ni, and the reaction was monitored by TLC. After stirring for 12 h, the suspension was filtered via a Celite pad and washed with EtOAc for several times. The obtained solution was concentrated under reduced pressure. Purification by silica gel column chromatography (petroleum ether/EtOAc 5:1 to 4:1) afforded **32** (1.8 mg, 0.0035 mmol, 85%) as white solid. Data for **32**: R_f = 0.15 (silica gel, petroleum ether/ethyl acetate = 1:1); $[\alpha]_D^{20}$ = +4.4 (c=0.18 in $CHCl_3$); IR (neat): 3462, 2969, 2930, 2871, 1759, 1457, 1374, 1204, 1179, 1102, 1065, 921, 730 cm^{-1}; ^{1}H-NMR (500 MHz, $CDCl_3$): δ = 4.87 (d, *J* = 8.5 Hz, 1H), 4.48 (d, *J* = 9.1 Hz, 1H), 4.18 (d, *J* = 6.3 Hz, 1H), 4.12 (d, *J* = 6.2 Hz, 1H), 3.47 (d, *J* = 9.6 Hz, 1H), 3.41 (dd, *J* = 18.4, 6.4 Hz, 1H), 3.32-3.24 (m, 1H), 2.97-2.89 (m, 1H), 2.57 (d, *J* = 18.5 Hz, 1H), 2.55 (d, *J* = 15.9 Hz, 1H), 2.48–2.39 (m, 1H), 2.23-2.12 (m, 3H), 2.04 – 1.95 (m, 2H), 1.84-1.67 (m, 4H), 1.74 (d, *J* = 15.7 Hz, 1H), 1.61 (dd, *J* = 13.8, 9.8 Hz, 1H),

1.56-1.51 (m, 1H), 1.29 (s, 3H), 1.27-1.20 (m, 2H), 1.22 (d, J = 7.2 Hz, 3H), 1.19 (s, 3H), 1.05 (s, 3H), 1.02 (d, J = 6.4 Hz, 3H) ppm; ^{13}C-NMR (125 MHz, $CDCl_3$): δ = 181.8, 176.1, 161.0, 100.0, 95.6, 87.5, 86.0, 84.5, 83.6, 81.8, 78.0, 75.9, 62.0, 55.5, 53.9, 38.8, 38.5, 36.2, 35.0, 34.7, 34.2, 33.5, 30.7, 28.2, 27.5, 23.8, 21.1, 16.1, 15.8 ppm; HRMS (ESI, m/z): $[M+H]^+$ calcd for $C_{29}H_{41}O_8$, 517.2796; found, 517.2798. CCDC 1507978 contains the supplementary crystallographic data for compound **32** and is available free of charge from The Cambridge Crystallographic Data Centre via www.ccdc.cam.ac.uk/data_request/cif.

Synthesis of compound 1a.

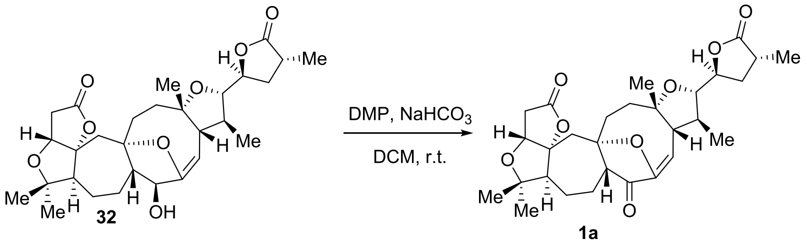

To a solution of **32** (3.2 mg, 0.0062 mmol) in 1 mL DCM was added $NaHCO_3$ (2.6 mg, 0.031 mmol) and DMP (5.2 mg, 0.0124 mmol) at r.t. After stirred for 2 h, saturated solution of Na_2SO_3 was added and the mixture was separated. The aqueous layer was extracted with EtOAc for 3 times and the combined organic layer was washed with brine, dried over Na_2SO_4 and concentrated under reduced pressure. Purification by silica gel column chromatography (petroleum ether/EtOAc 5:1 to 4:1) afforded **1a** (2.8 mg, 0.0054 mmol, 87%) as white solid. Data for **1a**: R_f = 0.5 (silica gel, petroleum ether/ethyl acetate = 1:2); $[\alpha]_D^{20}$ = -89.5 (c=0.4 in $CHCl_3$); IR (neat): 2969, 2931, 2871, 1768, 1740, 1456, 1374, 1195, 1064, 920, 729 cm^{-1}; ^{1}H-NMR (500 MHz, $CDCl_3$): δ = 5.72 (d, J = 8.7 Hz, 1H), 4.49 (d, J = 9.1 Hz, 1H), 4.22 (d, J = 6.0 Hz, 1H), 3.52 (dd, J = 9.7, 1.2 Hz, 1H), 3.46 (dd, J = 18.3, 6.1 Hz, 1H), 3.42-3.34 (m, 1H), 2.94-2.88 (m, 1H), 2.60 (d, J = 18.3 Hz, 1H), 2.44 (ddd, J = 12.7, 9.4, 1.9 Hz, 1H), 2.32-2.24 (m, 2H), 2.20 (dd, J = 12.3, 8.8 Hz, 1H), 2.12 (d, J = 17.6 Hz, 1H), 2.12-1.98 (m, 3H), 1.84 (d, J = 15.8 Hz, 1H), 1.82-1.71 (m, 2H), 1.57 (dd, J = 15.0, 8.9 Hz, 1H), 1.38-1.33 (m, 2H), 1.31 (s, 3H), 1.27-1.25 (m, 1H), 1.23 (d, J = 7.3 Hz, 3H), 1.17 (s, 3H), 1.08 (s, 3H), 1.06 (d, J = 6.4 Hz, 3H) ppm; ^{13}C-NMR (125 MHz, $CDCl_3$): δ = 199.9, 181.8, 175.8, 154.0, 111.4, 95.4, 87.7, 85.3, 84.6, 83.9, 81.8, 75.5, 61.6, 55.9, 54.4, 39.9, 38.3, 35.3, 35.0, 34.5, 34.2, 33.5, 28.1, 27.7, 25.5, 24.3, 21.2, 16.1, 15.8 ppm; HRMS (ESI, m/z): $[M+Na]^+$ calcd for $C_{29}H_{39}O_8$, 515.2640; found, 515.2639.

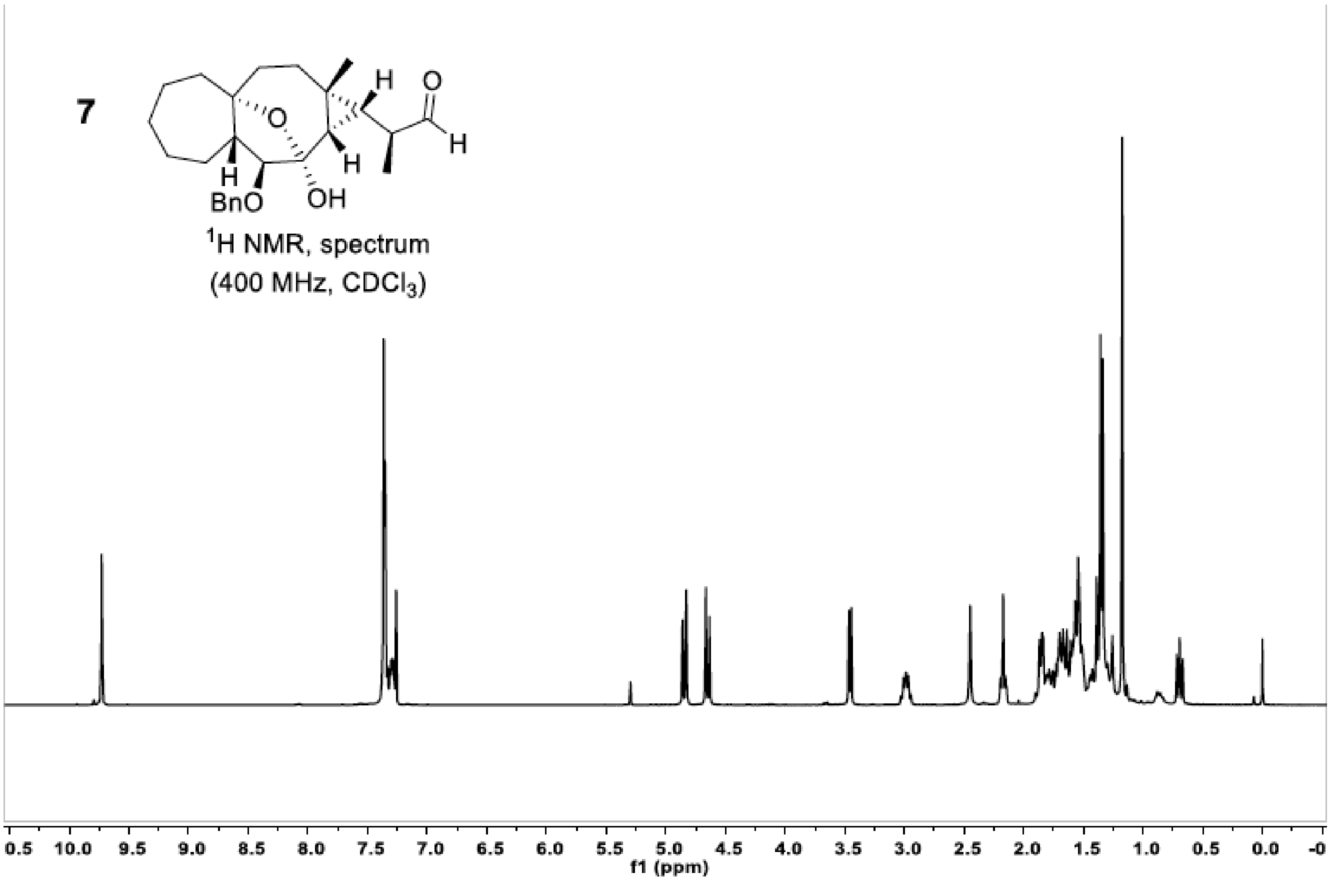

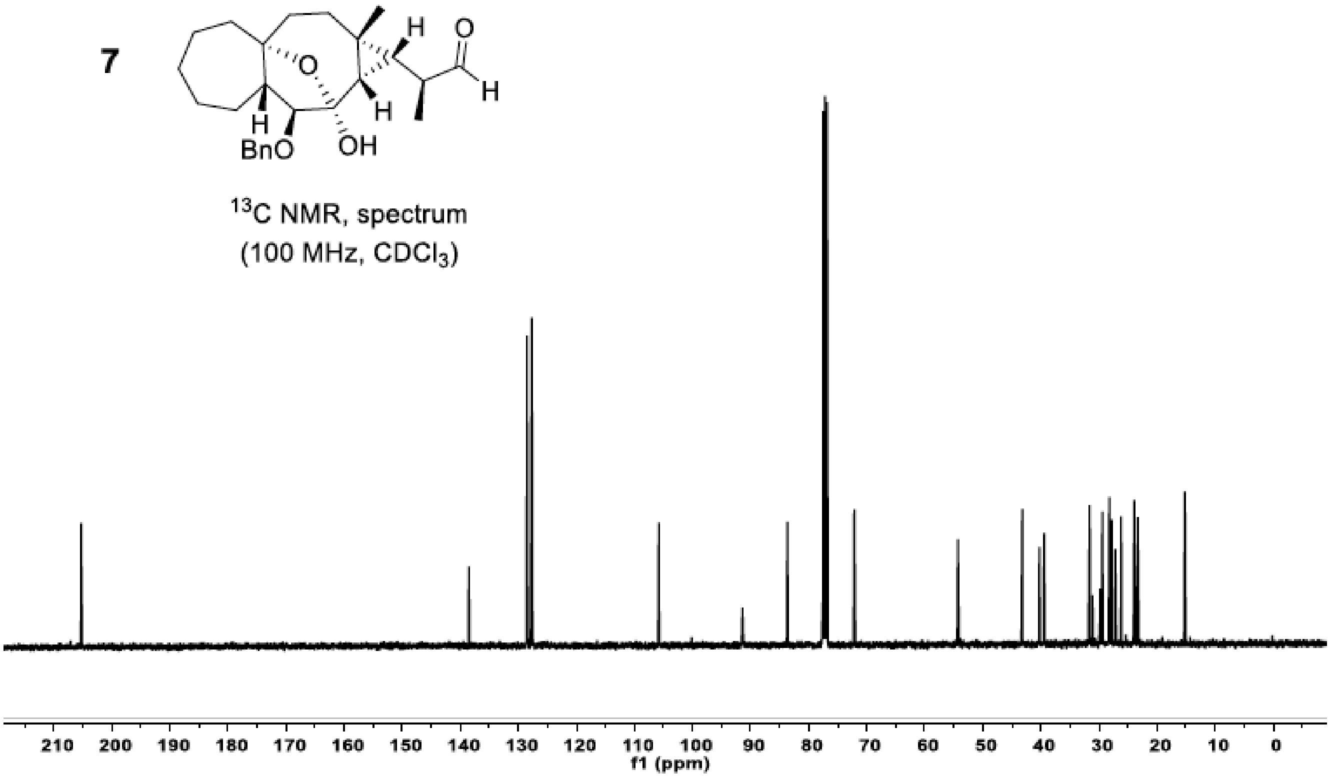

Supplementary Figure 1 | ^{1}H and ^{13}C NMR Spectra for Compound 7

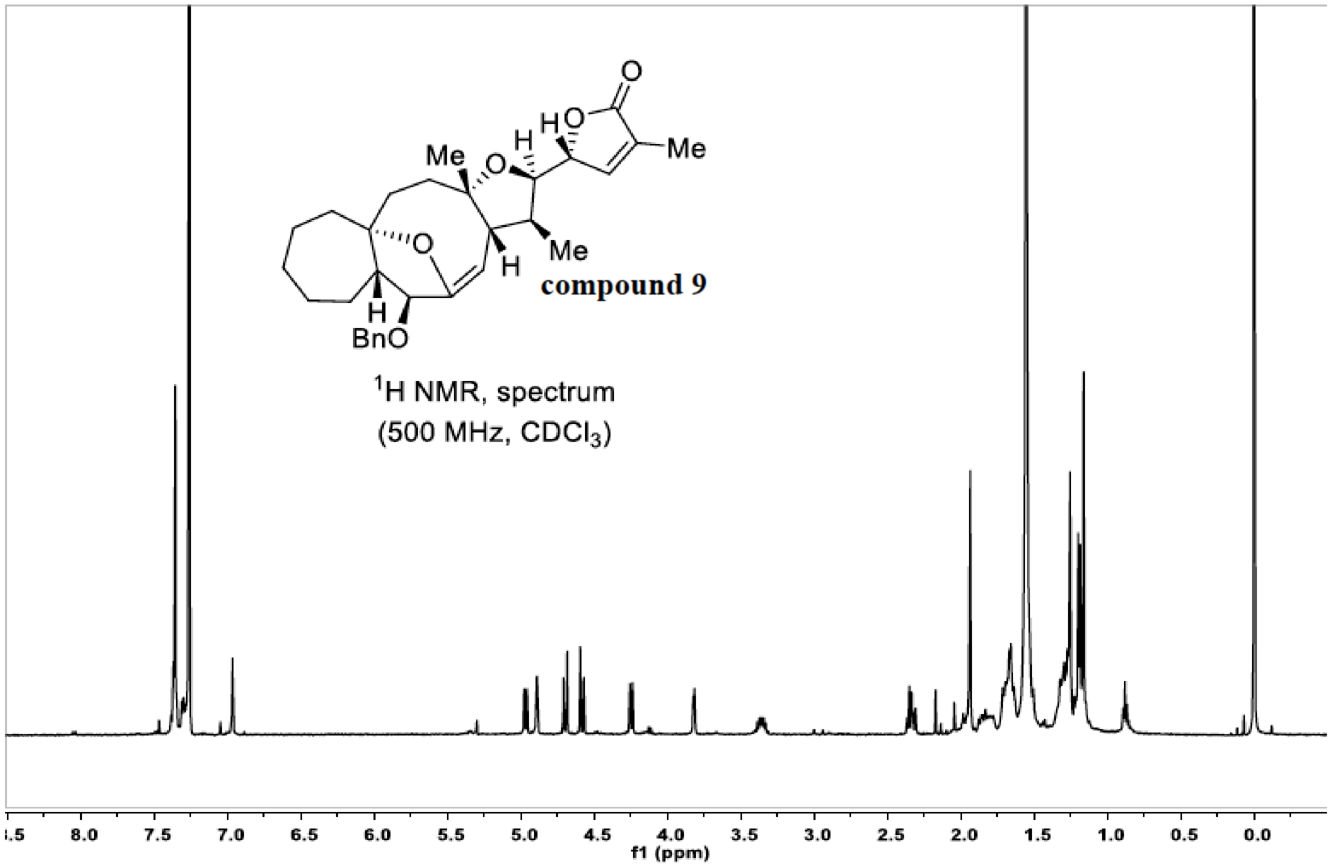
compound 9
^{1}H NMR, spectrum
(500 MHz, $CDCl_3$)
f1 (ppm)

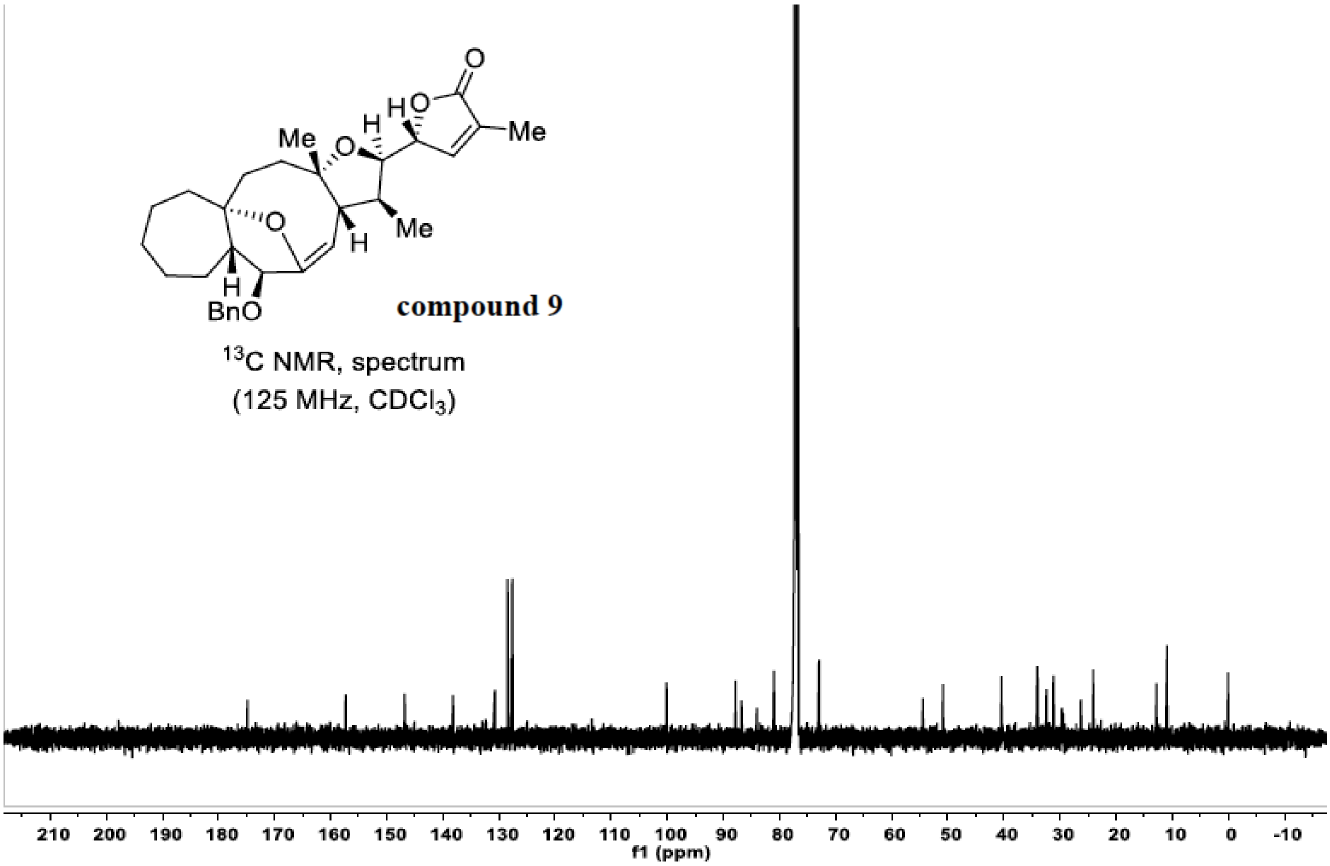
compound 9
^{13}C NMR, spectrum
(125 MHz, $CDCl_3$)
f1 (ppm)

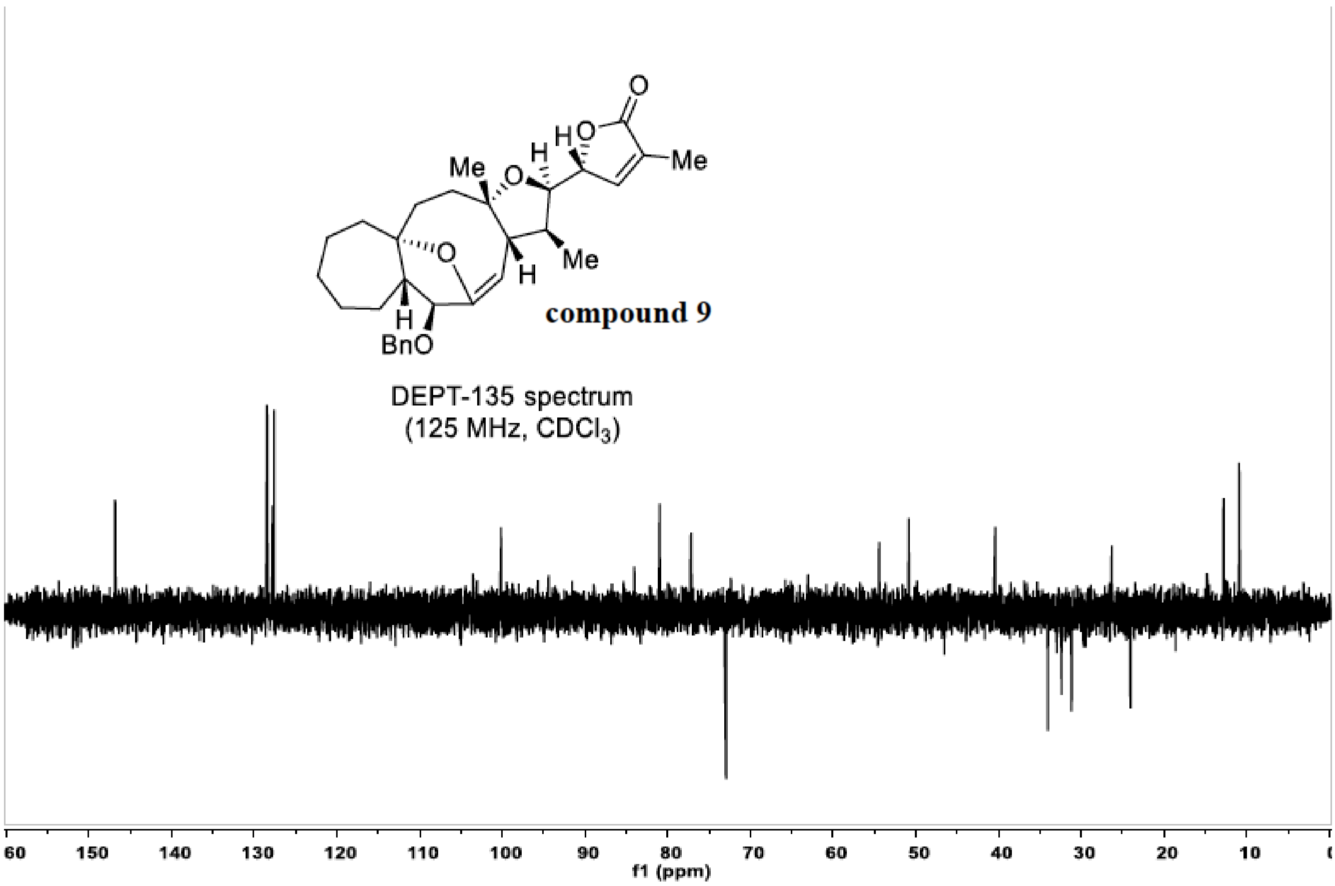
compound 9
DEPT-135 spectrum
(125 MHz, CDCl3)
f1 (ppm)

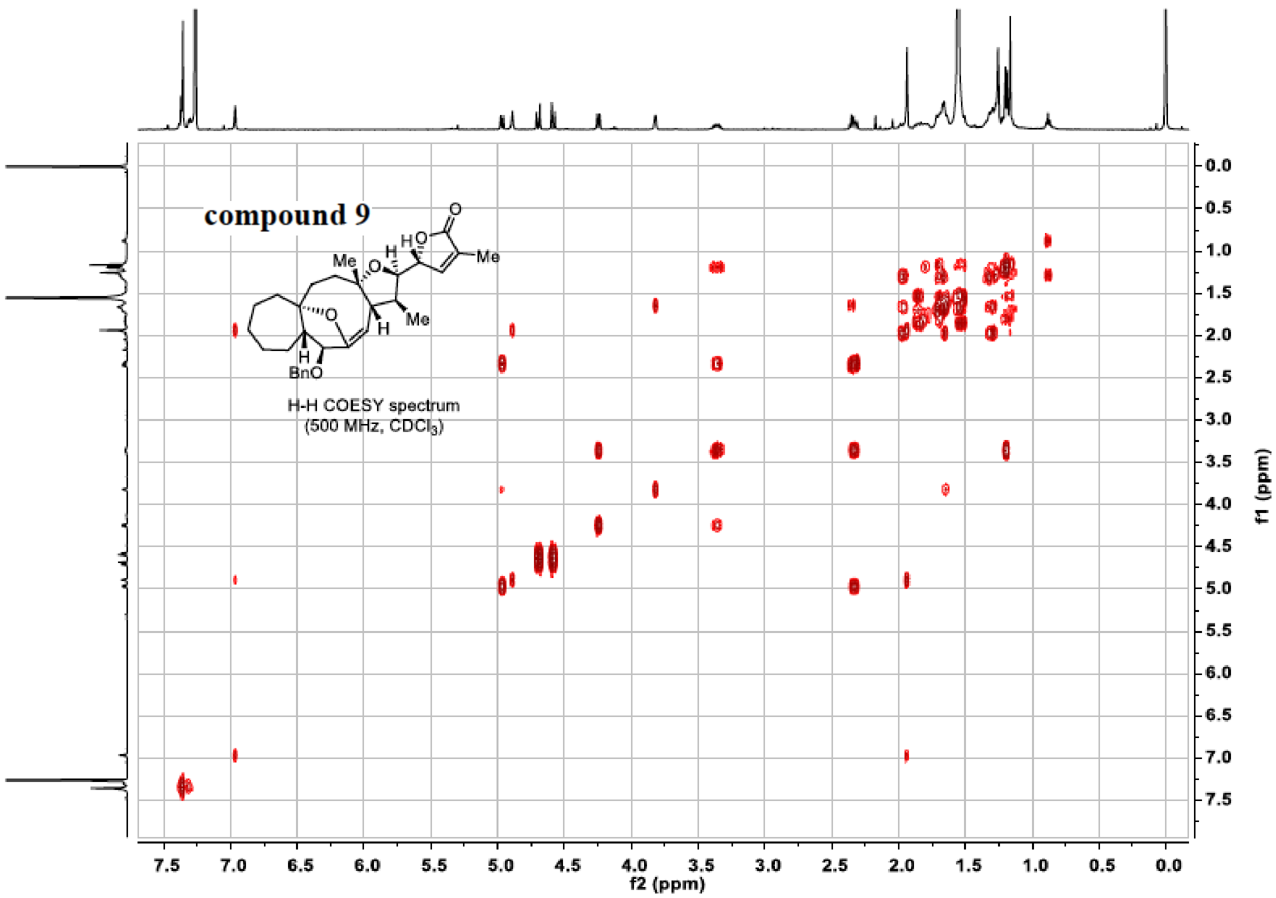
compound 9
H-H COESY spectrum
(500 MHz, CDCl3)
f2 (ppm)
f1 (ppm)

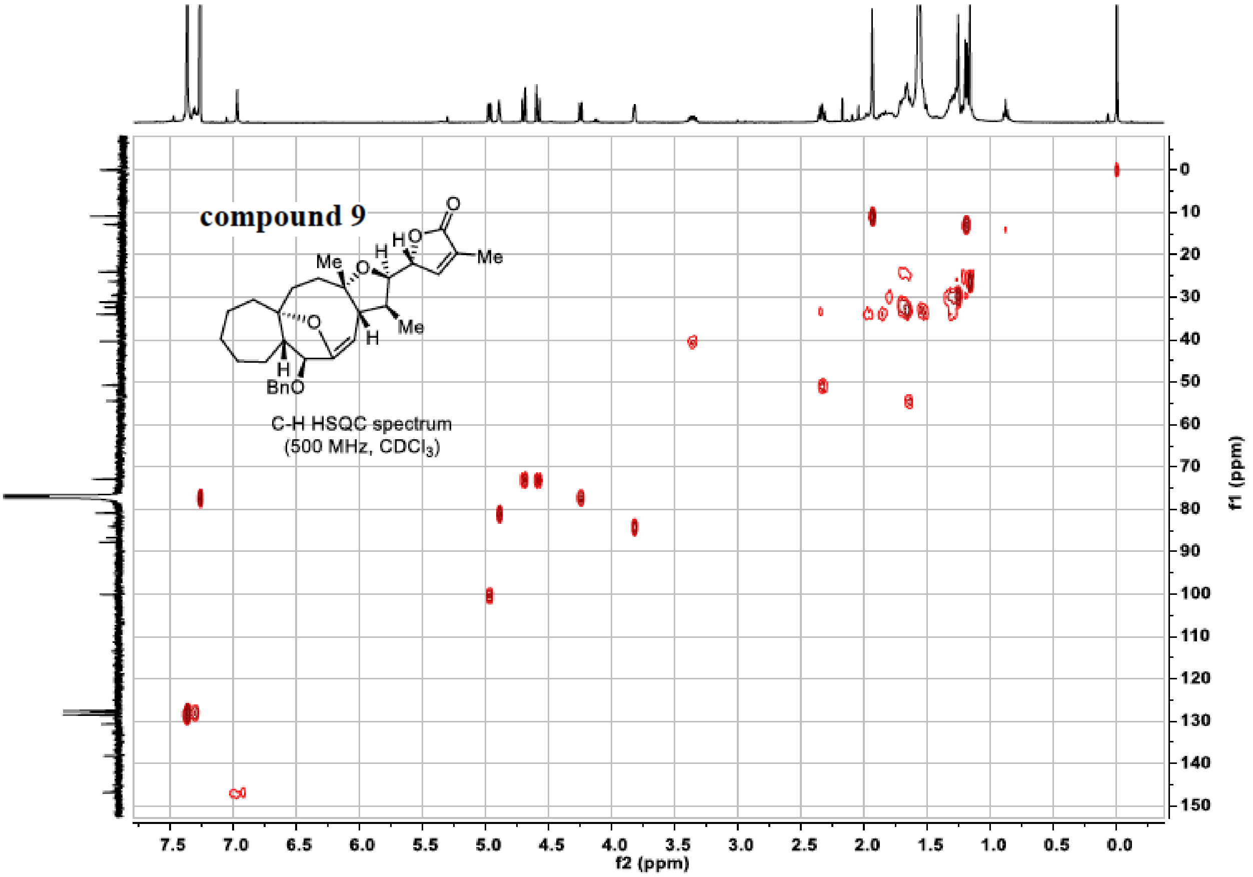
compound 9
C-H HSQC spectrum
(500 MHz, CDCl3)
f2 (ppm)
f1 (ppm)

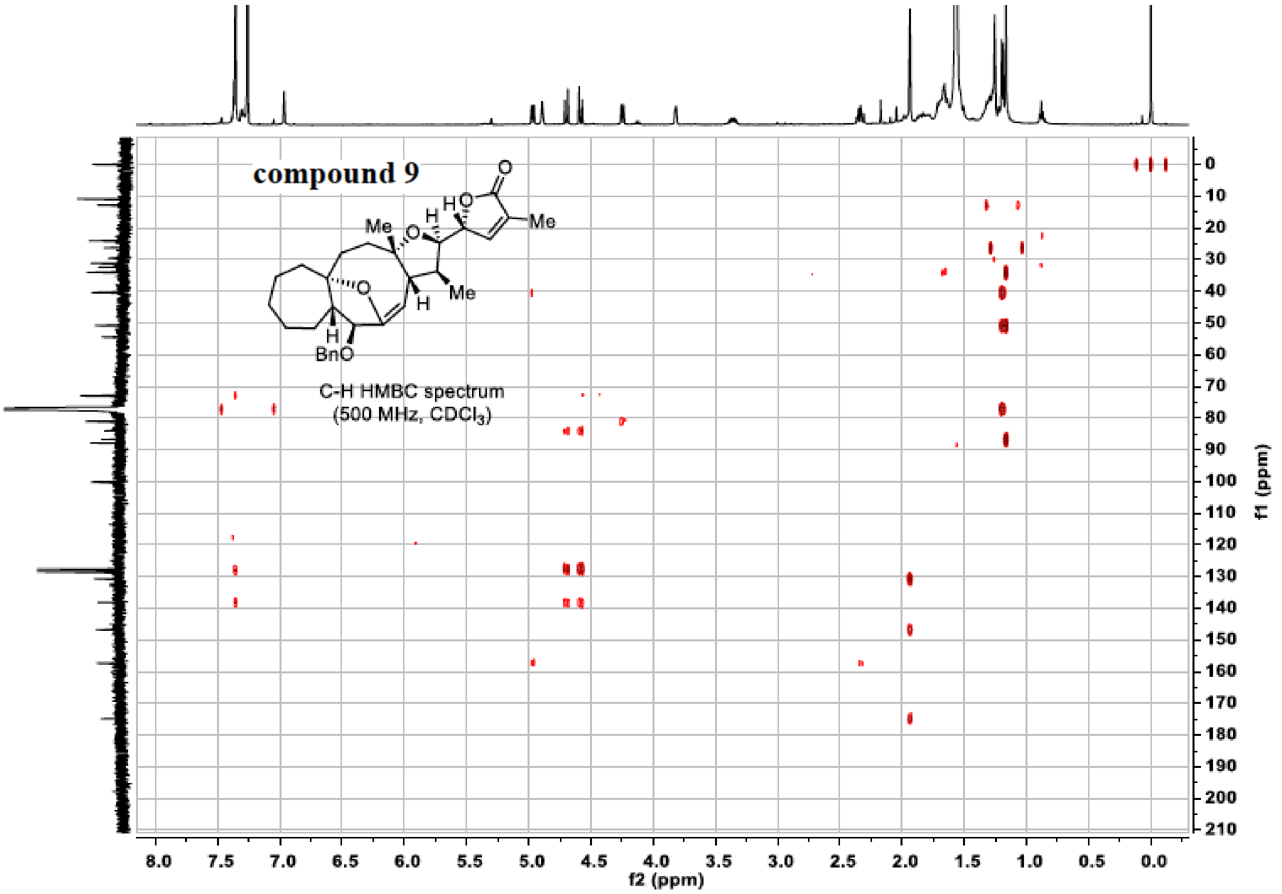
compound 9
C-H HMBC spectrum
(500 MHz, CDCl3)
f2 (ppm)
f1 (ppm)

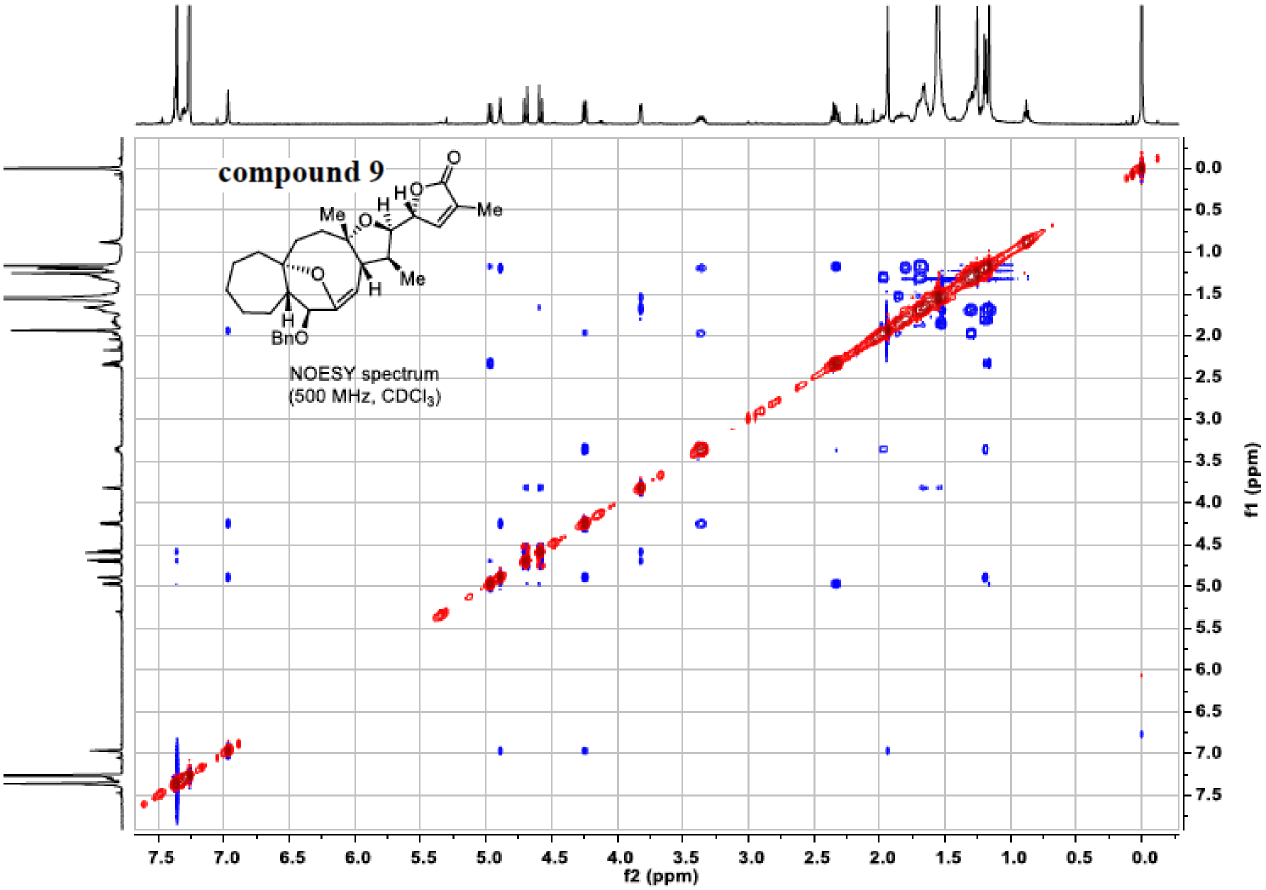

Supplementary Figure 2 | ^{1}H, ^{13}C, DEPT135, COESY, HSQC, HMBC, NOESY Spectra for Compound 9

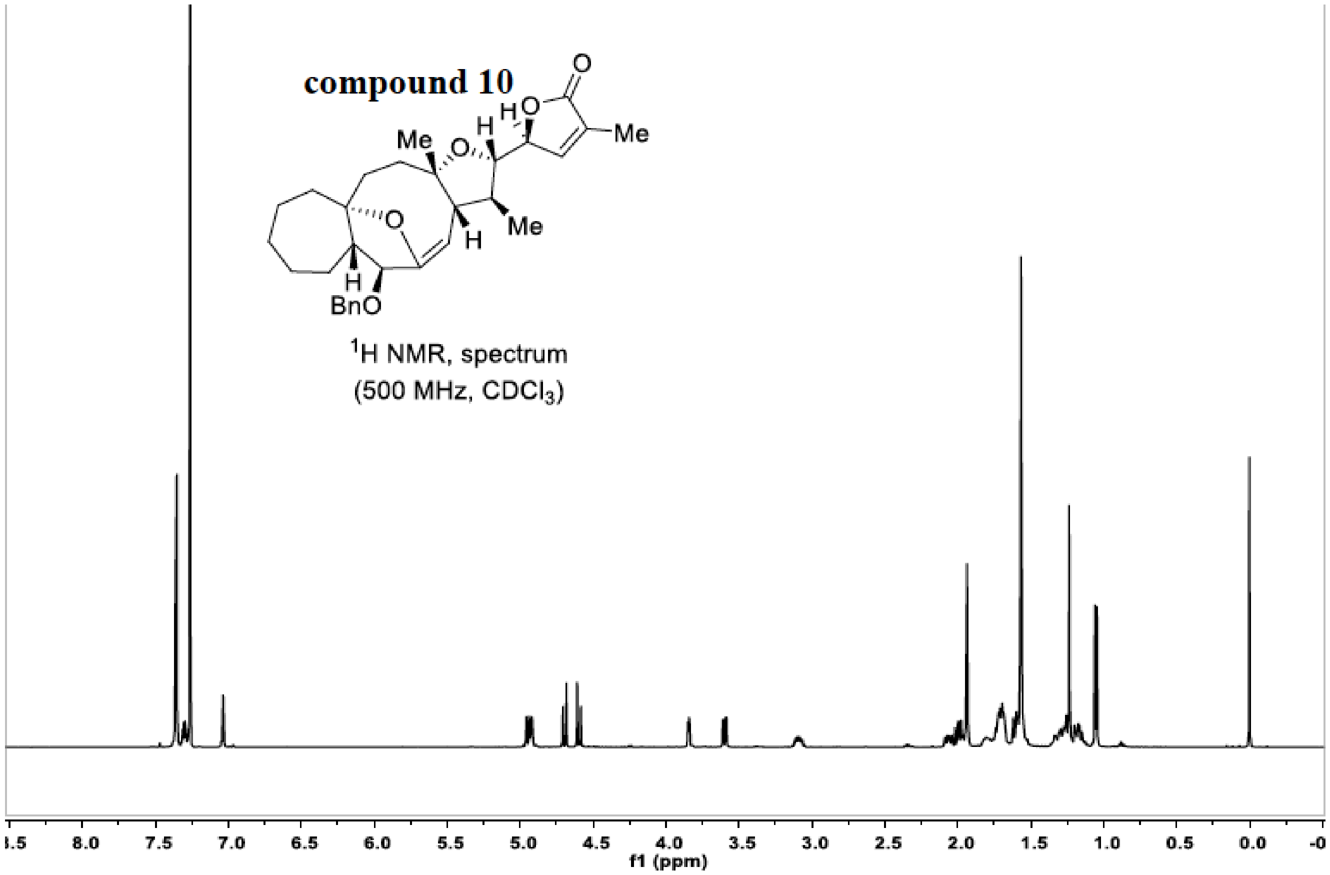
compound 10
Me
H
H
O
Me
Me
H
H
BnO
¹H NMR, spectrum
(500 MHz, CDCl3)
f1 (ppm)

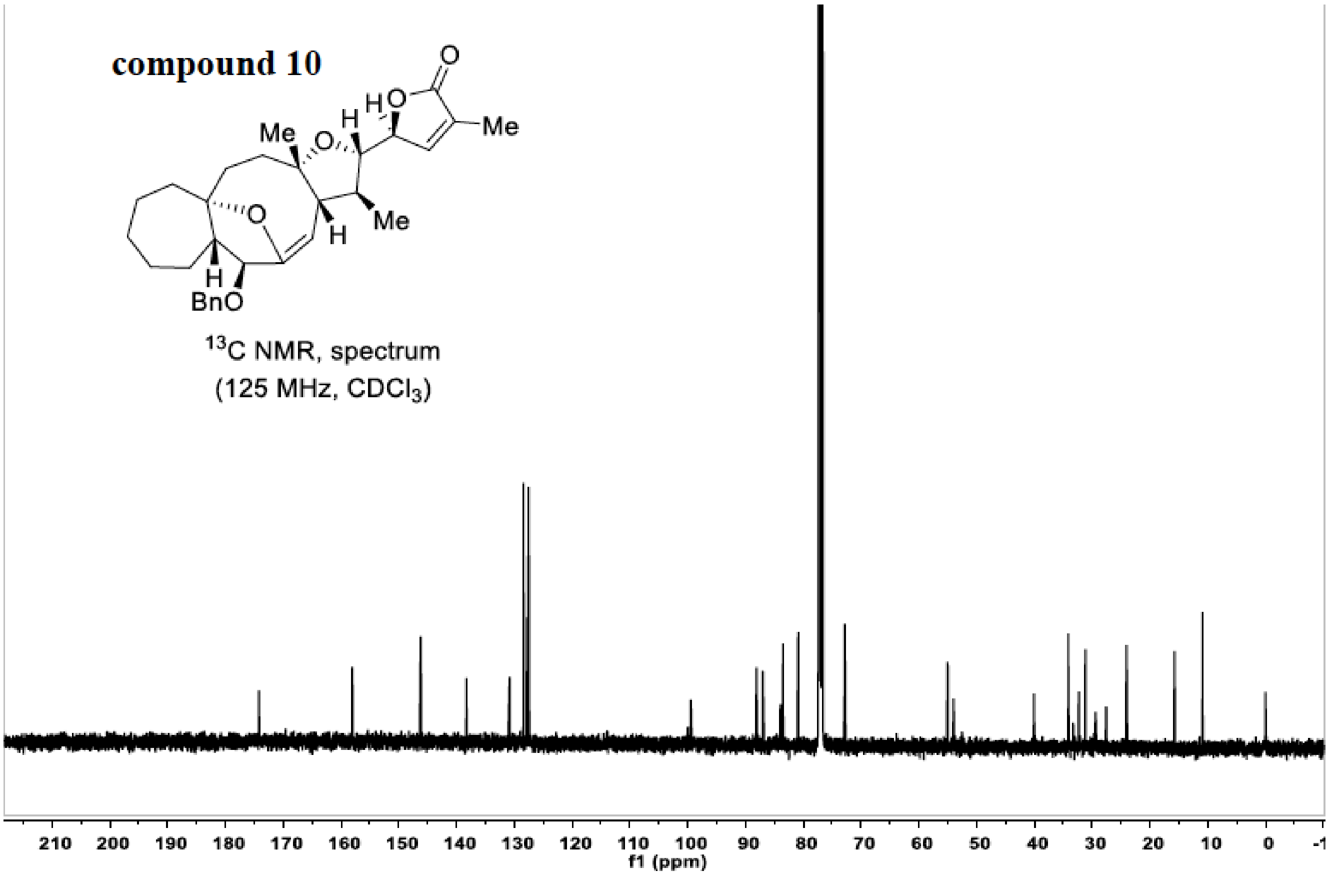
compound 10
Me
H
H
O
Me
Me
H
H
BnO
¹³C NMR, spectrum
(125 MHz, CDCl3)
f1 (ppm)

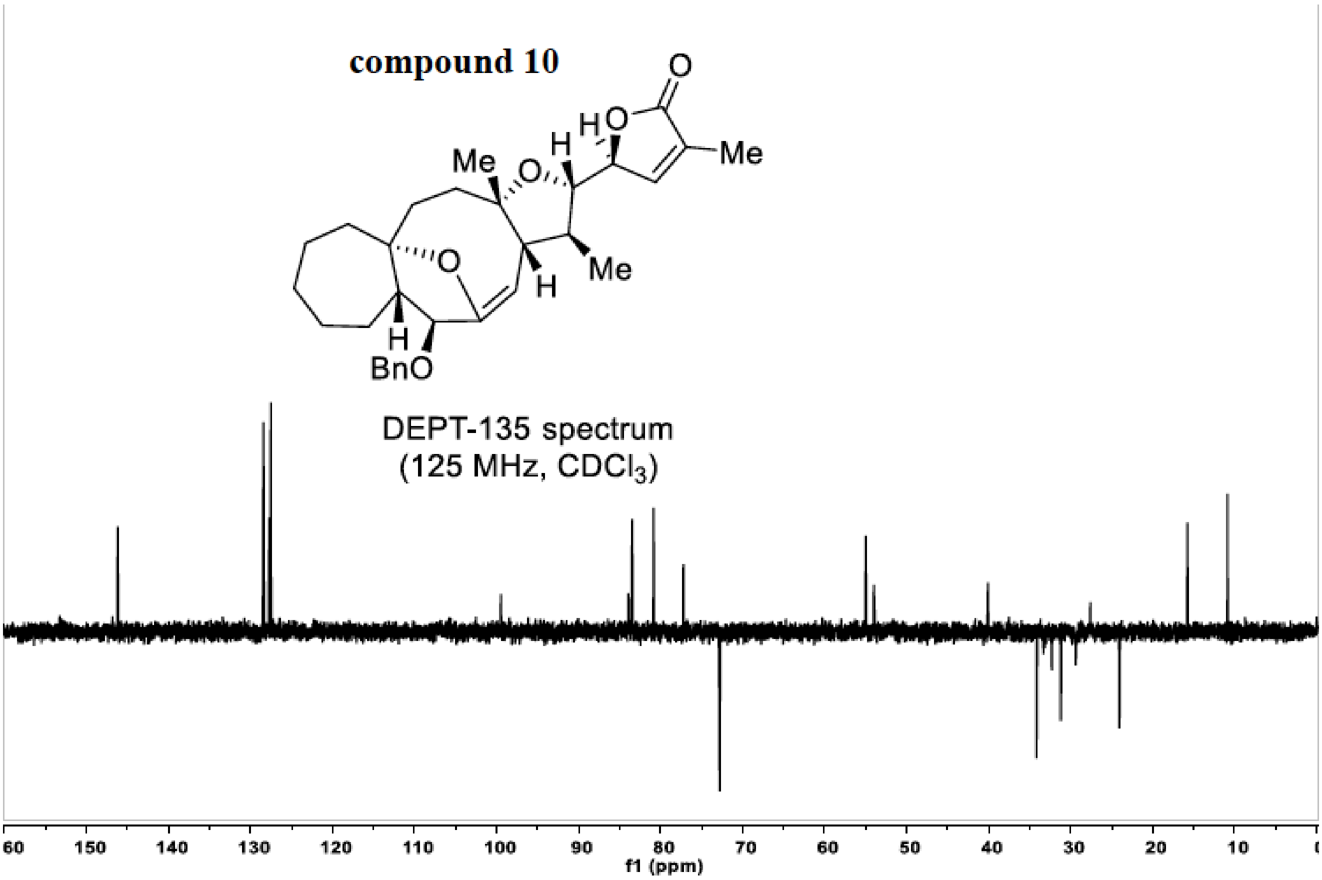
compound 10
Me
Me
Me
H
H
H
BnO
DEPT-135 spectrum
(125 MHz, CDCl3)
f1 (ppm)

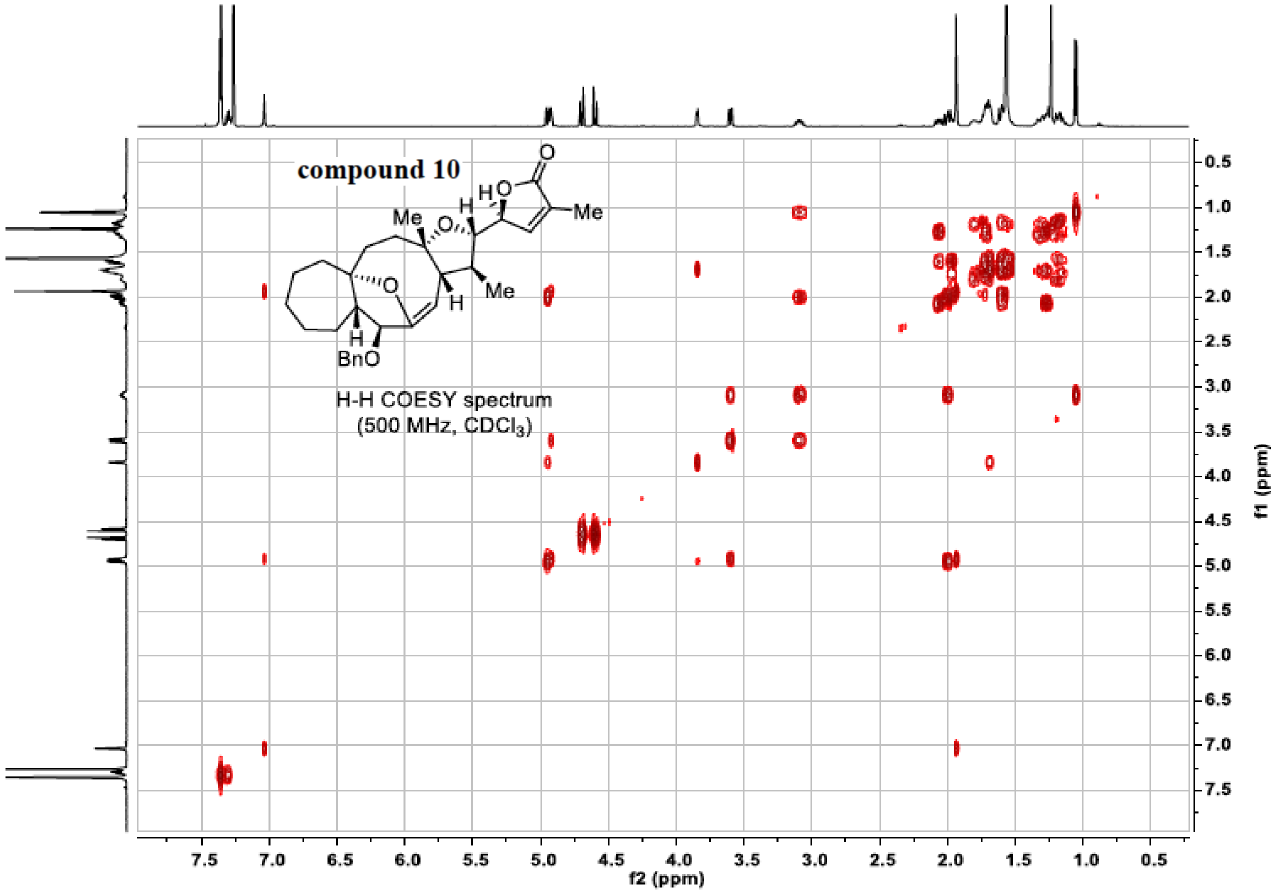
compound 10
Me
Me
Me
H
H
H
BnO
H-H COESY spectrum
(500 MHz, CDCl3)
f2 (ppm)
f1 (ppm)

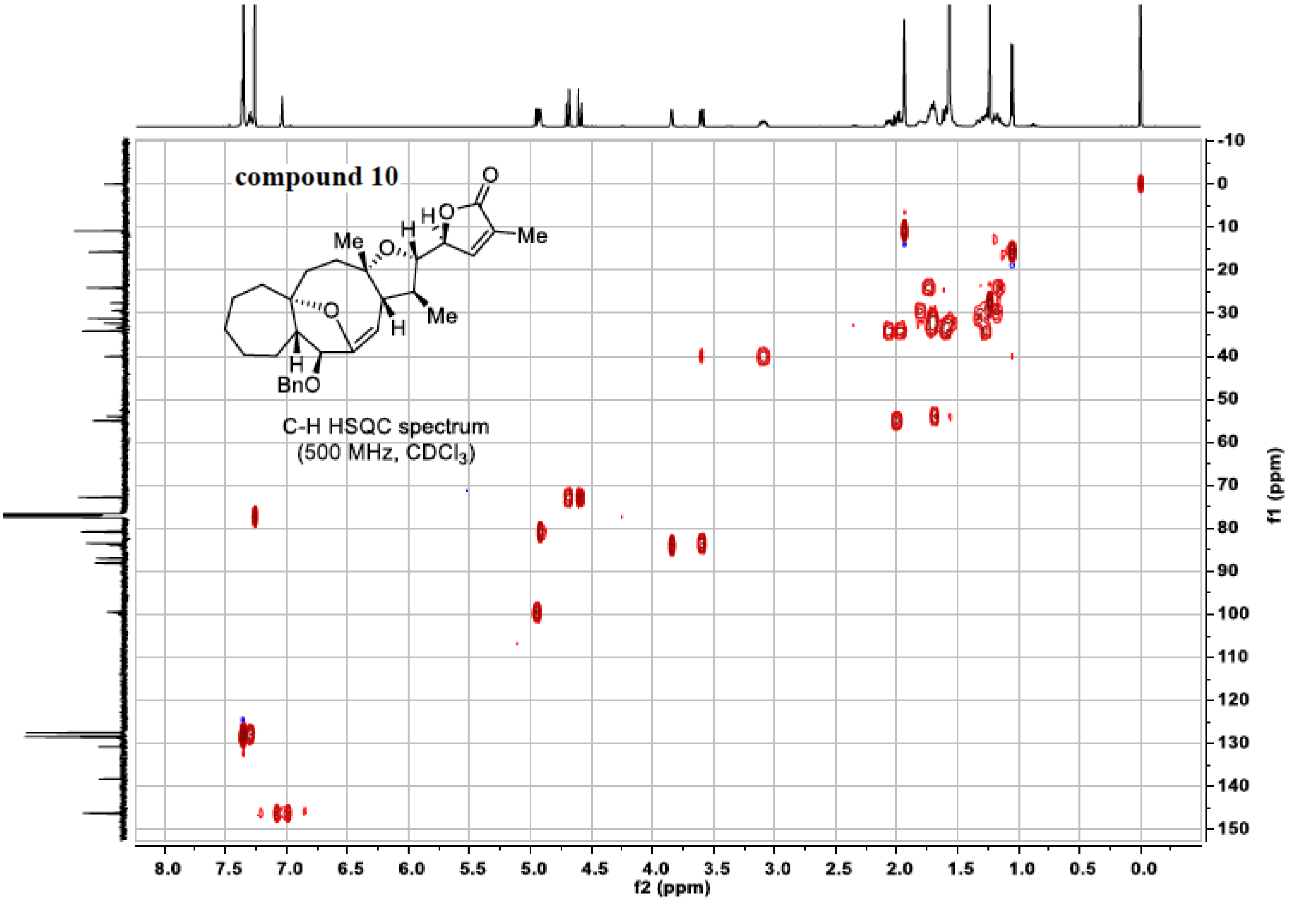
compound 10
C-H HSQC spectrum
(500 MHz, CDCl3)
f2 (ppm)
f1 (ppm)

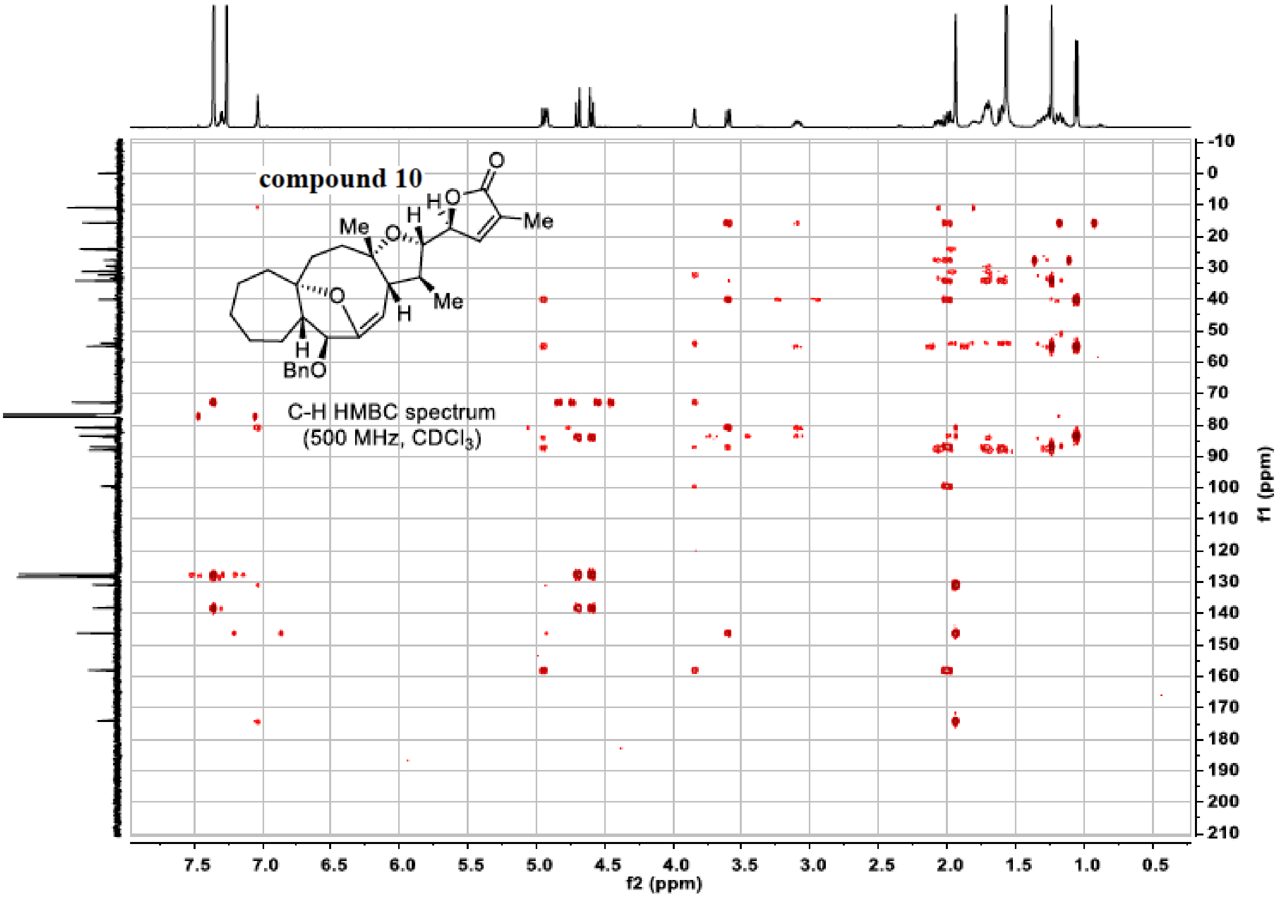
compound 10
C-H HMBC spectrum
(500 MHz, CDCl3)
f2 (ppm)
f1 (ppm)

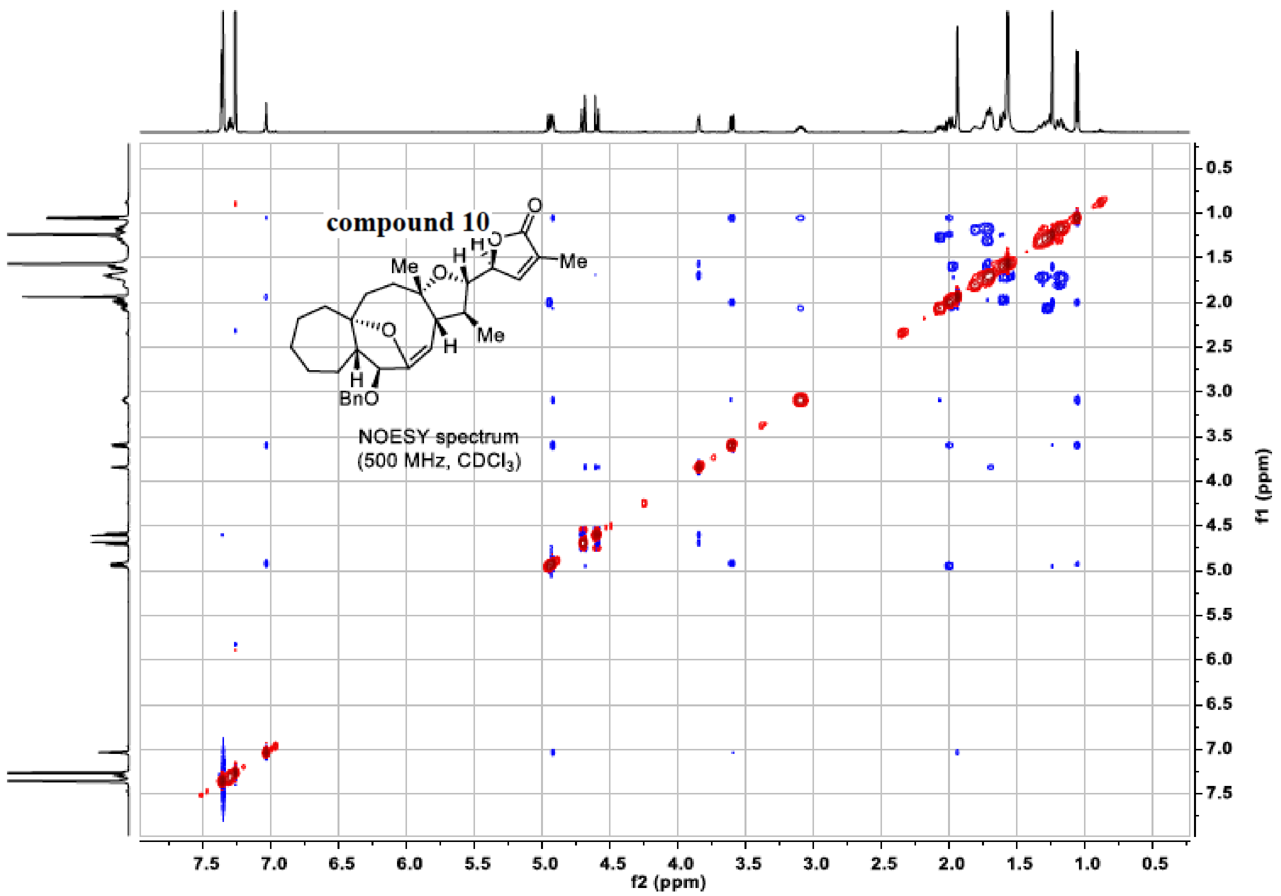

Supplementary Figure 3 | ^{1}H, ^{13}C, DEPT135, COESY, HSQC, HMBC, NOESY Spectra for Compound 10

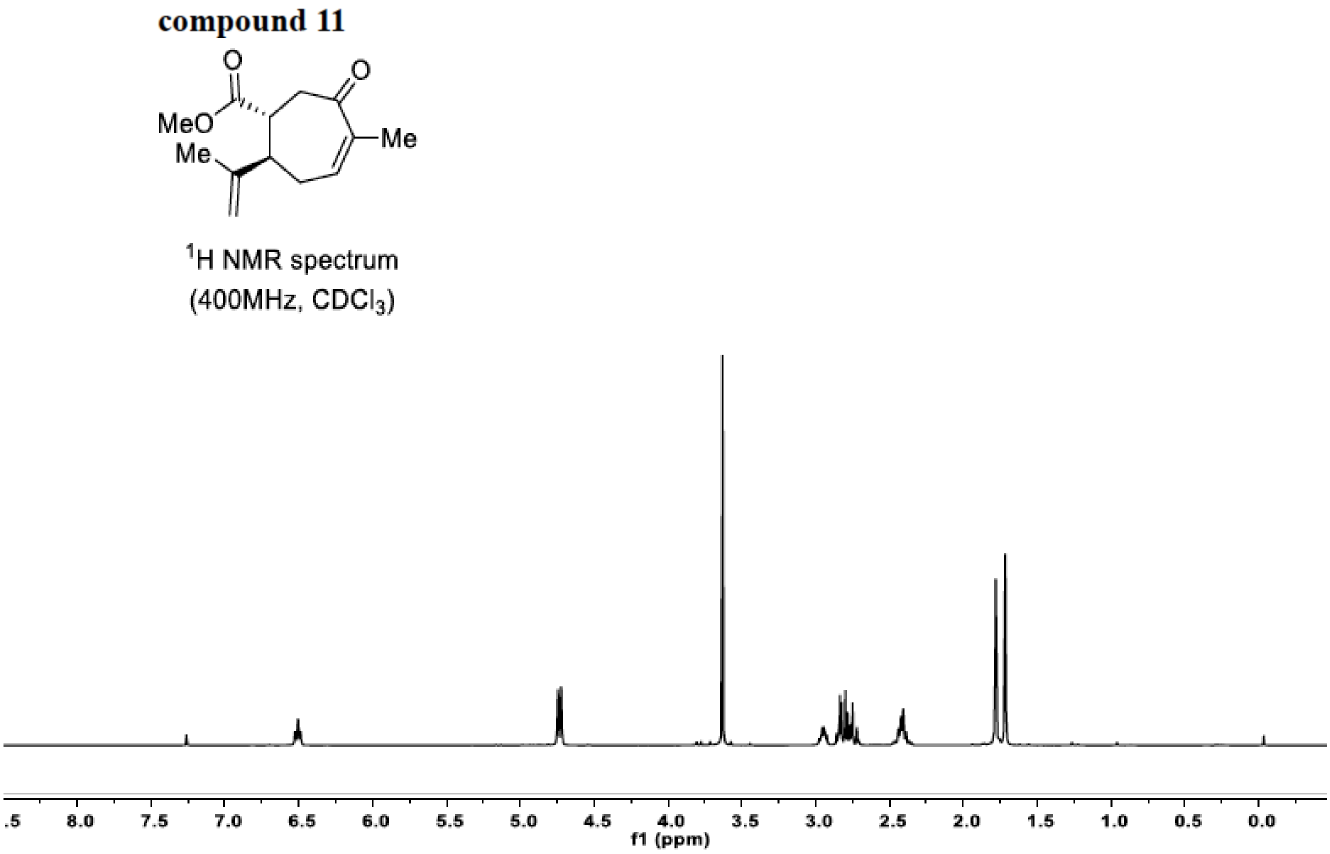

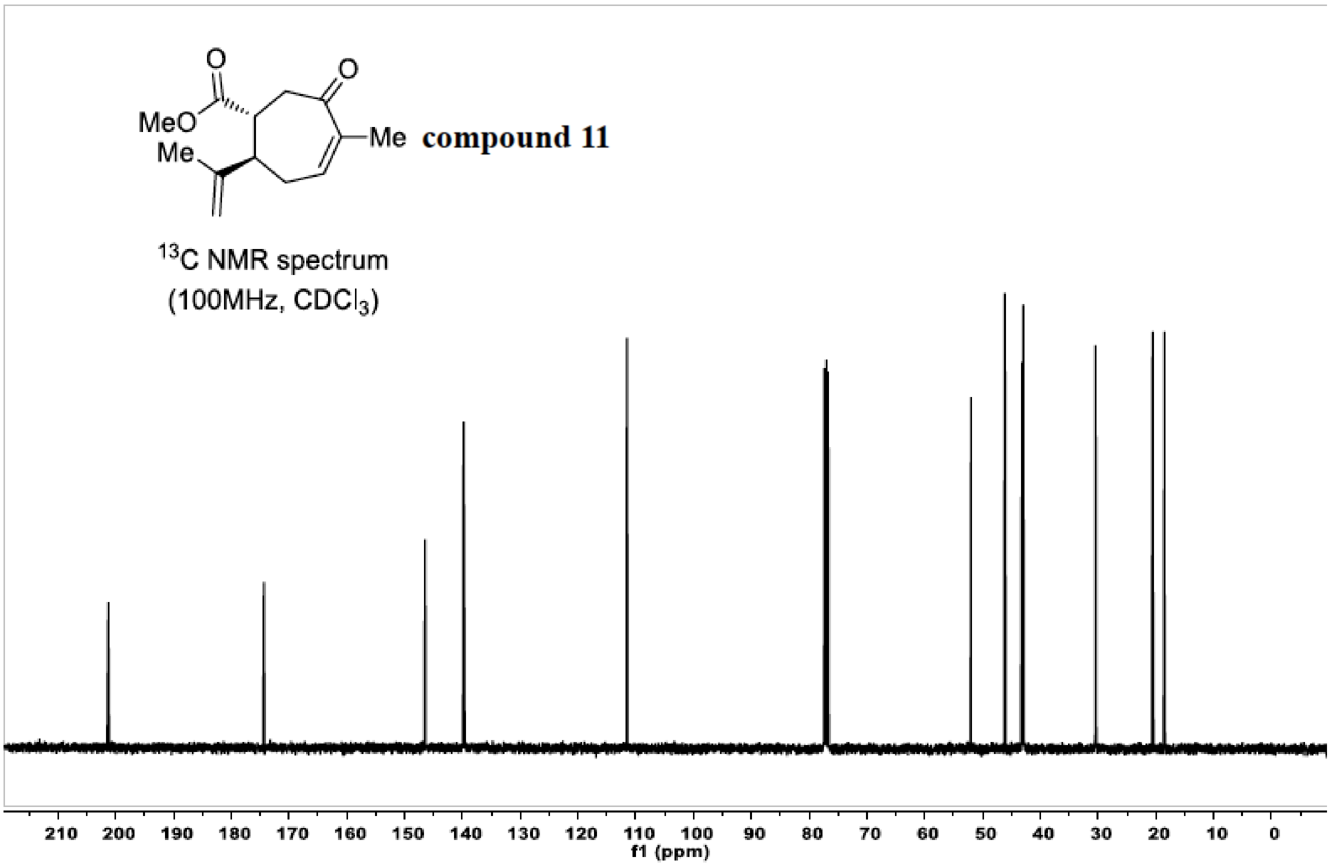

Supplementary Figure 4 | ^{1}H, ^{13}C NMR Spectra for Compound 11

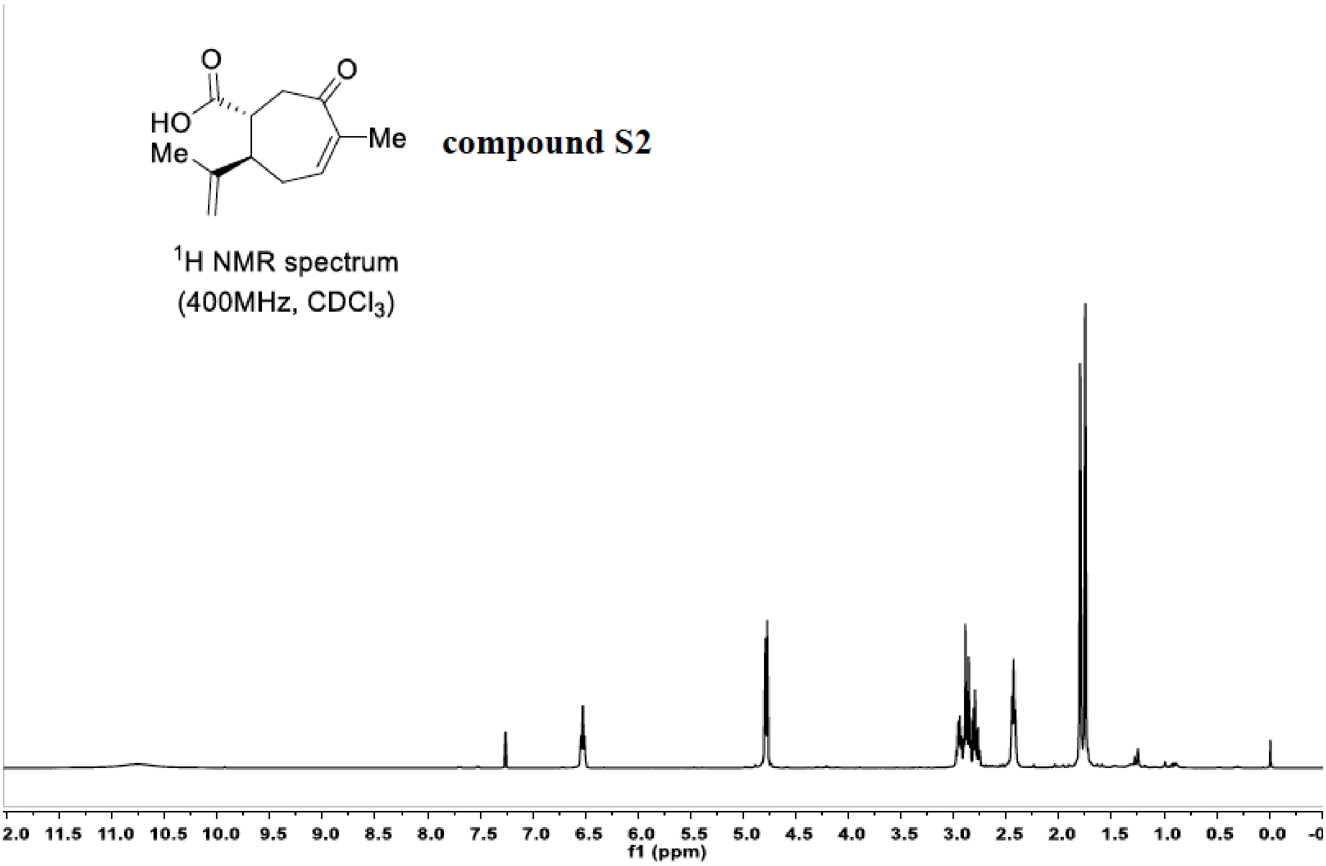

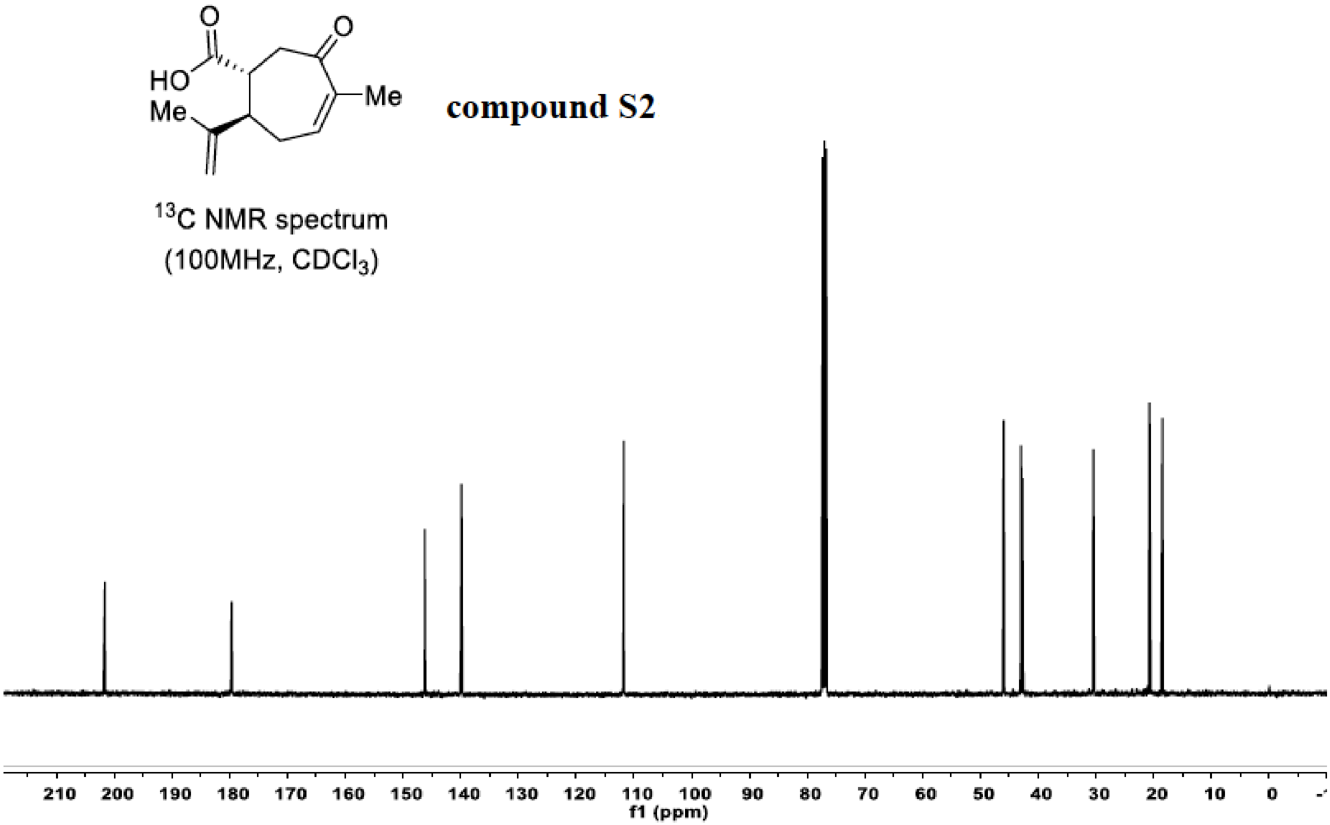

Supplementary Figure 5 | ^{1}H, ^{13}C NMR Spectra for Compound S2

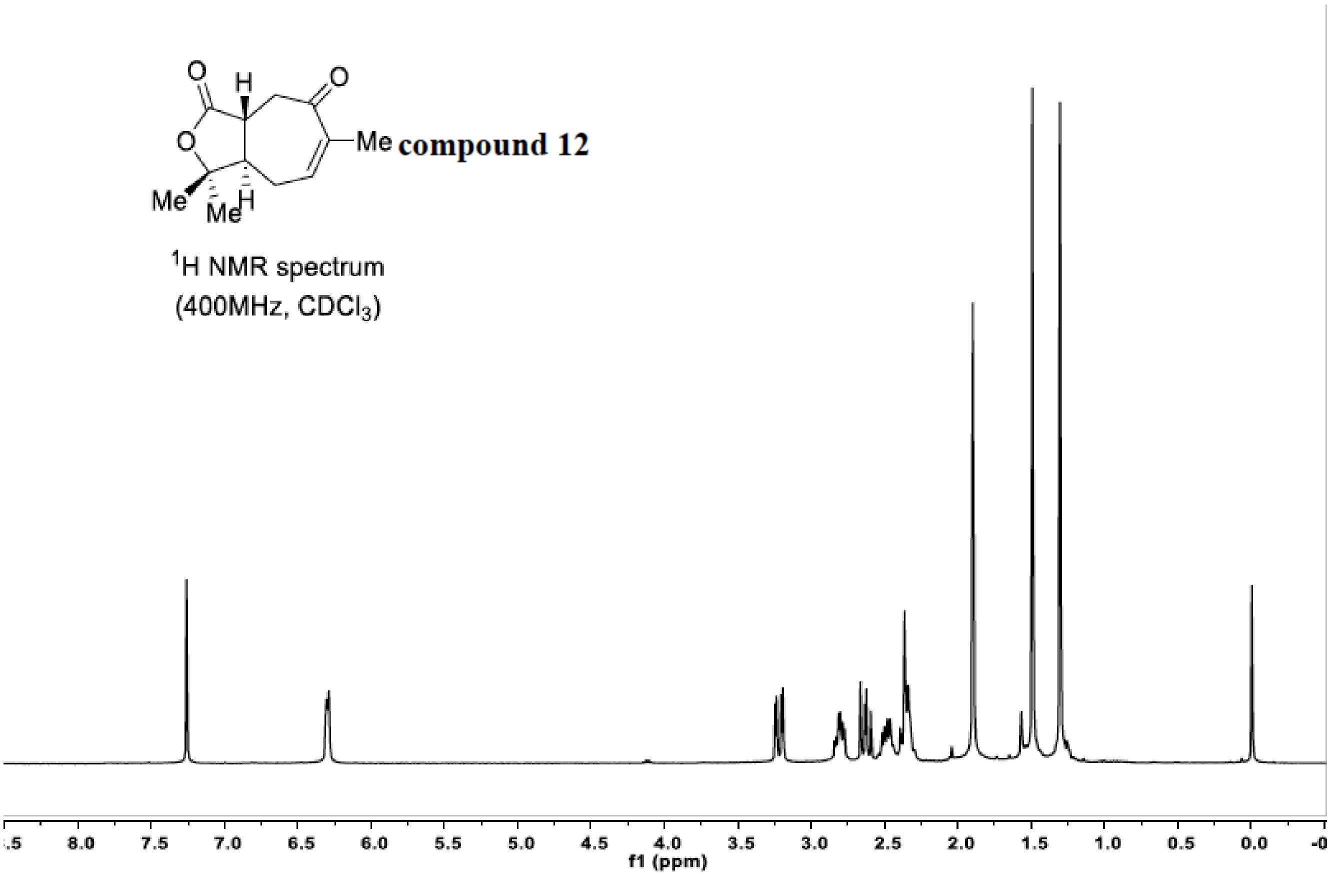

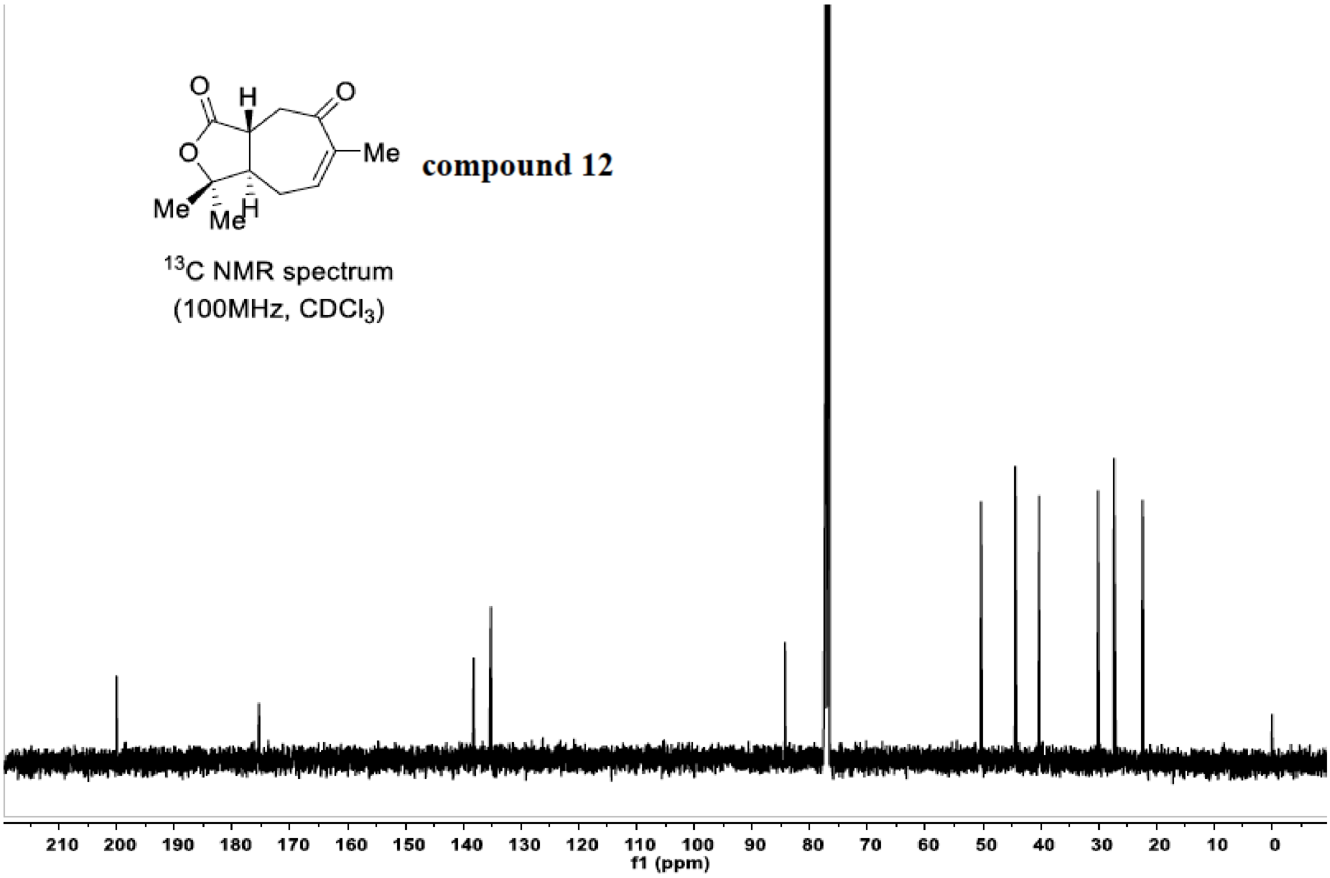

Supplementary Figure 6 | ^{1}H, ^{13}C NMR Spectra for Compound 12

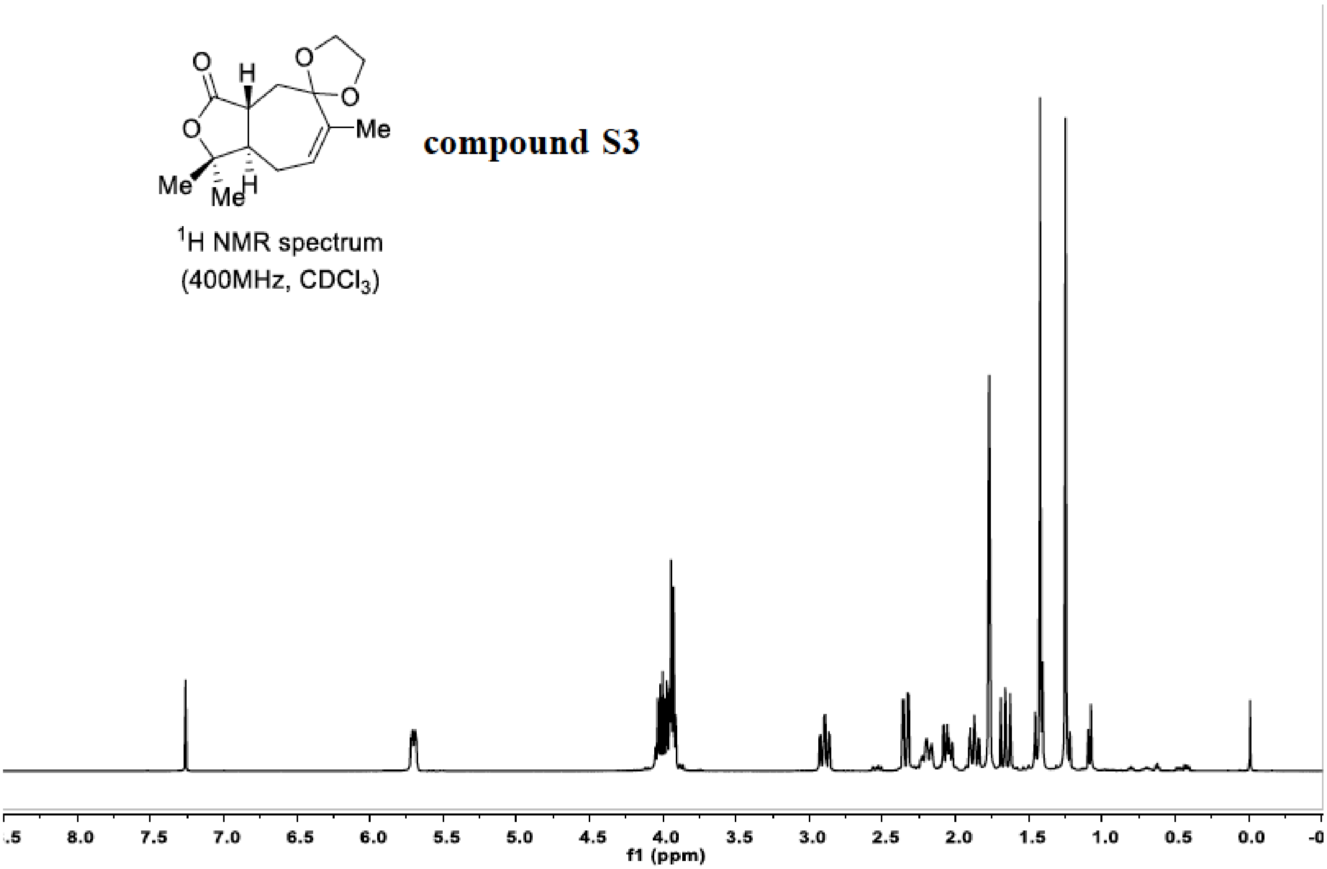

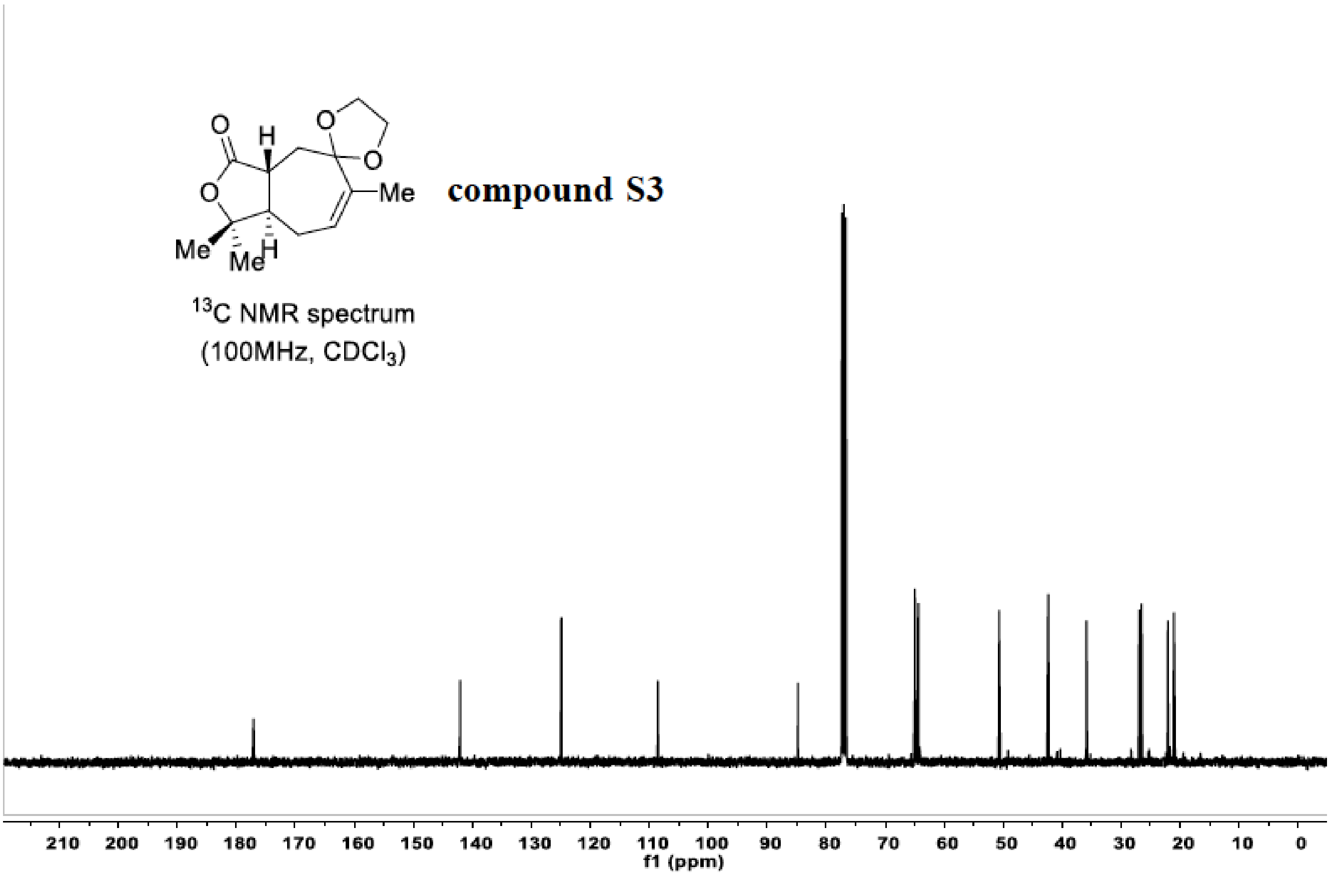

Supplementary Figure 7 | ^{1}H, ^{13}C NMR Spectra for Compound S3

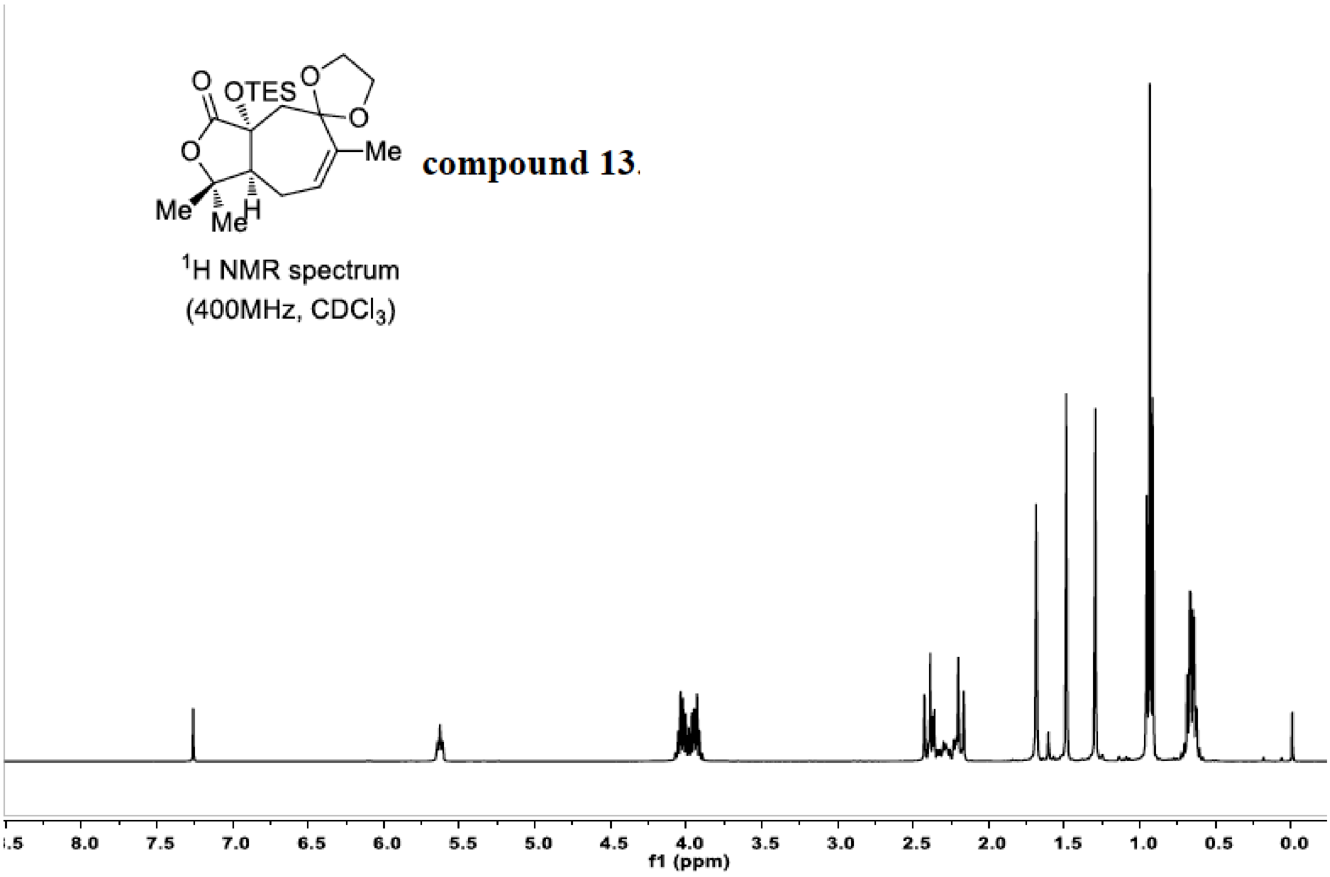

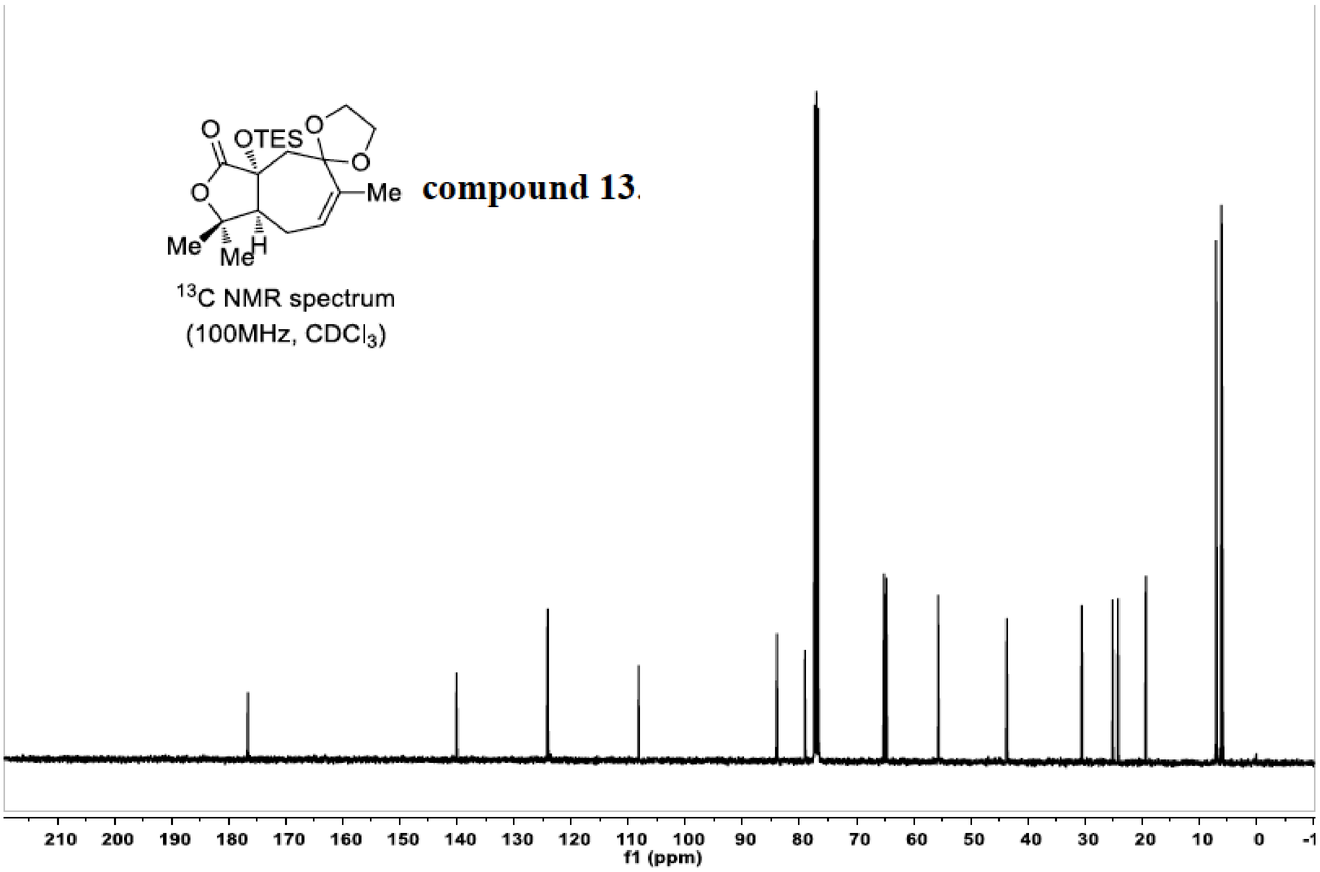

Supplementary Figure 8 | ^{1}H, ^{13}C NMR Spectra for Compound 13

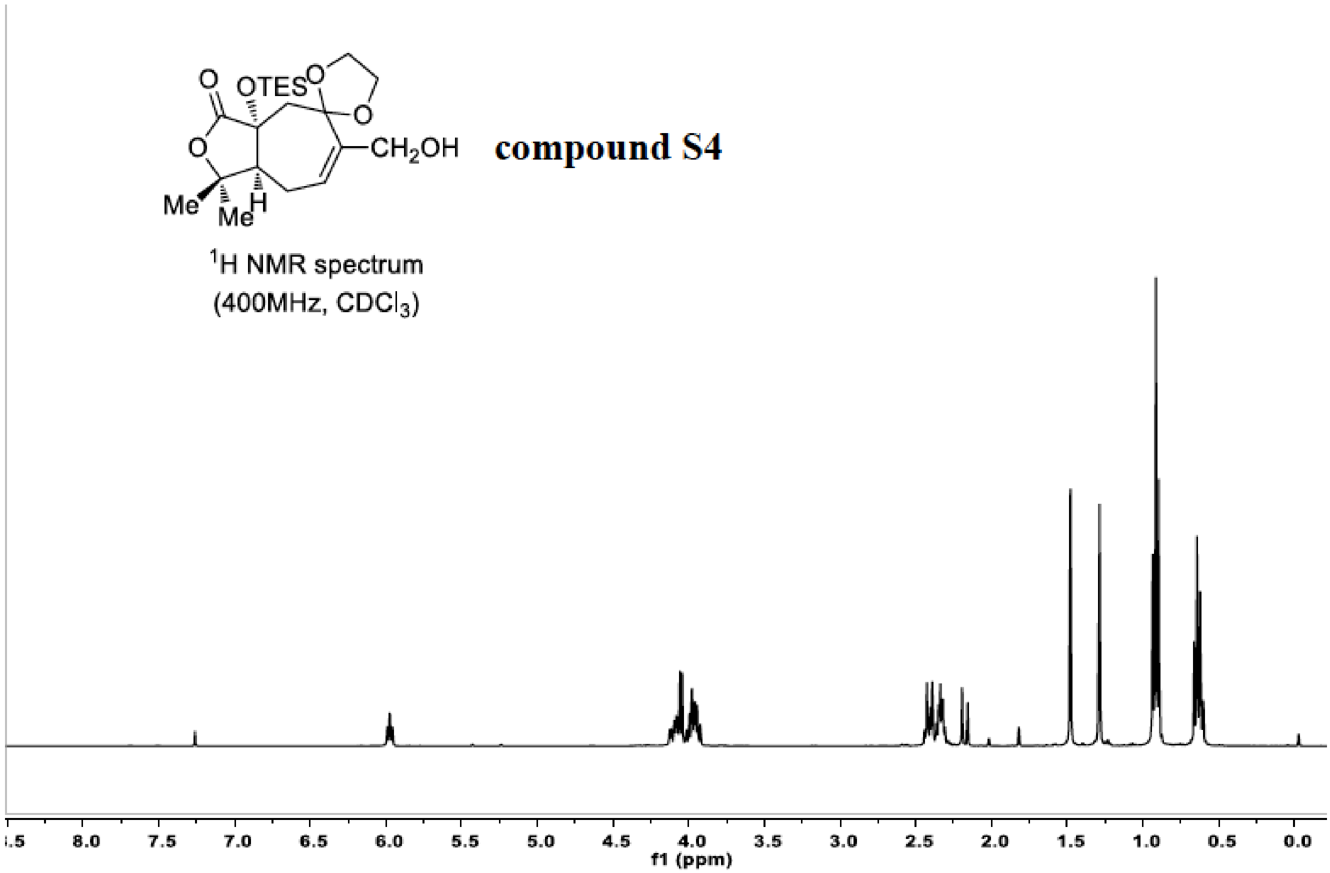

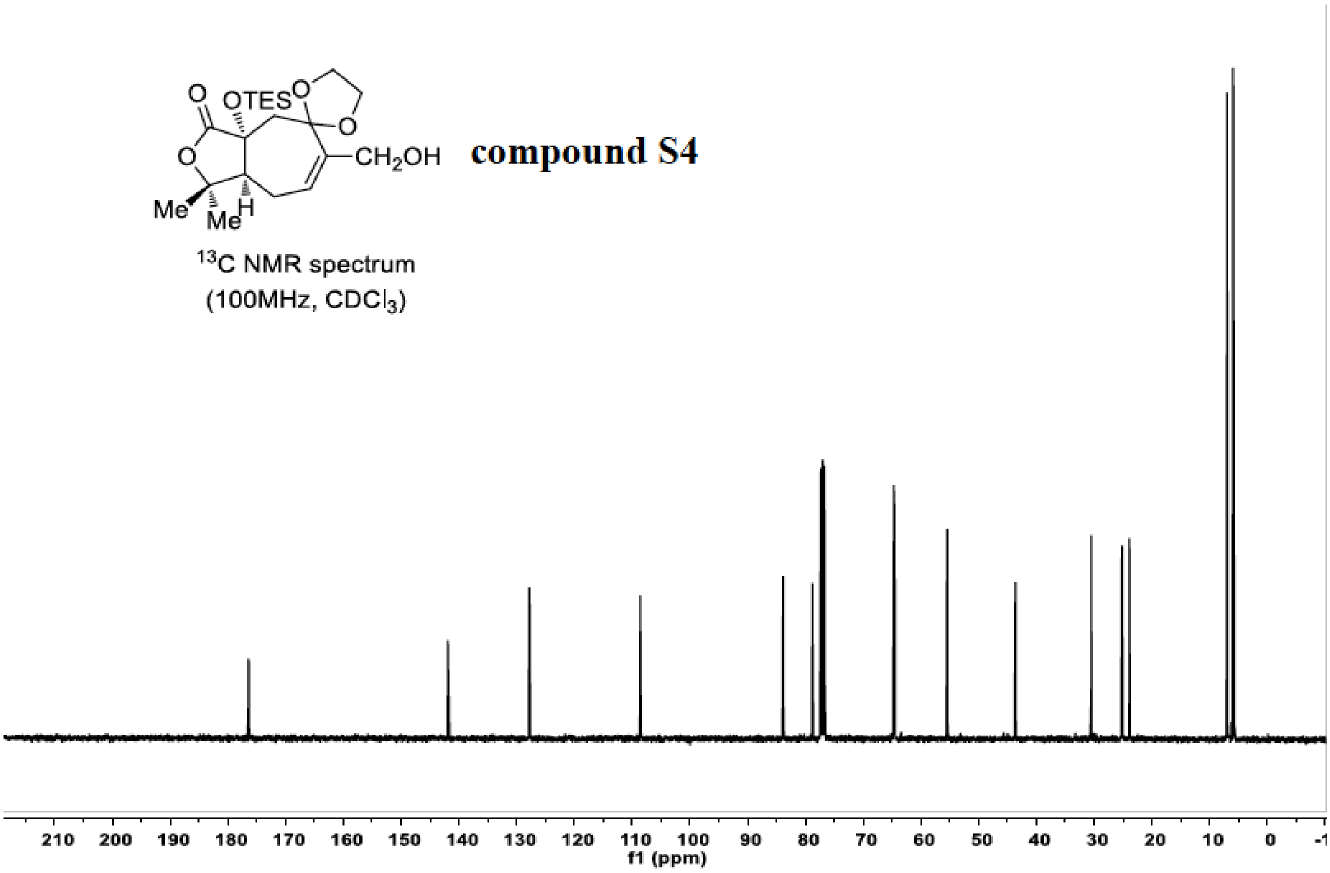

Supplementary Figure 9 | ^{1}H, ^{13}C NMR Spectra for Compound S4

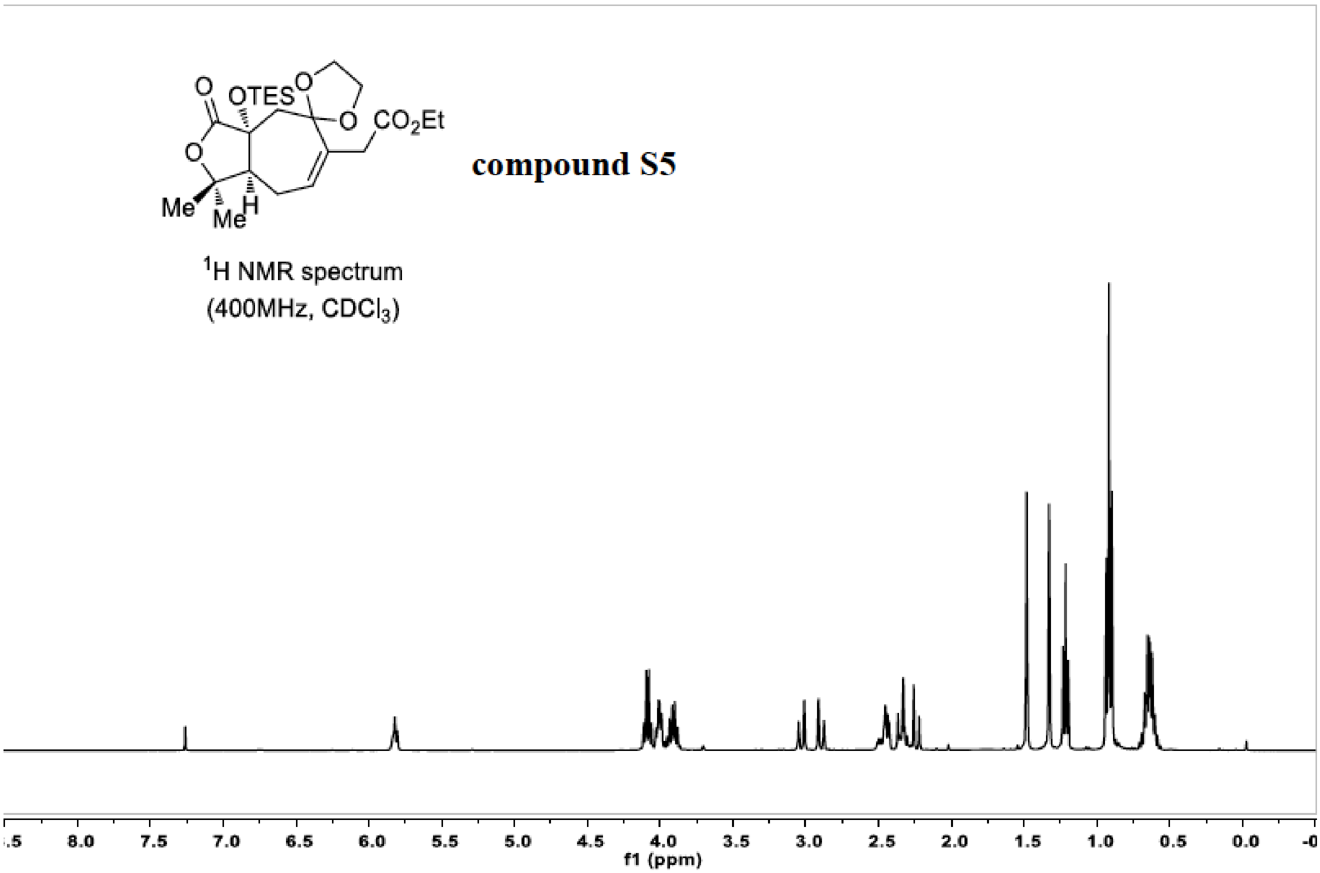

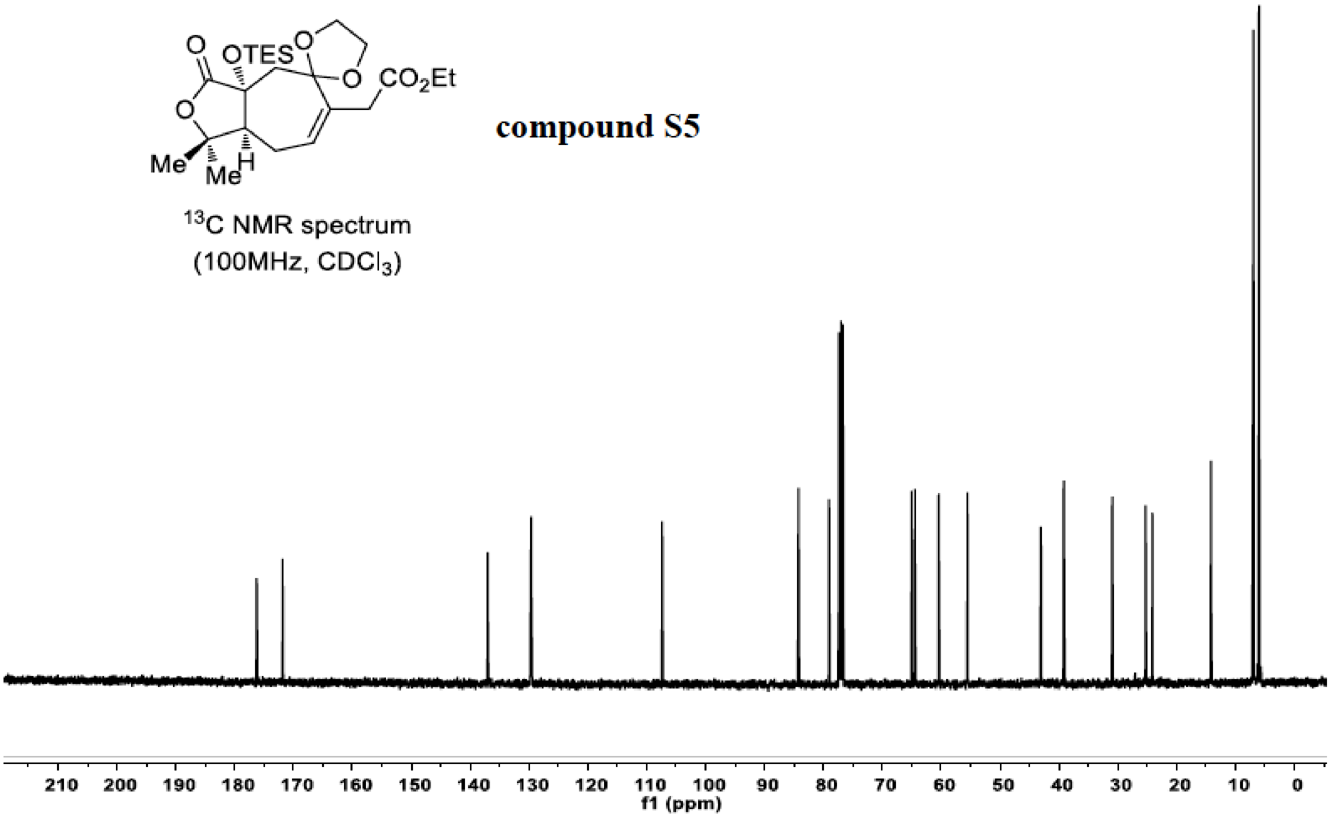

Supplementary Figure 10 | ^{1}H, ^{13}C NMR Spectra for Compound S5

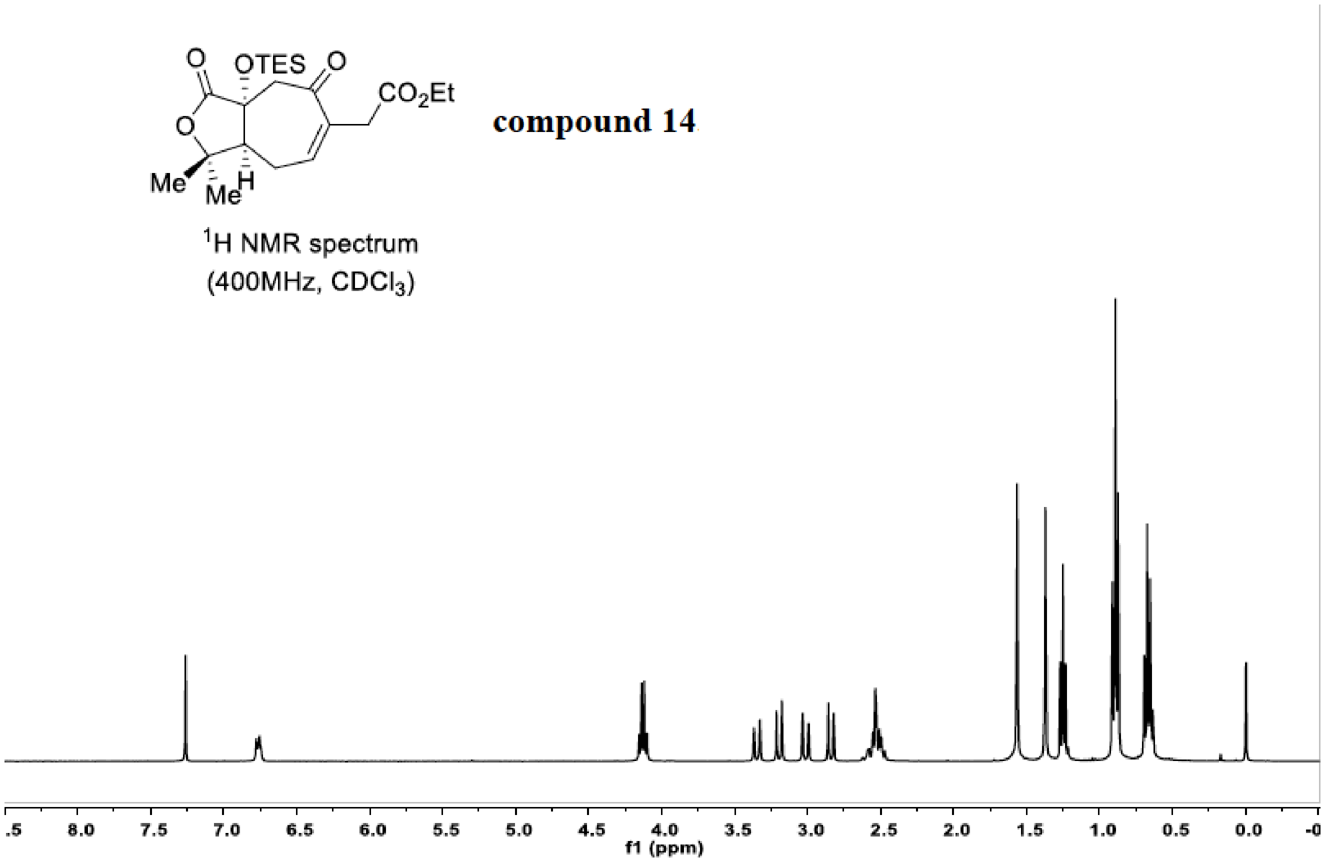

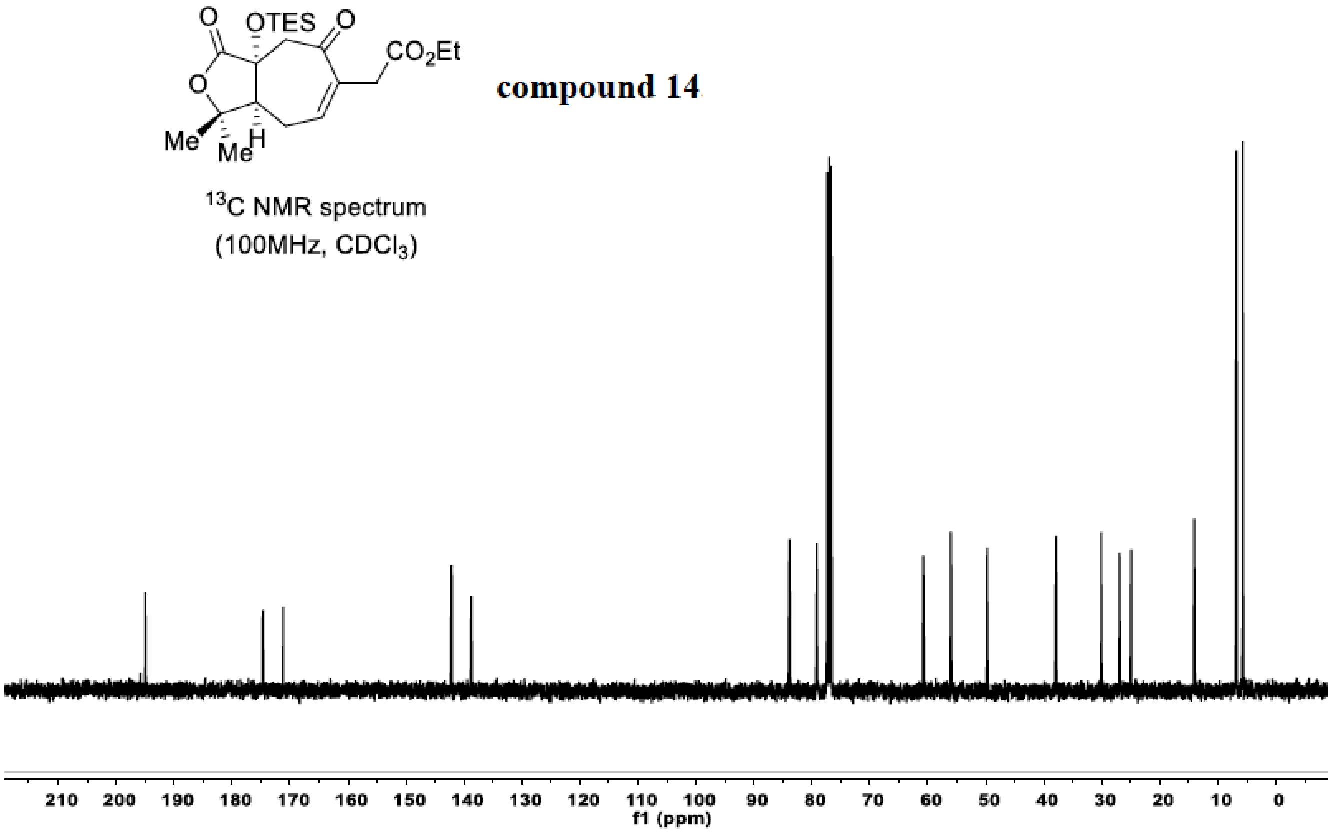

Supplementary Figure 11 | ^{1}H, ^{13}C NMR Spectra for Compound 14

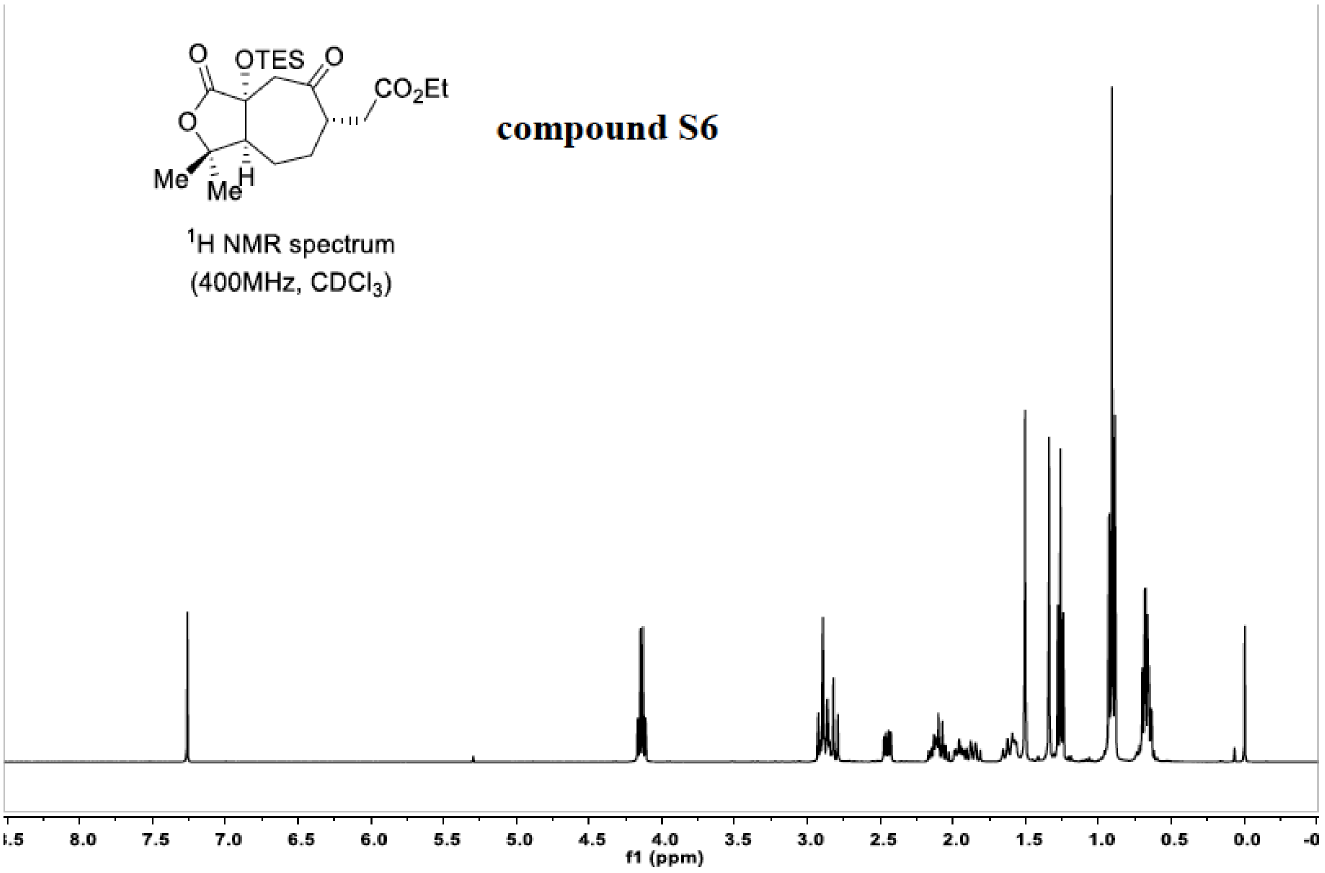

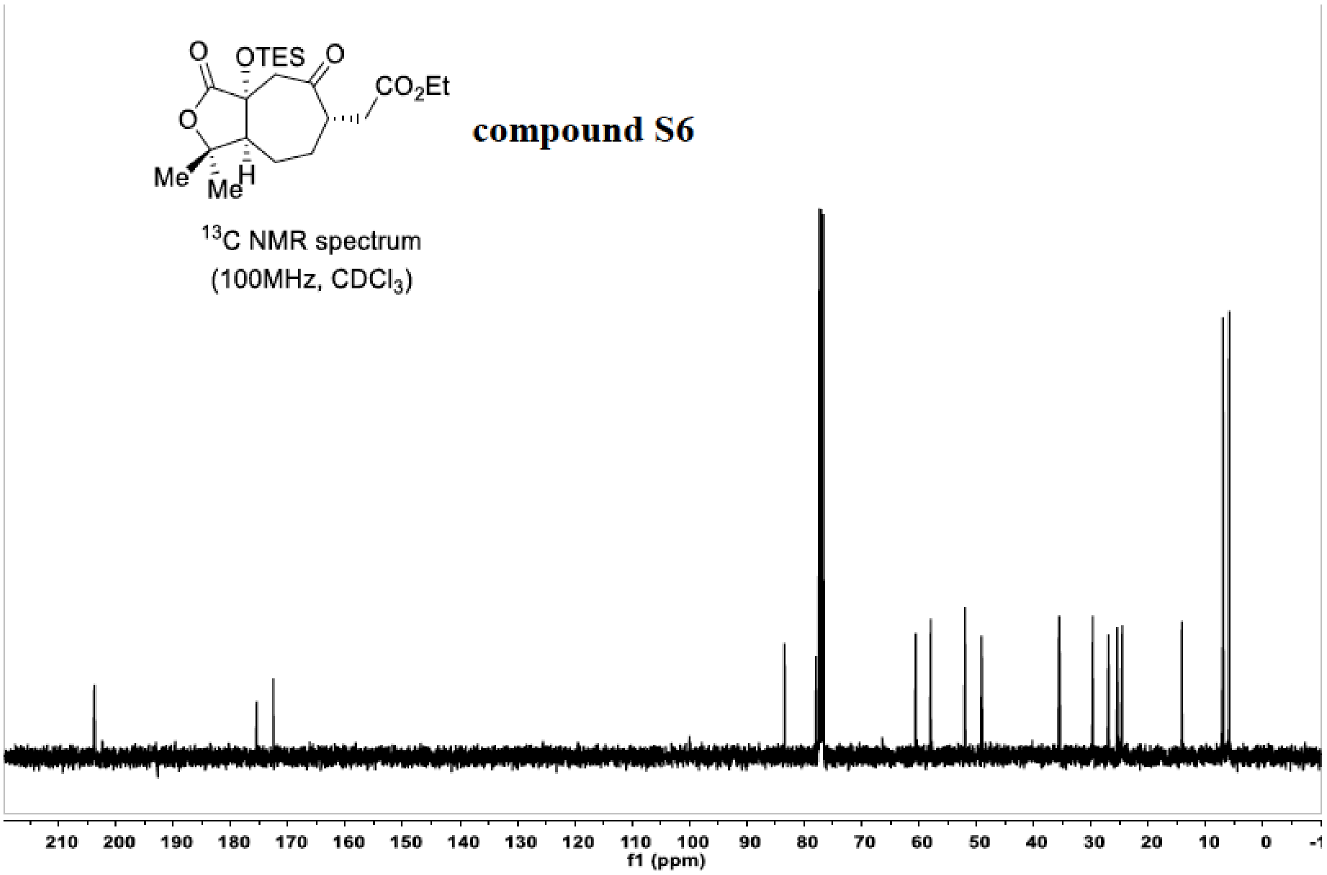

Supplementary Figure 12 | ^{1}H, ^{13}C NMR Spectra for Compound S6

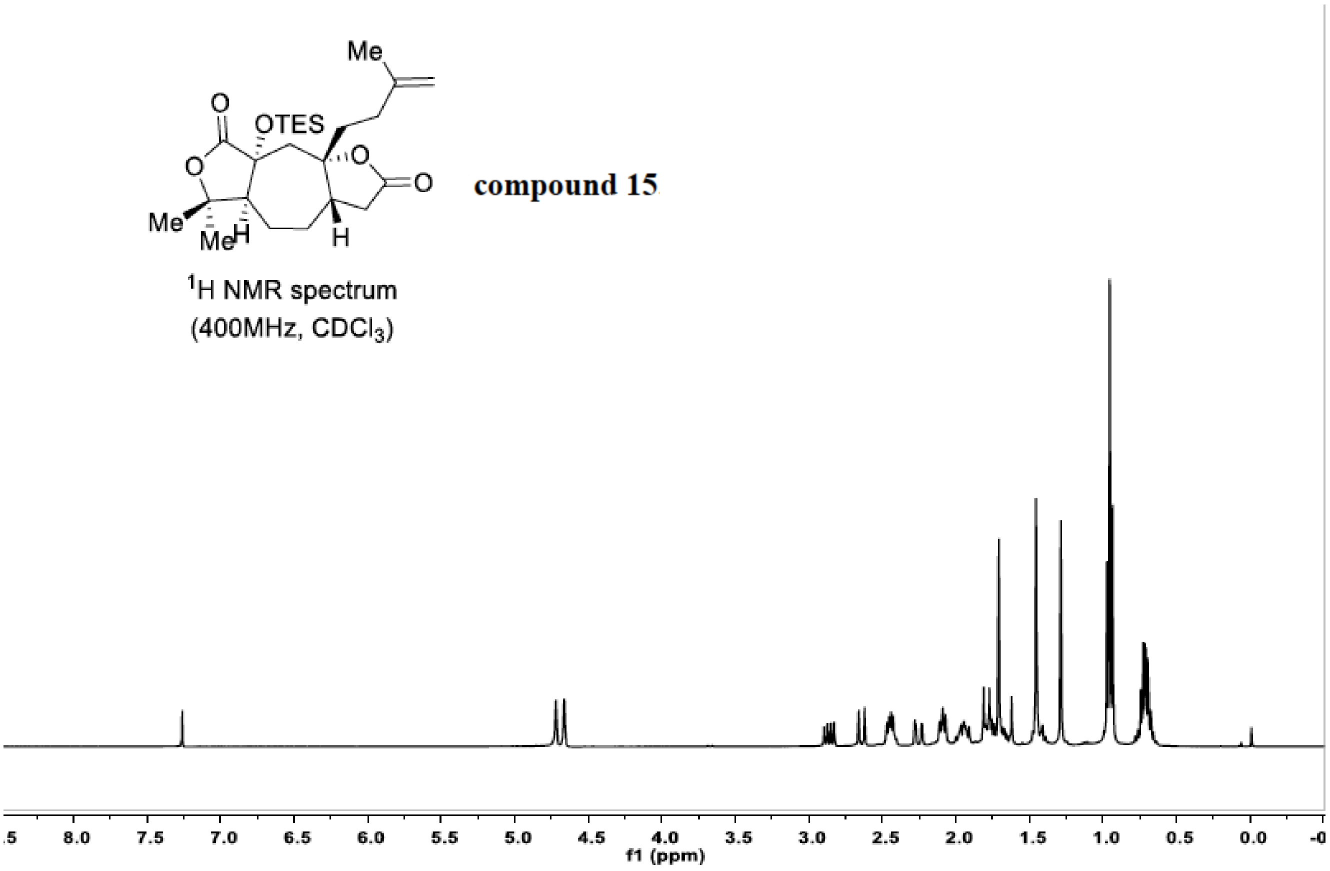

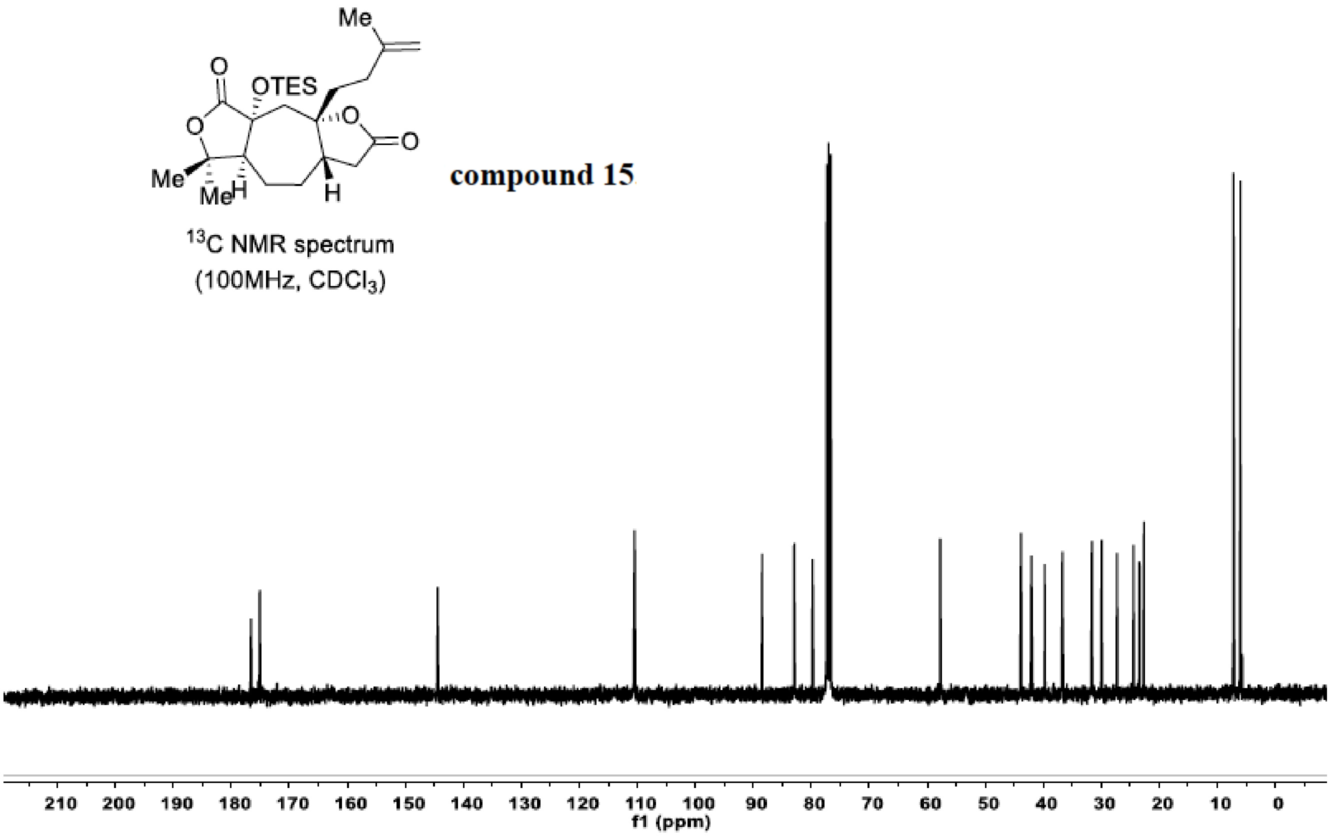

Supplementary Figure 13 | ^{1}H, ^{13}C NMR Spectra for Compound 15

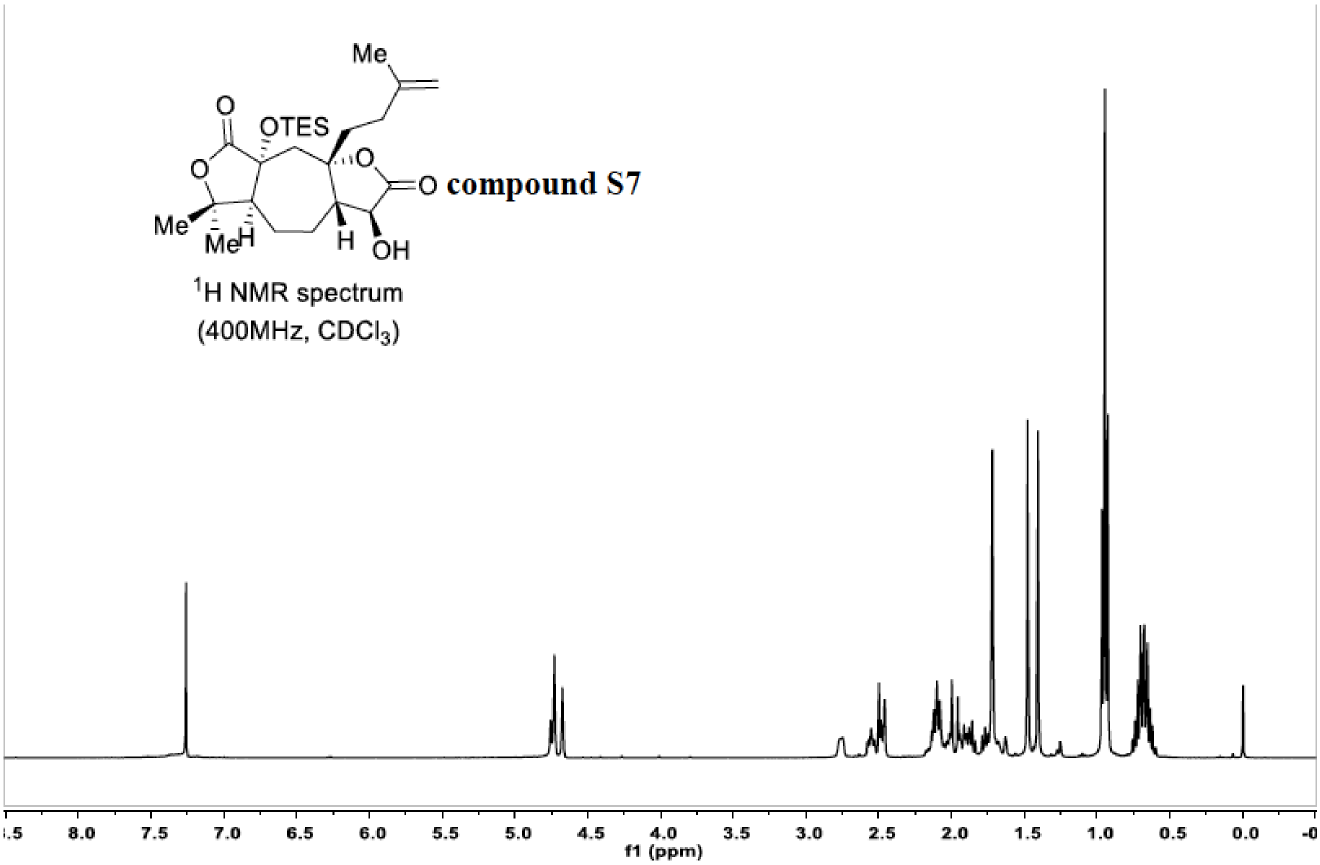

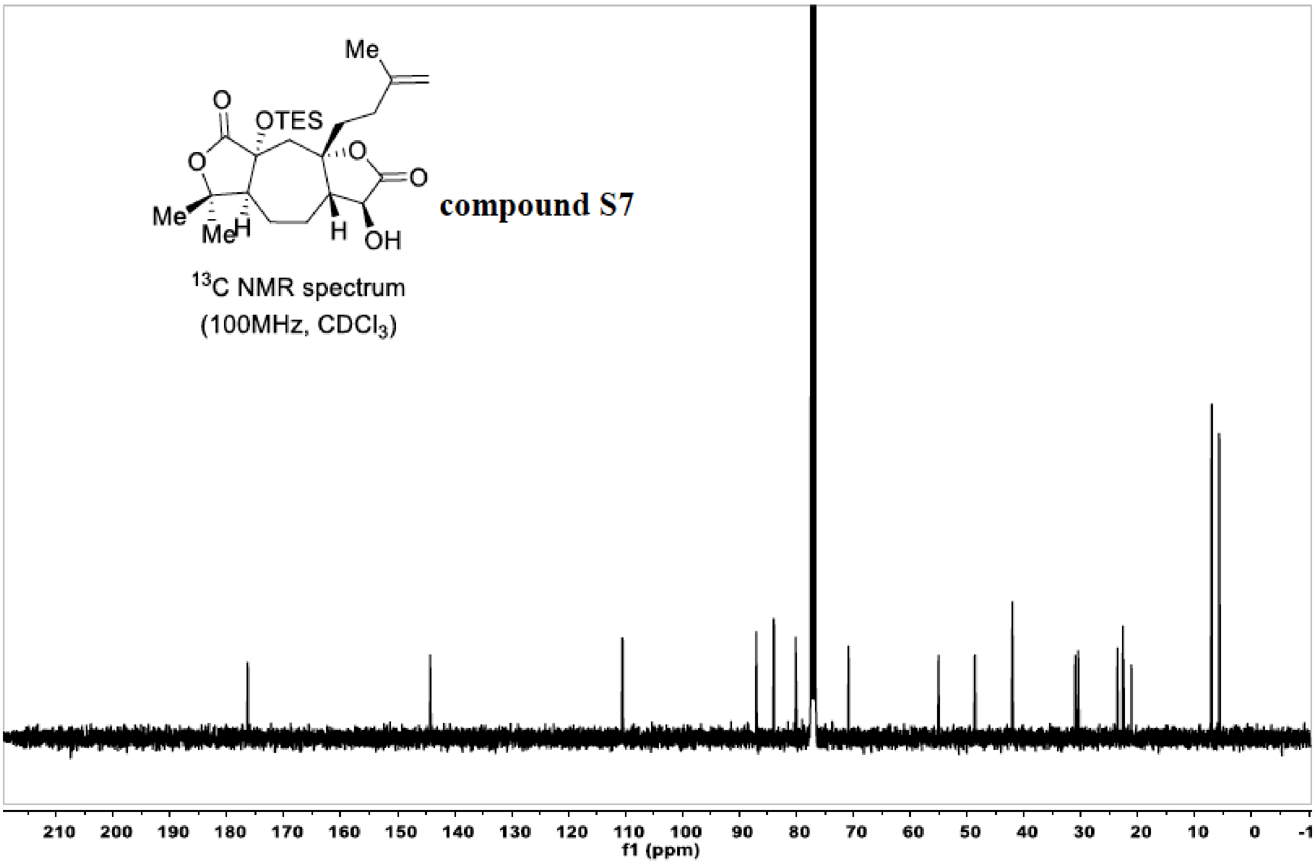

Supplementary Figure 14 | ^{1}H, ^{13}C NMR Spectra for Compound S7

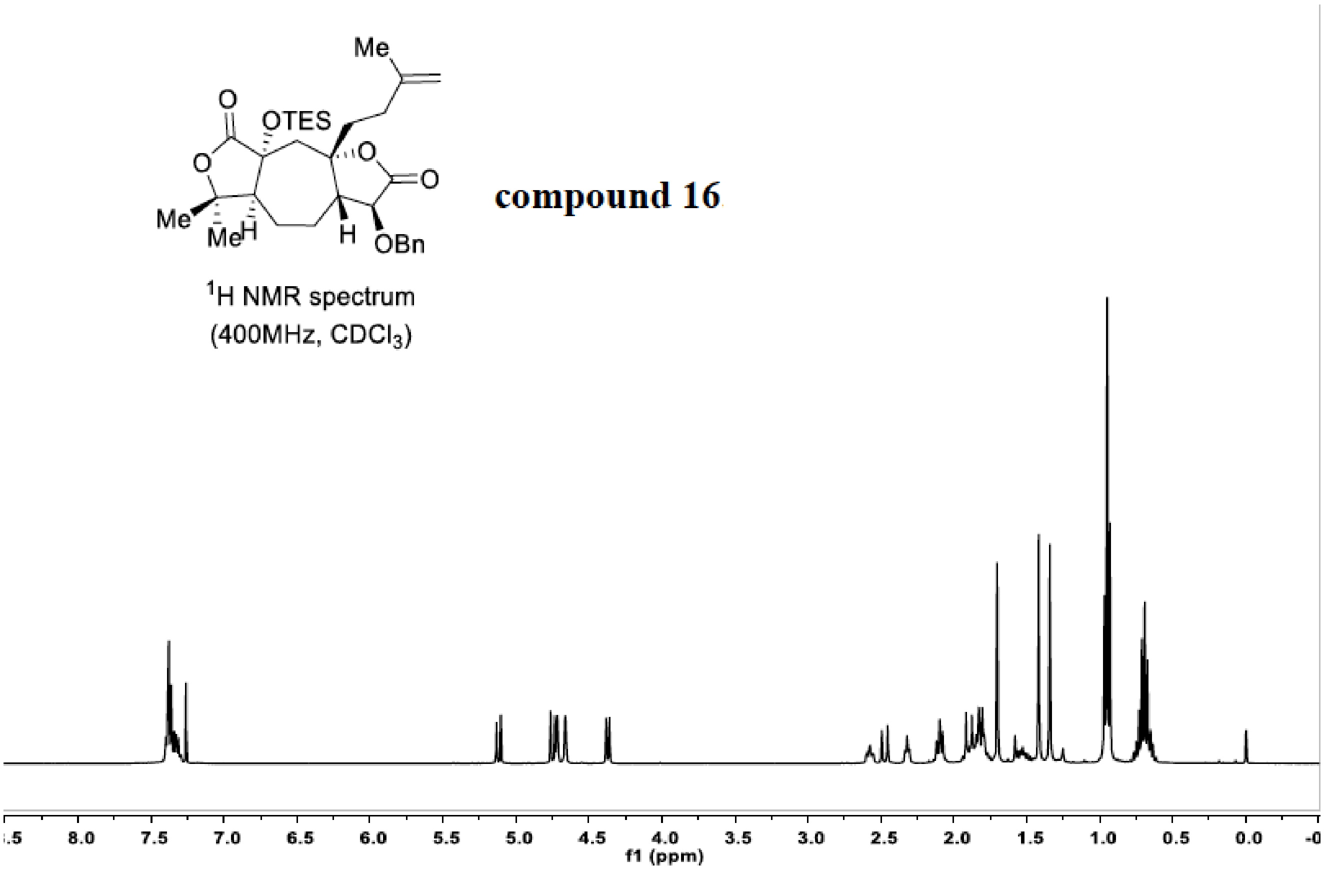

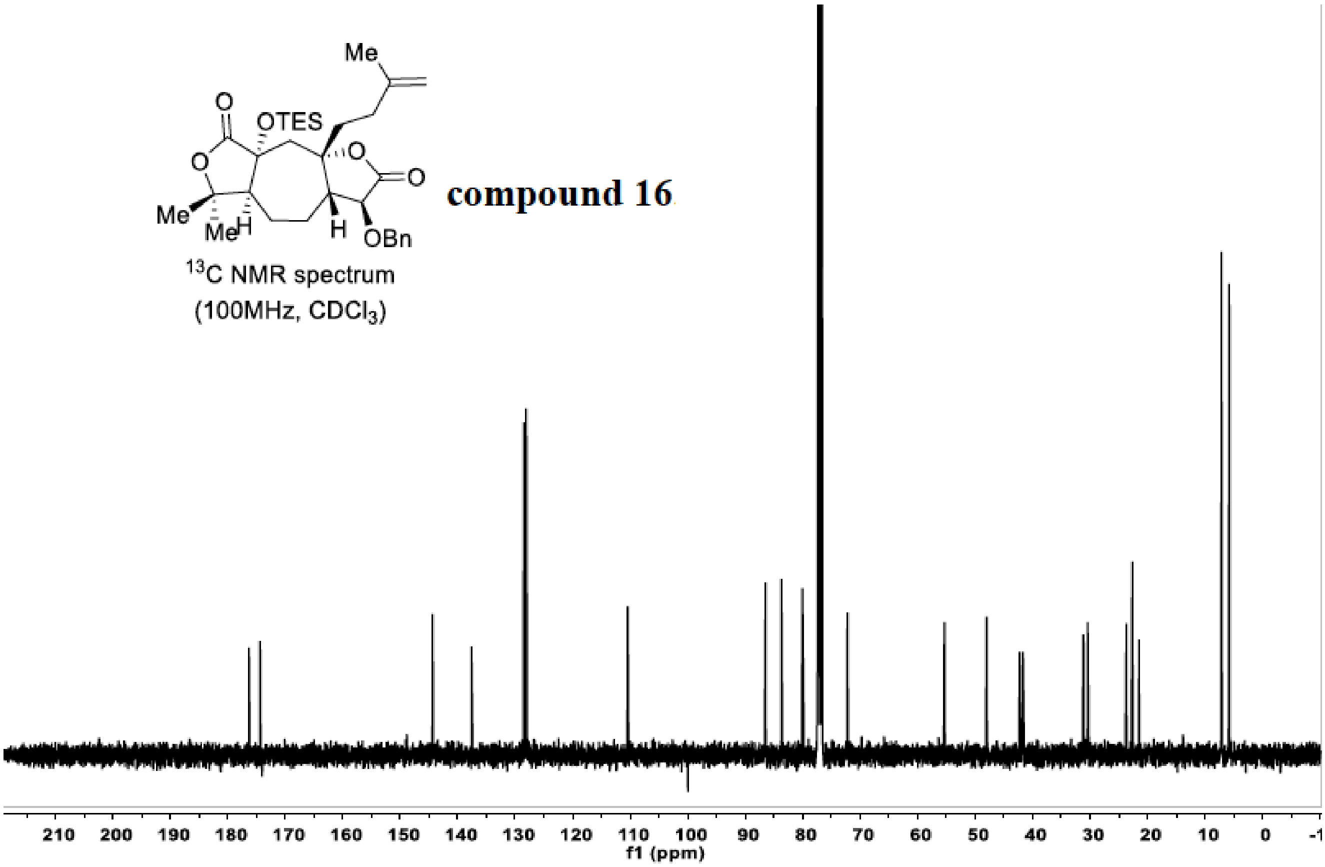

Supplementary Figure 15 | ^{1}H, ^{13}C NMR Spectra for Compound 16

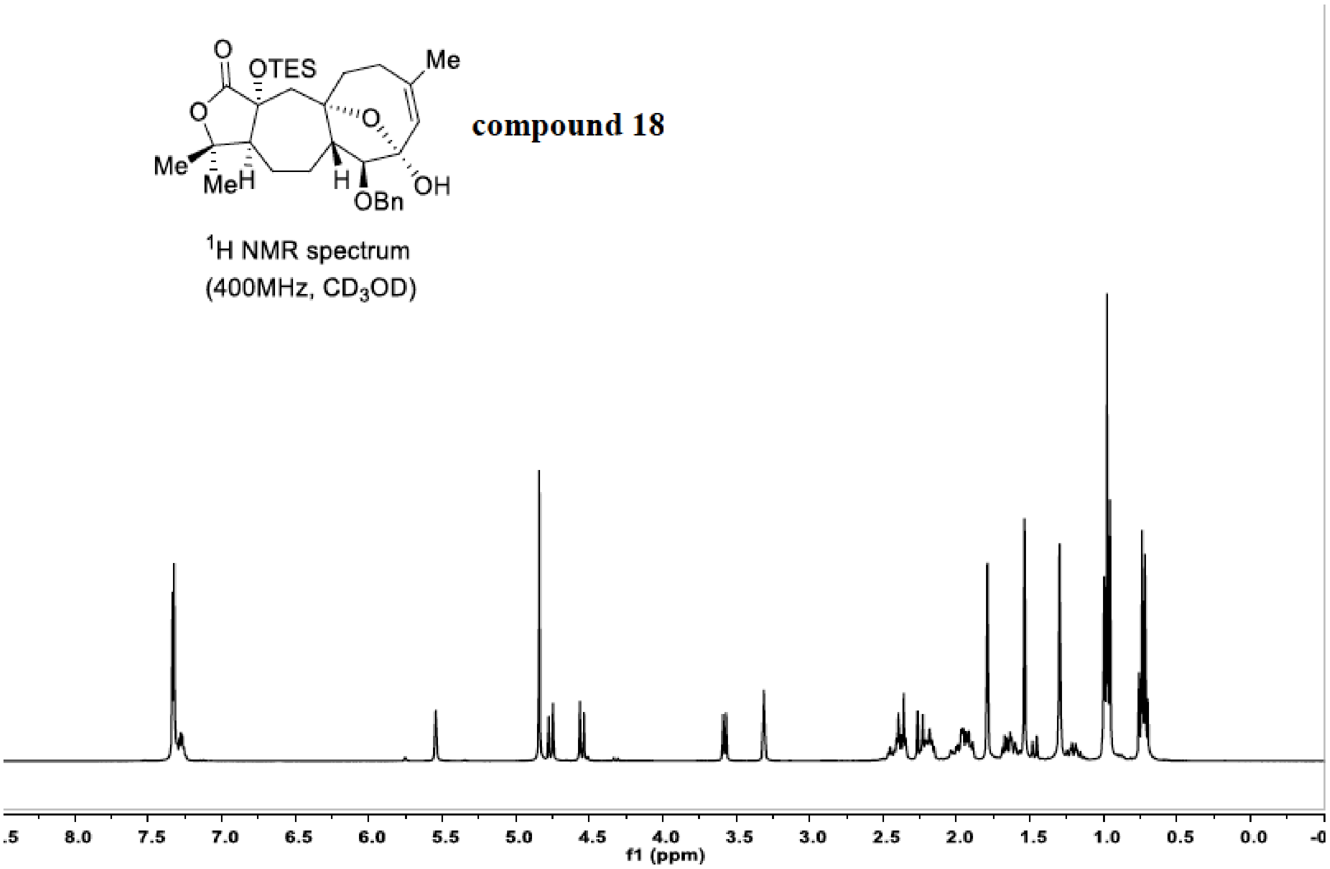

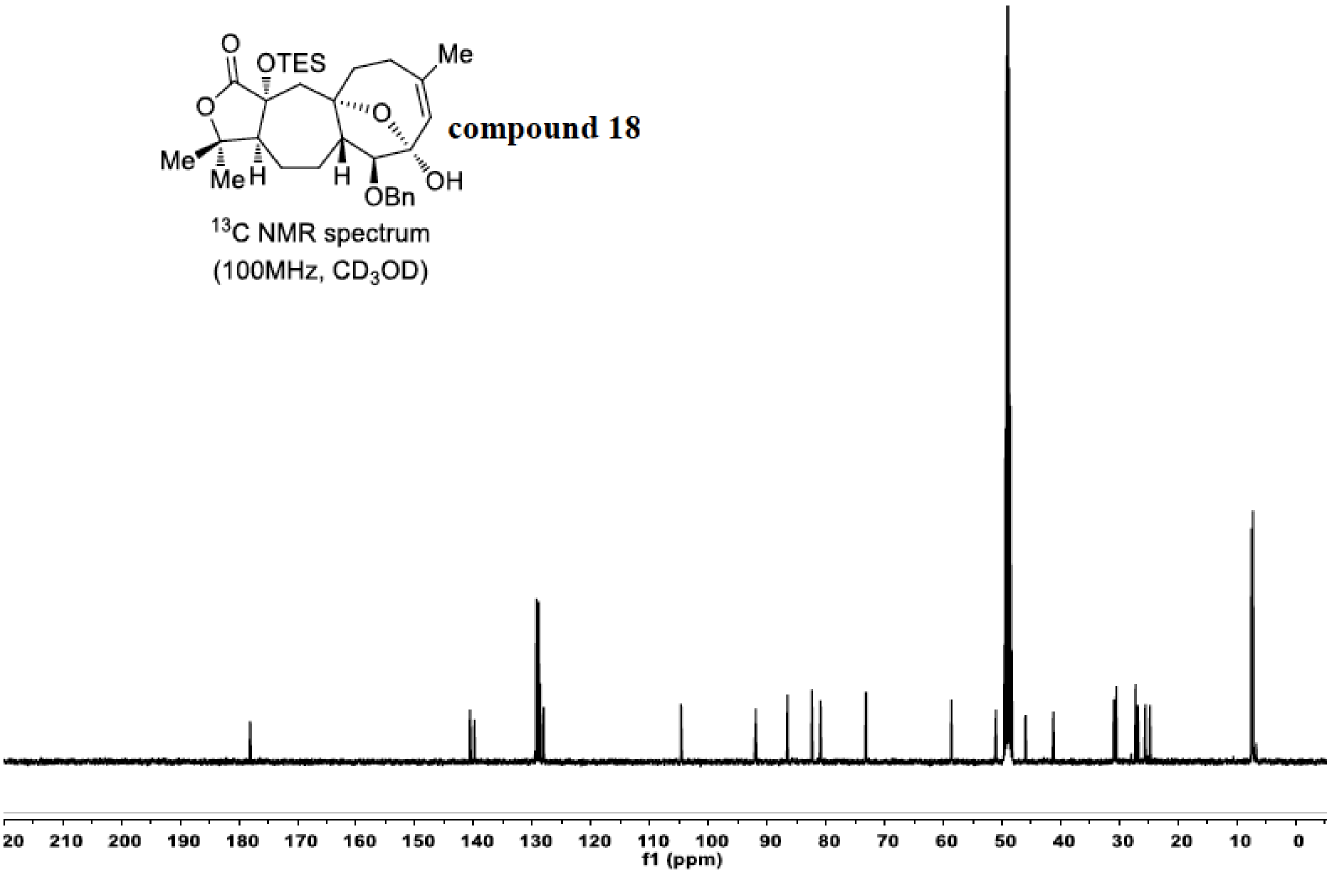

Supplementary Figure 16 | ^{1}H, ^{13}C NMR Spectra for Compound 18

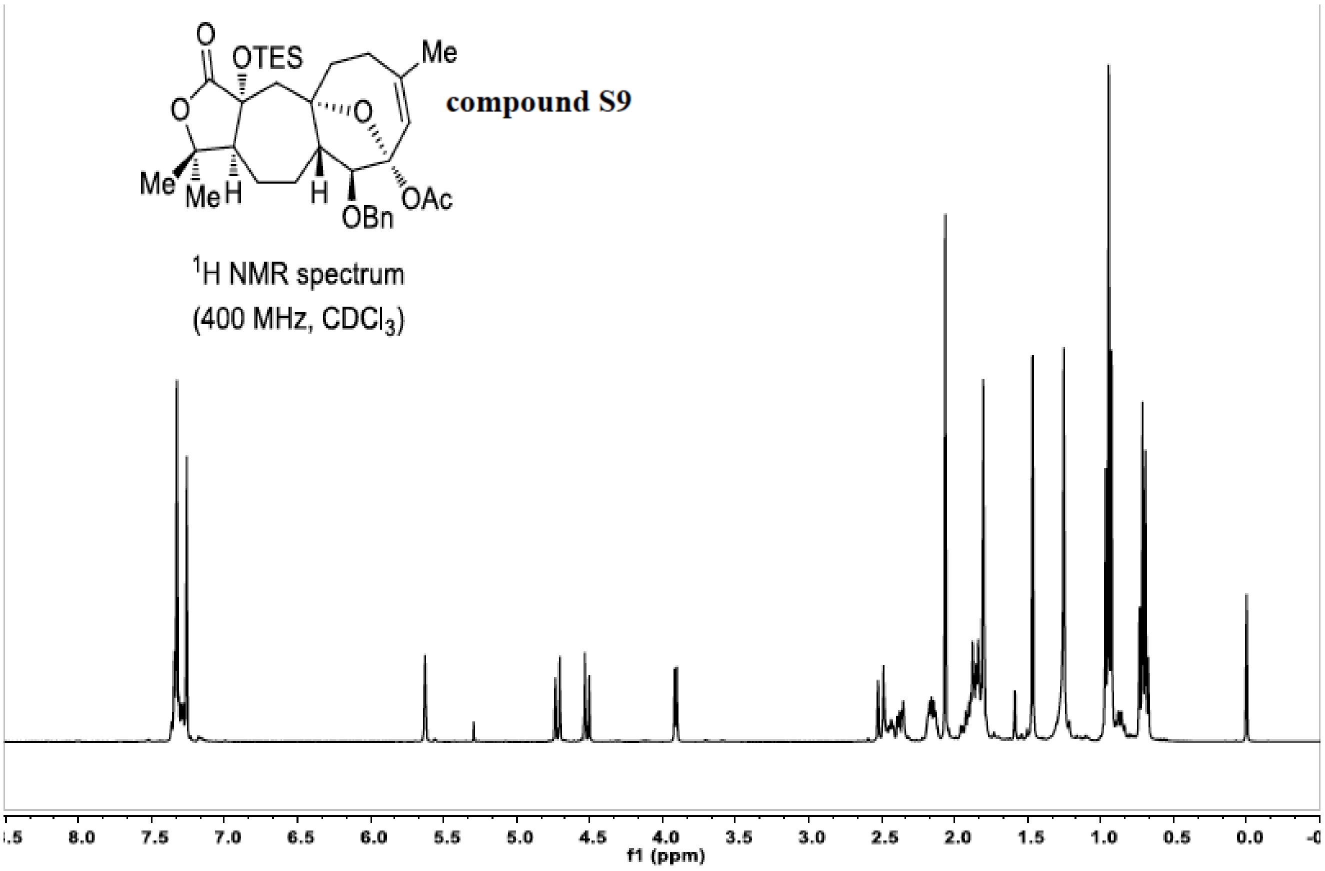

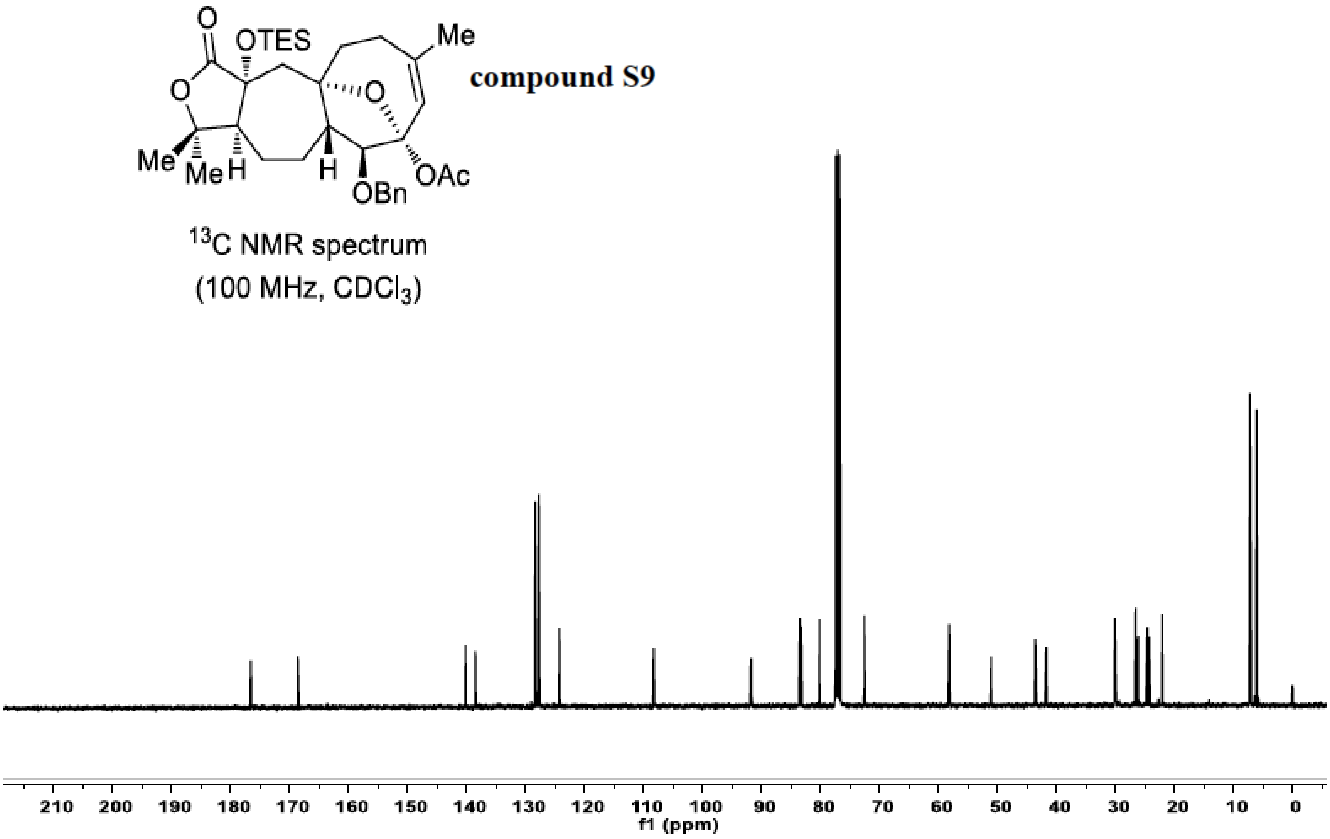

Supplementary Figure 17 | ^{1}H, ^{13}C NMR Spectra for Compound S9

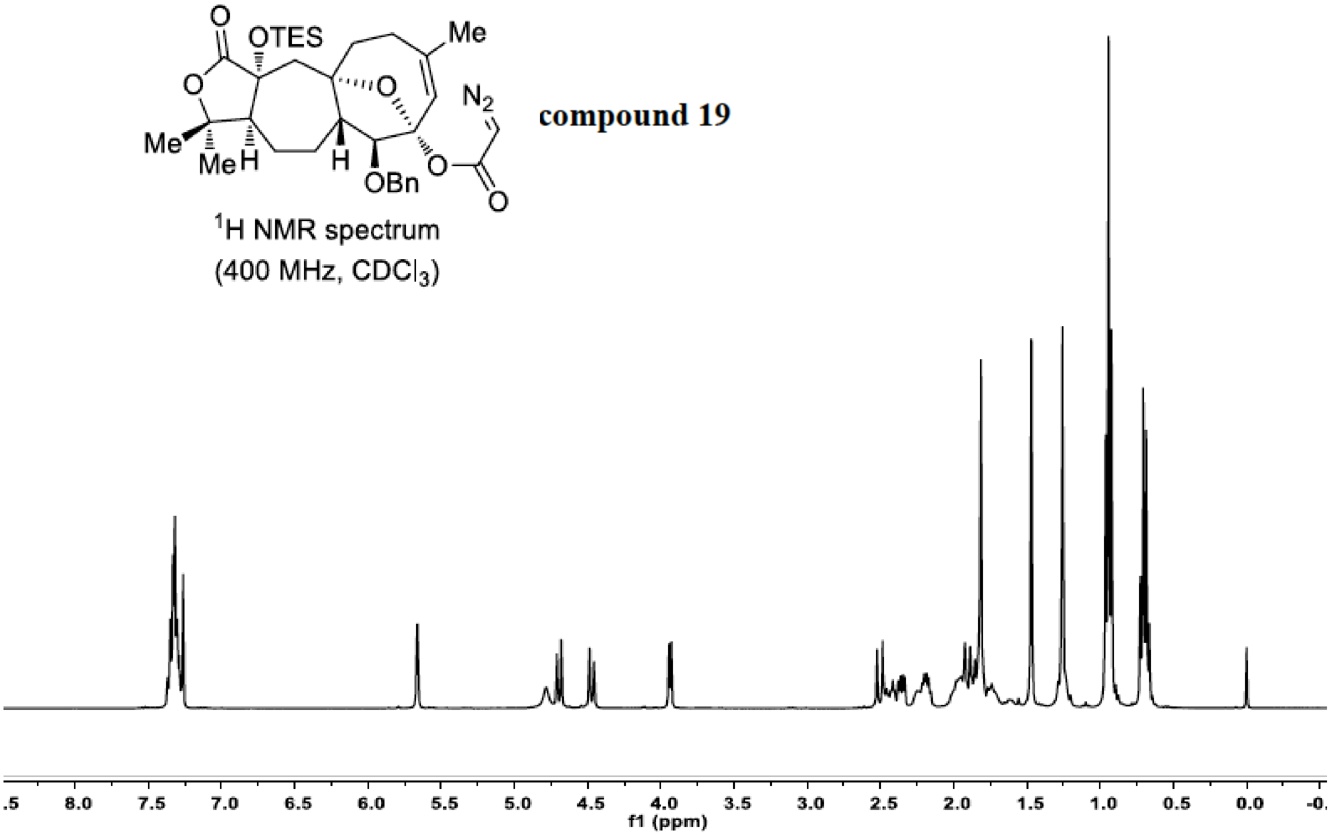

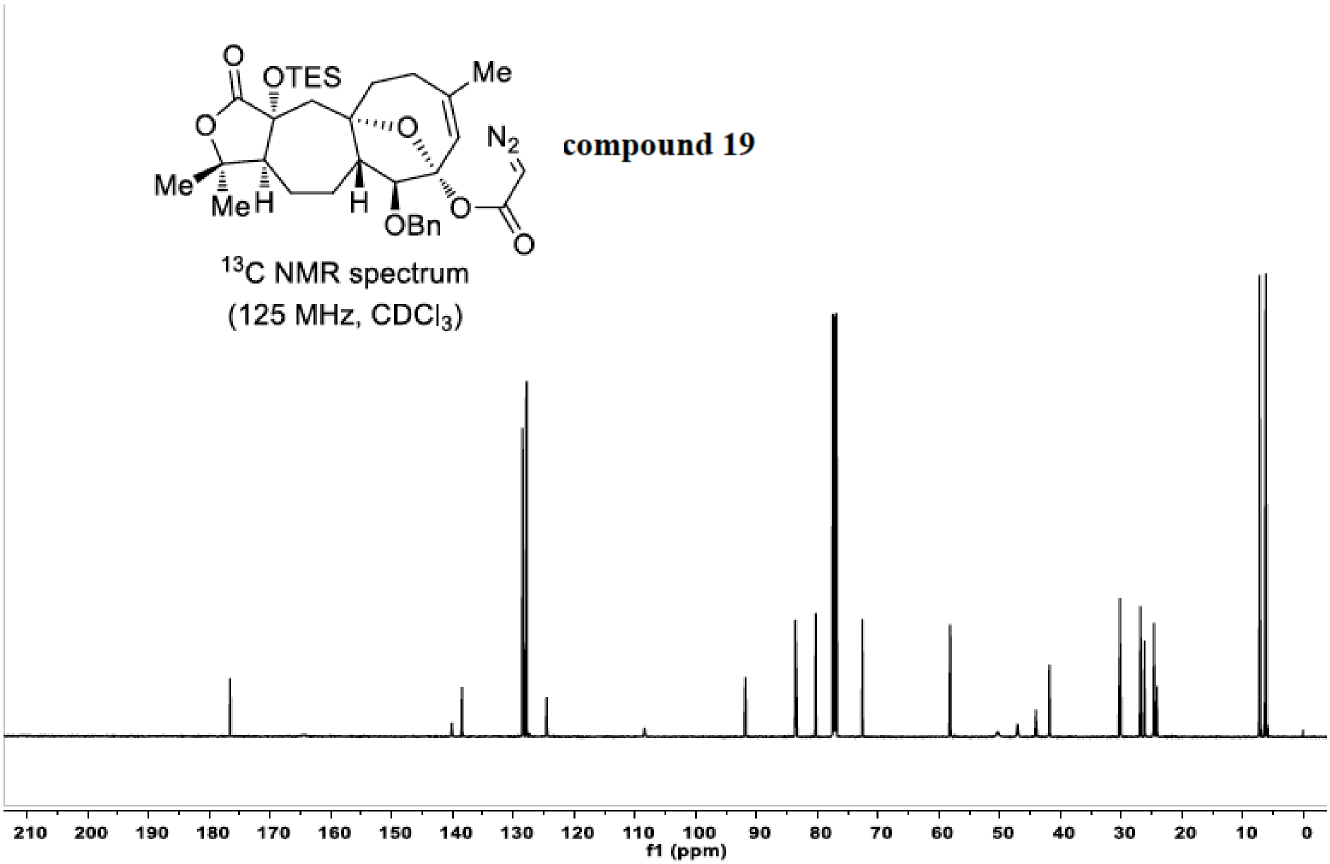

Supplementary Figure 18 | ^{1}H, ^{13}C NMR Spectra for Compound 19

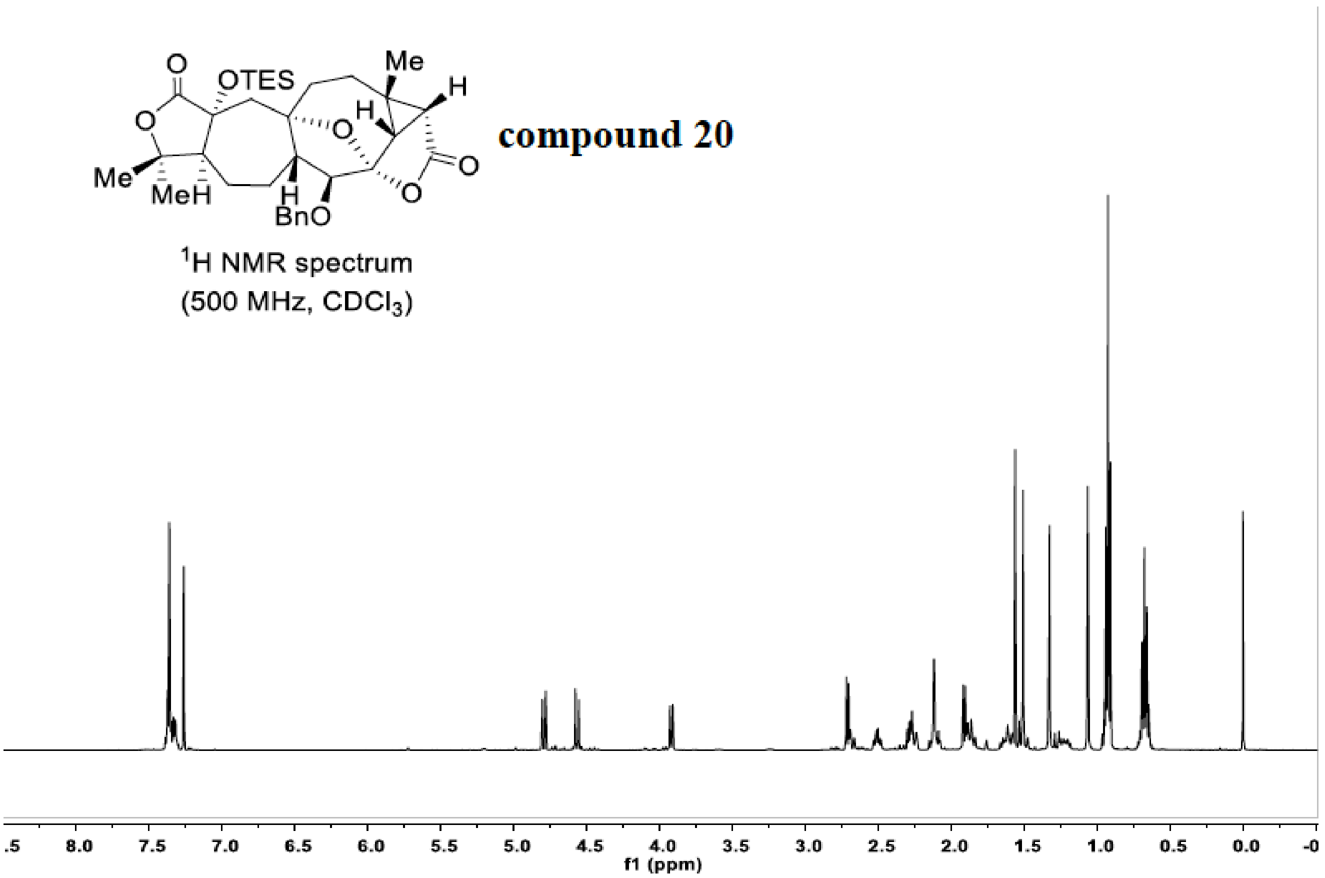
compound 20
^{1}H NMR spectrum
(500 MHz, $CDCl_3$)
f1 (ppm)

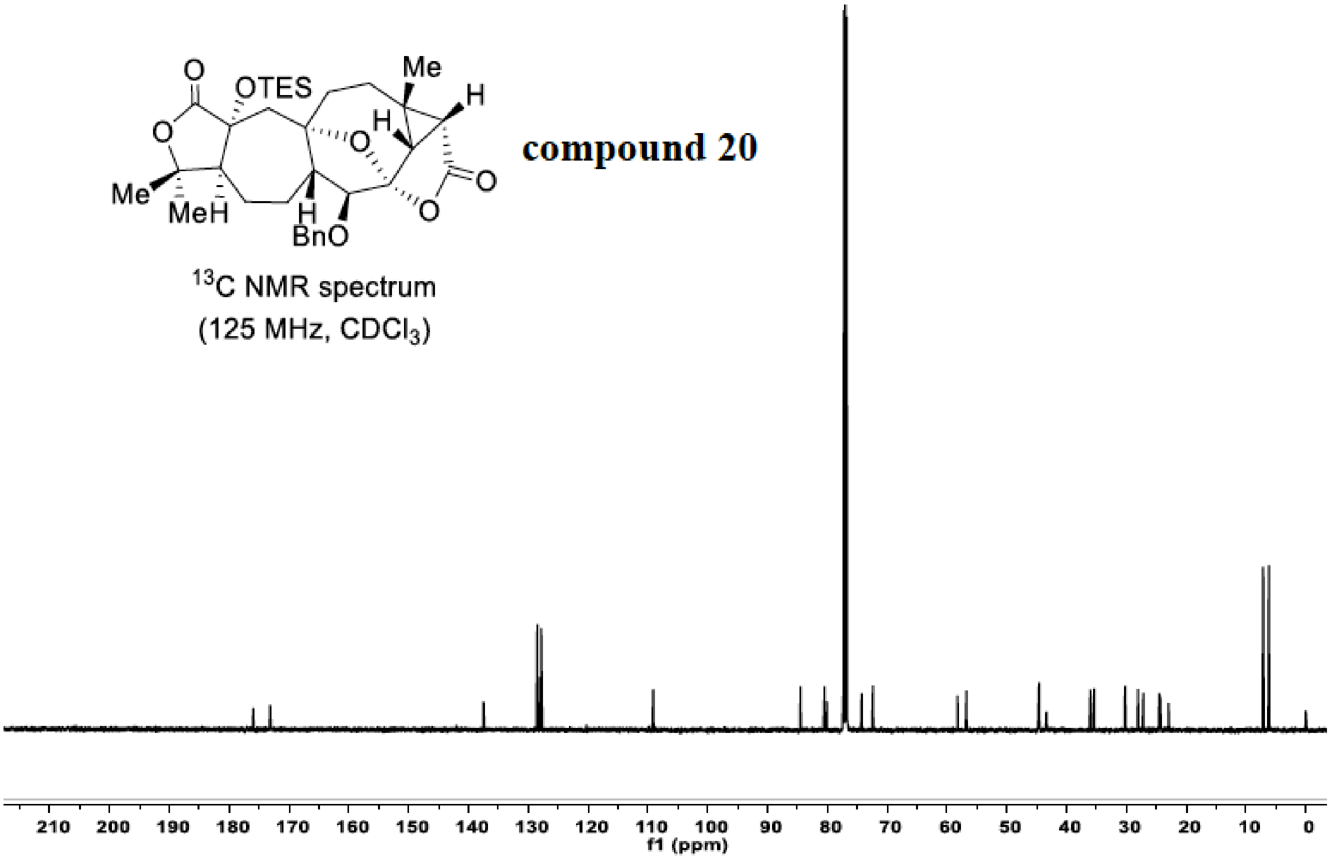
compound 20
^{13}C NMR spectrum
(125 MHz, $CDCl_3$)
f1 (ppm)

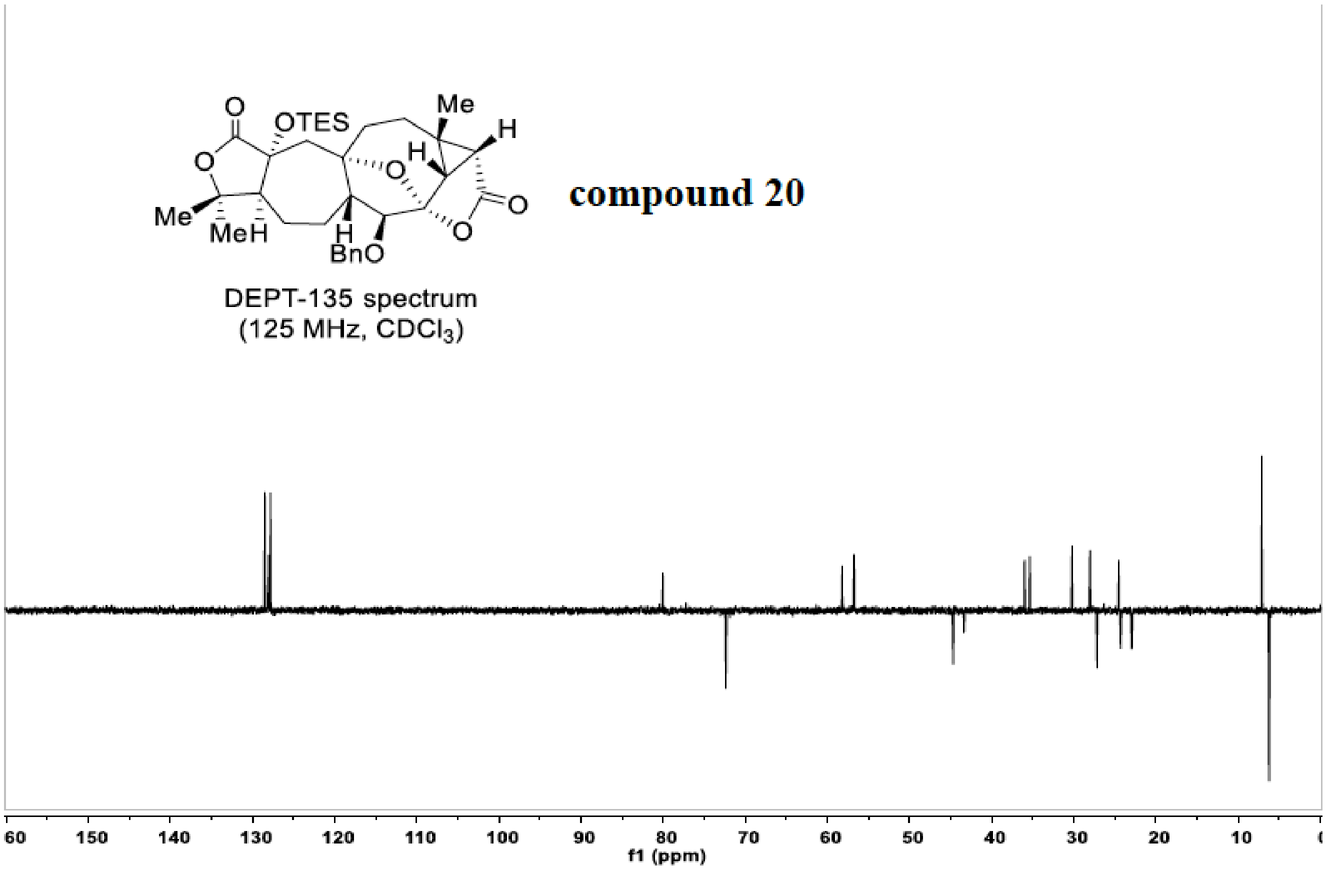
O
OTES
Me
H
O
H
O
O
Me
MeH
H
BnO
O
compound 20
DEPT-135 spectrum
(125 MHz, CDCl3)
f1 (ppm)

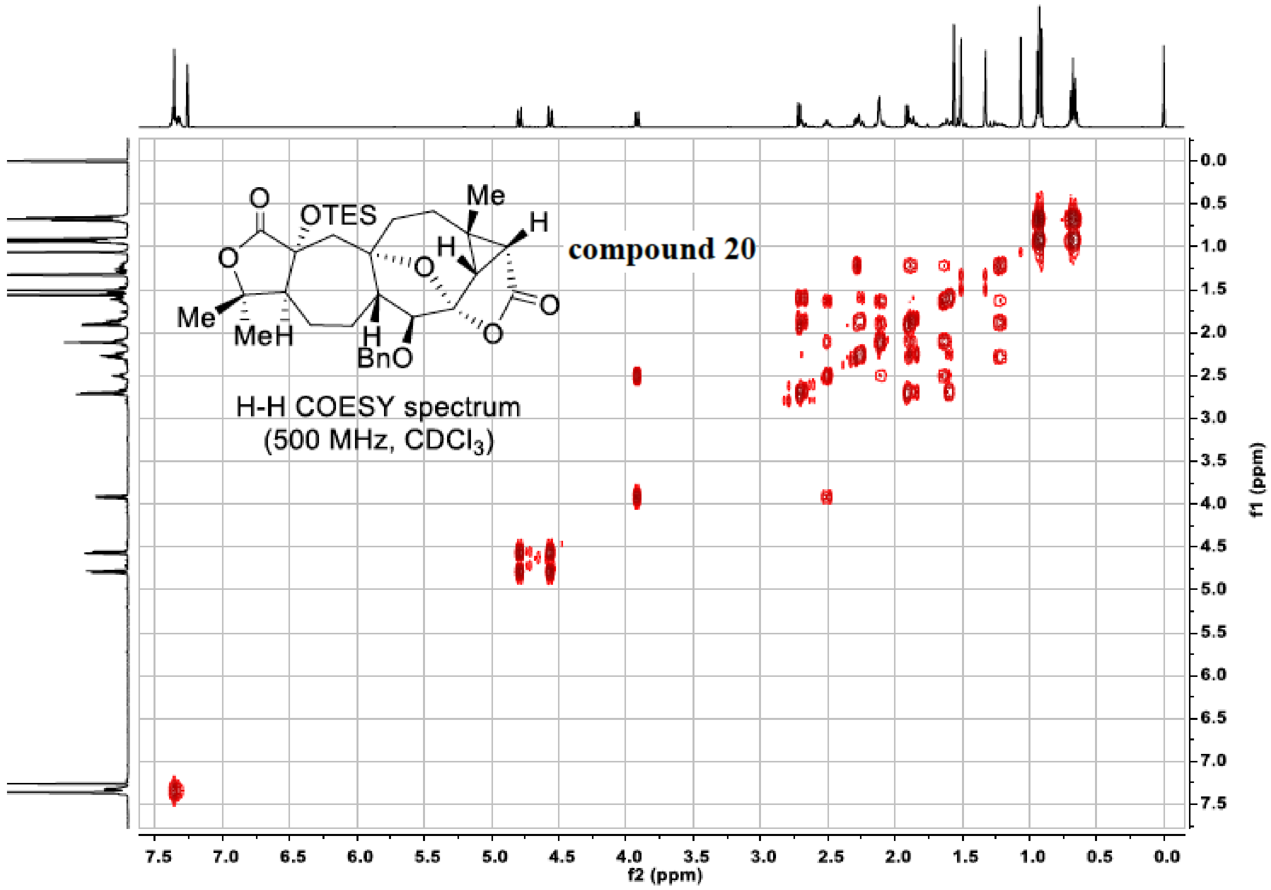
O
OTES
Me
H
O
H
O
O
Me
MeH
H
BnO
O
compound 20
H-H COESY spectrum
(500 MHz, CDCl3)
f2 (ppm)
f1 (ppm)

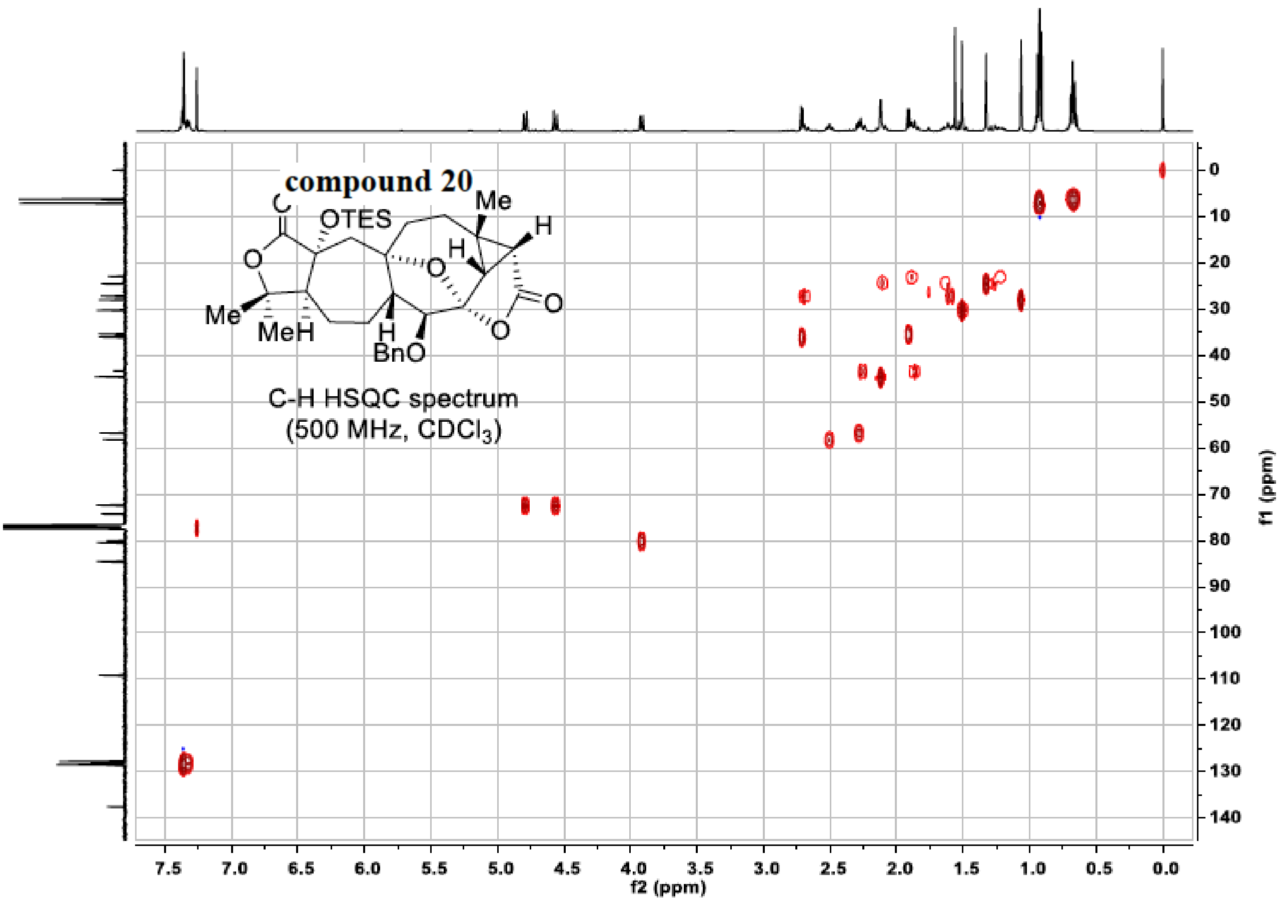
compound 20
C-H HSQC spectrum
(500 MHz, CDCl3)
f2 (ppm)
f1 (ppm)

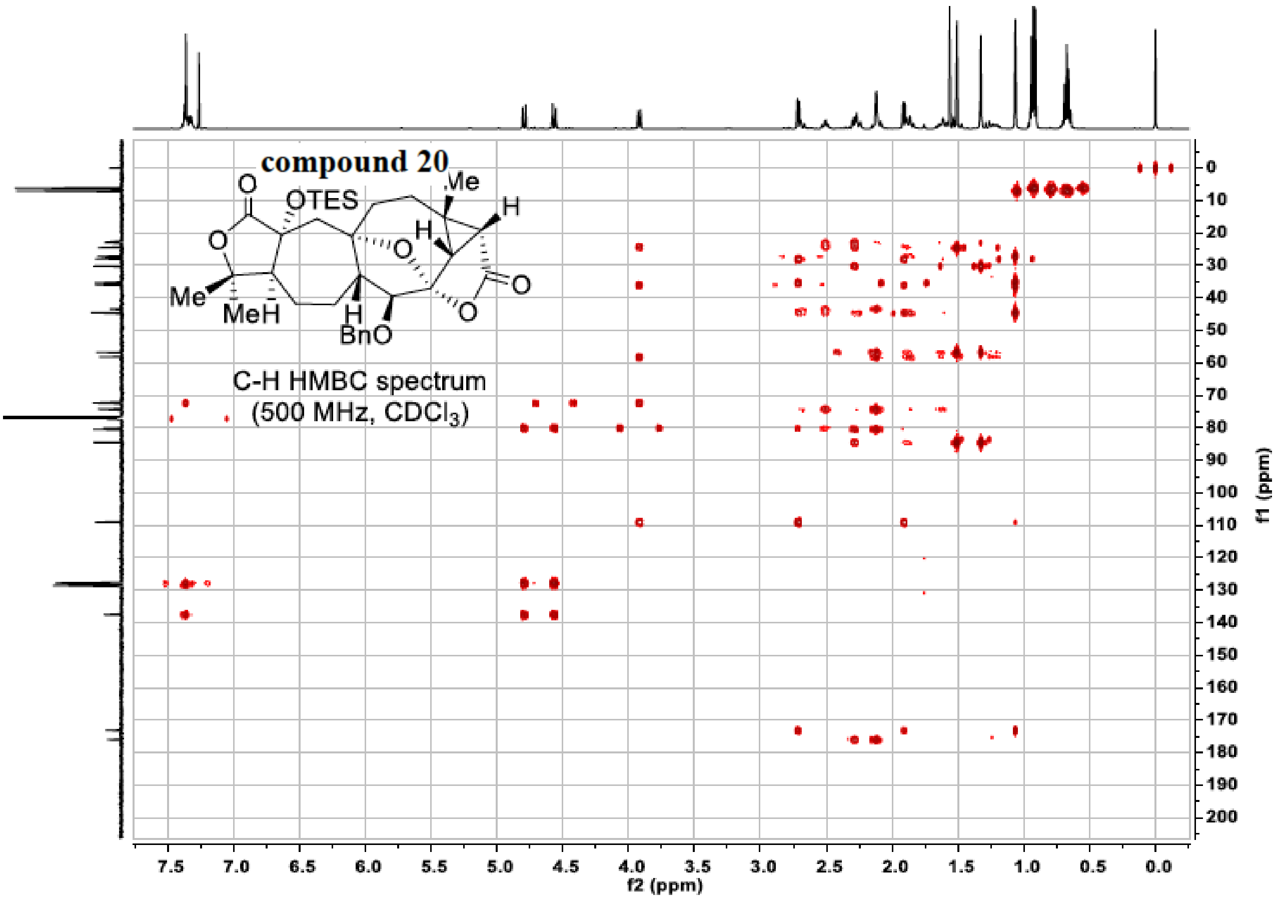
compound 20
C-H HMBC spectrum
(500 MHz, CDCl3)
f2 (ppm)
f1 (ppm)

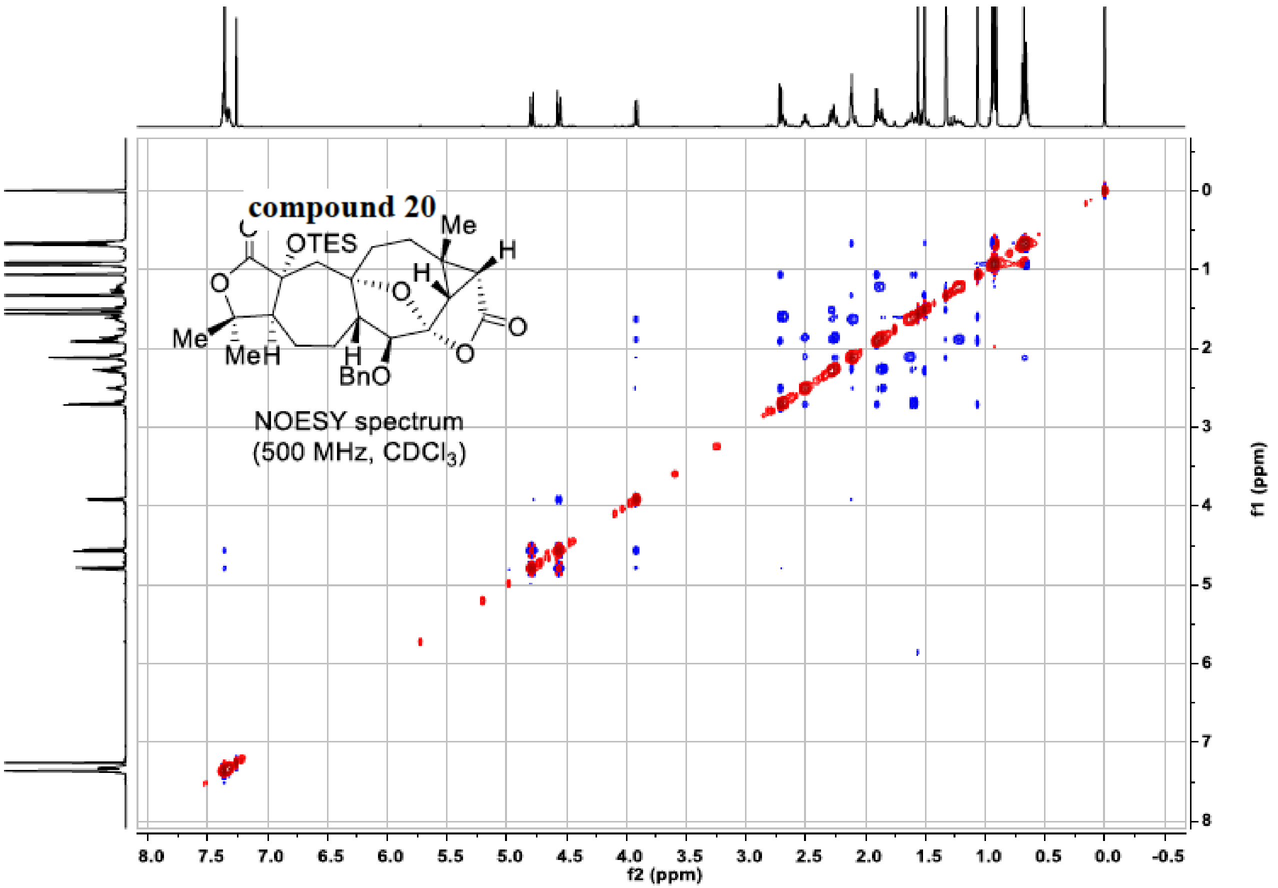

Supplementary Figure 19 | ^{1}H, ^{13}C, DEPT135, COESY, HSQC, HMBC, NOESY Spectra for Compound 20

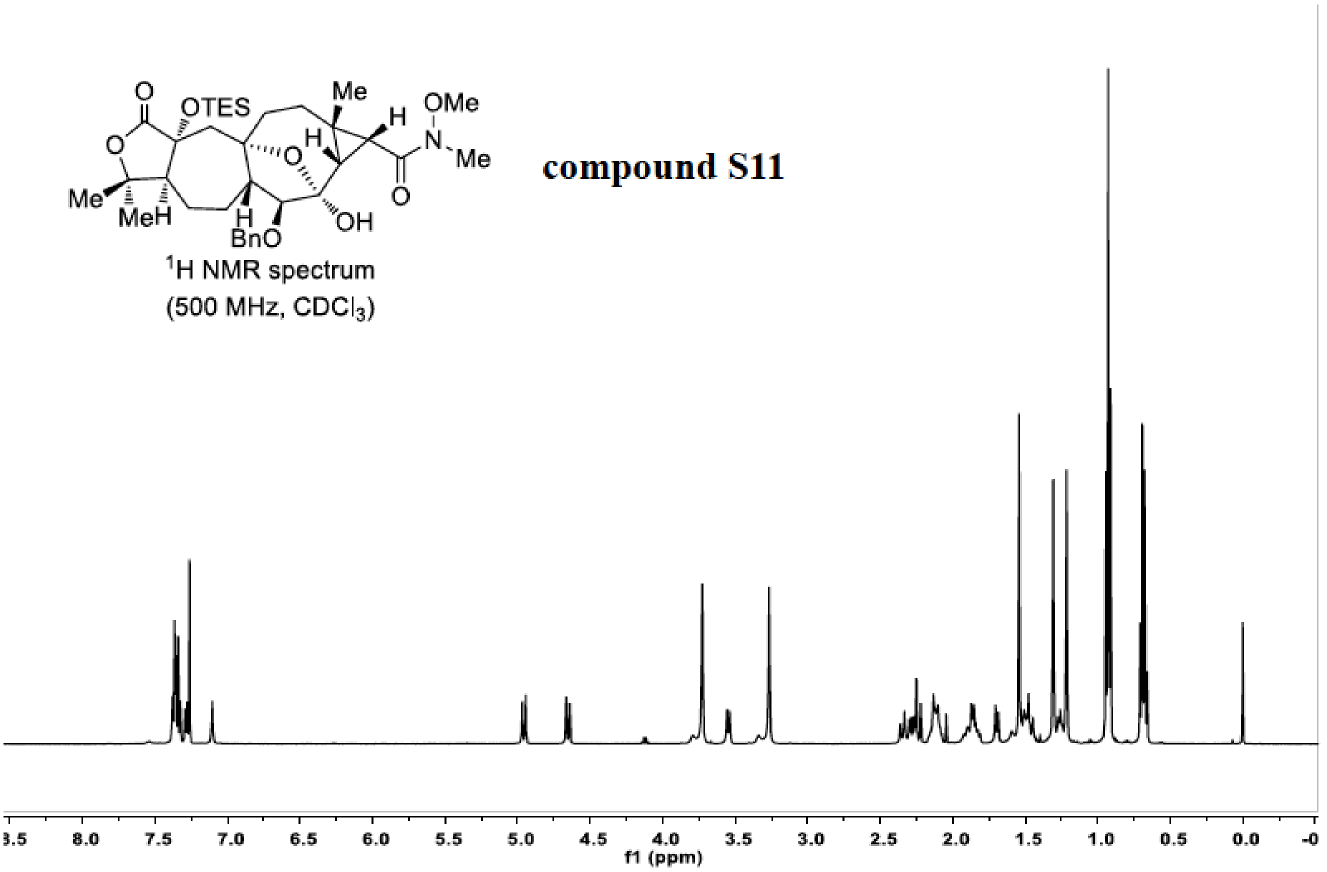

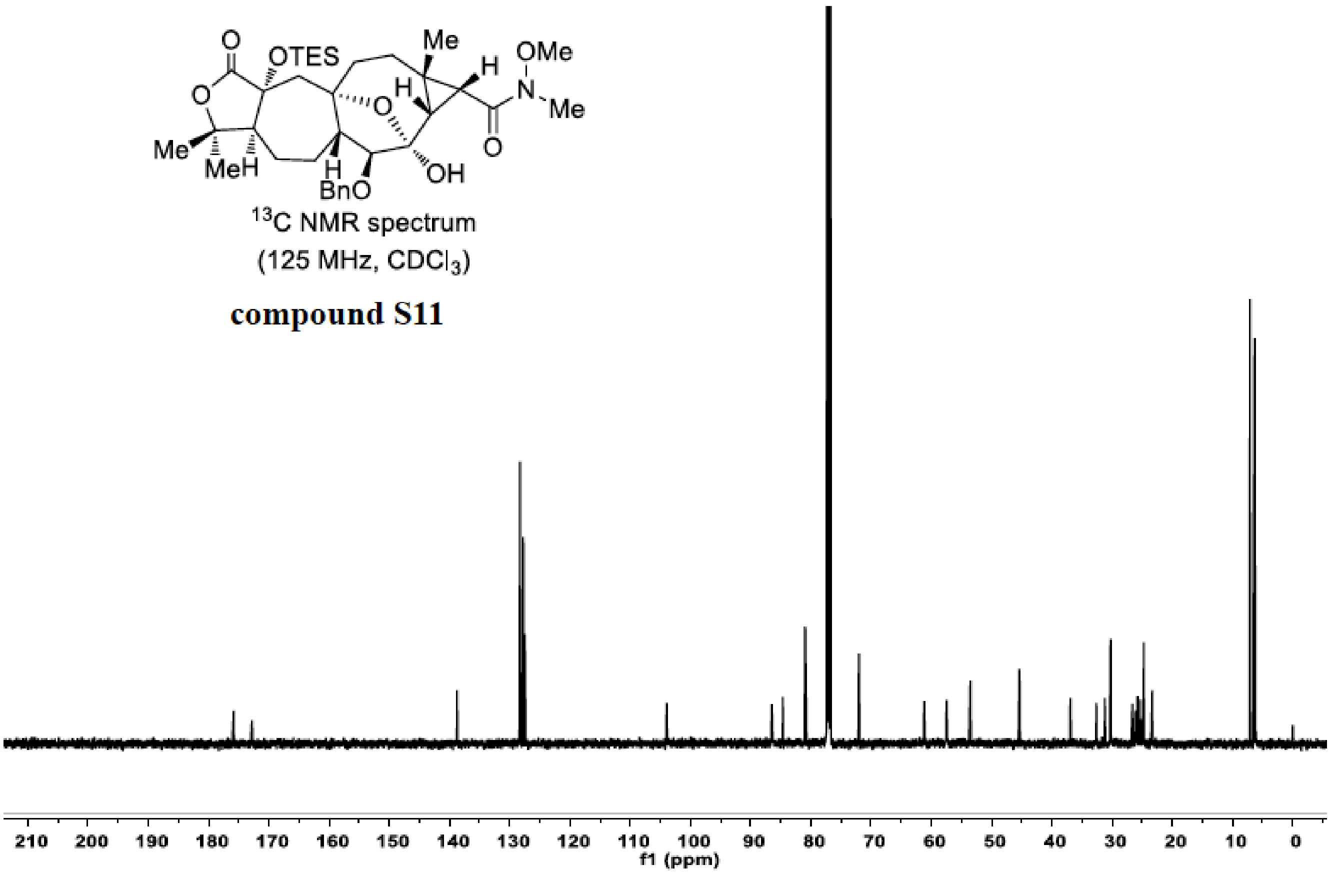

Supplementary Figure 20 | ^{1}H, ^{13}C NMR Spectra for Compound S11

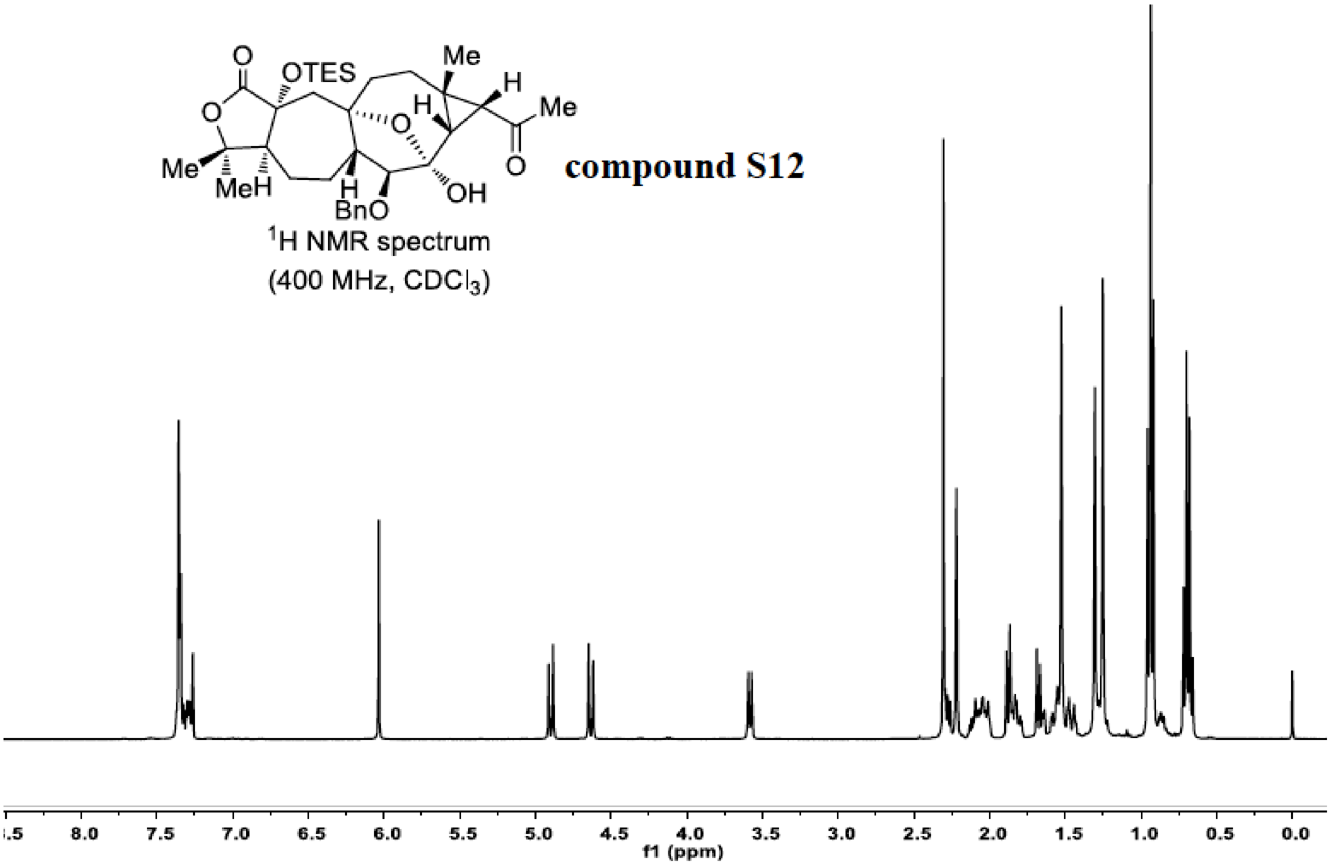

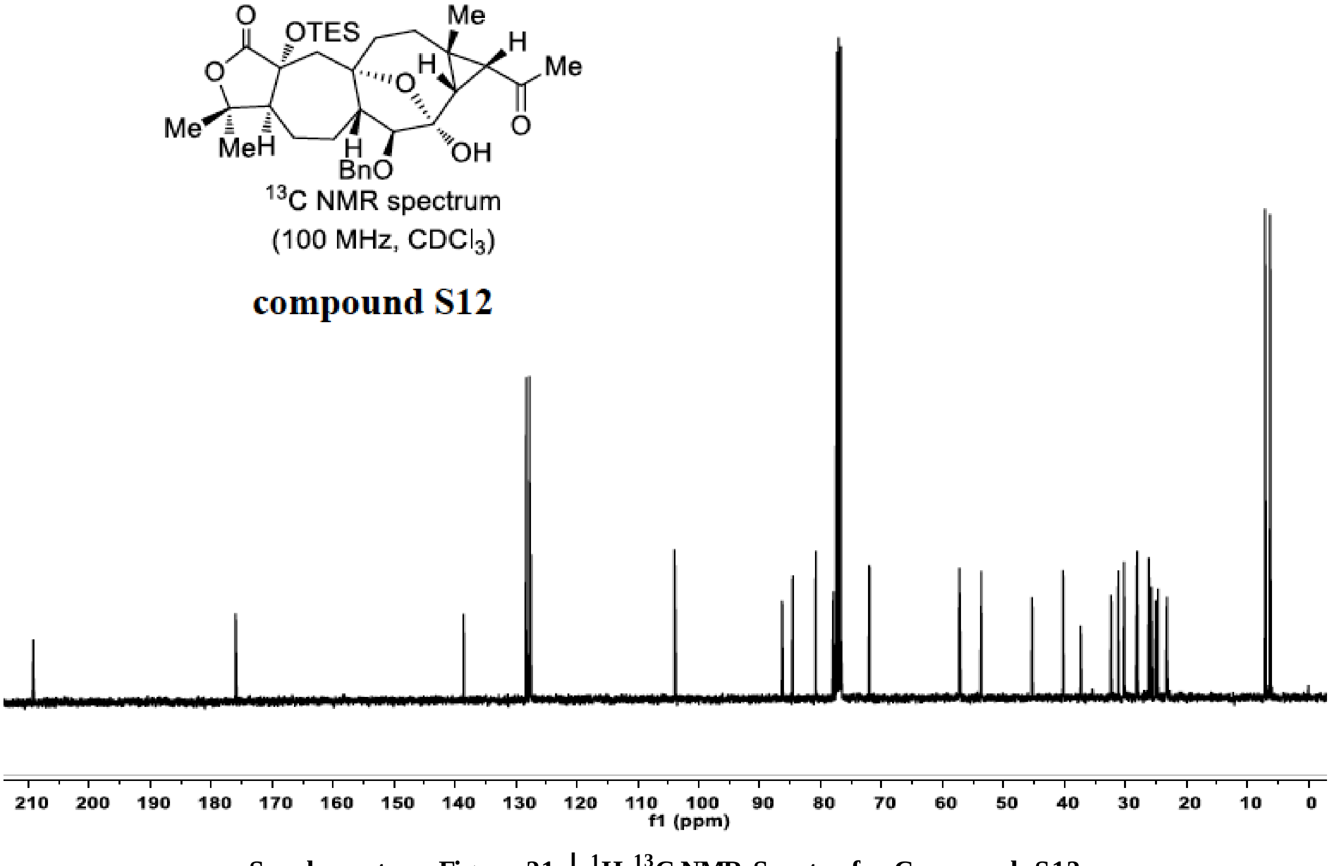

Supplementary Figure 21 | ^{1}H, ^{13}C NMR Spectra for Compound S12

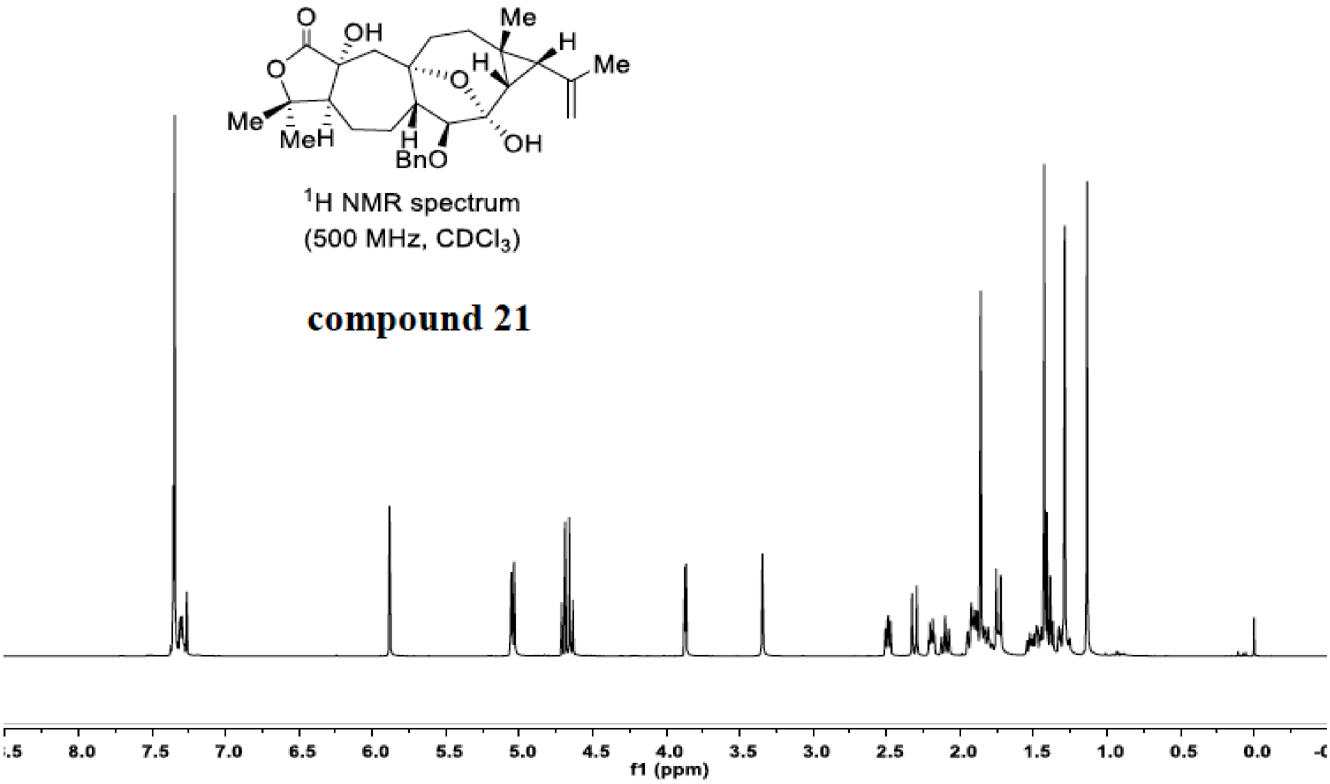

^{1}H NMR spectrum
(500 MHz, $CDCl_3$)
compound 21
f1 (ppm)

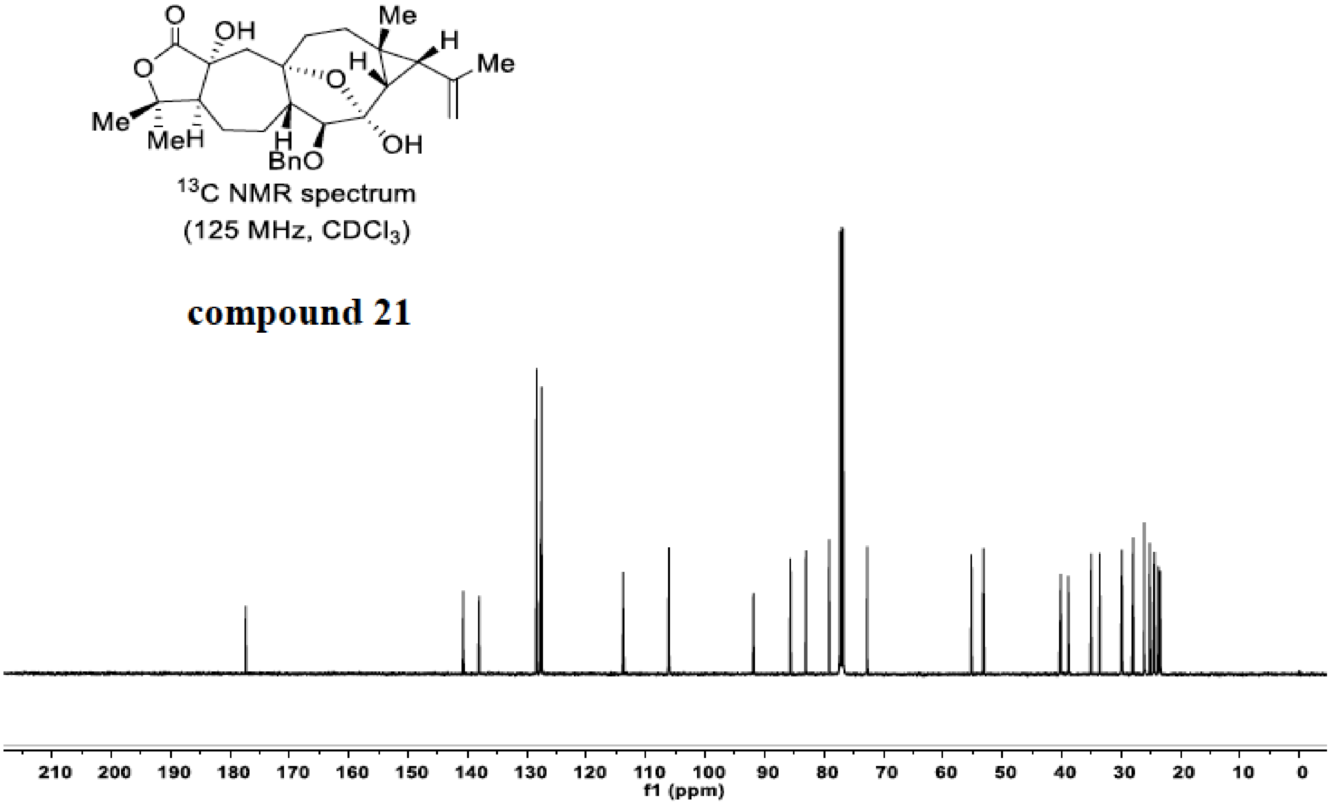

^{13}C NMR spectrum
(125 MHz, $CDCl_3$)
compound 21
f1 (ppm)

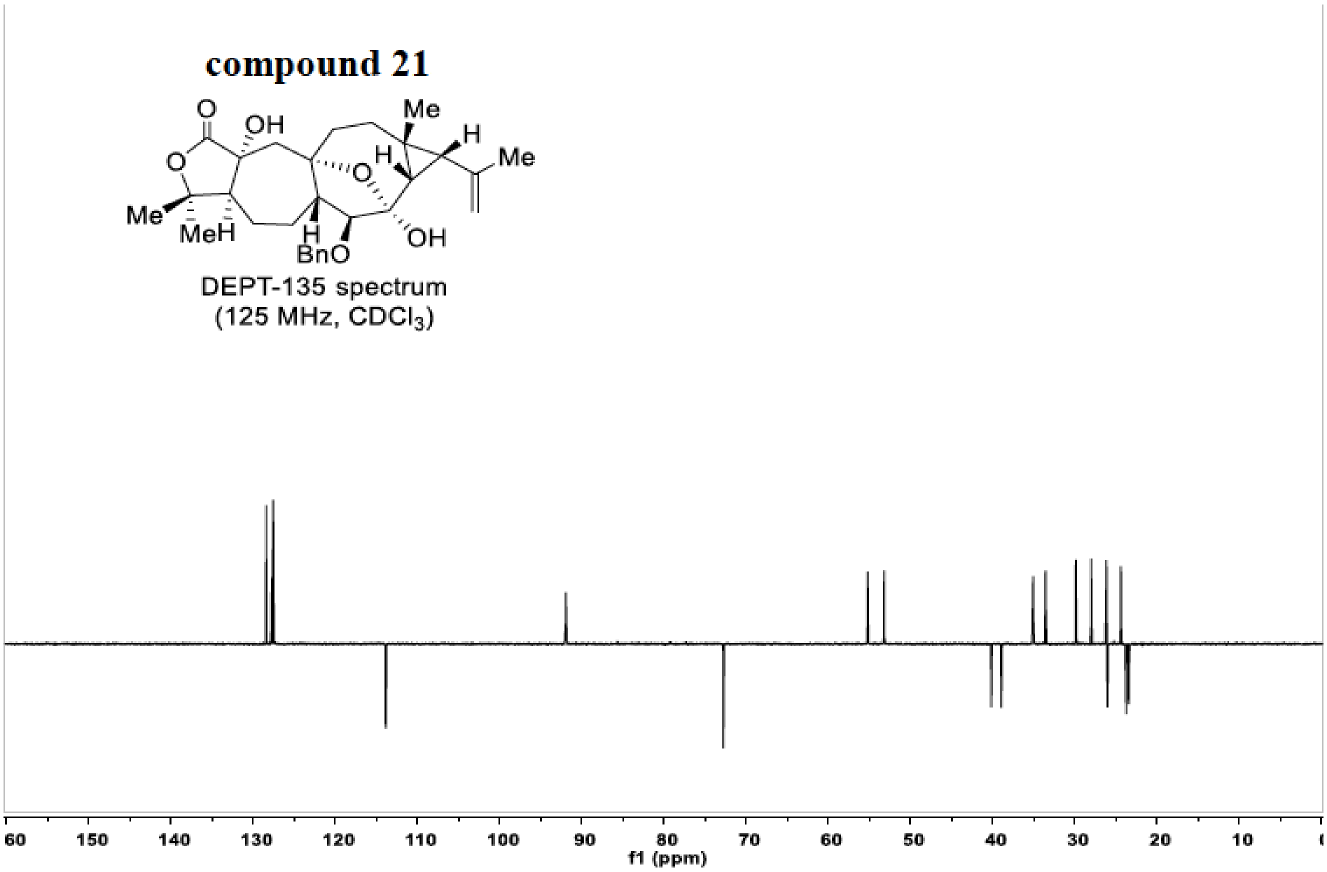
compound 21
DEPT-135 spectrum
(125 MHz, CDCl3)
f1 (ppm)

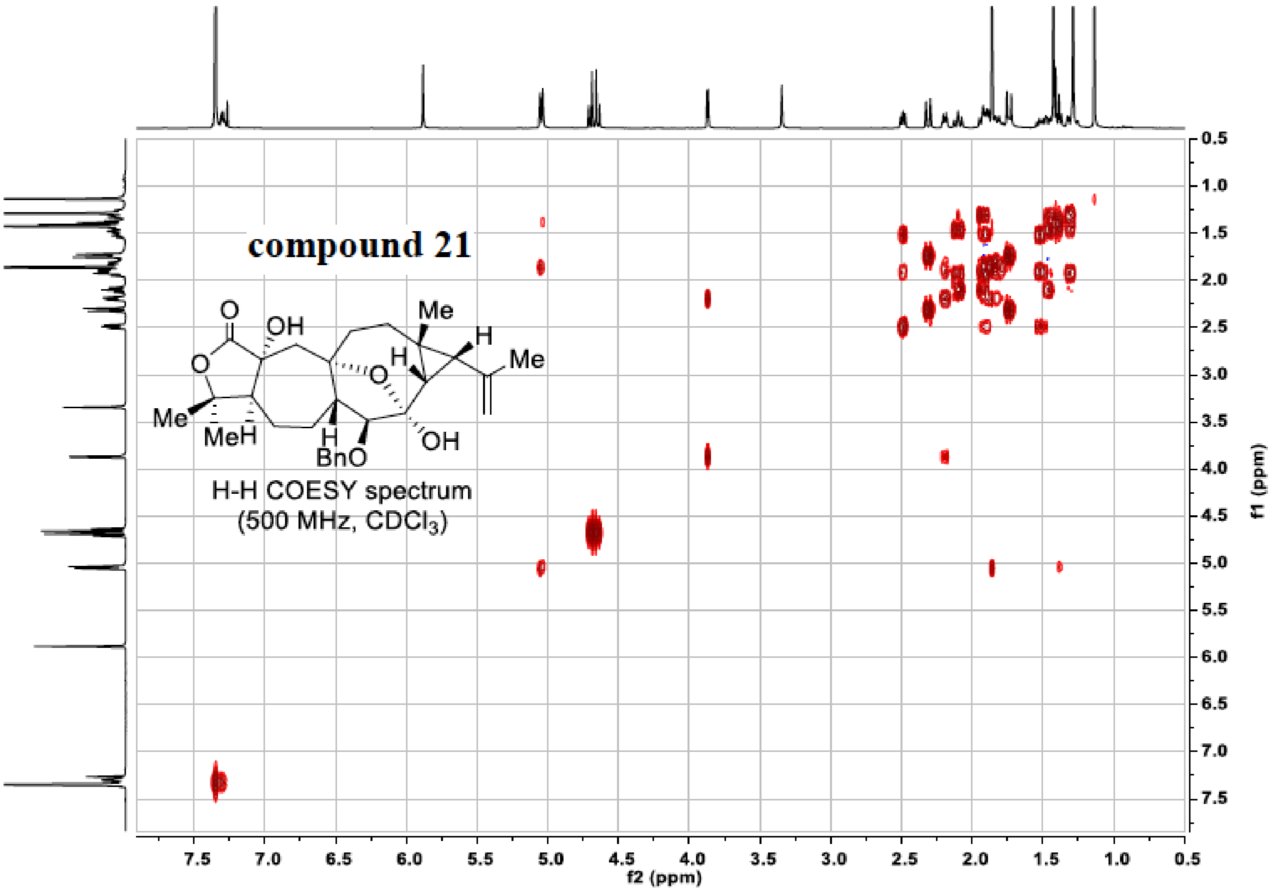
compound 21
H-H COESY spectrum
(500 MHz, CDCl3)
f2 (ppm)
f1 (ppm)

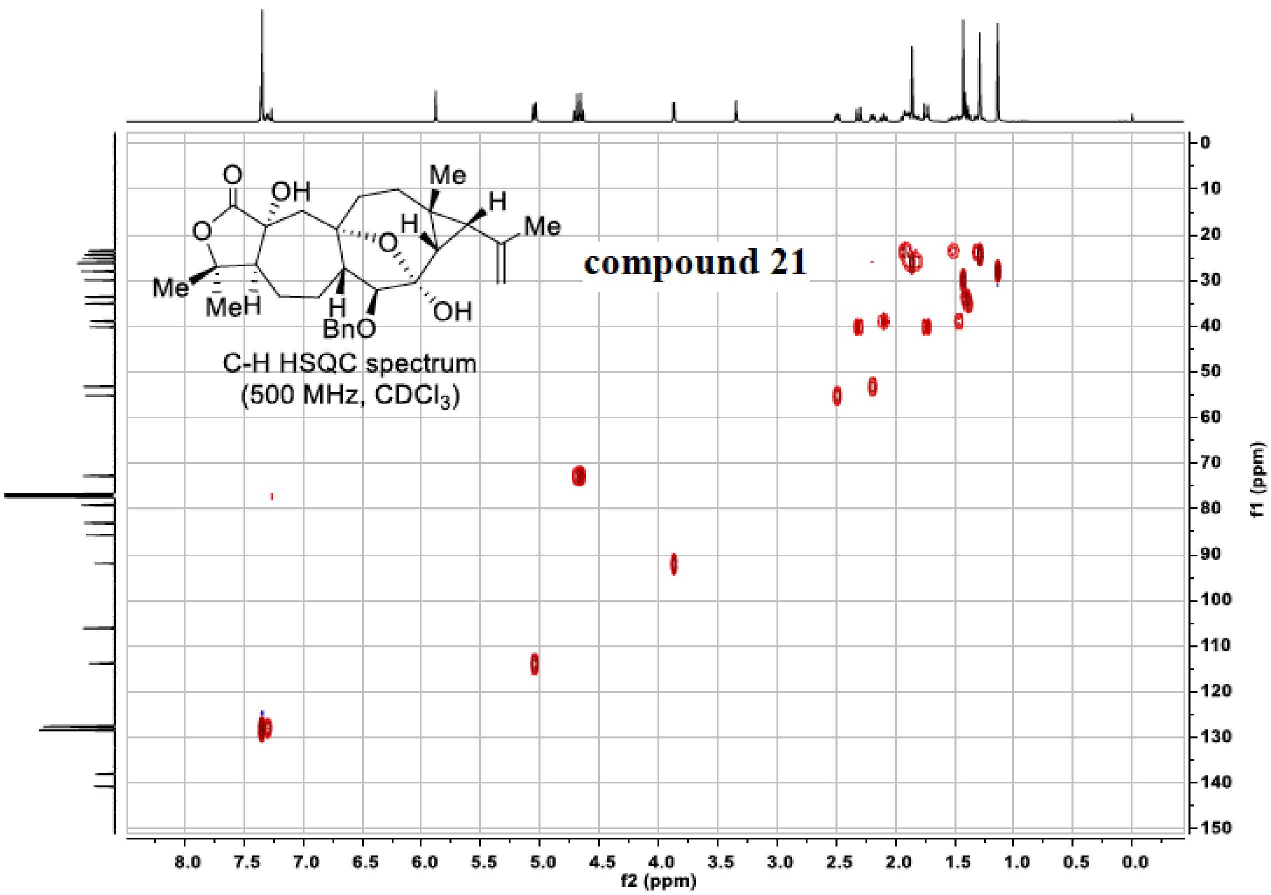
compound 21
C-H HSQC spectrum
(500 MHz, CDCl3)
f2 (ppm)
f1 (ppm)

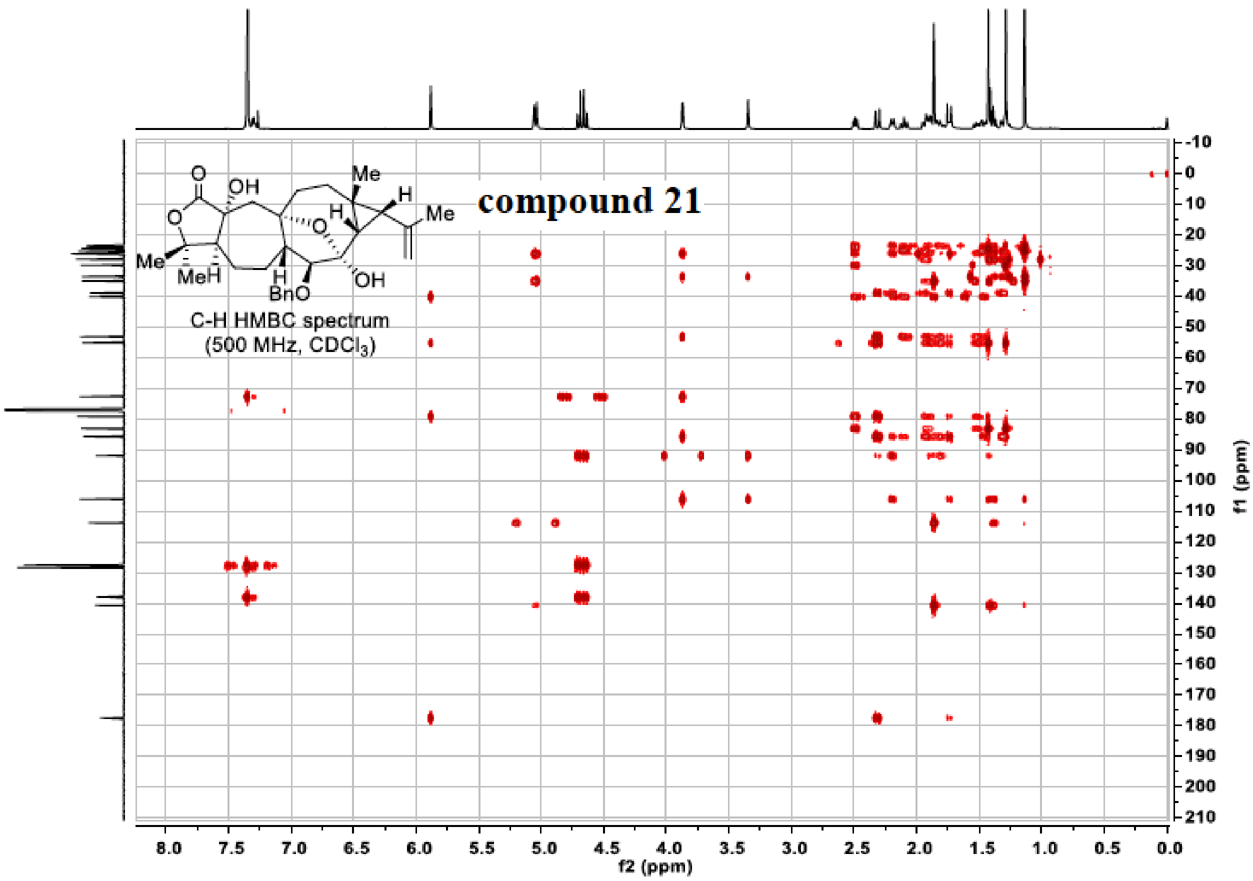
compound 21
C-H HMBC spectrum
(500 MHz, CDCl3)
f2 (ppm)
f1 (ppm)

compound 21

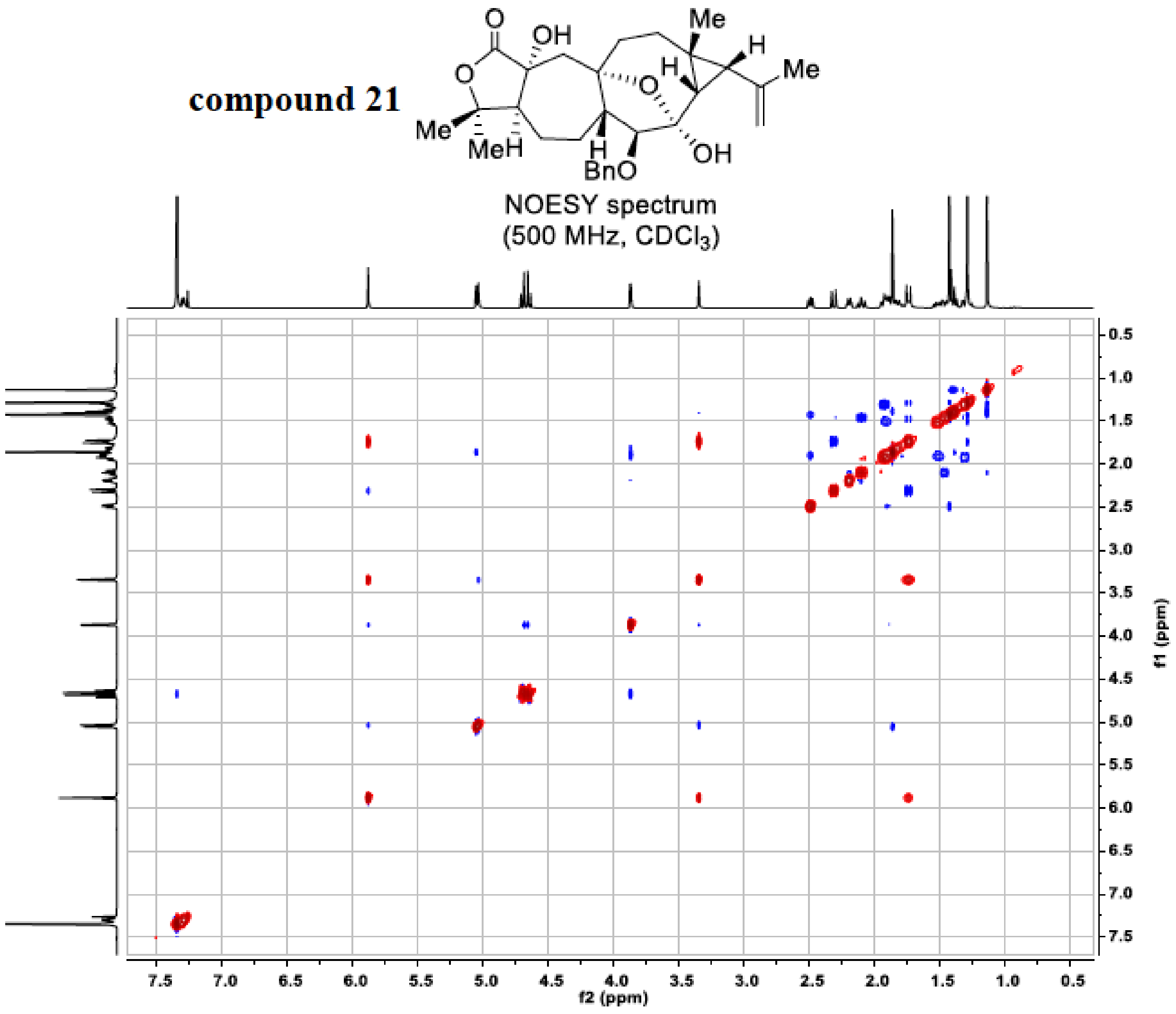

Supplementary Figure 22 | ^{1}H, ^{13}C, DEPT135, COESY, HSQC, HMBC, NOESY Spectra for Compound 21

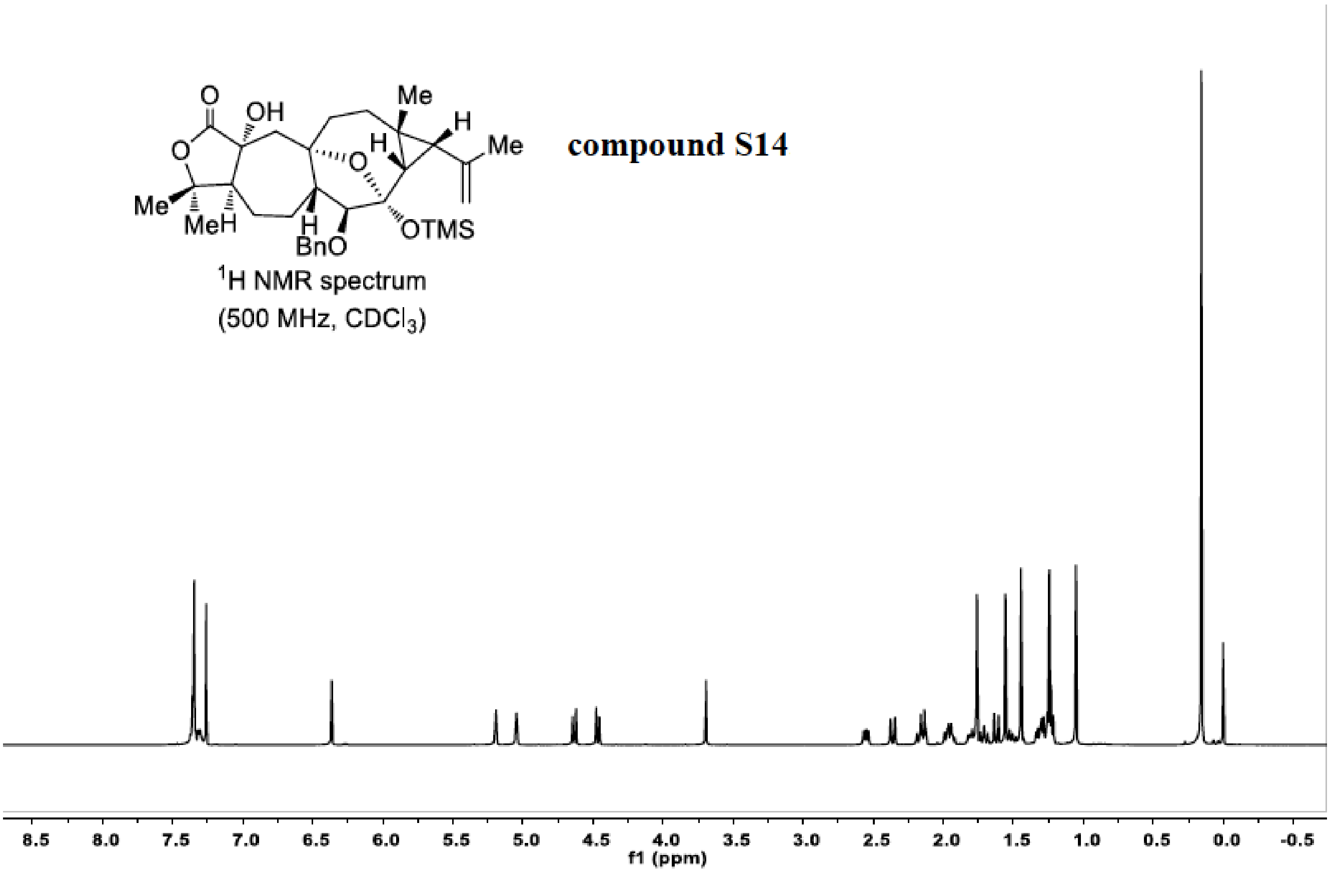

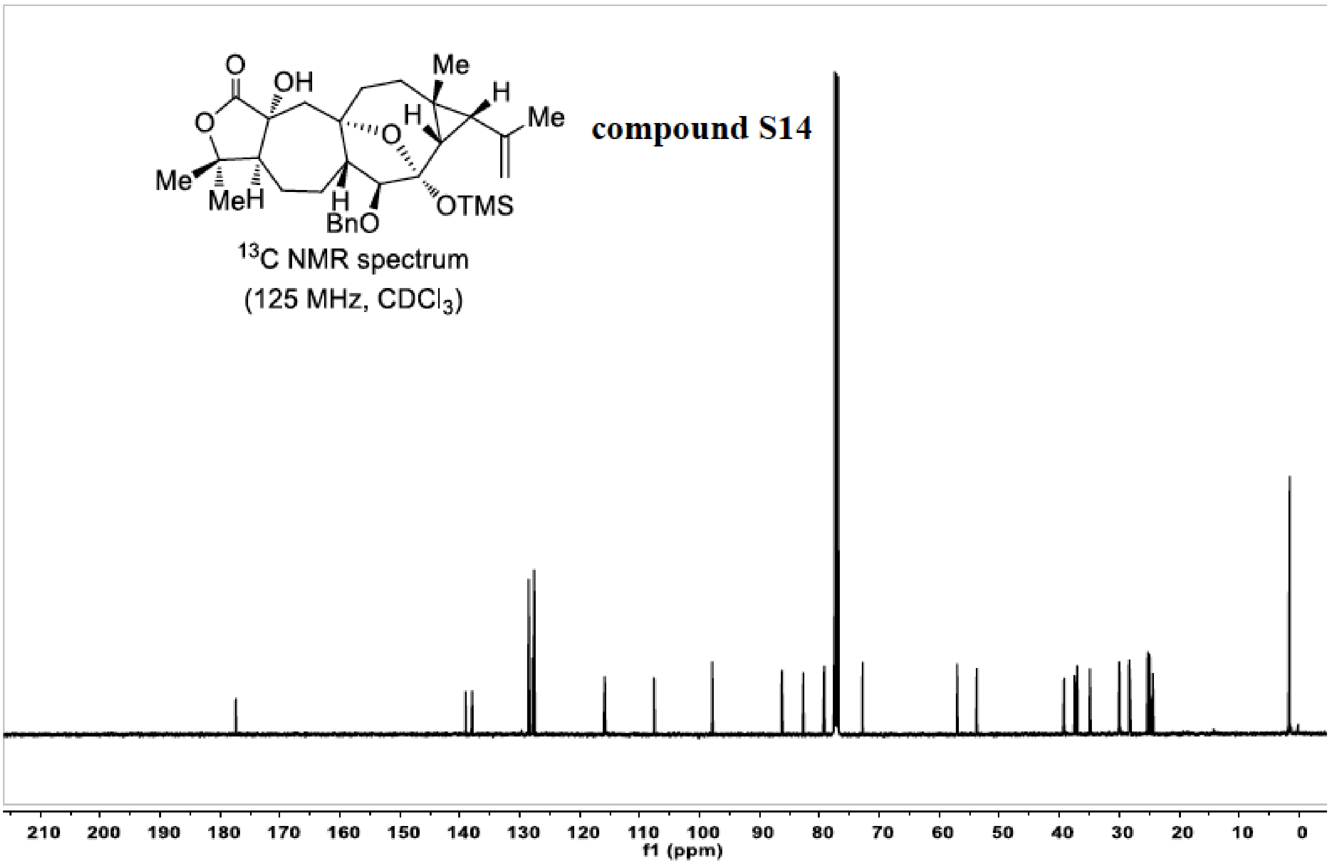

Supplementary Figure 23 | ^{1}H, ^{13}C NMR Spectra for Compound S14

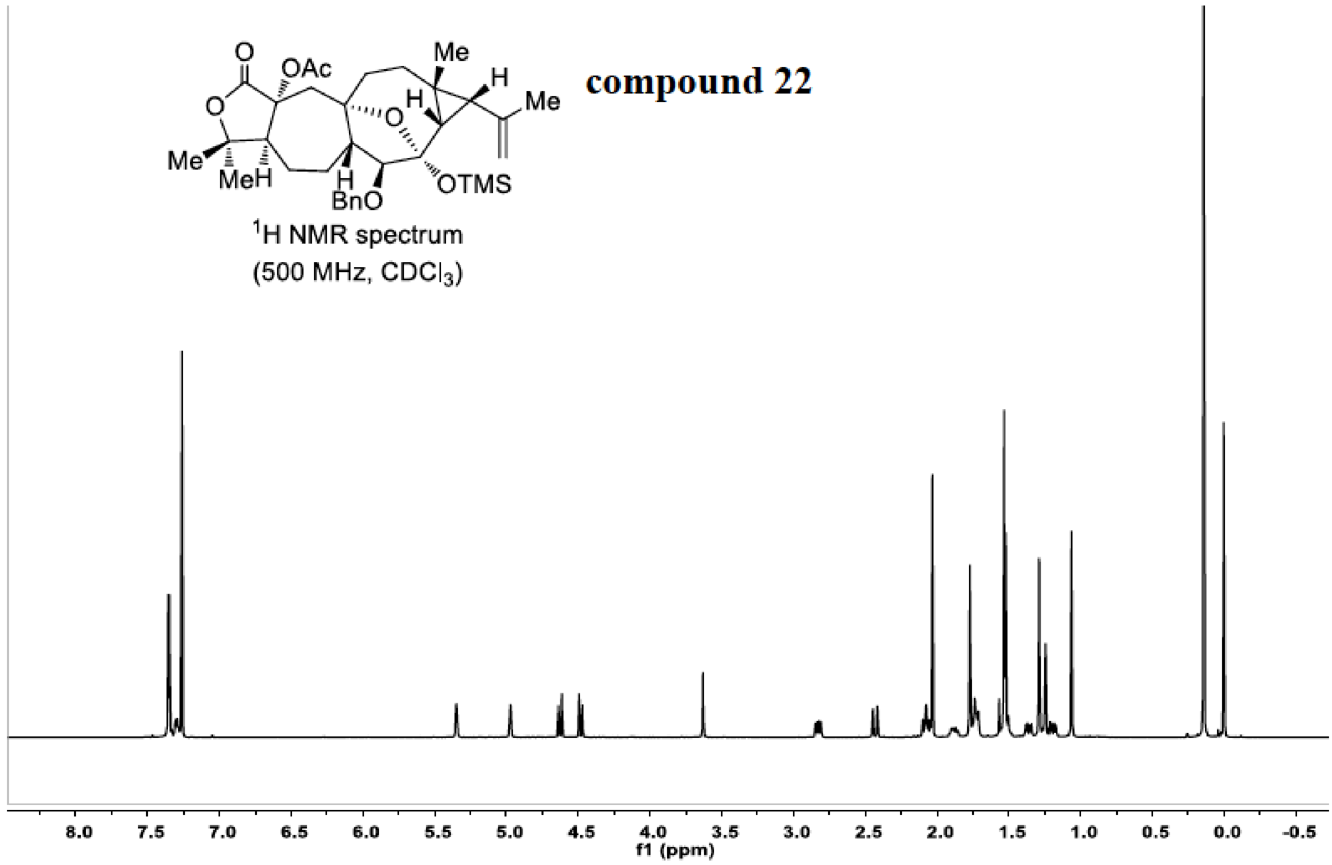

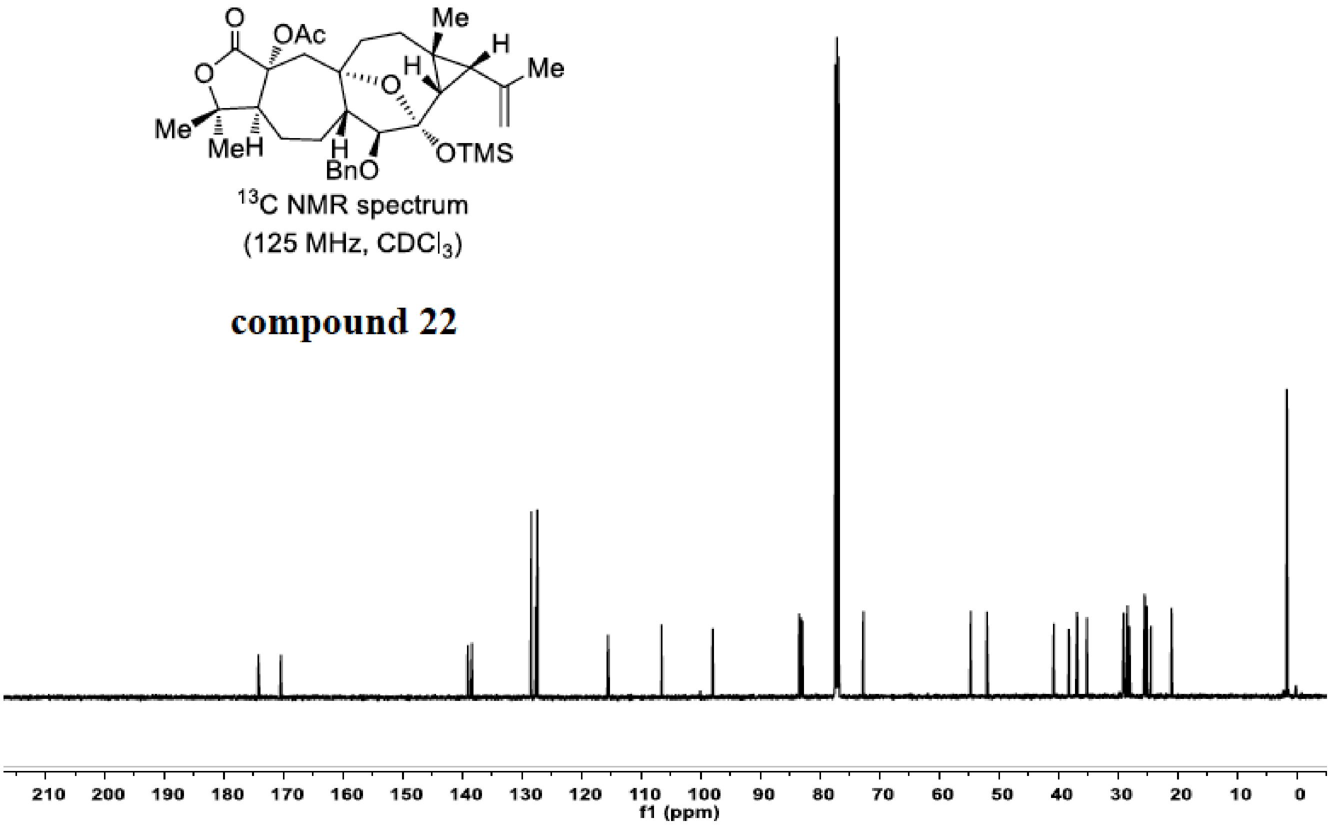

Supplementary Figure 24 | ^{1}H, ^{13}C NMR Spectra for Compound 22

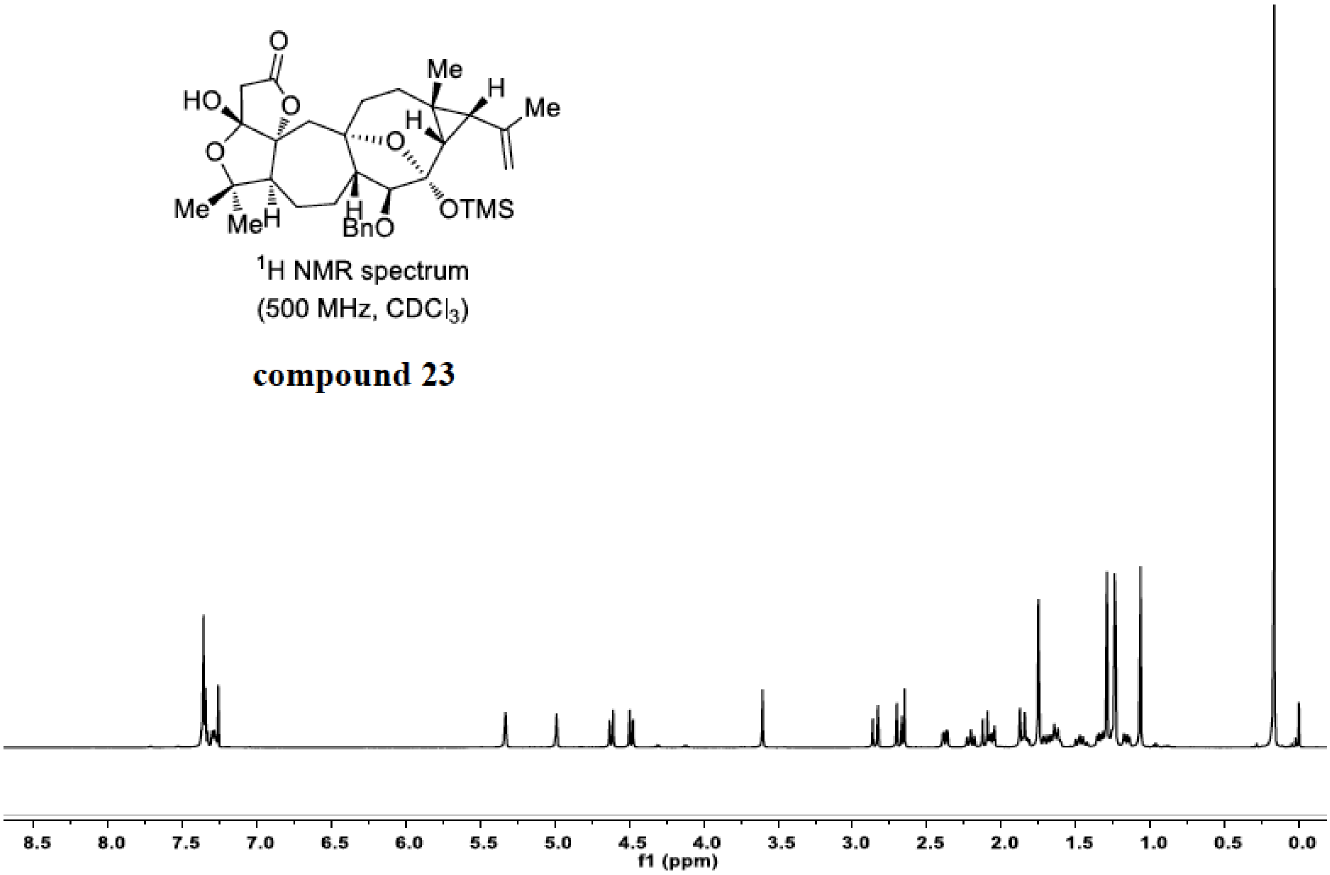

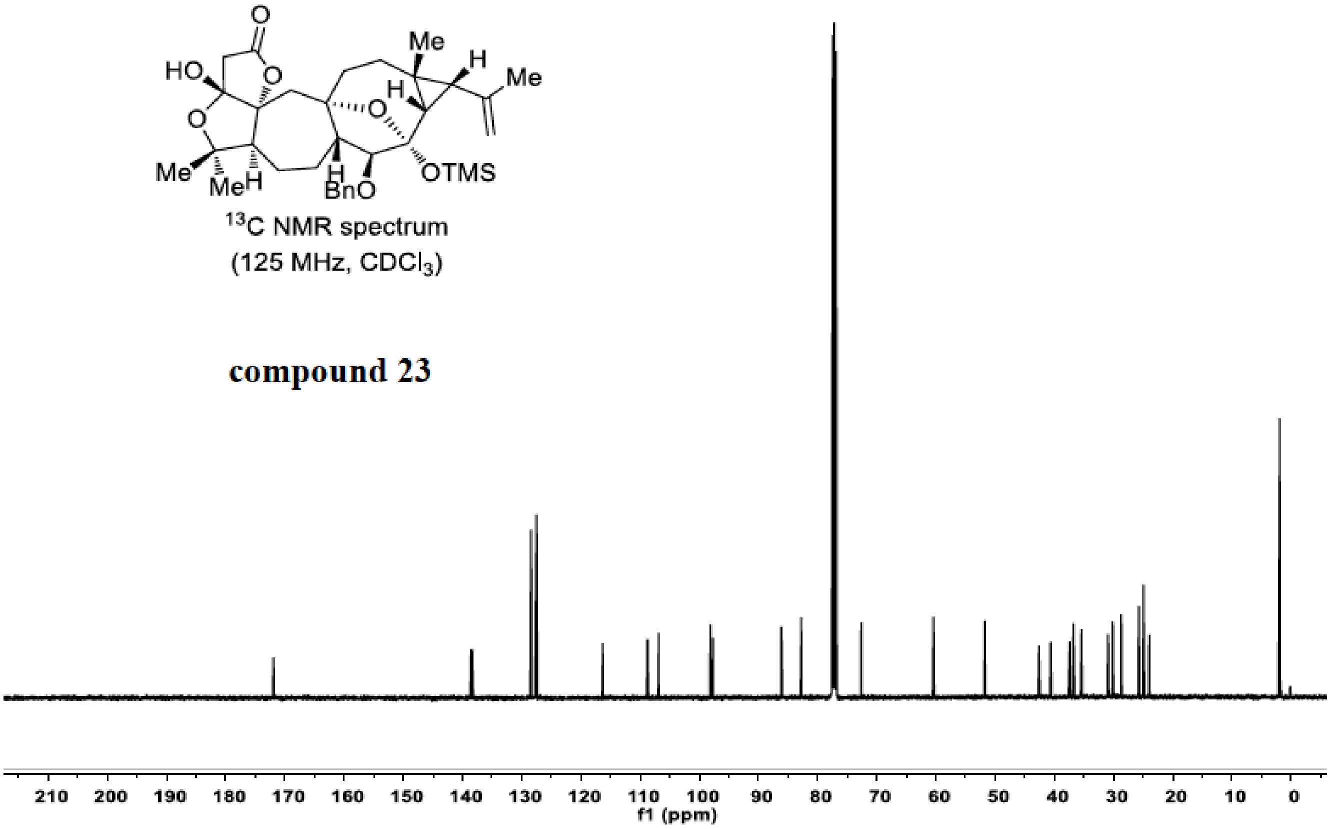

Supplementary Figure 25 | ^{1}H, ^{13}C NMR Spectra for Compound 23

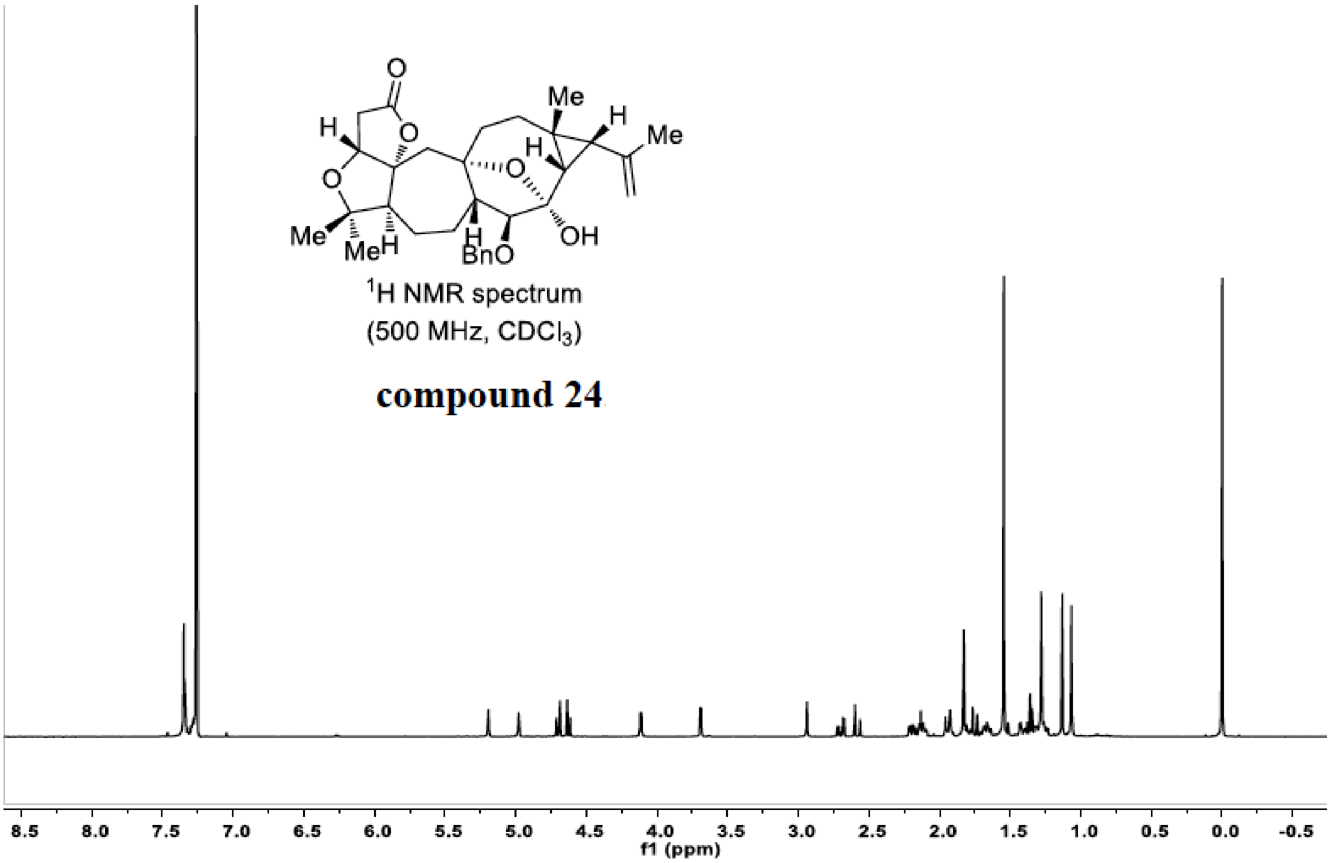

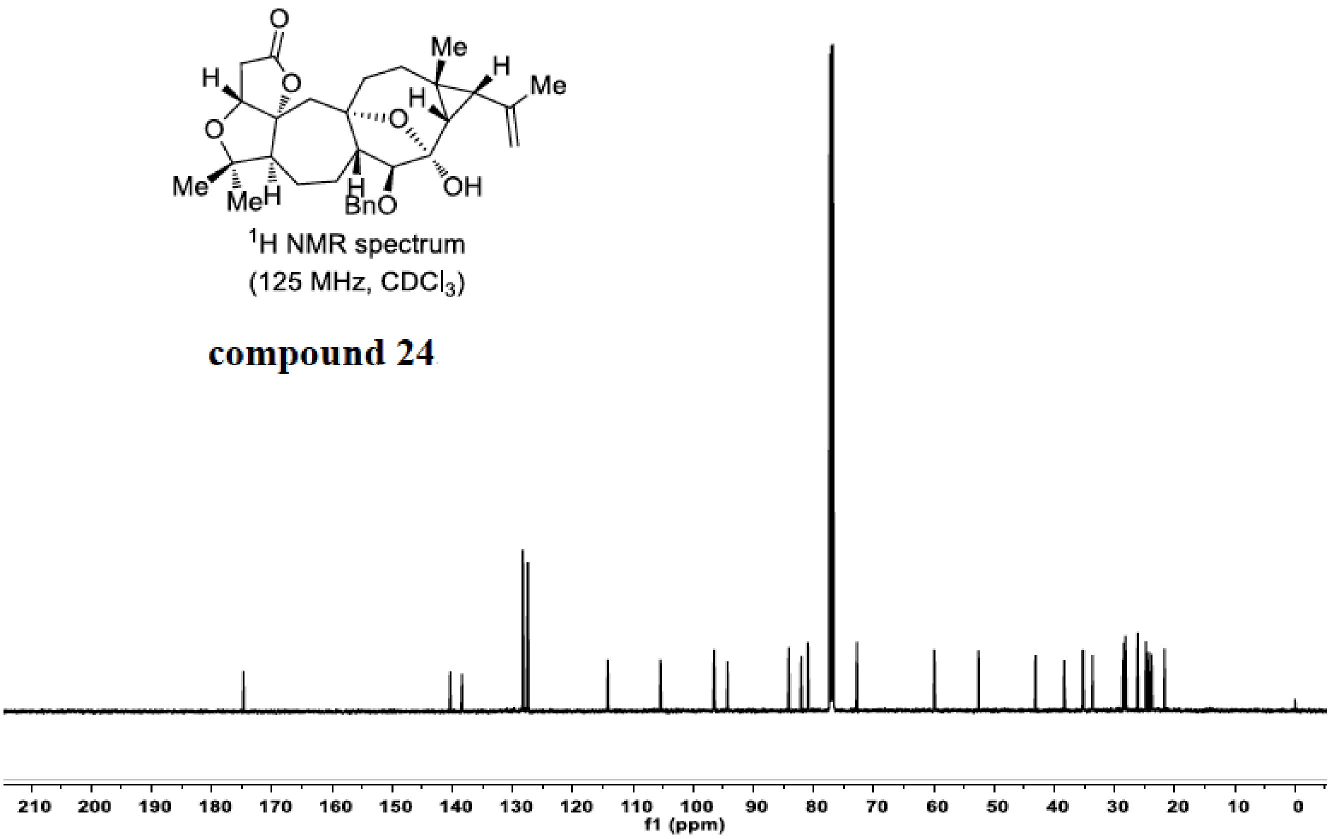

Supplementary Figure 26 | ^{1}H, ^{13}C NMR Spectra for Compound 24

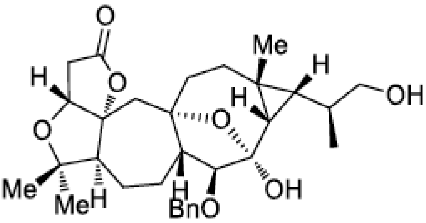

compound 26

^{1}H NMR spectrum
(500 MHz, $CDCl_3$)

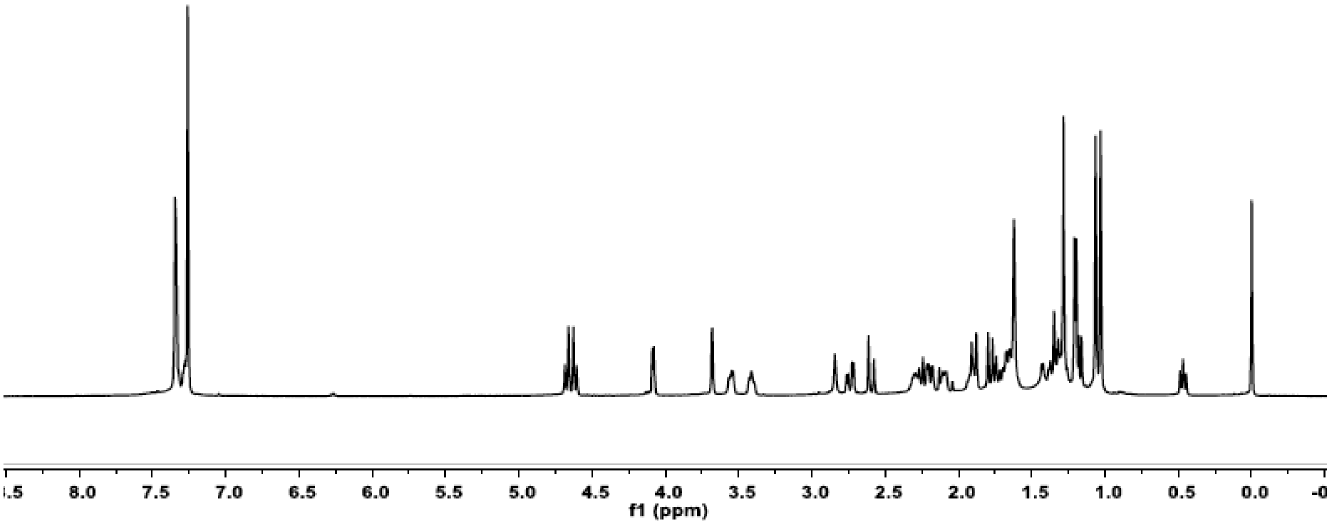

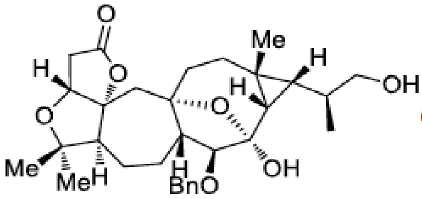

compound 26

^{13}C NMR spectrum
(125 MHz, $CDCl_3$)

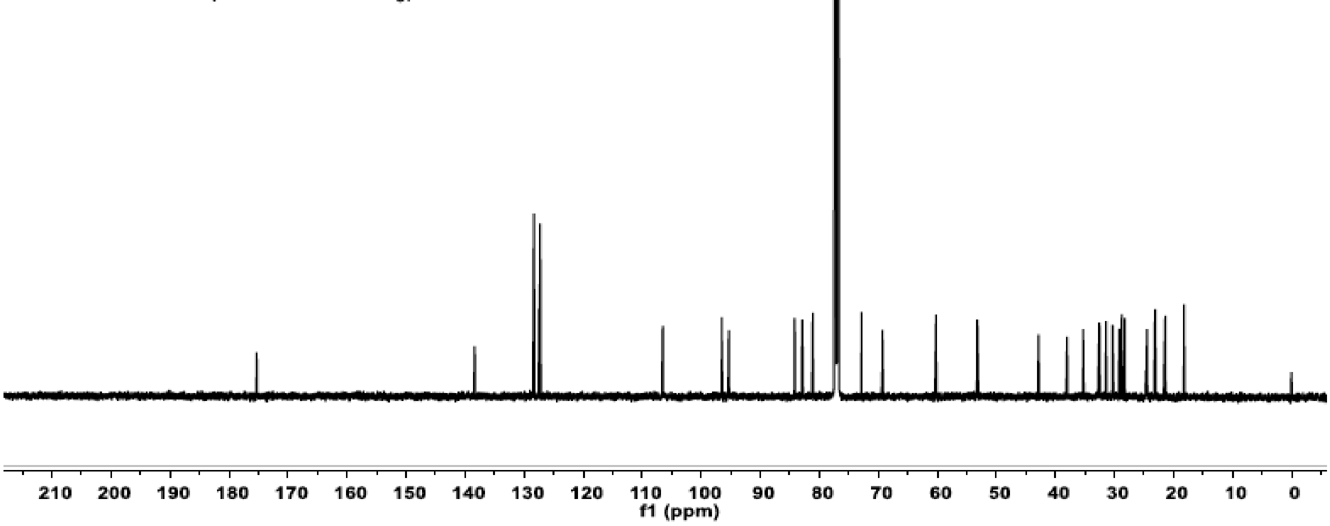

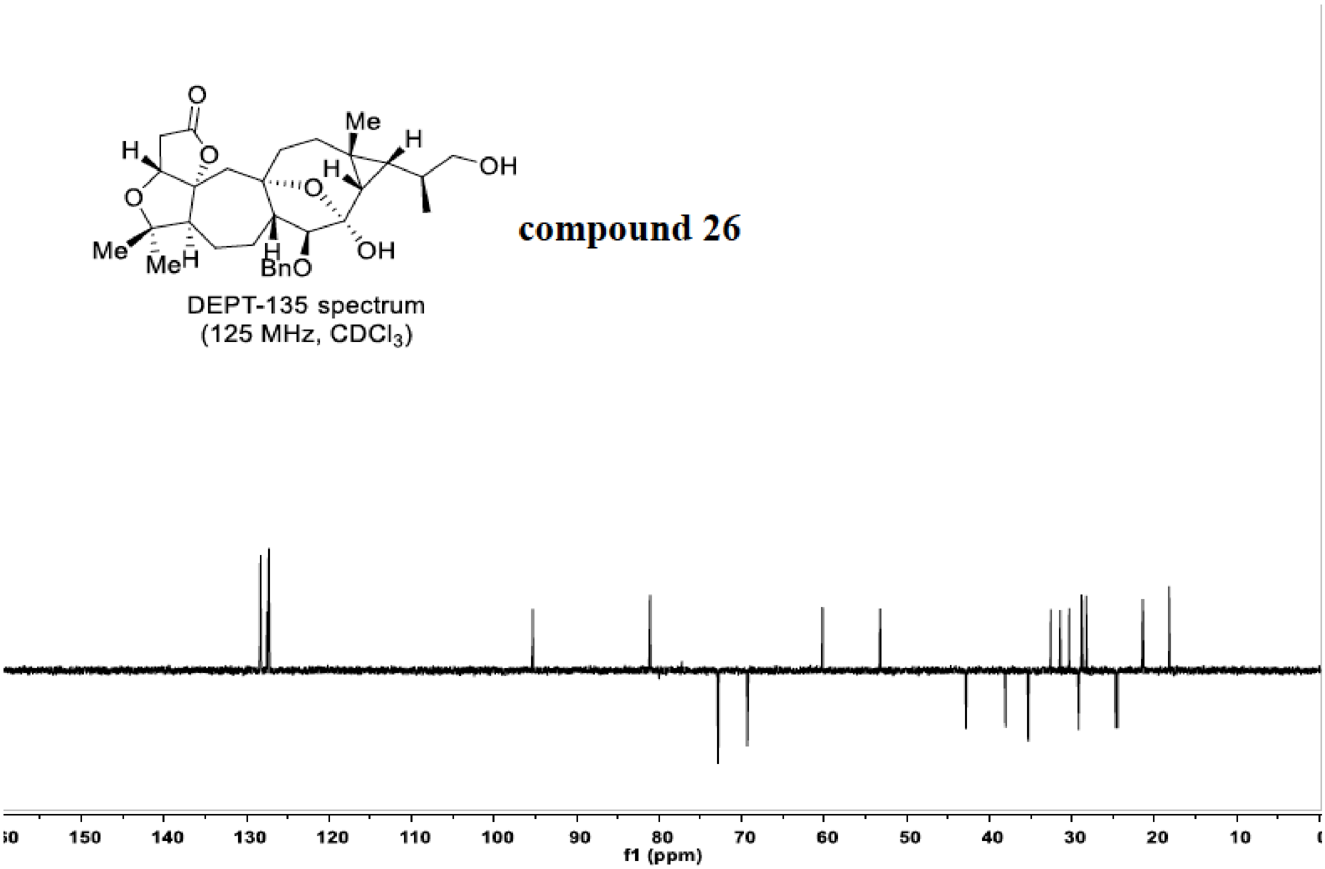
compound 26
DEPT-135 spectrum
(125 MHz, $CDCl_3$)
f1 (ppm)

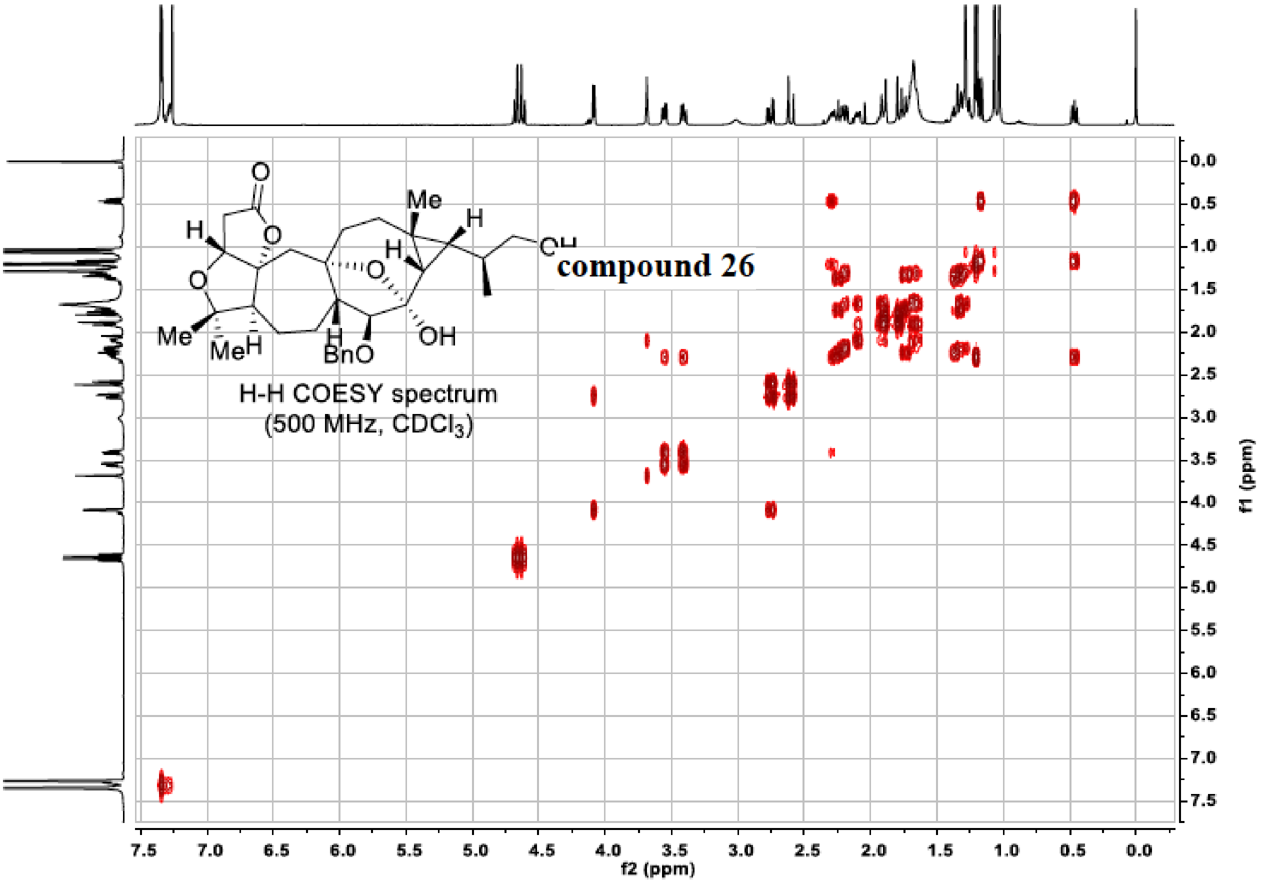
compound 26
H-H COESY spectrum
(500 MHz, $CDCl_3$)
f2 (ppm)
f1 (ppm)

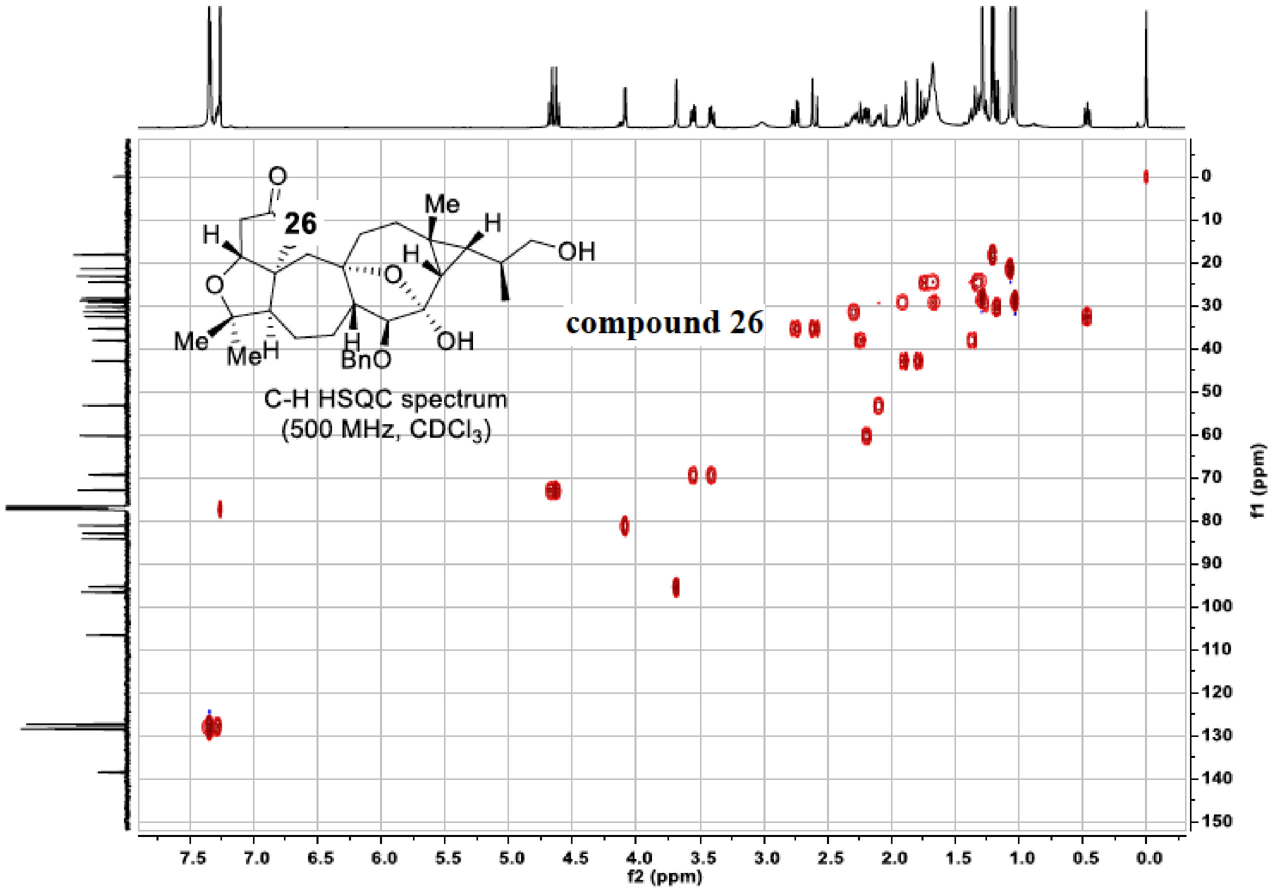
compound 26
C-H HSQC spectrum
(500 MHz, CDCl3)
f2 (ppm)
f1 (ppm)

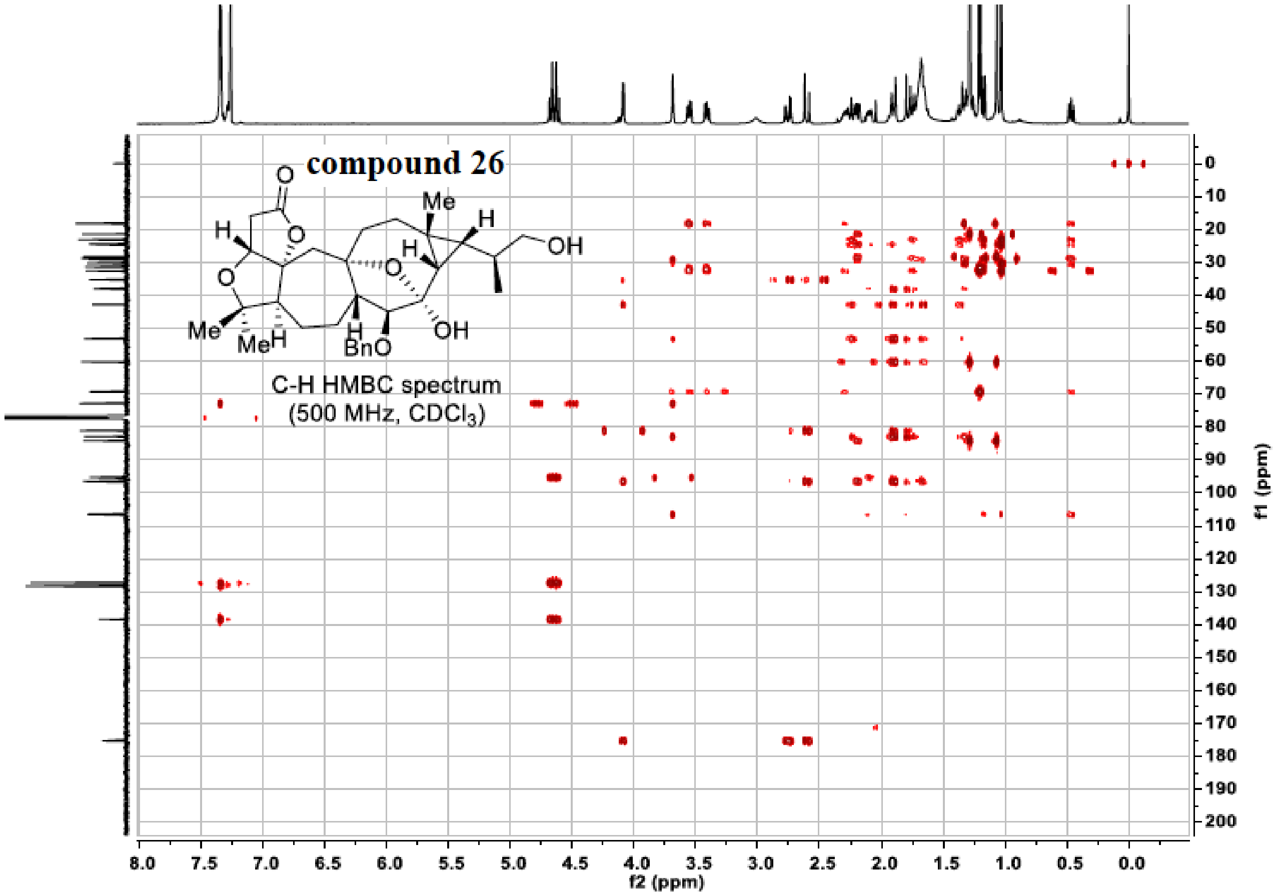
compound 26
C-H HMBC spectrum
(500 MHz, CDCl3)
f2 (ppm)
f1 (ppm)

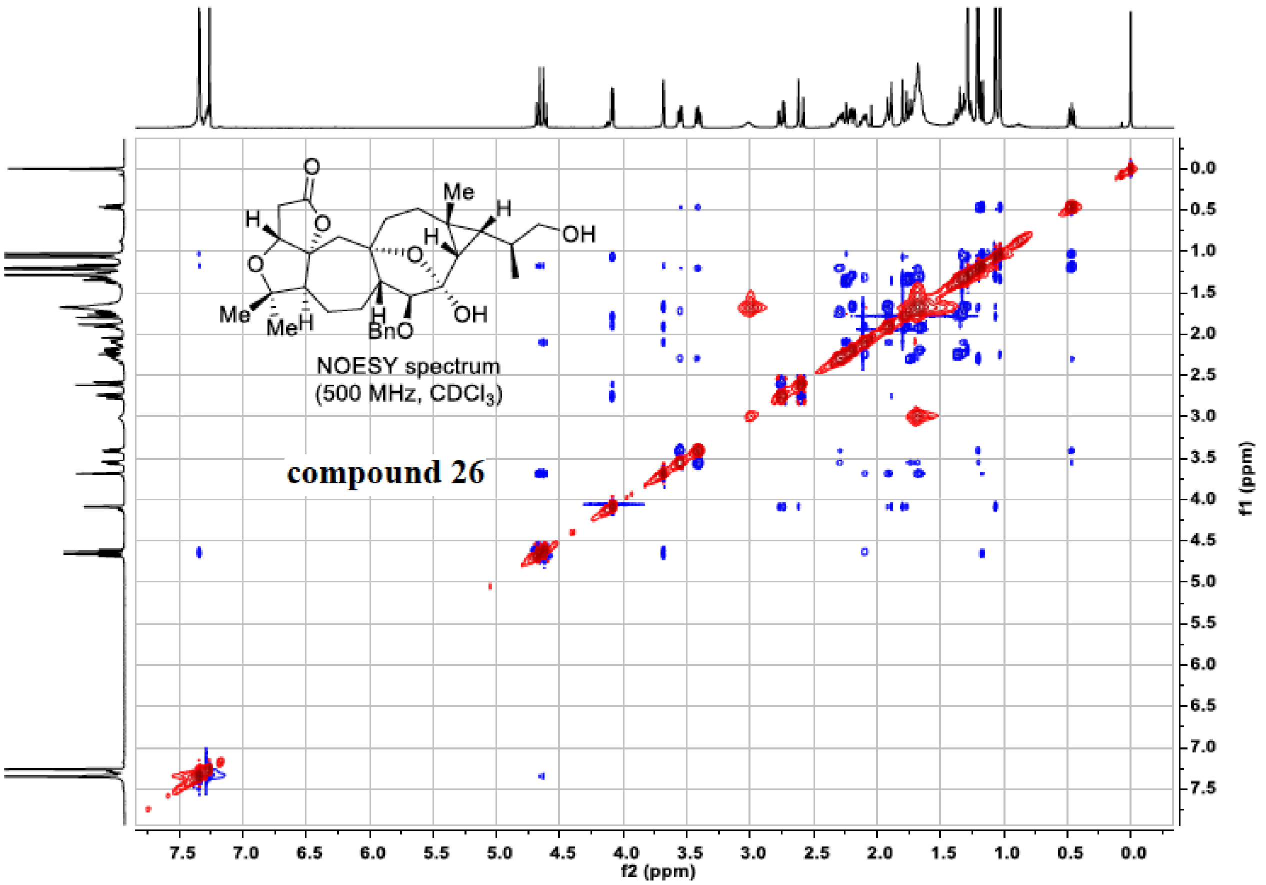

Supplementary Figure 27 | ^{1}H, ^{13}C, DEPT135, COESY, HSQC, HMBC, NOESY Spectra for Compound 26

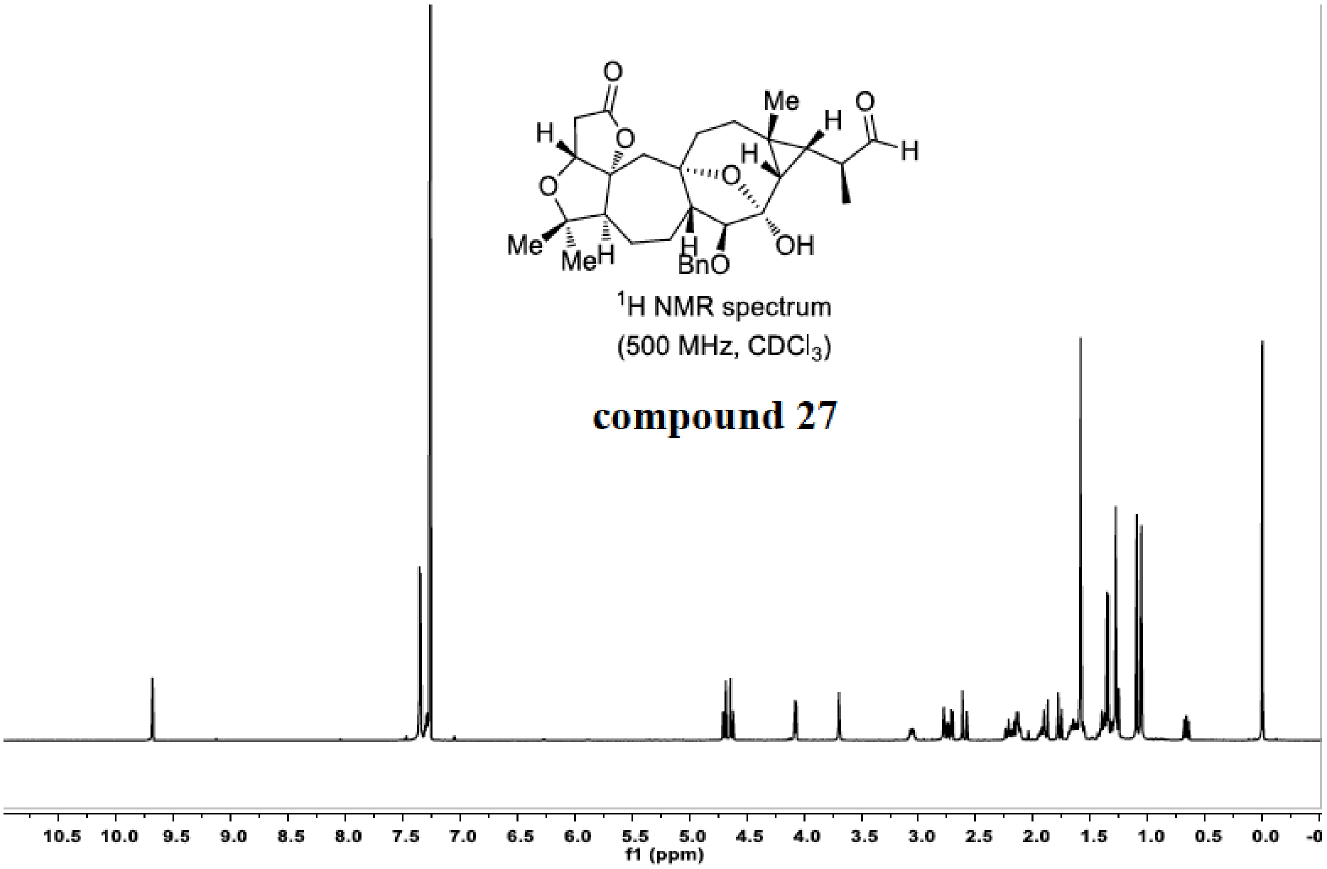

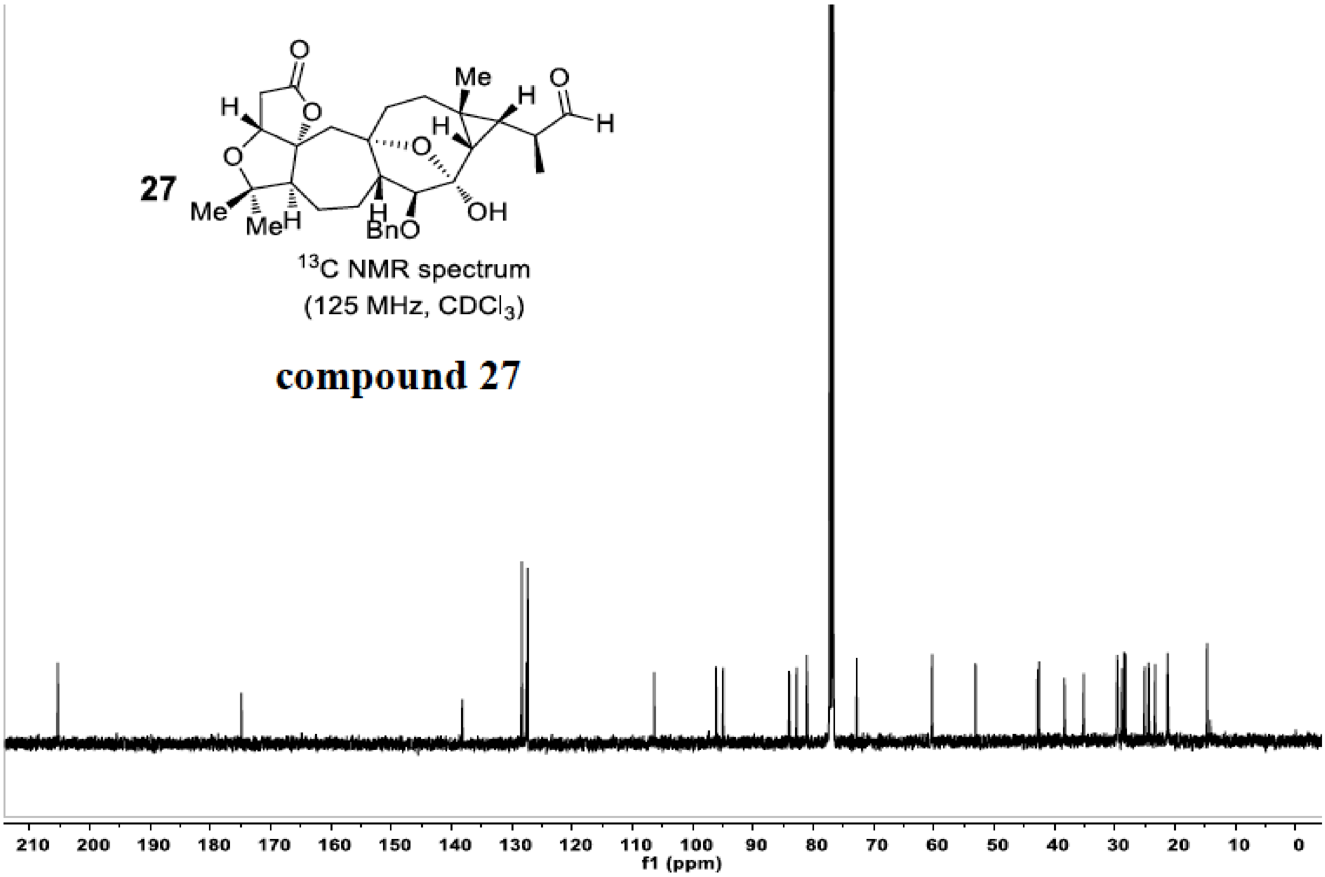

Supplementary Figure 28 | ^{1}H, ^{13}C NMR Spectra for Compound 27

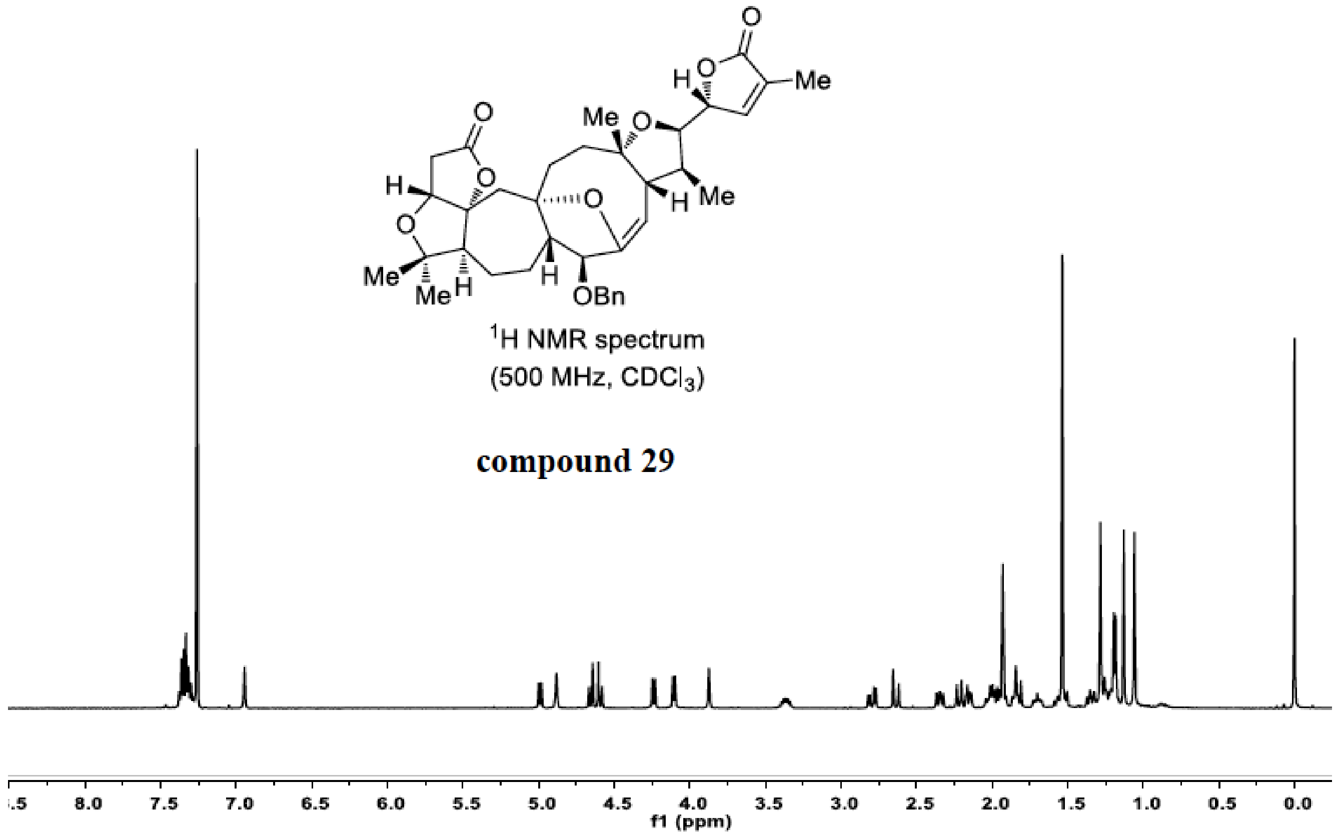

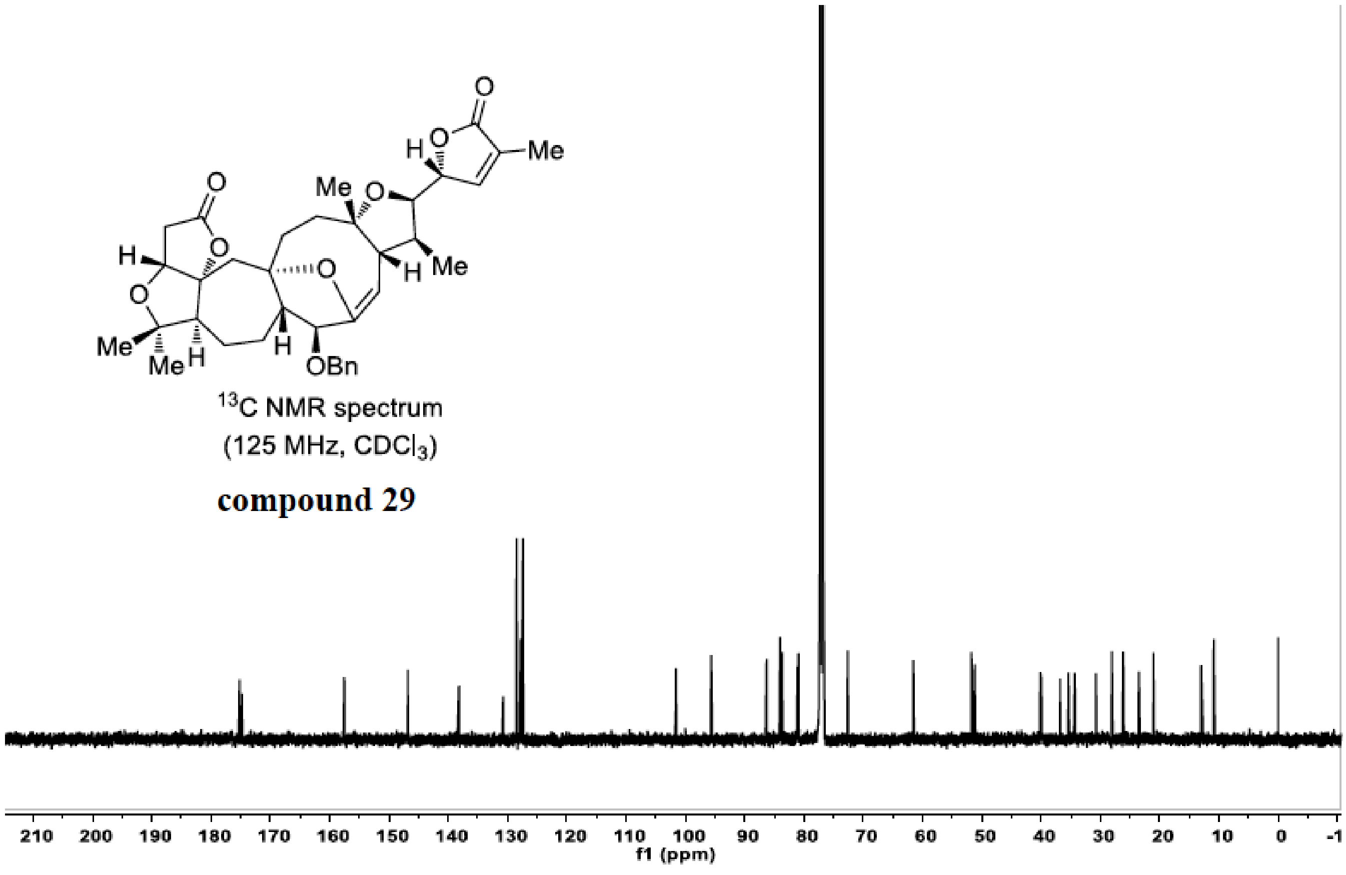

Supplementary Figure 29 | ^{1}H, ^{13}C NMR Spectra for Compound 29

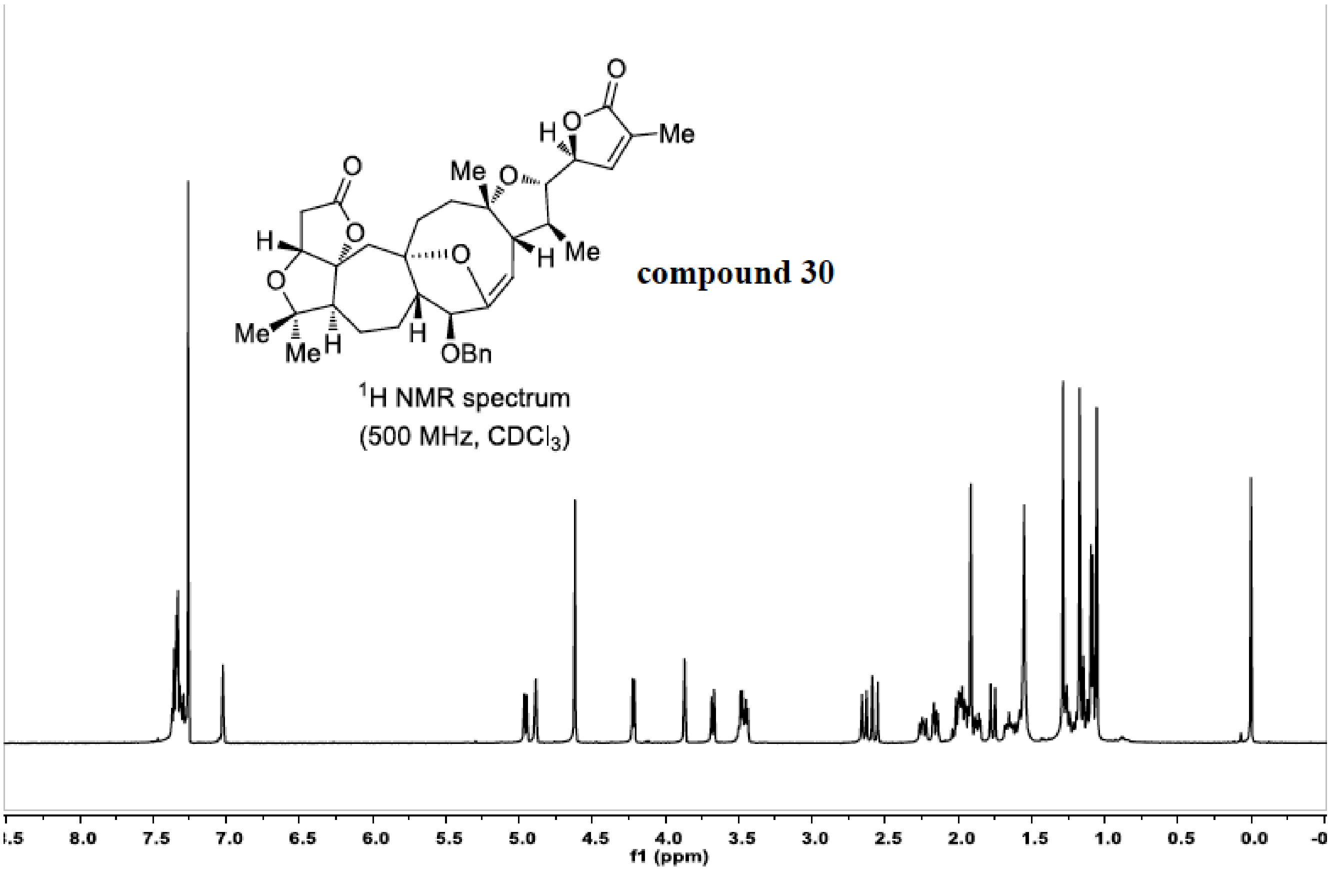

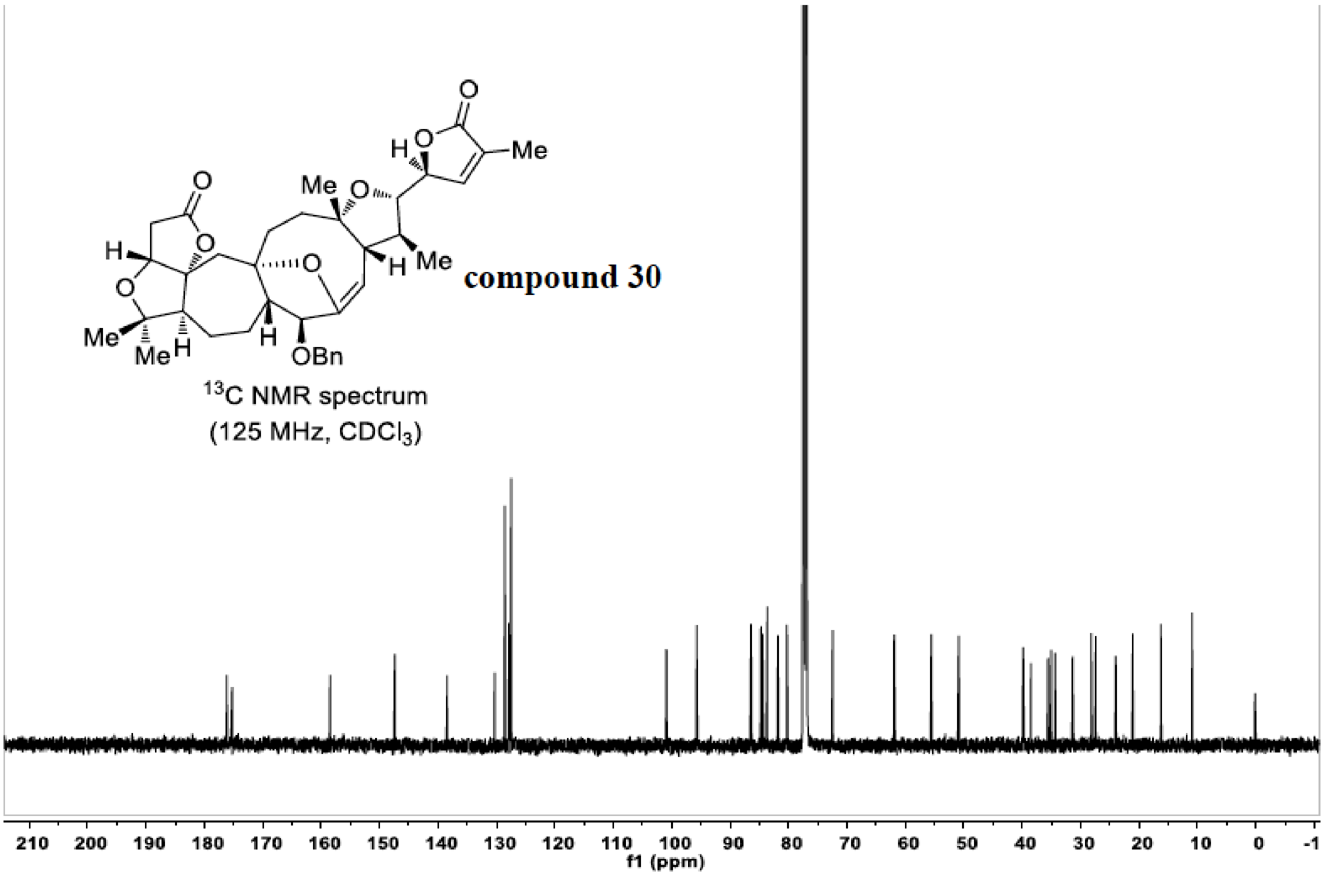

Supplementary Figure 30 | ^{1}H, ^{13}C NMR Spectra for Compound 30

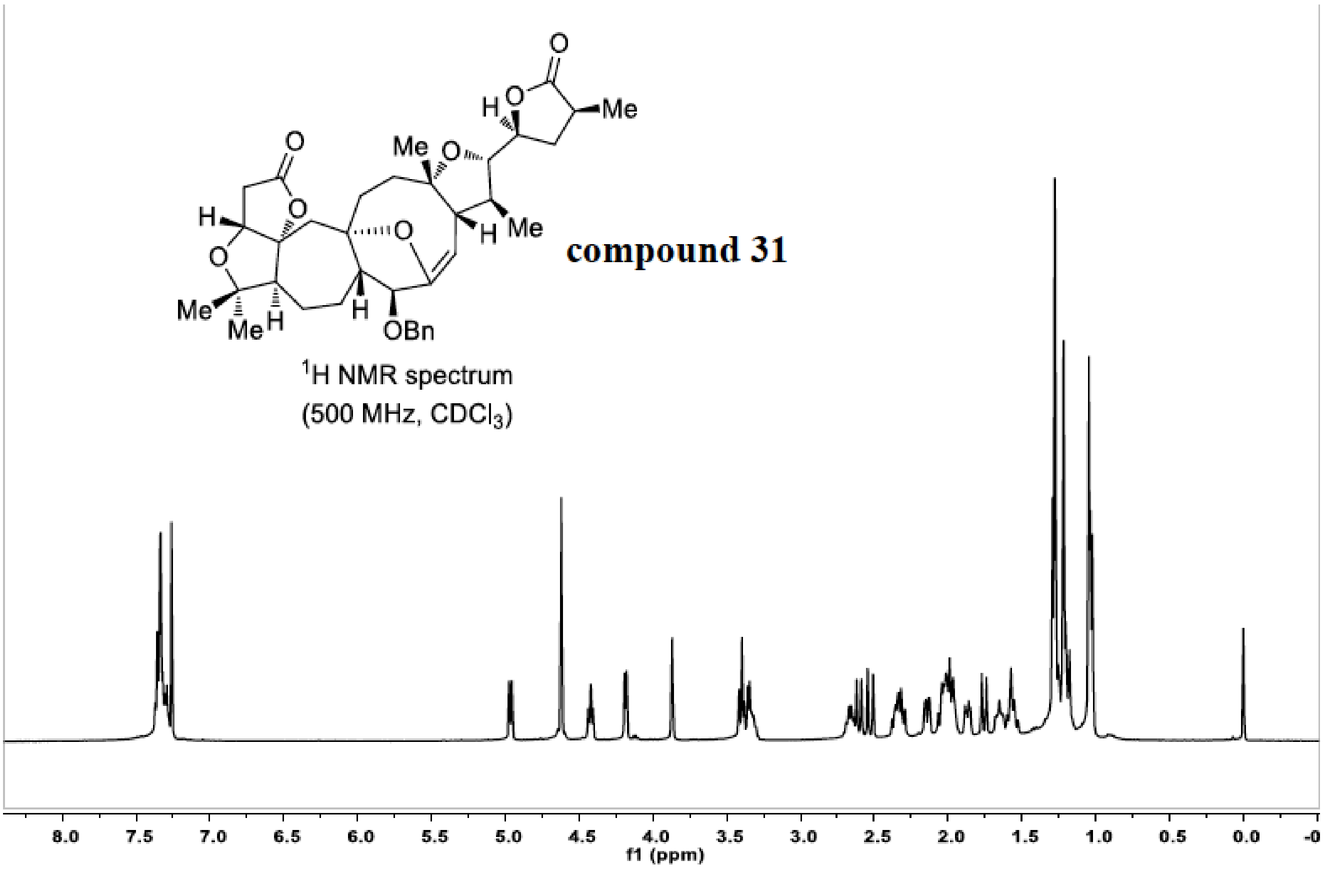

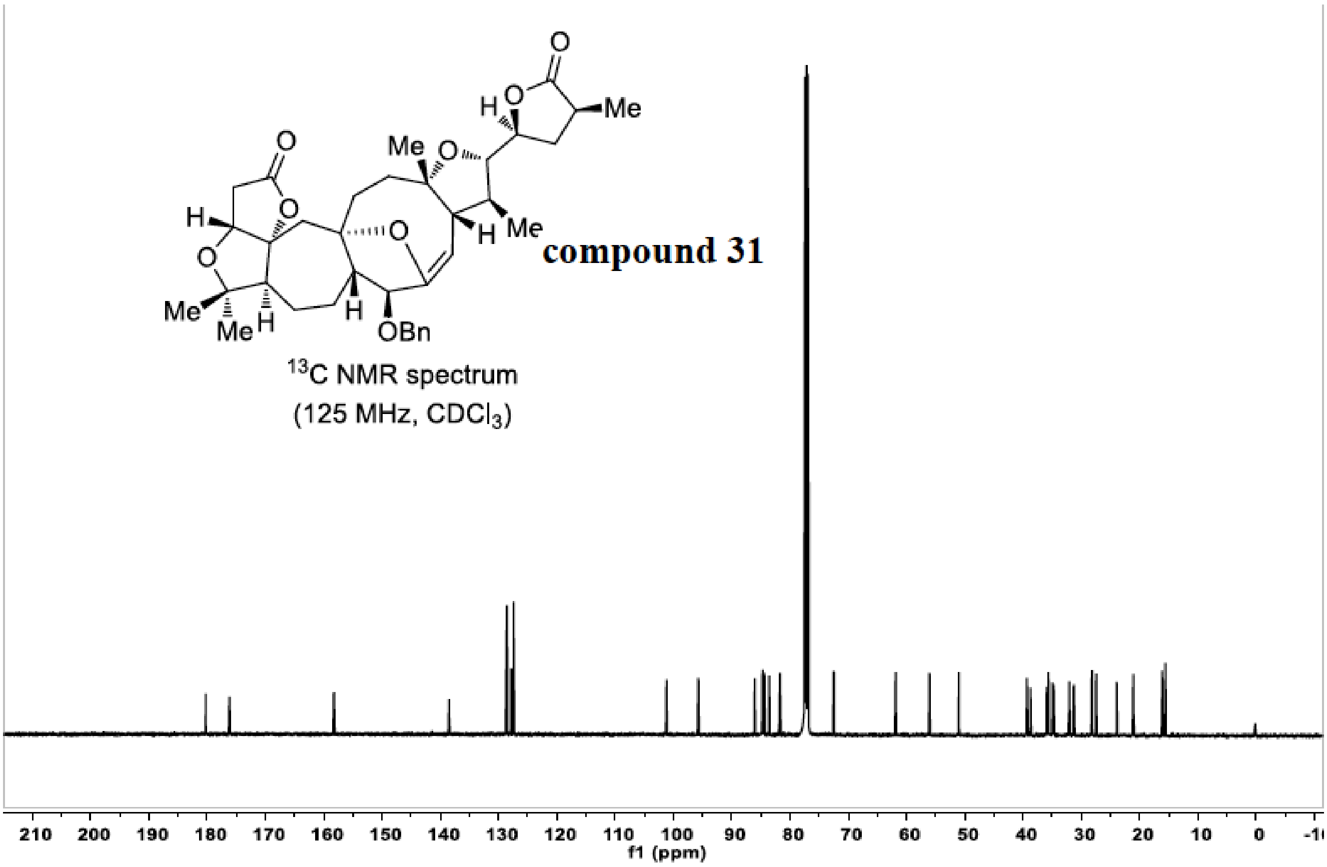

Supplementary Figure 31 | ^{1}H, ^{13}C NMR Spectra for Compound 31

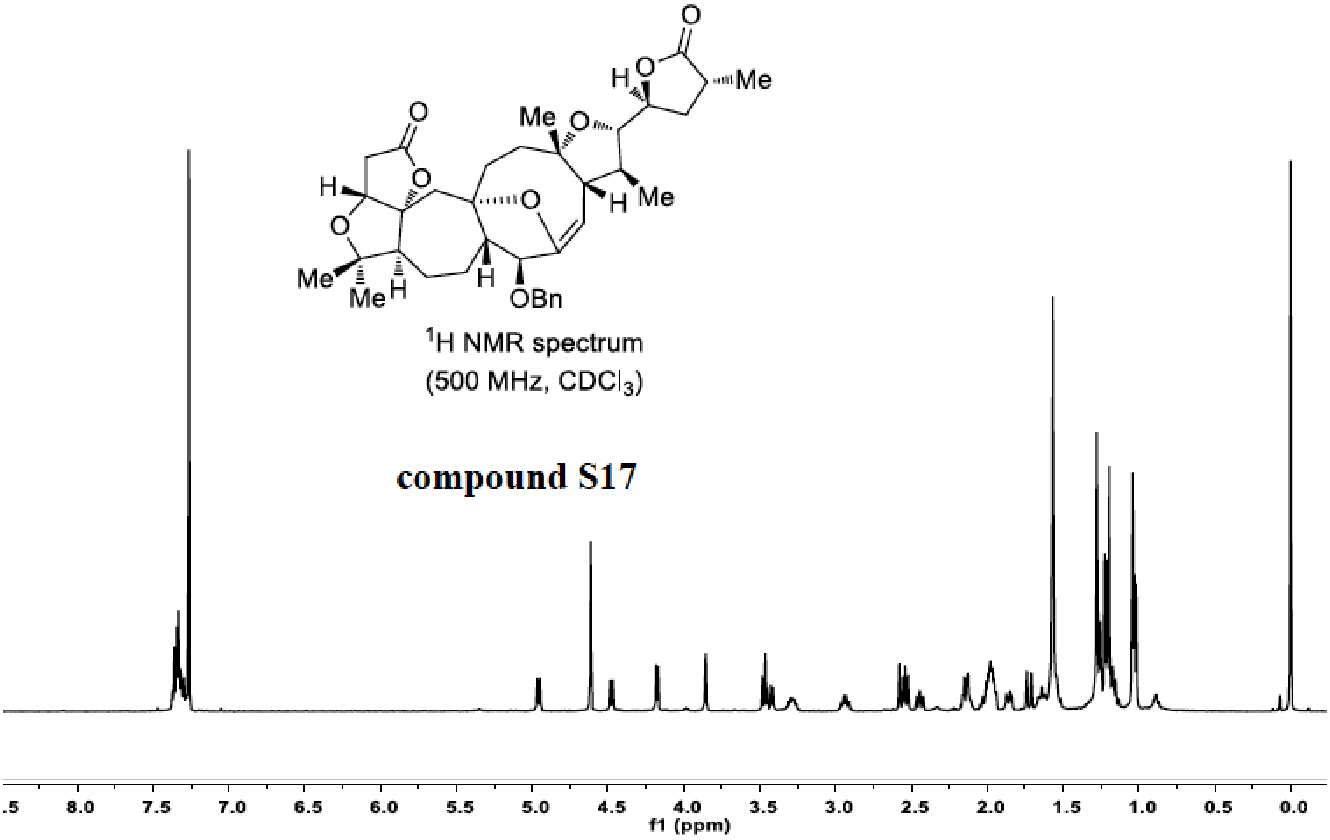

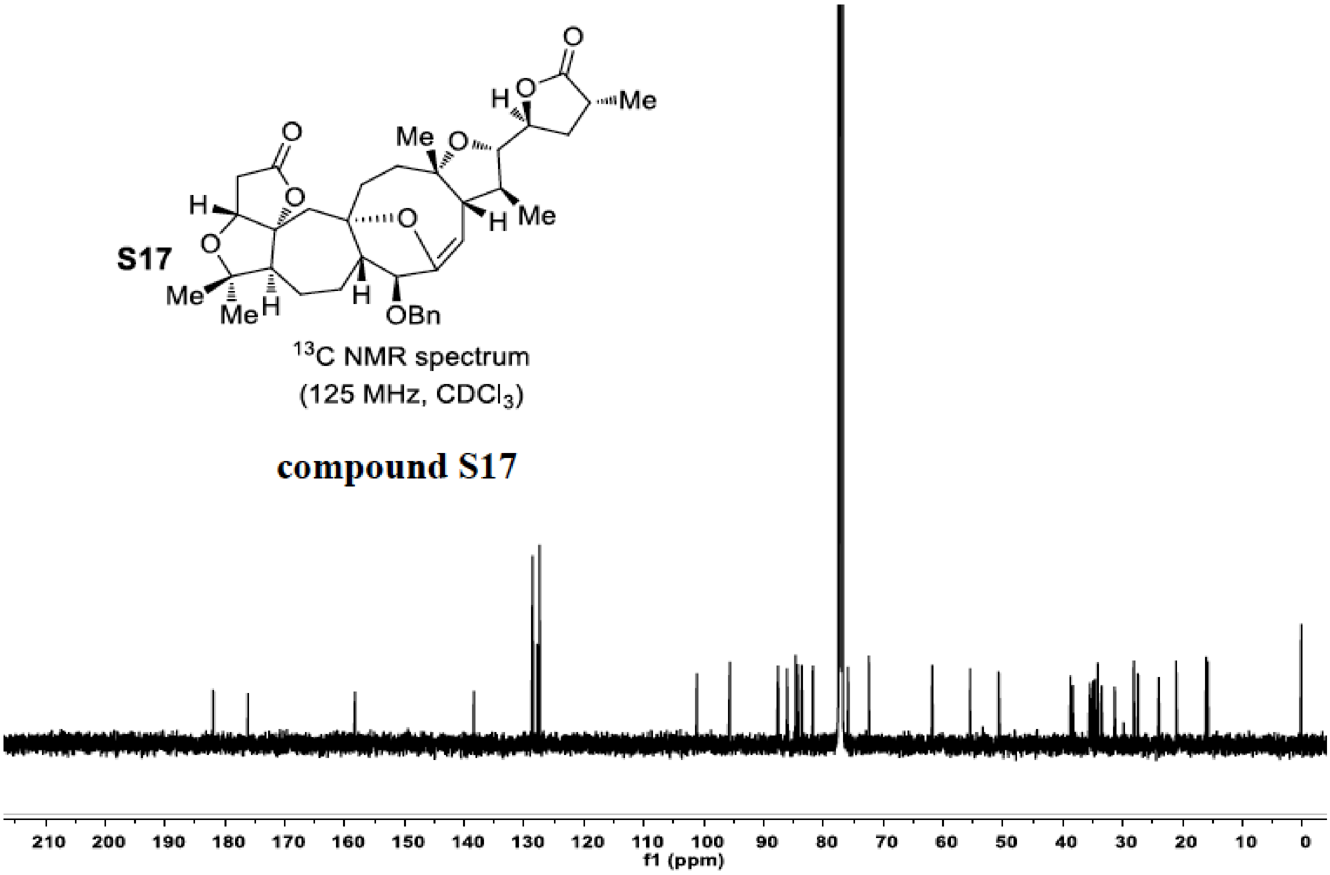

Supplementary Figure 32 | ^{1}H, ^{13}C NMR Spectra for Compound S17

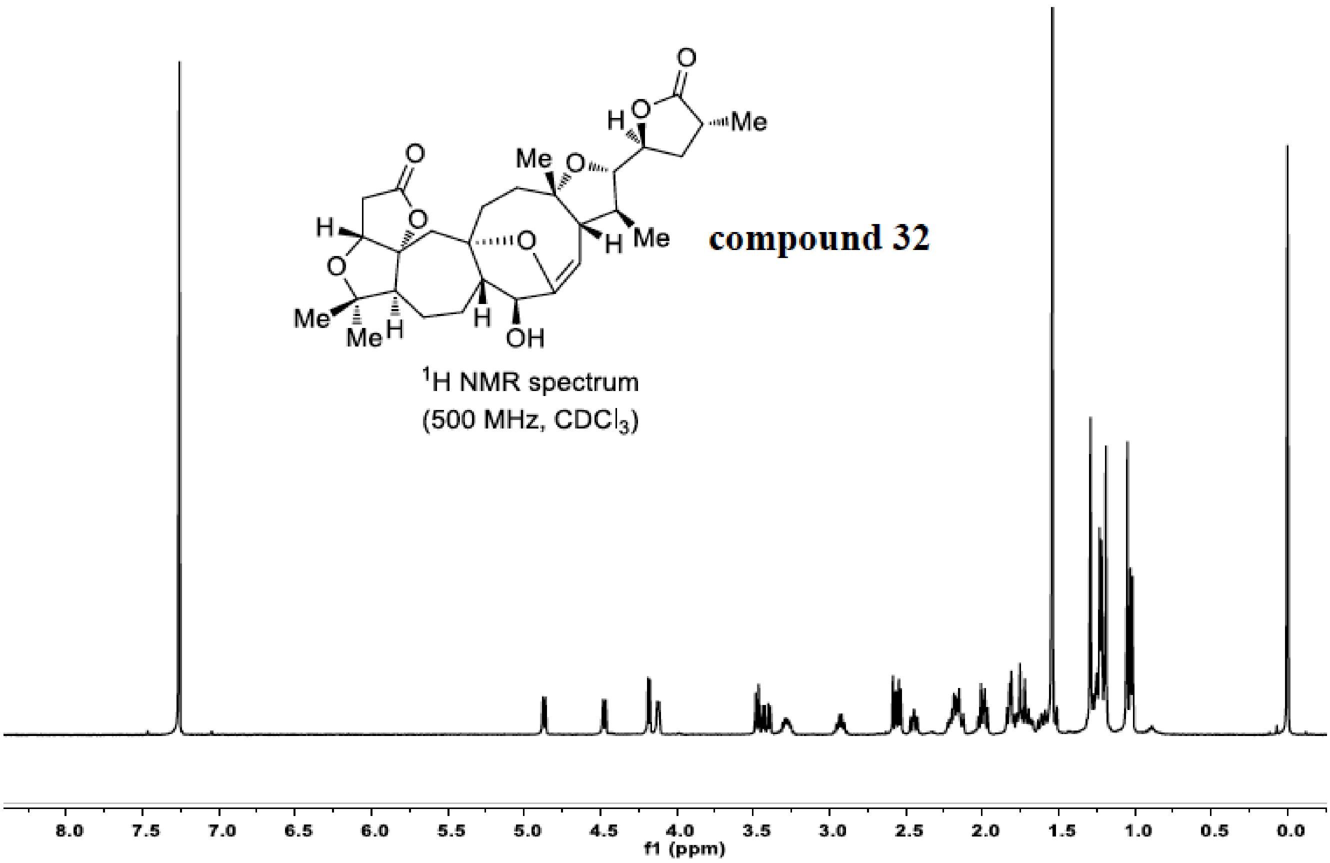

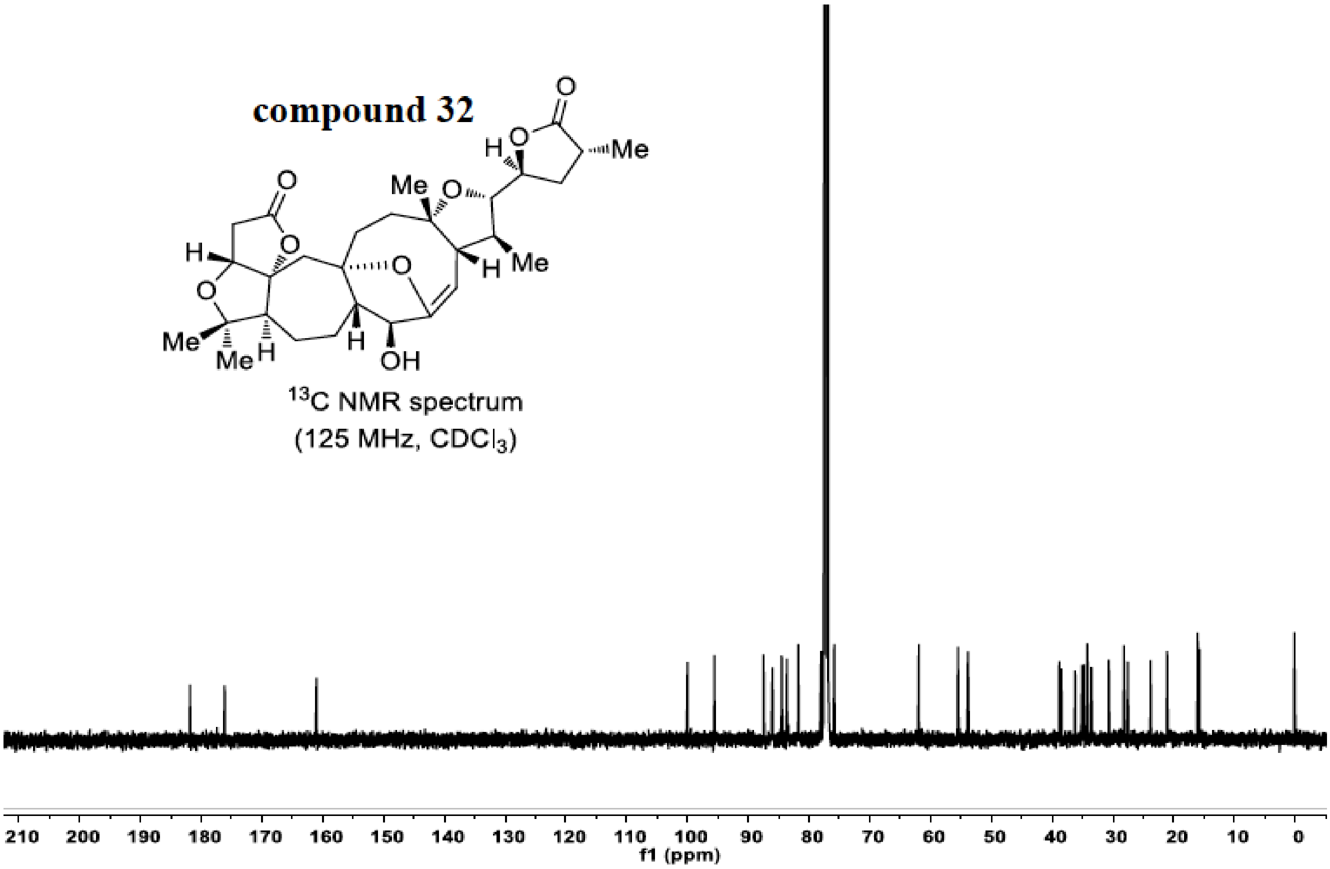

Supplementary Figure 33 | ^{1}H, ^{13}C NMR Spectra for Compound 32

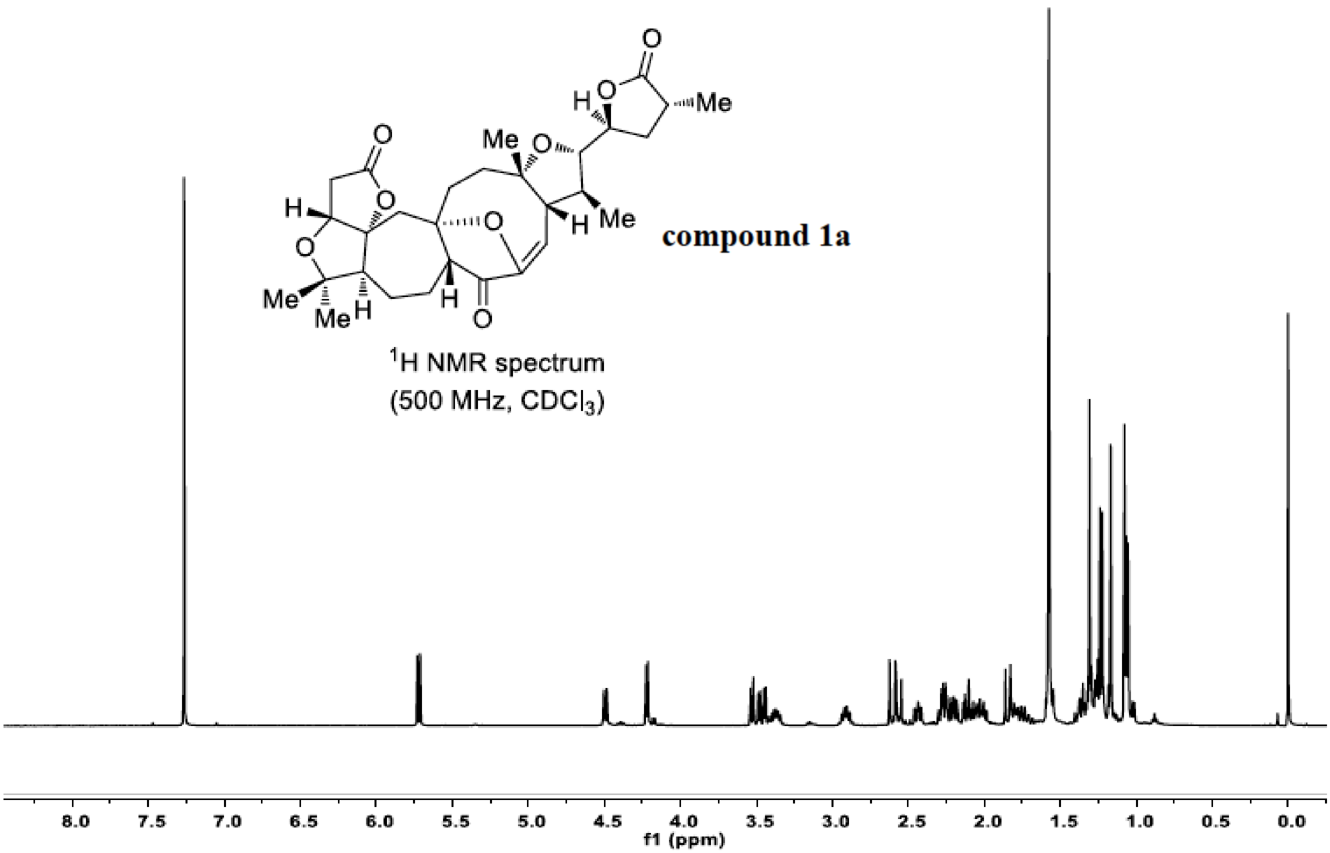
compound 1a
^{1}H NMR spectrum
(500 MHz, CDCl$_3$)
8.0 7.5 7.0 6.5 6.0 5.5 5.0 4.5 4.0 3.5 3.0 2.5 2.0 1.5 1.0 0.5 0.0
f1 (ppm)

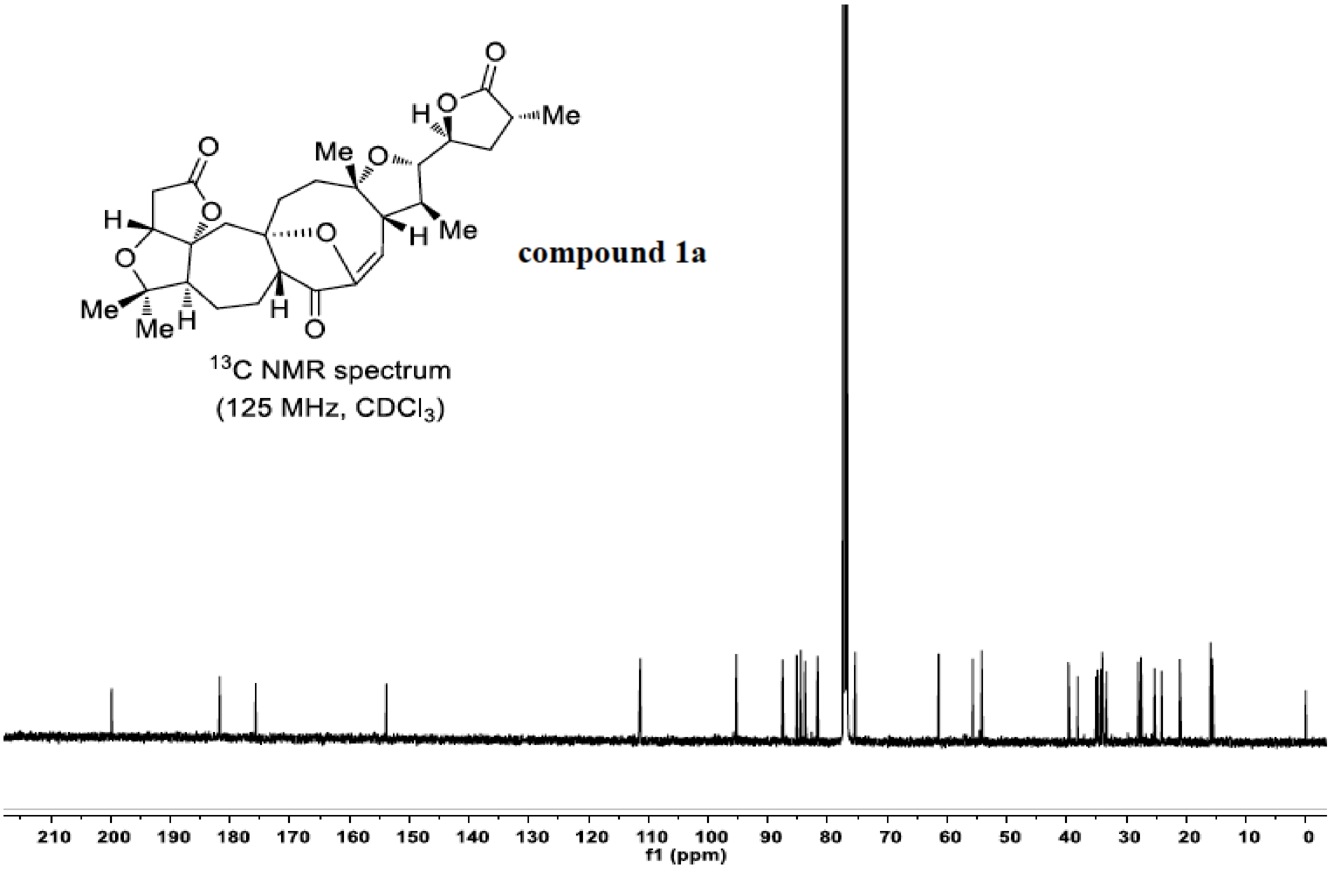
compound 1a
^{13}C NMR spectrum
(125 MHz, CDCl$_3$)
210 200 190 180 170 160 150 140 130 120 110 100 90 80 70 60 50 40 30 20 10 0
f1 (ppm)

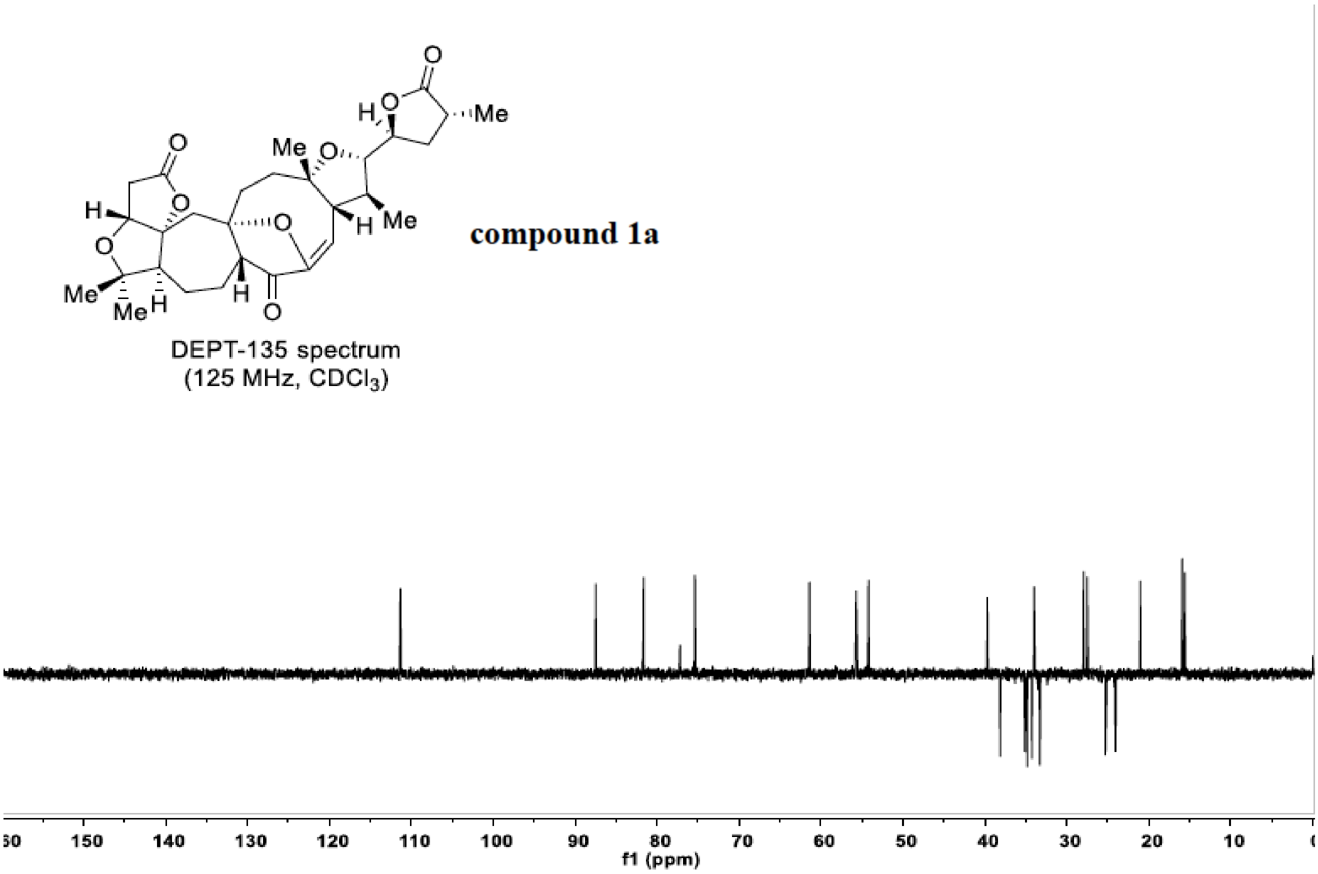
compound 1a
DEPT-135 spectrum
(125 MHz, CDCl3)
f1 (ppm)

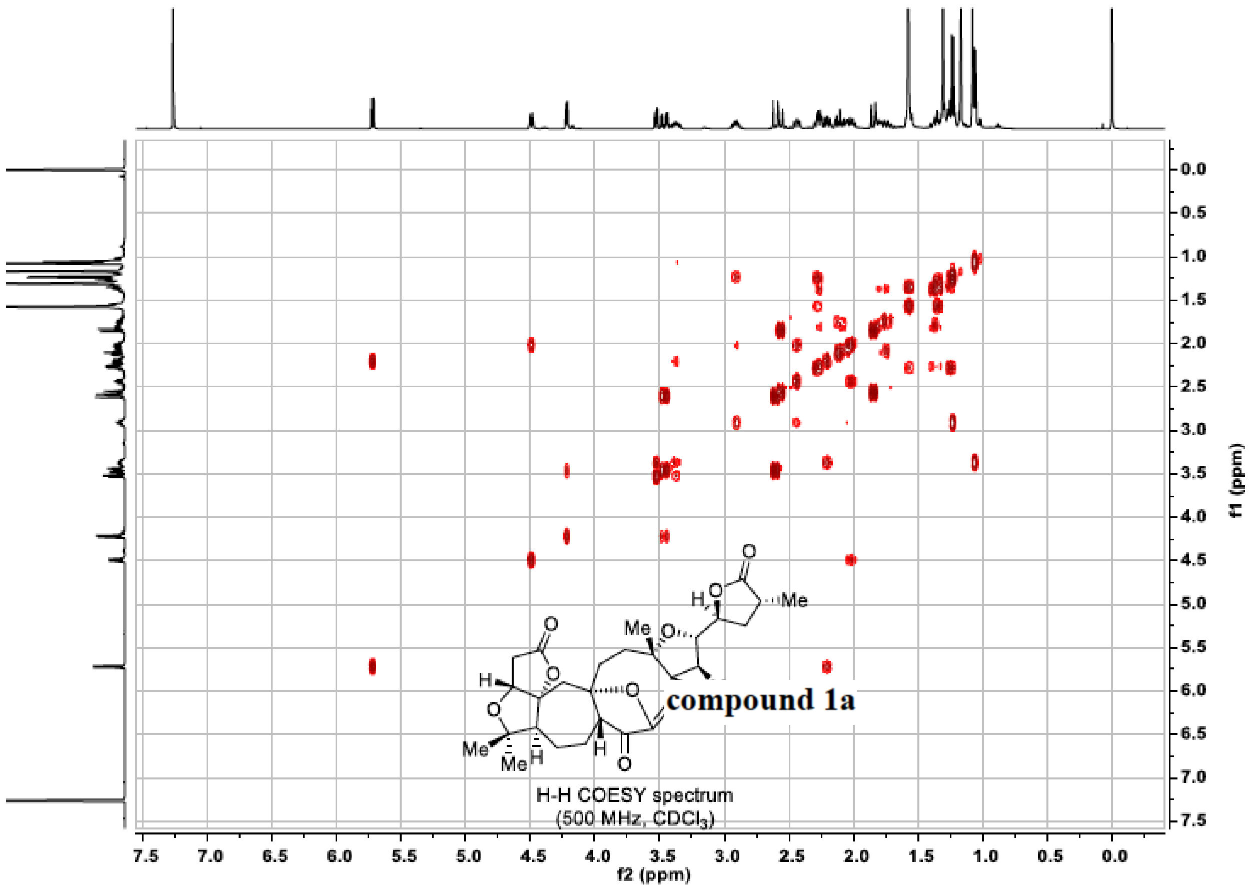
compound 1a
H-H COESY spectrum
(500 MHz, CDCl3)
f2 (ppm)
f1 (ppm)

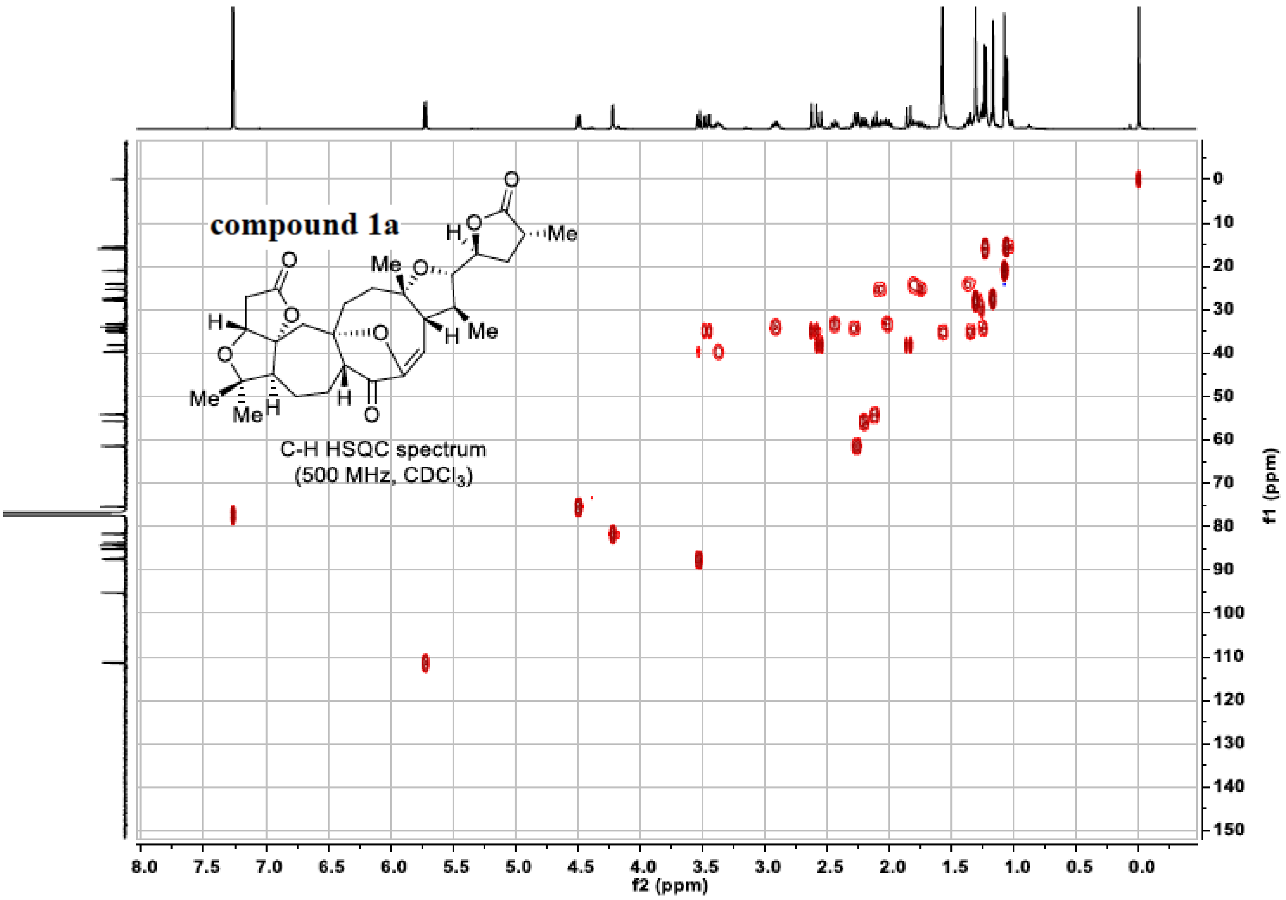

compound 1a
C-H HSQC spectrum
(500 MHz, CDCl3)
f2 (ppm)
f1 (ppm)

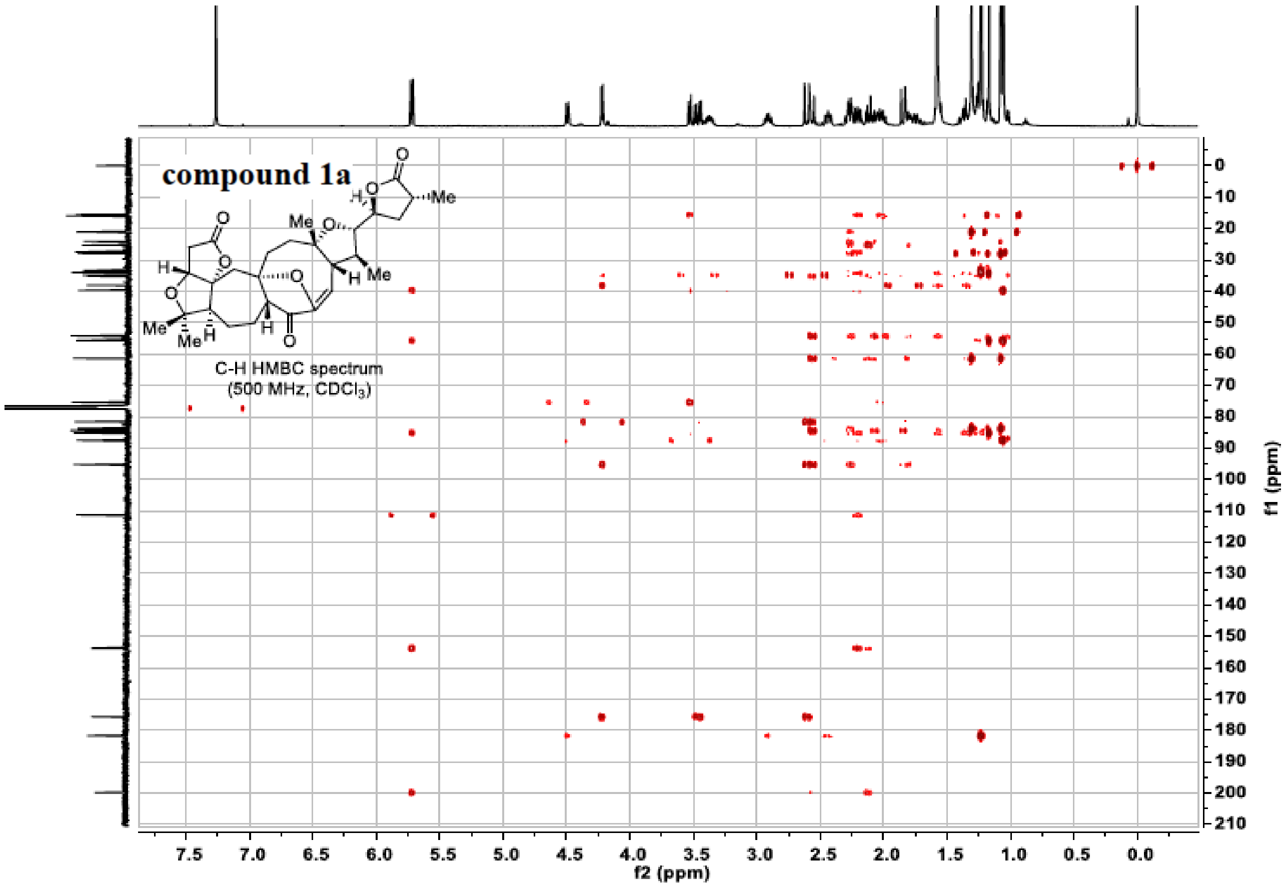

compound 1a
C-H HMBC spectrum
(500 MHz, CDCl3)
f2 (ppm)
f1 (ppm)

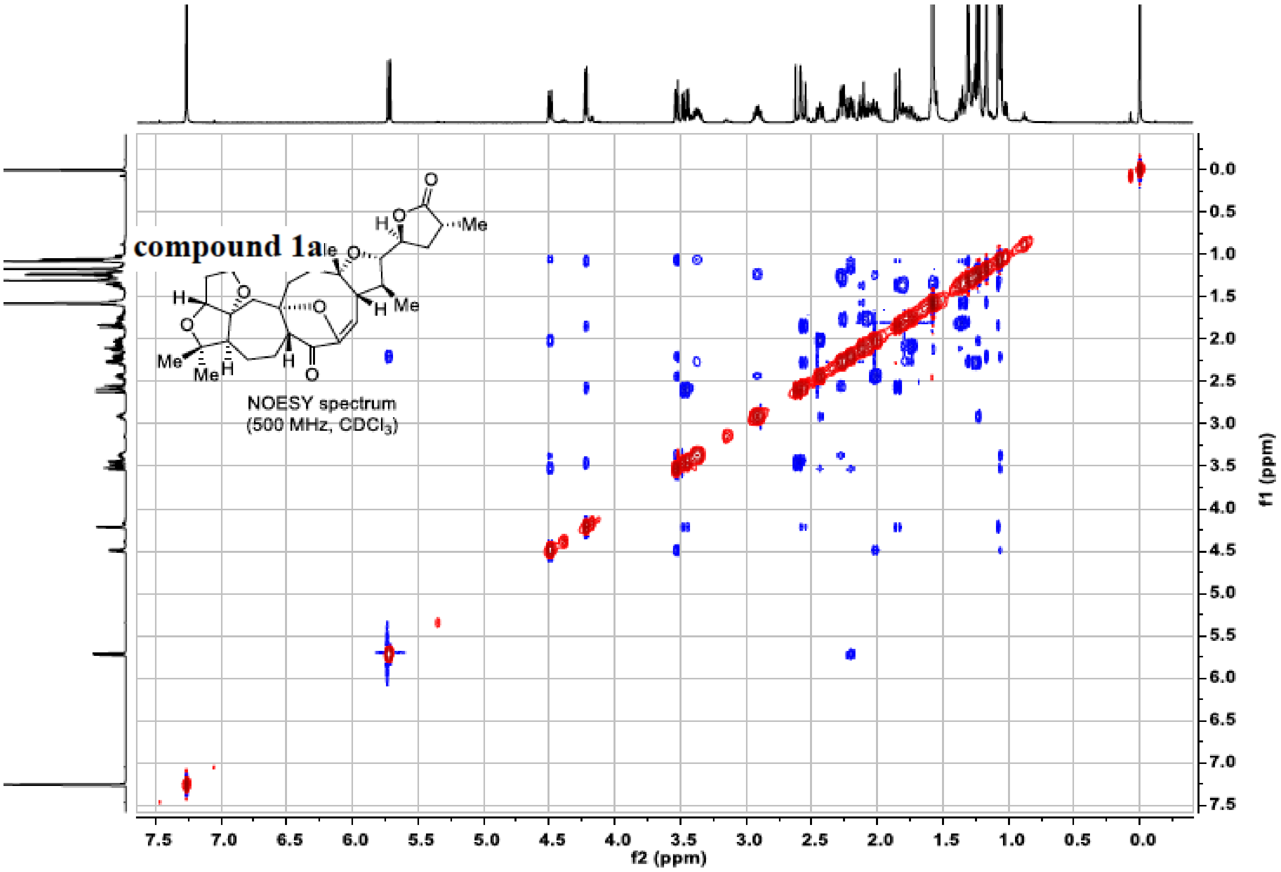

Supplementary Figure 34 | ^{1}H, ^{13}C, DEPT135, COESY, HSQC, HMBC, NOESY Spectra for Compound 1a

Comparison of NMR spectrum of natural Arisandilactone A and synthetic 19-dehydroxyl Arisandilactone A

no.	δH in ppm (mult, *J* in Hz) natural	δH synthetic	*Δδ*H in ppm	δC in ppm natural	δC synthetic	*Δδ*C in ppm
**1**	4.72 (d, 5.7)	4.22 (d, 6.1)	0.50	78.9	81.8	-2.9
**2**	2.61 (d, 18.3)	α: 2.60 (d, 18.4)	0.01	34.9	35.0	-0.1
	3.31 (dd, 5.7, 18.3)	β: 3.46 (dd, 18.4, 6.2)	-0.15			
**3**				175.7	175.8	-0.1
**4**				84.4	83.9	0.5
**5**	2.30 (m)	2.26 (dd, 13.0, 4.5)	0.04	62.0	61.5	0.5
**6**	1.98 (m)	α: 1.80 (dt, 4.5, 9.2)	0.18	22.7	24.2	-1.5
	1.56 (m)	β: 1.40-1.35 (m)	0.18			
**7**	1.70 (m)	α: 1.74 (dt, 10.0, 13.5)	-0.04	26.1	25.4	0.7
	2.03 (m)	β: 2.08 (dd, 14.5, 9.0)	-0.05			
**8**	2.35 (m)	2.12 (dd, 12.7, 5.6)	0.23	52.7	54.3	-1.6
**9**				87.4	84.6	2.8
**10**				97.2	95.4	1.8
**11**	1.44 (m)	α: 1.37-1.32 (m)	0.09	31.5	35.3	-3.8
	1.98 (m)	β: 1.57 (dd, 14.0, 9.5)	0.41			
**12**	2.24 (m)	α: 2.28 (dd, 13.8, 9.2)	-0.04	33.9	34.4	-0.5
	1.20 (m)	β: 1.27-1.21 (m)	-0.04			
**13**				85.1	85.3	-0.2
**14**				199.9	200.0	-0.1
**15**				153.5	153.9	-0.4
**16**	5.73 (d, 8.6)	5.72 (d, 8.7)	0.01	111.5	111.5	0.0
**17**	2.21 (m)	2.20 (dd, 12.3, 8.7)	0.01	55.8	55.9	-0.1
**18**	1.17 (s)	1.17 (s)	0.00	27.4	27.7	-0.3
**19**	3.93 (d, 7.9)	α: 2.56 (d, 15.9)	1.37	68.7	38.3	30.4
		β: 1.85 (d, 15.9)	-			
**20**	3.36 (m)	3.37 (ddq, 12.8, 9.5, 6.2)	-0.01	40.0	39.8	0.2
**21**	1.08 (d, 6.3)	1.06 (d, 6.4)	0.02	15.7	15.7	0.0
**22**	3.52 (d, 9.6)	3.53 (dd, 9.5, 1.0)	-0.01	87.7	87.6	0.1
**23**	4.52 (d, 8.8)	4.49 (d, 9.2)	0.03	76.0	75.5	0.5
**24**	2.45 (m)	α: 2.44 (ddd, 12.4, 9.4, 1.8)	0.01	33.7	33.4	0.3
	2.03 (m)	β: 2.02 (dt, 12.5, 10.0)	0.01			
**25**	2.92 (m)	2.91 (ddq, 10.0, 9.5, 7.2)	0.01	34.4	34.2	0.2
**26**				182.9	181.9	1.0
**27**	1.22 (overlapped)	1.23 (d, 7.3)	-0.01	16.1	16.1	0.0
**29 (β)**	1.21 (s)	1.08 (s)	0.13	21.7	21.2	0.5
**30 (α)**	1.31 (s)	1.31 (s)	0.00	28.6	28.1	0.5
**OH**	4.03 (d, 7.9)		-			

Supplementary Table 1 | NMR Spectra Comparison.

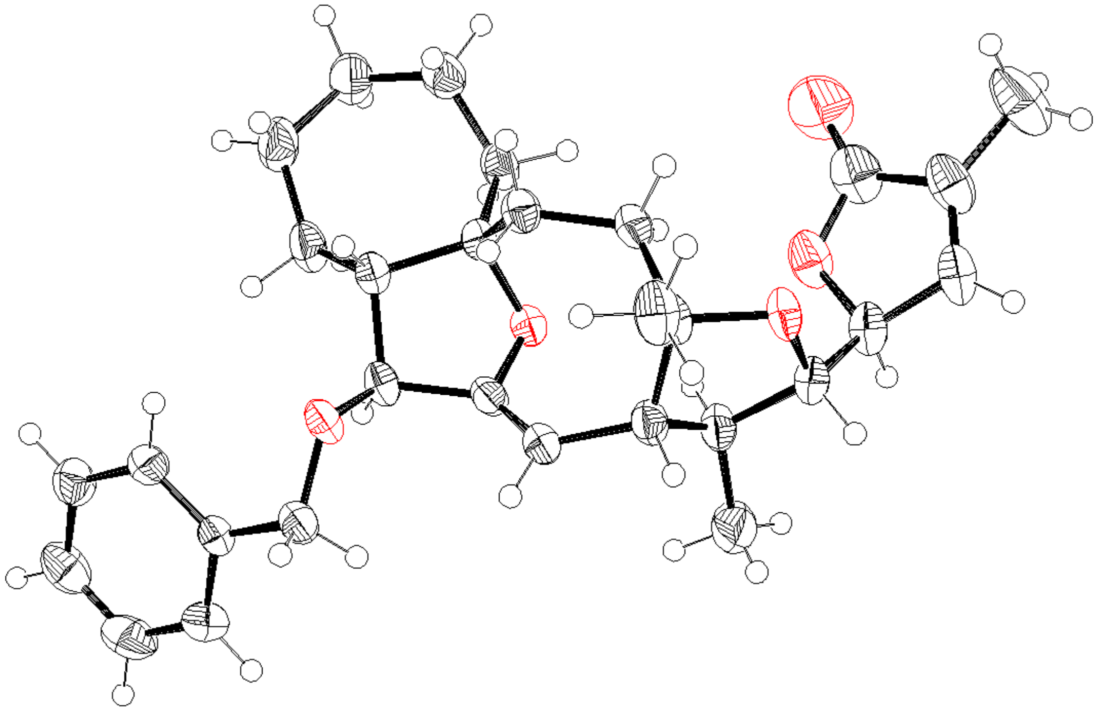

Supplementary Figure 35 | ORTEP of compound 10

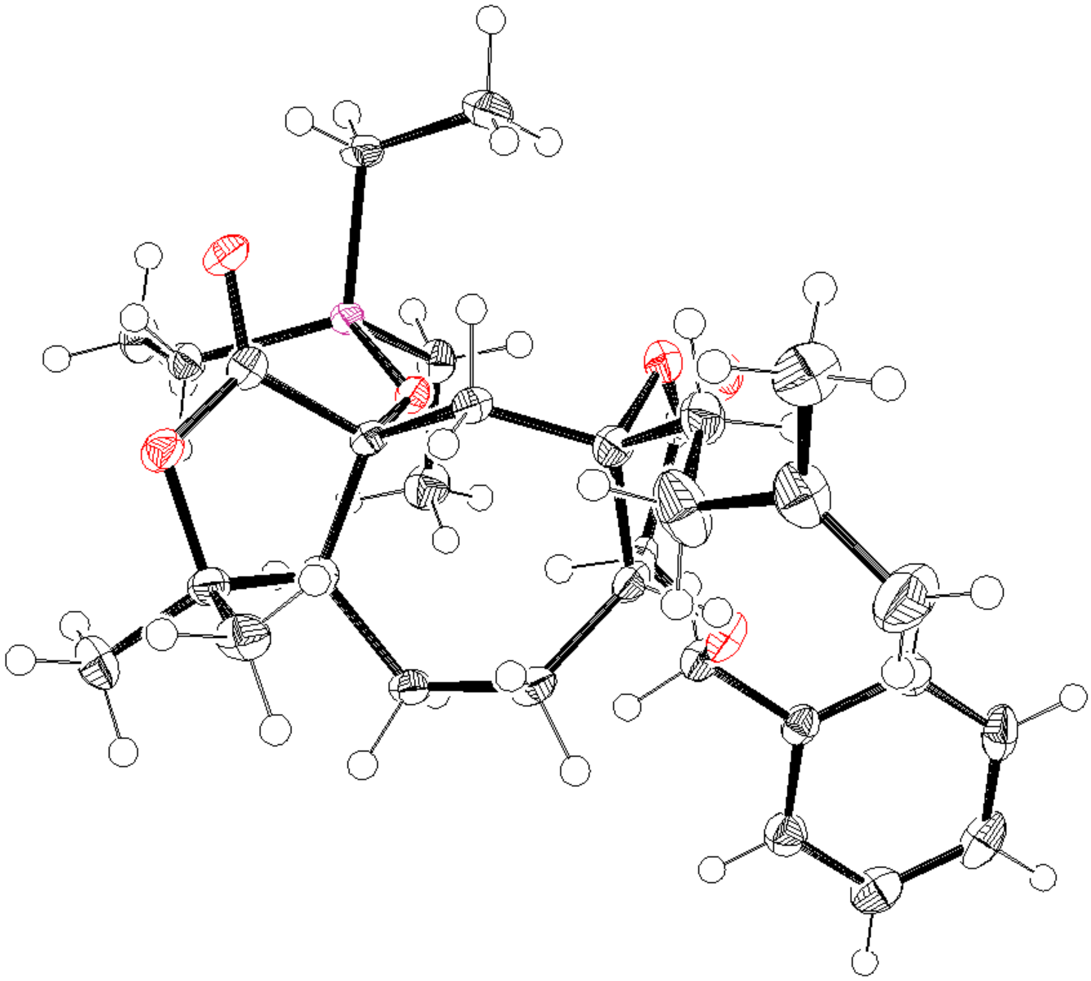

Supplementary Figure 36 | ORTEP of compound 16

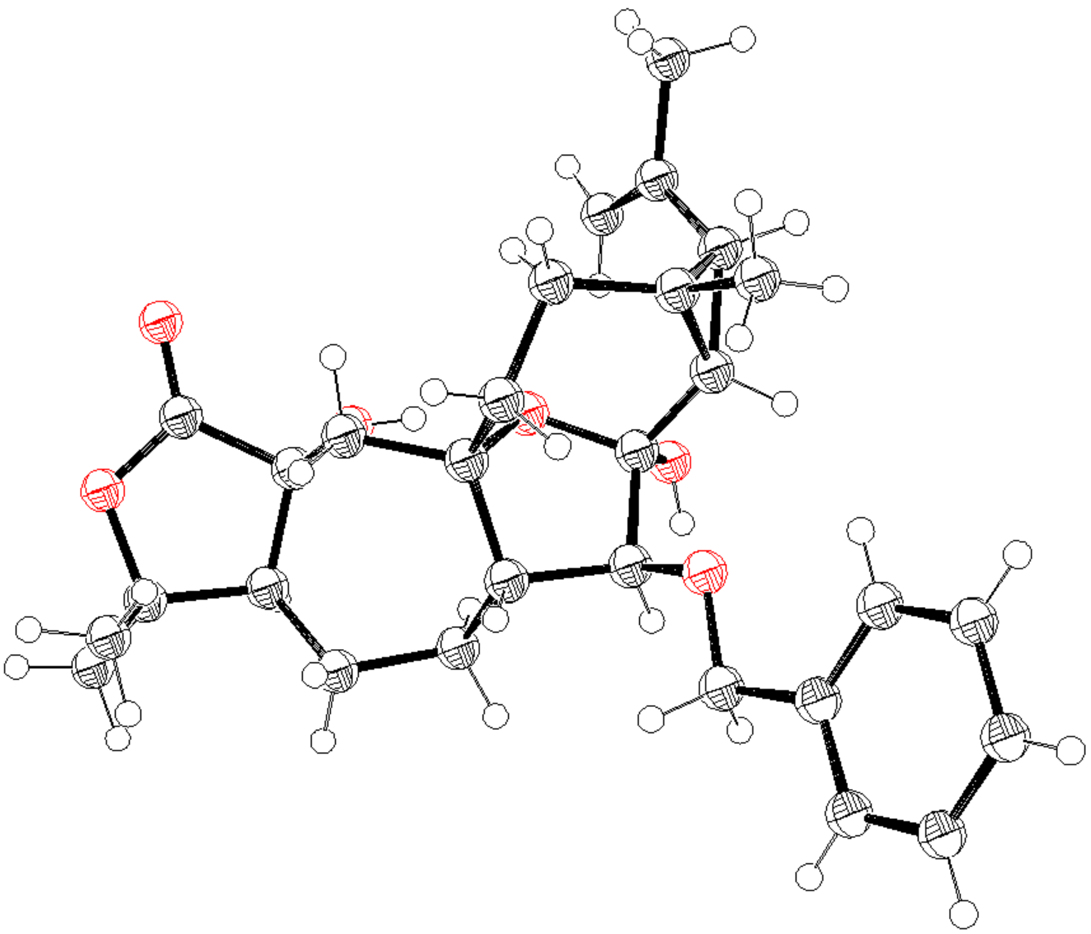

Supplementary Figure 37 | ORTEP of compound 21

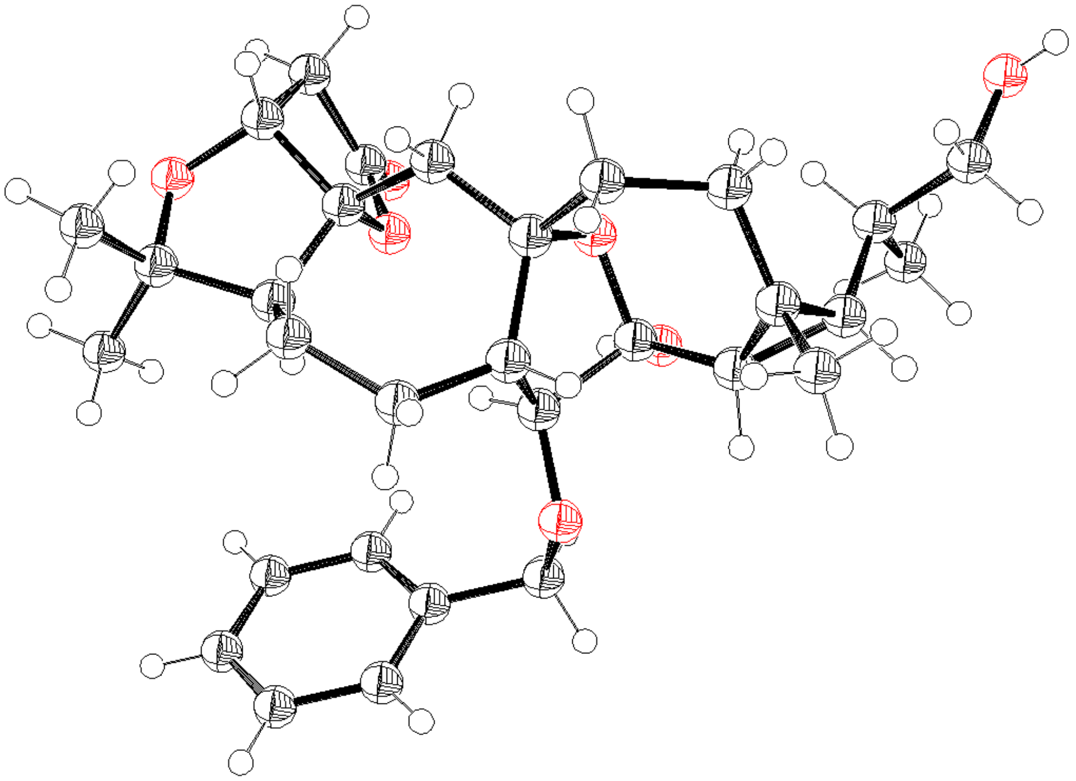

Supplementary Figure 38 | ORTEP of compound 26

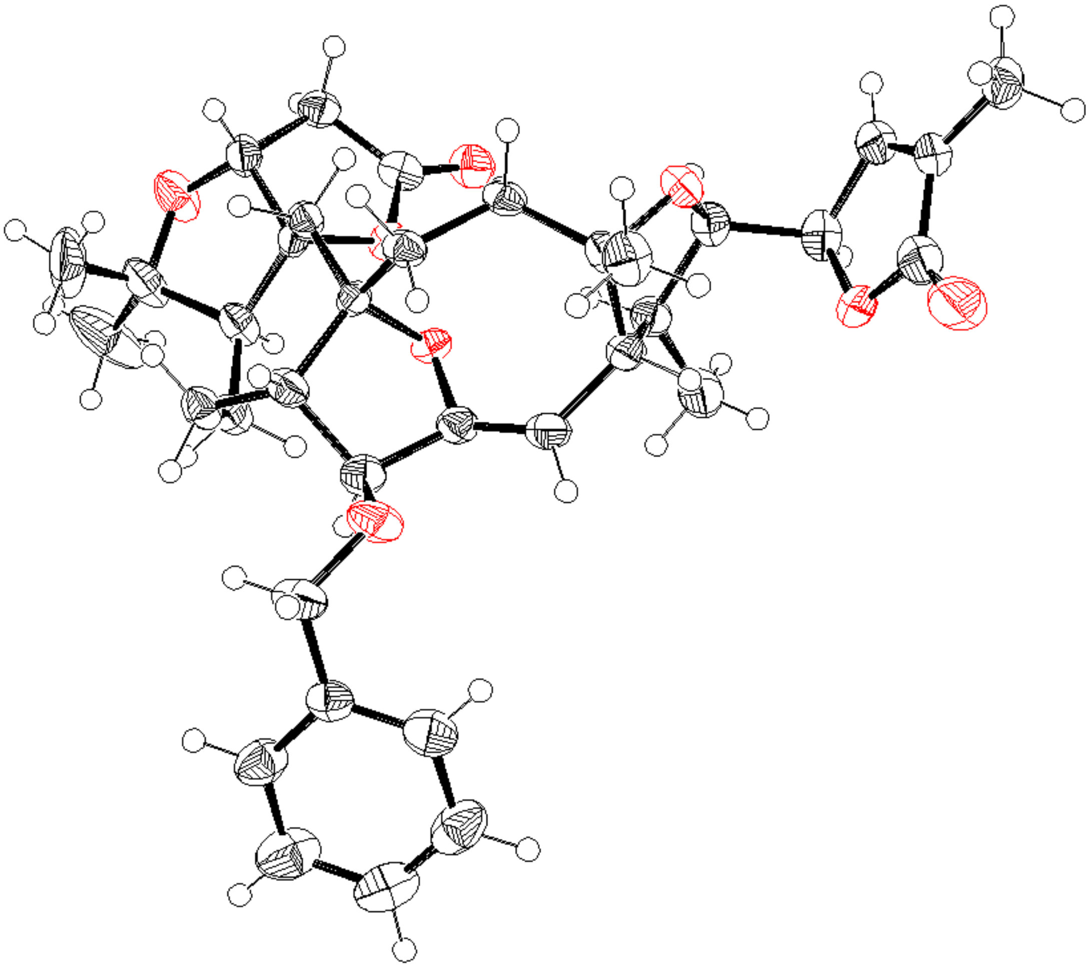

Supplementary Figure 39 | ORTEP of compound 29

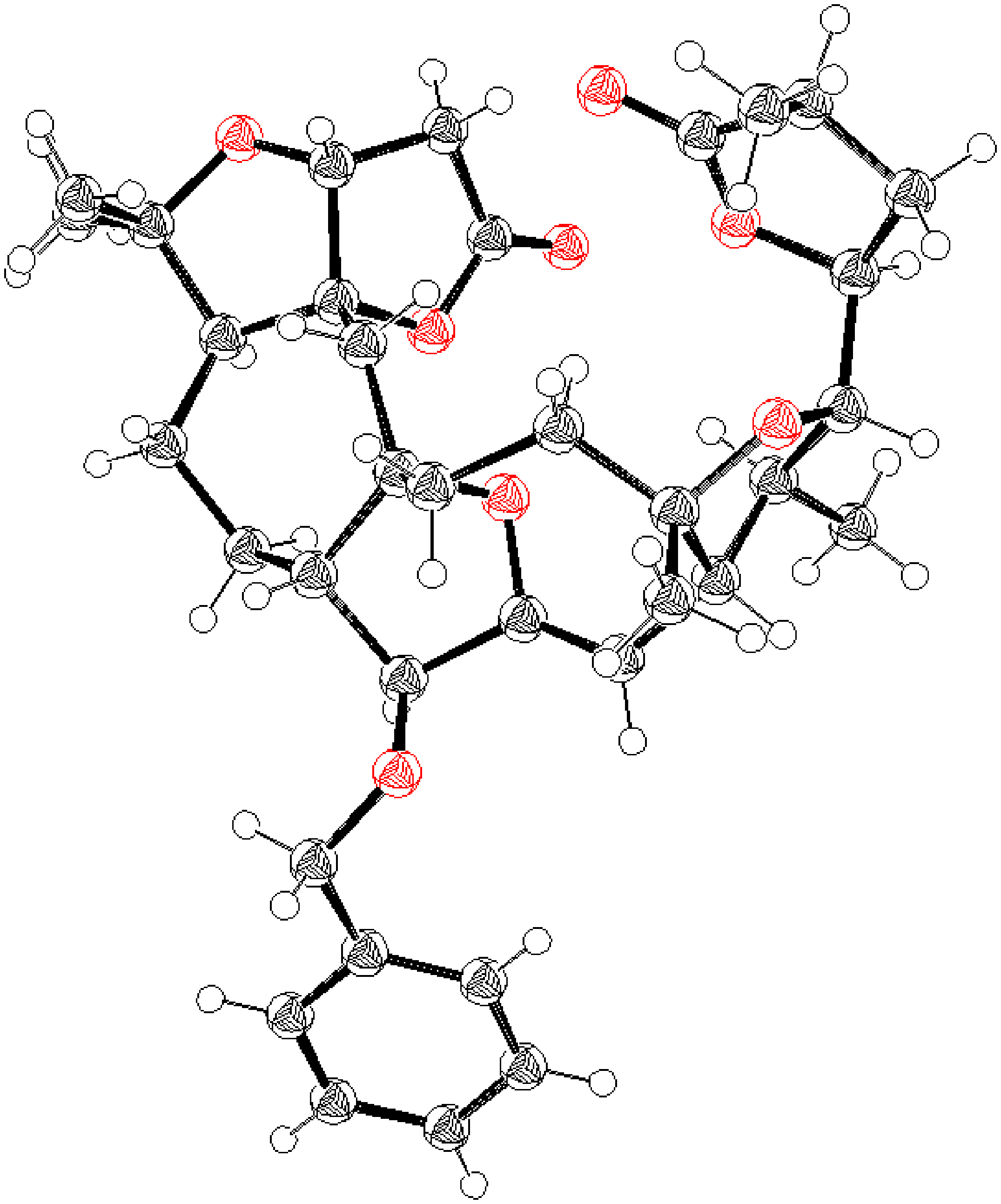

Supplementary Figure 40 | ORTEP of compound 31

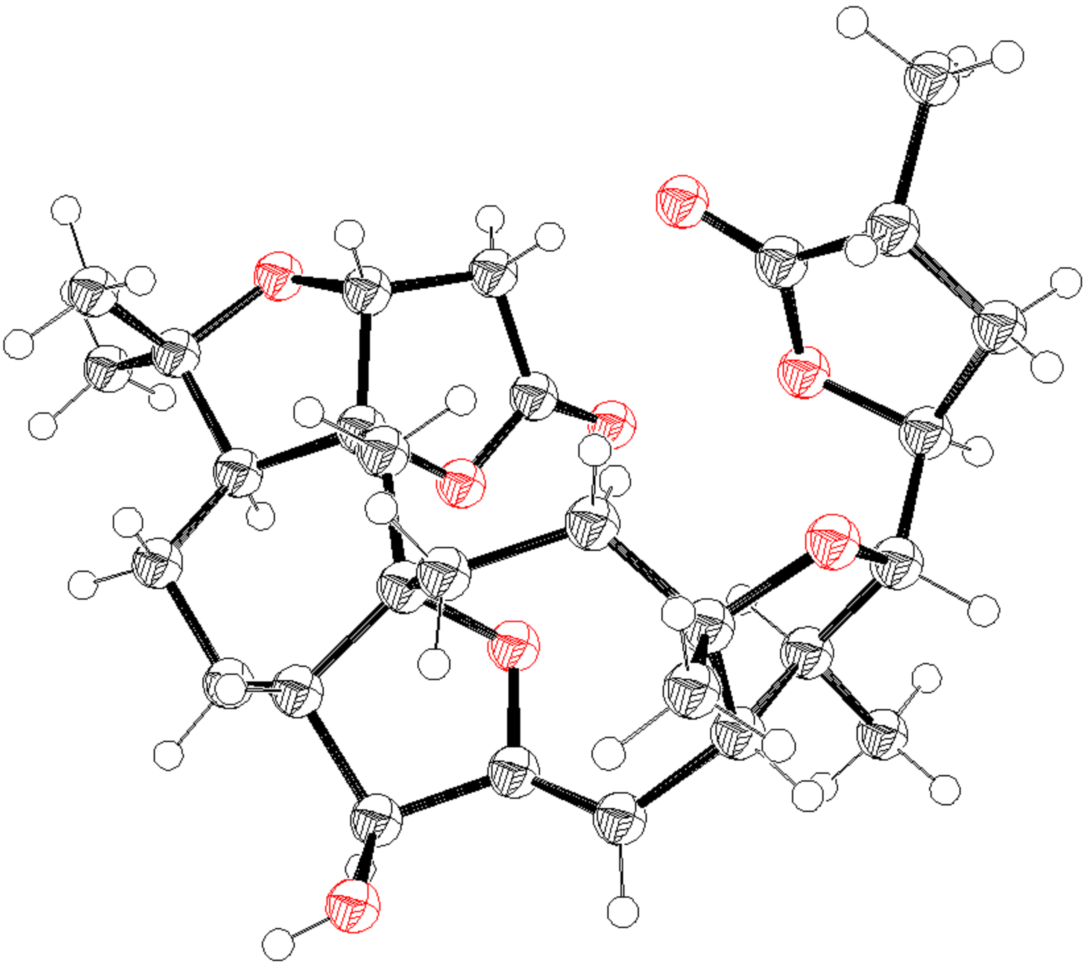

Supplementary Figure 41 | ORTEP of compound 32

General computational calculation details.

1. Completes reference for Gaussian 09

Frisch, M. J.; Trucks, G. W.; Schlegel, H. B.; Scuseria, G. E.; Robb, M. A.; Cheeseman, J. R.; Scalmani, G.; Barone, V.; Mennucci, B.; Petersson, G. A.; Nakatsuji, H.; Caricato, M.; Li, X.; Hratchian, H. P.; Izmaylov, A. F.; Bloino, J.; Zheng, G.; Sonnenberg, J. L.; Hada, M.; Ehara, M.; Toyota, K.; Fukuda, R.; Hasegawa, J.; Ishida, M.; Nakajima, T.; Honda, Y.; Kitao, O.; Nakai, H.; Vreven, T.; Montgomery, Jr., J. A.; Peralta, J. E.; Ogliaro, F.; Bearpark, M.; Heyd, J. J.; Brothers, E.; Kudin, K. N.; Staroverov, V. N.; Keith, T.; Kobayashi, R.; Normand, J.; Raghavachari, K.; Rendell, A.; Burant, J. C.; Iyengar, S. S.; Tomasi, J.; Cossi, M.; Rega, N.; Millam, J. M.; Klene, M.; Knox, J. E.; Cross, J. B.; Bakken, V.; Adamo, C.; Jaramillo, J.; Gomperts, R.; Stratmann, R. E.; Yazyev, O.; Austin, A. J.; Cammi, R.; Pomelli, C.; Ochterski, J. W.; Martin, R. L.; Morokuma, K.; Zakrzewski, V. G.; Voth, G. A.; Salvador, P.; Dannenberg, J. J.; Dapprich, S.; Daniels, A. D.; Farkas, O.; Foresman, J. B.; Ortiz, J. V.; Cioslowski, J.; and Fox, D. J. Gaussian 09, revision D.01; Gaussian, Inc.: Wallingford, CT, **2013**.

2. Computational Methods

All DFT calculations were carried out with the GAUSSIAN 09 series of programs. Density functional B3-LYP[2] with a standard 6-31G(d) basis set was used for geometry optimizations. Harmonic frequency calculations were performed for all stationary points to confirm them as local minima or transition structures and to derive the thermochemical corrections for the enthalpies and free energies. M06 functional was used to calculate the single point energies and provide highly accurate energy information.[3] The solvent effects were considered by single point calculations on the gas-phase stationary points with a continuum solvation model SMD.[4] The larger basis set 6-311+G(d) was used in the solvation single point calculations. The energies given in this report are the M06 calculated Gibbs free energies in solvent. Besides, the comparison of M06 with different methods was also considered, and these data could be found in Supplementary Figure 42-44.

3. B3-LYP and M06 absolute calculation energies, enthalpies, and free energies.

3.1 B3-LYP and M06 absolute calculation energies, enthalpies, and free energies for Fig. 10.

Geometry	$E_{(elec\text{-}B3\text{-}LYP)}$[a]	$H_{(corr\text{-}B3\text{-}LYP)}$[b]	$G_{(corr\text{-}B3\text{-}LYP)}$[c]	$E_{(solv\text{-}M06)}$[d]	IF[e]
**24**	-1656.646677	0.700071	0.600210	-1656.028403	-
$BH_3{\cdot}SMe_2$	-504.656214	0.116163	0.078547	-504.576112	-
**TS1**	-1683.272378	0.733599	0.632675	-1682.641249	-536.3
SMe_2	-478.009522	0.082156	0.049932	-477.949026	-
**CP1**	-1683.307325	0.735719	0.631470	-1682.668338	-

**TS2**	-1683.272035	0.733674	0.631933	-1682.638767	-453.7
**CP2**	-1683.308766	0.736020	0.632805	-1682.668951	-
**TS3**	-1656.635899	0.699360	0.602581	-1656.020276	-69.4
**CP3**	-1656.643152	0.700105	0.599823	-1656.023646	-
**TS4**	-1683.271912	0.733623	0.631812	-1682.638695	-453.6

[a] The electronic energy calculated by B3-LYP in gas phase. [b] The thermal correction to enthalpy calculated by B3-LYP in gas phase. [c] The thermal correction to Gibbs free energy calculated by B3-LYP in gas phase. [d] The electronic energy calculated by M06 in tetrahydrofuran solvent. [e] The B3-LYP calculated imaginary frequencies for the transition states.

3.2 B3-LYP and M06 absolute calculation energies, enthalpies, and free energies for Fig. 11.

Geometry	$E_{(elec\text{-}B3\text{-}LYP)}$[a]	$H_{(corr\text{-}B3\text{-}LYP)}$[b]	$G_{(corr\text{-}B3\text{-}LYP)}$[c]	$E_{(solv\text{-}M06)}$[d]	*IF*[e]
**CP4**	-2401.033719	0.839793	0.714249	-2400.336963	-
**TS5**	-2401.010418	0.836469	0.713037	-2400.320273	-39.1
**CP5**	-2401.010952	0.837481	0.711840	-2400.323778	-
**CP6**	-2401.026779	0.838623	0.709222	-2400.336505	-
**TS6**	-2400.986783	0.836755	0.709823	-2400.315159	-168.9
**CP7**	-2400.989532	0.838997	0.713271	-2400.319557	-
**CP8**	-2401.024633	0.839786	0.712067	-2400.340779	-
**TS7**	-2400.988170	0.836086	0.714274	-2400.300602	-351.4
**29**	-2000.050034	0.791362	0.677499	-1999.346658	-
$BF_3\cdot H_2O$	-400.975034	0.043641	0.008921	-400.998814	-

[a] The electronic energy calculated by B3-LYP in gas phase. [b] The thermal correction to enthalpy calculated by B3-LYP in gas phase. [c] The thermal correction to Gibbs free energy calculated by B3-LYP in gas phase. [d] The electronic energy calculated by M06 in dichloromethane solvent. [e] The B3-LYP calculated imaginary frequencies for the transition states.

3.3 B3-LYP and M06 absolute calculation energies, enthalpies, and free energies for Fig. 12.

Geometry	$E_{(elec\text{-}B3\text{-}LYP)}$[a]	$H_{(corr\text{-}B3\text{-}LYP)}$[b]	$G_{(corr\text{-}B3\text{-}LYP)}$[c]	$E_{(solv\text{-}M06)}$[d]	*IF*[e]
**29**	-2000.050034	0.791362	0.677499	-1999.342713	-
$BF_3 \cdot H_2O$	-400.975034	0.043641	0.008921	-400.998814	-
DBU	-462.078616	0.258055	0.212253	-461.861050	-
**TS8**	-2462.089801	1.047658	0.905515	-2461.174613	-179.0
$DBUH^+$	-462.497736	0.273068	0.226658	-462.338425	-
**CP9**	-1999.456111	0.775857	0.660309	-1998.830611	-
**TS9**	-2462.098639	1.049613	0.908877	-2461.169811	-201.5
**CP10**	-2462.122007	1.050485	0.903287	-2461.181511	-
**CP11**	-2462.131884	1.050581	0.907112	-2461.190966	-
**TS10**	-2462.110188	1.049906	0.910957	-2461.174570	-179.9
**CP12**	-1999.463232	0.775983	0.661692	-1998.832158	-
**TS11**	-1999.457115	0.774916	0.664073	-1998.824183	-57.7
**CP13**	-1999.484235	0.776493	0.663536	-1998.830537	-
**TS12**	-2462.108555	1.047069	0.908650	-2461.178332	-766.5
**30**	-2000.056236	0.791200	0.677116	-1999.345440	-
**TS13**	-2462.092825	1.046727	0.906315	-2461.172482	-729.1
**TS14**	-2462.095670	1.046437	0.907109	-2461.174851	-767.2
**CP14**	-2000.050848	0.790926	0.674999	-1999.339937	-

[a] The electronic energy calculated by B3-LYP in gas phase. [b] The thermal correction to enthalpy calculated by B3-LYP in gas phase. [c] The thermal correction to Gibbs free energy calculated by B3-LYP in gas phase. [d] The electronic energy calculated by M06 in tetrahydrofuran solvent. [e] The B3-LYP calculated imaginary frequencies for the transition states.

4. Benchmark of computational methods

In addition to M06 functional, other methods B3LYP-D3(BJ) and MP2 with basis set 6-311+G(d) were also employed for solvation single point calculations in THF based on gas-phase stationary points using the continuum solvation model SMD. As shown below, the free energy profiles in

Figure 10, 11 and 12 were recalculated, and the results were summarized in Supplementary Figure 42-44, respectively. Compared with the data in manuscript, the tendencies shown in Supplementary Figure 42-44 are almost the same as that obtained by M06. More importantly, the conclusions shown in Supplementary Figure 42-44 are consistent with that drawn in the manuscript. Consequently, the combination of M06 with B3LYP could give reliable results for this work.

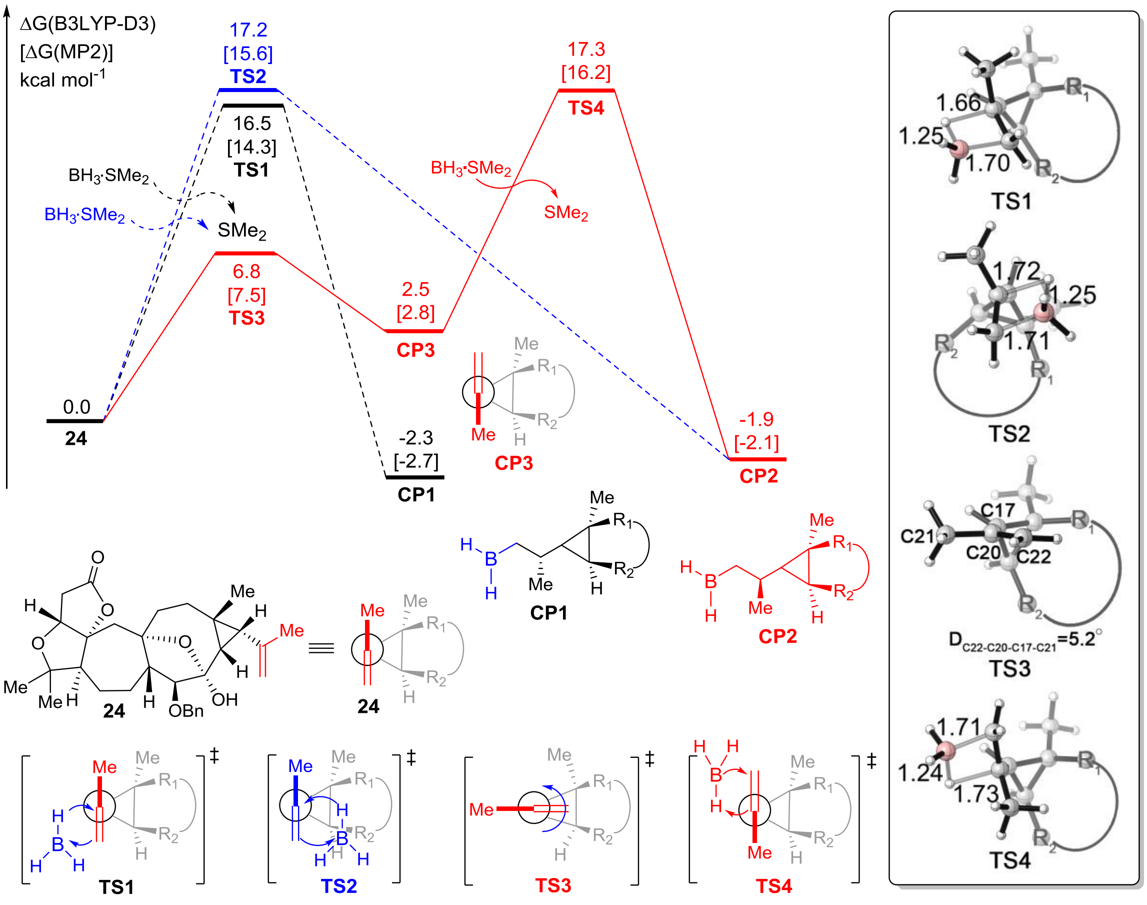

Supplementary Figure 42 | ** Energy profiles for the hydroboration of **24 calculated by B3LYP-D3(BJ)/6-311+G(d) and MP2/6-311+G(d) in THF.

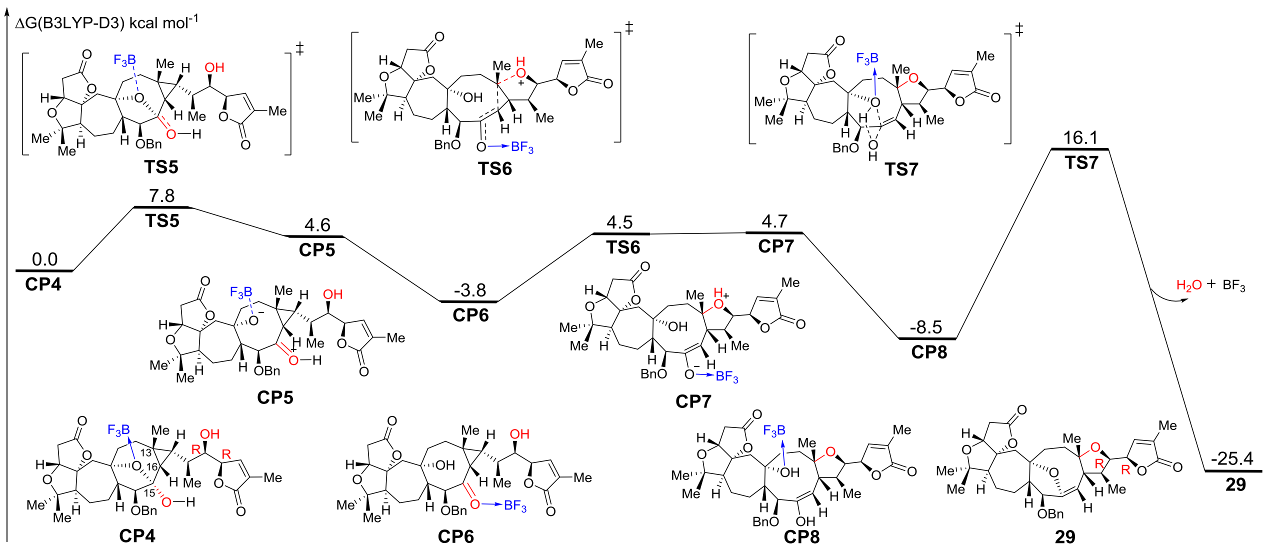

Supplementary Figure 43 | Energy profiles for the homo-Michael step calculated by MP2/6-311+G(d) in THF.

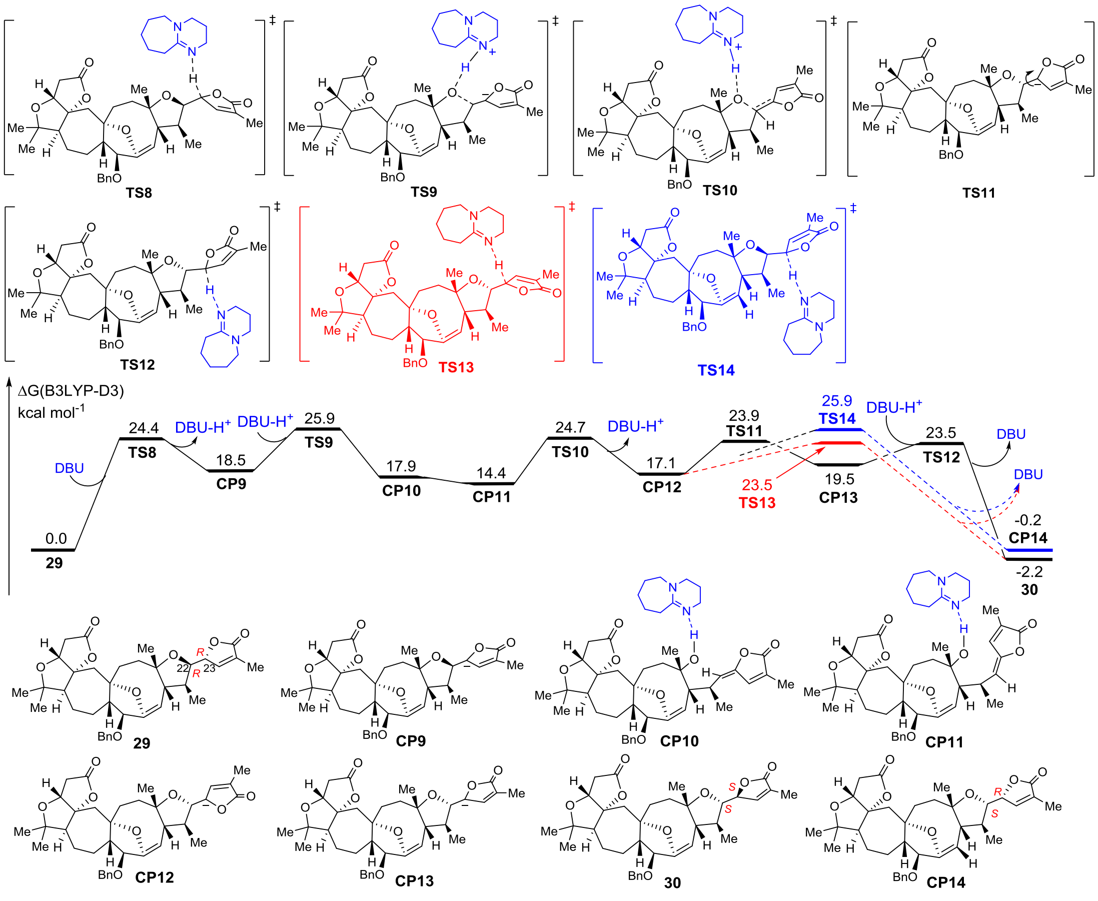

Supplementary Figure 44 | Energy profiles for the reversible retro-oxo Michael reaction of **29** calculated by MP2/6-311+G(d) in THF.

Supplementary References.

[1] Llera, J. M.; Fraser-Reid, B. *J. Org. Chem.* **1989**, *54*, 5544.
[2] (a) Lee, C.; Yang, W.; Parr, R. G. *Phys. Rev. B* **1988**, *37*, 785−789. (b) Becke, A. D. *J. Chem. Phys.* **1993**, *98*, 5648−5652.
[3] (a) Zhao, Y.; Truhlar, D. G. *Theor. Chem. Acc.* **2008**, *120*, 215−241. (b) Zhao, Y.; Truhlar, D. G. *J. Phys. Chem. C* **2008**, *112*, 6860−6868.
[4] (a) Cossi, M.; Barone, V.; Cammi, R.; Tomasi, J. *Chem. Phys. Lett.* **1996**, *255*, 327−335. (b) Cancès, E.; Mennunci, B.; Tomasi, J. *J. Chem. Phys.* **1997**, *107*, 3032−3041. (c) Barone, V.; Cossi, M.; Tomasi, J. *J. Comput. Chem.* **1998**, *19*, 404−417.
