# Supplementary material for: Biomimetically inspired asymmetric total synthesis of (+)-19-dehydroxyl arisandilactone A
Source: Nat Commun. 2017 Jan 31;8:14233. doi: 10.1038/ncomms14233 (PMC5290315; doi:10.1038/ncomms14233)
Supplement: Supplementary Data 1 — All DFT calculations were carried out with the GAUSSIAN 09 series of programs. Density functional B3-LYP with a standard 6-31G(d) basis set was used for geometry optimizations. Harmonic frequency calculations were performed for all stationary points to confirm them as local minima or transition structures and to derive the thermochemical corrections for the enthalpies and free energies. All the structural coordinates given in this manuscript are obtained at B3-LYP/6-31G(d) level of theory. [file ncomms14233-s2.docx]

**B3-LYP geometries for all the optimized compounds and transition states.**

**24**

C 0.98753300 -1.20881400 -0.87044800

C 2.13967200 -1.84147300 -1.70279300

C 1.72859900 0.61375800 0.43569900

C 3.53039400 -1.38150800 -1.21866700

C 3.04190800 1.03493500 -0.21029000

C 3.81719900 0.12656300 -1.16449100

H 1.98553100 -1.61953200 -2.76704300

H 3.68294900 -1.80817000 -0.22502600

H 2.11335100 -2.93612900 -1.61270500

H 4.28354200 -1.83843100 -1.87619300

H 2.96382300 2.06050700 -0.56393000

C 0.47328600 0.10936100 -1.54914400

H 1.27295800 0.44038100 -2.21955100

C 0.51473600 1.11964600 -0.40183700

O 1.53471100 -0.79778900 0.40950100

O 1.68013400 1.04203100 1.77285800

H 0.88669200 0.63573700 2.17232200

C 4.47082400 0.65299800 0.12096100

H 5.16207500 1.47798600 -0.07163000

C 4.26641900 0.72373400 -2.49007700

H 3.49769900 0.61252200 -3.26708600

H 4.48112000 1.79413800 -2.39524400

H 5.17632300 0.22960900 -2.85597200

C 5.01157000 -0.24944700 1.18770200

C 6.40233600 -0.76746600 0.88774400

H 7.10442800 0.05995800 0.71271700

H 6.79245800 -1.37389400 1.71132300

H 6.41147000 -1.38091400 -0.02360100

H -0.37416400 1.04326900 0.23168000

C -0.05655100 -2.28357300 -0.50257300

C -1.37326300 -1.82257600 0.13298300

C -2.40678600 -1.15808300 -0.81378900

C -0.82021700 0.14534600 -2.41203600

C -1.91983600 -0.92173100 -2.24599900

H -0.29867500 -2.89095700 -1.38207500

H 0.45558300 -2.95594300 0.19865800

H -2.64292900 -0.18514200 -0.36890600

O 0.62737500 2.43257200 -0.92322600

C 0.25827700 3.47113000 -0.00095500

H 0.63295400 4.39023100 -0.46351800

H 0.77582500 3.33195400 0.95485400

C -1.23924100 3.56023500 0.21167400

C -1.81423900 3.25843900 1.45152800

C -2.07848900 3.93127300 -0.84984200

C -3.19878300 3.32824800 1.63221700

H -1.17457500 2.96320700 2.28002000

C -3.45962300 4.00207800 -0.67400300

H -1.63950600 4.16519100 -1.81707600

C -4.02339300 3.70020800 0.57005900

H -3.62929000 3.09032600 2.60126700

H -4.09755200 4.29793300 -1.50305200

H -5.09977900 3.75875400 0.70918500

C -3.68628700 -2.04951400 -0.66165600

C -3.80885000 -3.16291200 -1.71496300

H -3.99862900 -2.75400800 -2.71261600

H -2.90619000 -3.78104000 -1.77401400

H -4.64555900 -3.81638500 -1.44792600

C -4.96795900 -1.22115200 -0.60376200

H -5.12372300 -0.68382800 -1.54600600

H -5.83615600 -1.86575700 -0.42992700

H -4.91119400 -0.49024200 0.20890400

O -3.52676100 -2.65231500 0.64416100

C -2.15874900 -2.97397200 0.82479800

H -1.91722700 -3.95334800 0.38839700

C -1.78664500 -2.87157200 2.29882800

H -2.64563200 -3.02278700 2.95698600

H -1.00840500 -3.58689900 2.58756200

C -1.23604500 -1.45934300 2.44906600

O -1.10357800 -0.89688600 1.22396300

O -0.92794900 -0.86328100 3.45205700

H -1.59033600 -1.86521400 -2.69592500

H -2.77435600 -0.59411900 -2.85356800

H -0.52919100 0.12485800 -3.46960600

H -1.26322600 1.13712500 -2.25522400

C 4.37802800 -0.56137000 2.32377300

H 4.84864400 -1.21187400 3.05863600

H 3.39245500 -0.17441200 2.55216600

BH_3_·SMe_2_

H -1.47524400 1.62295300 0.00000000

B -0.28268300 1.83034700 0.00000000

H 0.15828300 2.31694100 1.01439500

H 0.15828300 2.31694100 -1.01439500

S 0.53568100 0.01538700 0.00000000

C -0.28268300 -0.82026100 -1.39713400

H -1.36430900 -0.67988700 -1.32530700

H 0.08451200 -0.34403000 -2.30869500

H -0.02351100 -1.88190200 -1.40513100

C -0.28268300 -0.82026100 1.39713400

H -0.02351100 -1.88190200 1.40513100

H 0.08451200 -0.34403000 2.30869500

H -1.36430900 -0.67988700 1.32530700

**TS1**

C 0.73093100 -1.35758500 -0.86929900

C 1.83528800 -2.13099900 -1.64874700

C 1.58739500 0.58349200 0.17701000

C 3.25588200 -1.68073800 -1.24705200

C 2.88467200 0.86796400 -0.57279600

C 3.60050200 -0.19117700 -1.40388100

H 1.66628300 -2.02555100 -2.72816500

H 3.40707700 -1.98776900 -0.21136600

H 1.76704900 -3.20604000 -1.43391900

H 3.97478100 -2.25165500 -1.85118100

H 2.81427600 1.83283200 -1.06911400

C 0.23837800 -0.11179600 -1.68695700

H 1.02129200 0.10197100 -2.42208300

C 0.36001600 1.03011700 -0.67484800

O 1.33805000 -0.81362200 0.33108600

O 1.62781400 1.16434800 1.44846200

H 0.83633000 0.85971400 1.93283000

C 4.32246200 0.51237600 -0.24684600

H 5.01091200 1.28193700 -0.59658300

C 4.02347600 0.19186600 -2.81598500

H 3.22903400 -0.02022800 -3.54363700

H 4.25783800 1.25943300 -2.89028900

H 4.91321000 -0.37106500 -3.12719200

C 4.92634100 -0.24023100 0.91220900

C 6.24471100 -0.91128500 0.55345200

H 6.90718100 -0.23909200 -0.00235300

H 6.76662700 -1.25238100 1.45131200

H 6.03791700 -1.78337000 -0.07908800

H -0.50414100 1.06597200 -0.00494900

C -0.33455000 -2.33951900 -0.34029800

C -1.61193200 -1.75583400 0.27489200

C -2.65745000 -1.17461600 -0.71212400

C -1.08726300 -0.12619100 -2.50110200

C -2.21479500 -1.12558000 -2.17657400

H -0.62492000 -3.03993700 -1.13155800

H 0.17872300 -2.94070700 0.42216600

H -2.85021800 -0.14891200 -0.37903400

O 0.49413800 2.25989400 -1.36357900

C 0.29398700 3.42969400 -0.55012300

H 0.60743200 4.25799300 -1.19389900

H 0.95539200 3.40236400 0.32304100

C -1.14528900 3.61315800 -0.11533000

C -1.50705500 3.52272300 1.23383600

C -2.14419600 3.86310800 -1.06840800

C -2.83856600 3.68250000 1.62752400

H -0.74154900 3.32200700 1.97953400

C -3.47384300 4.02101600 -0.67983700

H -1.87146800 3.93600200 -2.11888500

C -3.82378400 3.93126100 0.67146000

H -3.10308100 3.60773400 2.67881200

H -4.23727800 4.22136400 -1.42740800

H -4.85956400 4.05803400 0.97555400

C -3.95573800 -1.99977600 -0.41672400

C -4.14361100 -3.22749800 -1.32407300

H -4.35808700 -2.93553200 -2.35725700

H -3.25984900 -3.87511000 -1.33811800

H -4.98676400 -3.81967500 -0.95407600

C -5.21106100 -1.13014400 -0.42425100

H -5.37810400 -0.70133100 -1.41881400

H -6.09211700 -1.72213300 -0.15483200

H -5.10994000 -0.31263600 0.29632300

O -3.77129800 -2.44485800 0.94739000

C -2.40819100 -2.78678400 1.12709400

H -2.21059200 -3.81996200 0.80872300

C -1.98388400 -2.51707300 2.56529900

H -2.82586800 -2.55127600 3.26089700

H -1.22365300 -3.22187200 2.92023800

C -1.37783700 -1.12001800 2.52347000

O -1.27057800 -0.71623800 1.23346300

O -1.01113100 -0.42006900 3.43457100

H -1.93611500 -2.12794100 -2.52097400

H -3.07889200 -0.83862200 -2.79097600

H -0.83931200 -0.27477800 -3.55929400

H -1.49029200 0.89282000 -2.44115100

C 4.19471600 -0.75557400 2.02071800

H 3.12177500 -0.62407600 2.04837200

H 5.62183600 1.10295000 1.59833300

H 4.57016000 -1.66461600 2.48348300

B 5.01103600 0.62742100 2.58038200

H 5.91225000 0.29781200 3.30355800

H 4.21503000 1.48142500 2.83968800

SMe_2_

S 0.00000000 0.00000000 0.66366400

C 0.00000000 1.39391900 -0.51529100

H -0.89465200 1.38095600 -1.14621100

H 0.00000000 2.31437800 0.07486100

H 0.89465200 1.38095600 -1.14621100

C 0.00000000 -1.39391900 -0.51529100

H 0.89465200 -1.38095600 -1.14621100

H 0.00000000 -2.31437800 0.07486100

H -0.89465200 -1.38095600 -1.14621100

**CP1**

C 0.74585900 -1.25503500 -0.97844300

C 1.85670900 -1.94105600 -1.82886900

C 1.53274000 0.69209700 0.10399100

C 3.27000100 -1.47354900 -1.42499300

C 2.79107000 1.05997200 -0.66982200

C 3.57721700 0.03220600 -1.48906700

H 1.66438100 -1.76665700 -2.89536100

H 3.45288000 -1.85024200 -0.41791100

H 1.82292900 -3.03006700 -1.68804700

H 3.99101600 -1.98382000 -2.07924300

H 2.67759300 2.02376900 -1.16042900

C 0.15672900 -0.00939200 -1.72859100

H 0.90412900 0.27823900 -2.47598600

C 0.24903500 1.09836200 -0.67679300

O 1.36714700 -0.72269500 0.22022500

O 1.58903700 1.22493600 1.40132600

H 0.84613400 0.83739500 1.90270700

C 4.21190100 0.70912800 -0.27988500

H 4.93083600 1.48881400 -0.54388300

C 4.04022500 0.49060300 -2.86647800

H 3.27729500 0.29960700 -3.63370200

H 4.25601900 1.56476600 -2.88050500

H 4.95335500 -0.03793500 -3.17105000

C 4.61234100 0.00090700 1.01095700

C 4.77984100 1.02694800 2.14575700

H 5.05205000 0.52671400 3.08430800

H 5.57722200 1.74481200 1.90904900

H 3.85014100 1.57761400 2.30606900

H -0.58997600 1.06108100 0.02479200

C -0.24656100 -2.31095100 -0.44894000

C -1.52838200 -1.82044800 0.23410600

C -2.63817700 -1.25351100 -0.68990000

C -1.18969700 -0.07152400 -2.50244400

C -2.25701300 -1.13190000 -2.16780700

H -0.53146000 -2.99633200 -1.25510900

H 0.32592200 -2.91217100 0.27034800

H -2.86528900 -0.25082400 -0.31135300

O 0.28793100 2.35886000 -1.32318200

C 0.05915800 3.48189500 -0.45712300

H 0.31937100 4.35095100 -1.07062600

H 0.74253200 3.44797700 0.39880500

C -1.37577200 3.58349000 0.01857800

C -1.69720900 3.45861300 1.37498700

C -2.41056700 3.79534800 -0.90482500

C -3.02391400 3.54971900 1.80533400

H -0.90346000 3.28389800 2.09748200

C -3.73539000 3.88451500 -0.48003700

H -2.16900100 3.89228200 -1.96086000

C -4.04491100 3.76264100 0.87866900

H -3.25684300 3.44891400 2.86197800

H -4.52717300 4.05586500 -1.20502100

H -5.07716700 3.83568700 1.21111700

C -3.88398200 -2.15187800 -0.37876000

C -4.05149800 -3.34825000 -1.33043700

H -4.32060900 -3.02417200 -2.34095200

H -3.13940900 -3.95052200 -1.40635900

H -4.85041200 -3.99540200 -0.95447500

C -5.17764200 -1.34433700 -0.29806600

H -5.40669200 -0.88337700 -1.26550100

H -6.01789700 -1.98896200 -0.01902100

H -5.08438900 -0.55314900 0.45237100

O -3.62299300 -2.64444000 0.95644000

C -2.23890600 -2.92580300 1.06780500

H -2.00491200 -3.93304200 0.69535600

C -1.76992800 -2.69839900 2.49936100

H -2.57950600 -2.80791300 3.22501400

H -0.96045600 -3.37715100 2.79045500

C -1.23799500 -1.27069200 2.49830800

O -1.20614400 -0.80487700 1.22609400

O -0.86962500 -0.59435400 3.42693500

H -1.94787600 -2.10811600 -2.55843300

H -3.15755600 -0.86524400 -2.73764800

H -0.96835100 -0.18515600 -3.57092200

H -1.63940800 0.92497300 -2.40687000

C 5.92098400 -0.79958300 0.82570200

H 5.82523100 -1.48733000 -0.04145500

H 6.74330800 -0.13011800 0.52756800

B 6.35619100 -1.77951600 1.95753900

H 5.57037500 -2.19640200 2.76296100

H 7.47796600 -2.20240800 1.99660600

H 3.81578300 -0.68951100 1.30968600

**TS2**

C 0.72968000 -1.35272400 -0.95577300

C 1.80899300 -2.12016300 -1.77747100

C 1.61872300 0.57402300 0.07949900

C 3.25046700 -1.74892100 -1.36634700

C 2.88869900 0.84334400 -0.71495300

C 3.64722700 -0.26352700 -1.43809900

H 1.64290700 -1.94843100 -2.84882700

H 3.43319700 -2.14991600 -0.37418600

H 1.70253000 -3.20238200 -1.62420200

H 3.93638000 -2.29464100 -2.02710700

H 2.82197300 1.78462000 -1.25504600

C 0.21330400 -0.09500600 -1.74062900

H 0.97778800 0.13158000 -2.49195800

C 0.36172200 1.03282300 -0.71509400

O 1.37151100 -0.82232300 0.23411500

O 1.72441000 1.14578700 1.35601000

H 0.97599400 0.80895800 1.88545600

C 4.29879600 0.47462500 -0.27055400

H 5.03157100 1.20356200 -0.61651100

C 4.19830100 0.11450100 -2.81076300

H 3.47162500 -0.11328100 -3.60187200

H 4.43174400 1.18288300 -2.87757200

H 5.11536100 -0.44636600 -3.02979100

C 4.64718500 0.05816100 1.13565000

C 5.16548400 1.22085300 1.96436200

H 4.30638300 1.86159600 2.19550700

H 5.60281100 0.86915700 2.90234000

H 5.91241000 1.81562300 1.42838700

H -0.47917100 1.05715400 -0.01651000

C -0.32398100 -2.33544200 -0.40447100

C -1.57565200 -1.75018800 0.26054000

C -2.64922400 -1.14749400 -0.68241600

C -1.13185300 -0.10269500 -2.52018200

C -2.25874700 -1.09183500 -2.16170200

H -0.64559200 -3.02276900 -1.19485800

H 0.20912300 -2.95139300 0.33224900

H -2.81576900 -0.12190800 -0.33513800

O 0.47412400 2.27412300 -1.38826900

C 0.27958100 3.42789100 -0.55273600

H 0.57953500 4.26955900 -1.18561800

H 0.95313400 3.38884000 0.31081900

C -1.15434400 3.59605100 -0.09399900

C -1.49600300 3.48581700 1.25889900

C -2.16872600 3.85142500 -1.02904500

C -2.82242100 3.63240700 1.67429800

H -0.71882500 3.27843800 1.99067400

C -3.49339000 3.99618700 -0.61902800

H -1.91194400 3.93871700 -2.08237000

C -3.82302300 3.88750000 0.73602000

H -3.07115400 3.54245100 2.72827400

H -4.26894300 4.20087200 -1.35275900

H -4.85493100 4.00410500 1.05679600

C -3.94621900 -1.95822100 -0.34403900

C -4.18628200 -3.17390800 -1.25504000

H -4.43417800 -2.86881200 -2.27687300

H -3.31306700 -3.83366800 -1.30704400

H -5.02384400 -3.75721500 -0.85930100

C -5.18854800 -1.07175100 -0.29230900

H -5.39026600 -0.63164600 -1.27542900

H -6.06585800 -1.65435100 0.00800300

H -5.04705500 -0.26202000 0.43030400

O -3.71418200 -2.42077800 1.00747100

C -2.35013700 -2.78373100 1.12857600

H -2.17884400 -3.81461100 0.78829600

C -1.86454200 -2.54095300 2.55272000

H -2.67867400 -2.57346300 3.28091100

H -1.10138500 -3.26167300 2.86698200

C -1.24126000 -1.15159500 2.50556800

O -1.19443500 -0.72515000 1.22060900

O -0.81861900 -0.47302500 3.40960400

H -2.00325200 -2.09420300 -2.52341600

H -3.14082000 -0.79103300 -2.74304800

H -0.91251300 -0.25814700 -3.58368700

H -1.52440700 0.92002300 -2.45420600

C 4.14581400 -1.06157200 1.84935000

H 4.08757300 -0.95394000 2.92888600

H 3.34317800 -1.64819700 1.42666900

B 5.71311700 -1.51426900 1.34376100

H 6.42791400 -1.59321200 2.30674800

H 6.19976900 -0.55842700 0.70888800

H 5.63177900 -2.38547500 0.52101300

**CP2**

C 0.66455000 -1.42366000 -0.94070100

C 1.70764200 -2.24268600 -1.75793700

C 1.60700600 0.52964400 -0.00471100

C 3.15912700 -1.85087600 -1.40988200

C 2.87400200 0.75468700 -0.81688500

C 3.57085100 -0.37873800 -1.57632500

H 1.50490000 -2.12895000 -2.83070800

H 3.33830800 -2.17561500 -0.38433000

H 1.60134700 -3.31443000 -1.54103500

H 3.82857000 -2.45161700 -2.04129100

H 2.82170800 1.69031000 -1.36858000

C 0.15135800 -0.19102500 -1.76616900

H 0.90297700 -0.00890300 -2.54186500

C 0.34200700 0.97535200 -0.79328600

O 1.34534300 -0.85753700 0.20964800

O 1.72348000 1.14259200 1.25530800

H 0.96467500 0.84173000 1.79171600

C 4.27194400 0.32649400 -0.42196100

H 5.04035800 1.03438500 -0.74296500

C 4.04210700 -0.04418300 -2.98624200

H 3.25555300 -0.23098300 -3.73029400

H 4.33135200 1.00905900 -3.07391800

H 4.91156600 -0.65397400 -3.26561200

C 4.63809800 -0.31883400 0.91112300

C 5.88896300 -1.20134800 0.75223400

H 6.74798600 -0.60241800 0.42108100

H 6.16073100 -1.67200000 1.70545900

H 5.73762000 -1.99778700 0.01492000

H -0.48544200 1.04639200 -0.08121600

C -0.38910300 -2.36748500 -0.32426400

C -1.61929300 -1.73878200 0.34037000

C -2.70385500 -1.16213200 -0.60645700

C -1.21251100 -0.20585200 -2.51240400

C -2.34060800 -1.16916600 -2.09397000

H -0.73599100 -3.08581800 -1.07558600

H 0.15369400 -2.95557900 0.42802700

H -2.85179800 -0.12165100 -0.29720700

O 0.45898200 2.18361400 -1.52420500

C 0.32458600 3.37660400 -0.73473400

H 0.62394900 4.18185300 -1.41373800

H 1.02622000 3.35712400 0.10683700

C -1.08776500 3.60510300 -0.23597300

C -1.38658700 3.55586300 1.13053900

C -2.12571800 3.85445800 -1.14632200

C -2.69331100 3.75792700 1.58342400

H -0.59115200 3.35226900 1.84350200

C -3.43095100 4.05465500 -0.69889600

H -1.90254200 3.89274300 -2.21018100

C -3.71746100 4.00758900 0.66940600

H -2.90849200 3.71483500 2.64771200

H -4.22486900 4.25416900 -1.41426300

H -4.73416600 4.16723600 1.01899700

C -4.00354600 -1.94173100 -0.21141700

C -4.27544900 -3.19066400 -1.06657800

H -4.53769200 -2.92476300 -2.09570000

H -3.41190800 -3.86386300 -1.10622000

H -5.11321300 -3.74534400 -0.63199200

C -5.23411700 -1.03828900 -0.17426200

H -5.44666400 -0.63445900 -1.17058900

H -6.11335800 -1.59738300 0.16298300

H -5.07088300 -0.20287100 0.51362400

O -3.75198800 -2.35149800 1.15349000

C -2.38977200 -2.72488100 1.26533000

H -2.23608200 -3.77113800 0.96623400

C -1.87607000 -2.42794200 2.66906900

H -2.67725100 -2.41927700 3.41213100

H -1.11561300 -3.14385500 3.00037200

C -1.23735600 -1.04978800 2.55174700

O -1.20559200 -0.68003300 1.24908700

O -0.79318000 -0.33833900 3.41938200

H -2.09890100 -2.18702100 -2.42054800

H -3.23120800 -0.88425600 -2.67057000

H -1.02209000 -0.39855800 -3.57544100

H -1.59155400 0.82330100 -2.47060300

C 4.87456700 0.75747200 1.99281600

H 5.67976900 1.44682200 1.69350400

H 3.97030100 1.39765200 2.04439400

B 5.01026500 0.30195700 3.47302800

H 4.61861600 -0.77193300 3.84029000

H 3.80634800 -0.94405700 1.25409500

H 5.44661600 1.05174000 4.30167600

**TS3**

C 0.81905000 -1.44614500 -0.87528400

C 1.87167300 -2.28152400 -1.66784900

C 1.79583600 0.42892900 0.17266100

C 3.31478900 -2.01461800 -1.19250400

C 3.11102300 0.60237000 -0.57214600

C 3.82421700 -0.56477300 -1.24585200

H 1.76278000 -2.08607100 -2.74228800

H 3.40517600 -2.41517600 -0.18773800

H 1.68211400 -3.35539300 -1.53458200

H 3.98951800 -2.61832900 -1.81469300

H 3.13479800 1.53756400 -1.12631600

C 0.40019800 -0.16710700 -1.68429300

H 1.19388600 0.00285800 -2.41994200

C 0.59779400 0.95899500 -0.66746000

O 1.45622600 -0.94953300 0.32965600

O 1.88678200 1.01240400 1.44306800

H 1.11024100 0.71205300 1.95239900

C 4.46147200 0.13106100 -0.04206200

H 5.25458300 0.81011900 -0.35874800

C 4.46957600 -0.23821300 -2.59033000

H 3.76672800 -0.40655600 -3.41749100

H 4.79362300 0.80698700 -2.64362300

H 5.34783000 -0.87233300 -2.76768700

C 4.72354300 -0.30043000 1.37536300

C 5.48192000 0.76334800 2.14414300

H 4.89935300 1.69347600 2.17414200

H 5.67982900 0.45235900 3.17435700

H 6.44297500 0.99985900 1.66632000

H -0.26062900 1.05266700 0.00450600

C -0.30382600 -2.36512900 -0.35149100

C -1.53917900 -1.70923700 0.27675700

C -2.54999300 -1.05542400 -0.70121600

C -0.92608700 -0.09627900 -2.49420300

C -2.11241800 -1.02495600 -2.16806500

H -0.64292200 -3.03671700 -1.14834400

H 0.17408100 -3.00621100 0.40149700

H -2.67270700 -0.02151800 -0.36070200

O 0.81514300 2.18023700 -1.35345300

C 0.68677600 3.35598300 -0.53701200

H 1.08259700 4.16231000 -1.16333800

H 1.31781700 3.26973500 0.35445600

C -0.74613100 3.64986100 -0.14262500

C -1.16092400 3.55553000 1.19080400

C -1.68752600 4.00556600 -1.12012300

C -2.48727000 3.81694500 1.54581200

H -0.44110600 3.26993100 1.95413000

C -3.01176900 4.26636700 -0.77035600

H -1.37364400 4.07850600 -2.15900500

C -3.41438400 4.17357700 0.56598600

H -2.79340500 3.73794700 2.58549800

H -3.72969500 4.54828900 -1.53649500

H -4.44569900 4.38054100 0.83984100

C -3.89750600 -1.79551400 -0.40273400

C -4.17129800 -3.00219400 -1.31639600

H -4.37076700 -2.69067700 -2.34685500

H -3.33196200 -3.70617900 -1.33729900

H -5.04998200 -3.53902400 -0.94473200

C -5.09340000 -0.84572300 -0.39514300

H -5.23704600 -0.39747600 -1.38480800

H -6.00997500 -1.38133300 -0.12595100

H -4.93609800 -0.04303000 0.33200300

O -3.73412800 -2.26341100 0.95679700

C -2.39491400 -2.69516300 1.12377500

H -2.26649000 -3.73578100 0.79422600

C -1.94512500 -2.46764400 2.56147500

H -2.78224900 -2.45880800 3.26367400

H -1.22572200 -3.22072200 2.90207000

C -1.25654700 -1.10890300 2.52879100

O -1.13214900 -0.70042800 1.24291100

O -0.84552400 -0.44137200 3.44582500

H -1.89730500 -2.03997400 -2.52062500

H -2.96060300 -0.68138000 -2.77564100

H -0.69177000 -0.25799600 -3.55366600

H -1.26478400 0.94575600 -2.43115000

C 4.36760400 -1.42933800 1.99960000

H 4.63515100 -1.57754400 3.04295300

H 3.77762100 -2.21721900 1.55310300

**CP3**

C 0.98891200 -1.19631400 -0.91679100

C 2.12549800 -1.81579300 -1.77987800

C 1.75655800 0.59749500 0.42322800

C 3.52787300 -1.37397600 -1.31218100

C 3.06414000 1.02146500 -0.22951800

C 3.81677600 0.13162000 -1.21182000

H 1.95539900 -1.56787700 -2.83577600

H 3.71787000 -1.84939400 -0.34889600

H 2.09423400 -2.91211600 -1.71572500

H 4.26618000 -1.80262400 -2.00381700

H 2.99072600 2.05692400 -0.55398500

C 0.47332100 0.13978200 -1.55778900

H 1.26614800 0.48191200 -2.23076800

C 0.53500800 1.12452700 -0.38921500

O 1.55474700 -0.81588700 0.36550700

O 1.73131600 0.99894800 1.76623600

H 0.94718400 0.58493200 2.17514800

C 4.49829500 0.61879600 0.07451500

H 5.18281500 1.44669200 -0.10903500

C 4.26305700 0.76000200 -2.52467400

H 3.49398400 0.66214000 -3.30336700

H 4.47271100 1.82900300 -2.40671400

H 5.17486000 0.27769500 -2.90108500

C 5.05839700 -0.30168600 1.13128400

C 4.20525700 -0.81503700 2.26499600

H 3.34892800 -1.39305400 1.90335900

H 4.79814400 -1.44444400 2.93703200

H 3.77996000 0.01734700 2.83491100

H -0.34606300 1.03972400 0.25416300

C -0.05636200 -2.27359800 -0.55989700

C -1.36148000 -1.82080200 0.10495300

C -2.40390800 -1.12967600 -0.81269200

C -0.83092800 0.20086300 -2.40290800

C -1.93427700 -0.86364700 -2.24555500

H -0.31431400 -2.85719000 -1.45075500

H 0.46108100 -2.96674000 0.11689700

H -2.62954600 -0.16612400 -0.34266400

O 0.64949700 2.44766500 -0.88351200

C 0.28283900 3.46713400 0.06053100

H 0.66679800 4.39414500 -0.37809000

H 0.79396100 3.30181600 1.01559400

C -1.21538600 3.56151900 0.26603900

C -1.79883600 3.25092200 1.49964900

C -2.04668700 3.94785000 -0.79635600

C -3.18397900 3.32715200 1.67354500

H -1.16544800 2.94412700 2.32879000

C -3.42831400 4.02482900 -0.62734500

H -1.60098800 4.18858200 -1.75880500

C -4.00063700 3.71415700 0.61065700

H -3.62119300 3.08256900 2.63793100

H -4.05996100 4.33237200 -1.45694300

H -5.07740300 3.77760700 0.74451900

C -3.68562100 -2.01870300 -0.66446300

C -3.82620700 -3.10834900 -1.74012000

H -4.02688300 -2.67712200 -2.72614000

H -2.92694500 -3.72861600 -1.82380700

H -4.66218400 -3.76415700 -1.47661900

C -4.96273300 -1.18643100 -0.57216400

H -5.12798700 -0.62774100 -1.50024300

H -5.83151400 -1.83114000 -0.40169400

H -4.89277000 -0.47379400 0.25558200

O -3.51241500 -2.65065200 0.62574300

C -2.14417200 -2.98347400 0.78114400

H -1.91354300 -3.95409500 0.31991100

C -1.75253300 -2.91630100 2.25220300

H -2.60473700 -3.07467600 2.91749100

H -0.97724400 -3.64431700 2.51602100

C -1.18865400 -1.51204900 2.42644200

O -1.07097800 -0.92211200 1.21265900

O -0.86034800 -0.94198100 3.43803500

H -1.61585300 -1.79860800 -2.72061500

H -2.79481500 -0.51822900 -2.83442700

H -0.55353900 0.20155500 -3.46431700

H -1.26607500 1.19138100 -2.21907400

C 6.36222000 -0.60789700 1.06321300

H 6.83678100 -1.24614300 1.80420000

H 7.00206800 -0.22646100 0.27025800

**TS4**

C 0.69627300 -1.36567300 -0.88615300

C 1.78162900 -2.14736000 -1.68165300

C 1.57061100 0.55632700 0.18014200

C 3.21428600 -1.72285700 -1.29523100

C 2.87527400 0.83343700 -0.55440000

C 3.57995100 -0.23288200 -1.39703900

H 1.60241600 -2.02738400 -2.75786500

H 3.39608600 -2.08466800 -0.28381200

H 1.69958400 -3.22377500 -1.47860700

H 3.91434100 -2.27321300 -1.93837500

H 2.82858700 1.80691100 -1.03667200

C 0.22026200 -0.10716700 -1.69205600

H 1.00793800 0.10535600 -2.42250100

C 0.35076600 1.02345200 -0.66995200

O 1.31810000 -0.84199700 0.31804600

O 1.59863100 1.13216400 1.45965500

H 0.79894100 0.82570500 1.93009700

C 4.29449400 0.42486000 -0.21686400

H 5.01786800 1.17288900 -0.53619700

C 4.03252500 0.18126400 -2.79185300

H 3.24617300 0.00233300 -3.53741100

H 4.28652800 1.24601900 -2.83307400

H 4.91784800 -0.38803700 -3.10356900

C 4.85202700 -0.31787400 0.98036300

C 3.98103700 -0.85067800 2.10109800

H 4.61053000 -1.13614400 2.94858600

H 3.24100600 -0.11647700 2.41854100

H 3.44106200 -1.74080700 1.76189400

H -0.51467700 1.06414200 -0.00258900

C -0.38420000 -2.33394000 -0.36209000

C -1.64857800 -1.73241000 0.26264300

C -2.68534800 -1.12029200 -0.71484300

C -1.10196300 -0.10247600 -2.51091800

C -2.25037100 -1.07671300 -2.18193300

H -0.68958700 -3.02182600 -1.15865100

H 0.12033500 -2.95135700 0.39323600

H -2.84977800 -0.09124400 -0.37692900

O 0.50001800 2.25791200 -1.34680800

C 0.30228600 3.42324400 -0.52717600

H 0.60584800 4.25510900 -1.17101900

H 0.97230300 3.39650100 0.33947500

C -1.13318600 3.59903800 -0.07618100

C -1.47775700 3.51646100 1.27797300

C -2.14584000 3.83276000 -1.01875100

C -2.80550100 3.66848200 1.68706500

H -0.70144800 3.32741200 2.01553600

C -3.47198600 3.98203800 -0.61494000

H -1.88667000 3.89973300 -2.07300000

C -3.80462300 3.90057000 0.74128200

H -3.05643500 3.60016100 2.74212400

H -4.24626500 4.16968500 -1.35458200

H -4.83764100 4.02088000 1.05709700

C -4.00214700 -1.91392500 -0.41443300

C -4.22955400 -3.12940000 -1.32916400

H -4.44370800 -2.82447200 -2.35863800

H -3.36333800 -3.79998400 -1.35389500

H -5.08543500 -3.70145400 -0.95686200

C -5.23428800 -1.01187100 -0.40385000

H -5.39904500 -0.57162800 -1.39379700

H -6.12796400 -1.58275900 -0.13056800

H -5.10562000 -0.20226400 0.32126200

O -3.81761500 -2.37558500 0.94456100

C -2.46245800 -2.75357400 1.10939100

H -2.29245500 -3.78765500 0.77815700

C -2.02076800 -2.51063000 2.54749200

H -2.85835500 -2.53235100 3.24892900

H -1.27544800 -3.23763900 2.88895100

C -1.38219600 -1.12802300 2.51582900

O -1.28289200 -0.70929200 1.23090600

O -0.98563000 -0.44866900 3.43070700

H -1.99632600 -2.08394700 -2.53089500

H -3.11136000 -0.76873700 -2.79034900

H -0.85359200 -0.26394900 -3.56711700

H -1.48406900 0.92498800 -2.45959800

C 6.15531200 -0.86374800 0.81892300

H 6.43226400 -1.71964400 1.42746400

H 6.60864000 -0.83760300 -0.16756000

B 6.37211700 0.60817300 1.66921100

H 6.73503300 0.35628700 2.78578700

H 5.25836100 1.13295000 1.83554900

H 6.93621100 1.38218100 0.94252000

**CP4**

C -0.72073300 -1.11747400 -0.79025400

C 0.35767700 -1.91249100 -1.57013700

C 0.30232500 0.83309900 0.32790300

C 1.81594100 -1.50105000 -1.27096200

C 1.55779000 1.02533100 -0.52105800

C 2.19021400 -0.02328900 -1.43100100

H 0.12258500 -1.82409000 -2.63914400

H 2.05210500 -1.85372700 -0.27016500

H 0.27763700 -2.97577500 -1.31924300

H 2.46996200 -2.08152200 -1.93223400

H 1.44559700 1.99980200 -0.99336900

C -1.09168800 0.21277100 -1.53158200

H -0.27266800 0.40015400 -2.22877900

C -0.94708200 1.29397700 -0.45464200

O -0.06034900 -0.61673600 0.47675900

O 0.31037700 1.43771400 1.56584100

H 0.95020100 0.97339500 2.13532600

C 3.02380400 0.64901900 -0.34443400

H 3.65055200 1.45278000 -0.73815000

C 2.47583500 0.38277700 -2.87205000

H 1.61707600 0.19060200 -3.52847200

H 2.71510100 1.44932800 -2.95217300

H 3.32431000 -0.18687000 -3.27307700

C 3.68975000 -0.01279600 0.85807800

H 3.03979800 -0.78584900 1.26861100

C 3.92062100 1.01559400 1.98242400

H 2.98164700 1.50659000 2.25344100

H 4.32139300 0.53312100 2.88043900

H 4.61654500 1.80938700 1.68998800

C 4.99359800 -0.76808700 0.50210100

H 5.56894000 -0.91320800 1.43131700

O 4.62304900 -2.02760600 -0.03962400

H -1.78512500 1.25484700 0.24311300

C -1.86828900 -2.05892100 -0.37332200

C -3.22915800 -1.49564400 0.05534100

C -4.14433100 -1.01288200 -1.10157800

C -2.39409800 0.35641500 -2.38369700

C -3.43662600 -0.77223100 -2.43452400

H -2.05584200 -2.71952200 -1.22665500

H -1.47820700 -2.71435700 0.40718400

H -4.55325200 -0.05317100 -0.76625800

O -0.83314600 2.56672800 -1.04972800

C -1.23842900 3.66699500 -0.19973800

H -0.89380700 4.55458800 -0.73817800

H -0.70575500 3.61079100 0.75528300

C -2.73344700 3.72210900 0.01915800

C -3.31428500 3.16981500 1.16896600

C -3.56881800 4.28832900 -0.95563900

C -4.70107600 3.17423600 1.33806700

H -2.68892000 2.71552300 1.93271400

C -4.95286300 4.30534500 -0.78424400

H -3.12765000 4.72070900 -1.85135800

C -5.52171200 3.74542300 0.36412300

H -5.12808600 2.72162500 2.22806700

H -5.58760900 4.75662500 -1.54268800

H -6.60049000 3.75716300 0.49758400

C -5.33110700 -2.02316100 -1.09883400

C -5.10859100 -3.26595100 -1.97715200

H -5.10946900 -3.00798700 -3.04162500

H -4.16137300 -3.77072000 -1.75671800

H -5.91683900 -3.98242000 -1.79852100

C -6.66470300 -1.36083500 -1.44075700

H -6.65503500 -0.97355500 -2.46583200

H -7.48435900 -2.08219200 -1.35644900

H -6.86372000 -0.53174000 -0.75468100

O -5.41322400 -2.41703700 0.28899800

C -4.09754900 -2.55684300 0.80340500

H -3.72151900 -3.57622900 0.64184000

C -4.05181500 -2.13262800 2.26396700

H -5.01274300 -2.24960800 2.76856300

H -3.28786800 -2.68081600 2.82509800

C -3.62597500 -0.67361300 2.22084500

O -3.08066700 -0.40569400 0.99648800

O -3.71646300 0.16917000 3.07422100

H -2.98801700 -1.69589100 -2.81927900

H -4.18890800 -0.48012500 -3.17972500

H -2.08304400 0.55257300 -3.41582600

H -2.89549700 1.27474300 -2.05633600

H 5.44843200 -2.47904000 -0.28535600

C 5.95053900 -0.08729400 -0.50733400

H 5.42497800 0.03214900 -1.46201000

C 6.65380200 1.16864900 -0.08116500

H 6.13479200 2.09687600 0.12688700

C 7.97634000 0.96578700 -0.02303900

C 9.09801800 1.88003200 0.34755400

H 9.81467700 1.96352200 -0.47778900

H 8.73576800 2.87977100 0.60282900

H 9.65373300 1.47829100 1.20305300

C 8.23659300 -0.44203100 -0.42954600

O 7.02728000 -1.02725300 -0.73463600

O 9.28525100 -1.03119200 -0.51310100

B 0.20749300 -1.52770900 1.82835500

F -0.96132700 -1.56774100 2.52812600

F 0.59910900 -2.74651900 1.34555500

F 1.21808800 -0.84614500 2.48895900

**TS5**

C -0.89956000 -1.21052200 -0.35532100

C 0.24968000 -2.10855500 -0.92156800

C 0.75650500 1.18211900 0.04312800

C 1.69549500 -1.63517200 -0.64616900

C 1.77289300 1.02976900 -0.98732500

C 2.20610200 -0.42154100 -1.41857300

H 0.07773100 -2.28725600 -1.99166900

H 1.79540100 -1.49600500 0.43016900

H 0.16691900 -3.08752300 -0.43296500

H 2.37491900 -2.45704000 -0.89780700

H 1.54450600 1.65437900 -1.84712500

C -1.11419900 0.07319100 -1.28300000

H -0.40317900 -0.02247400 -2.10869000

C -0.68379500 1.30765200 -0.44288400

O -0.47090400 -0.67062300 0.87028100

O 1.12034200 1.37392600 1.25763400

H 0.42857200 1.05359400 1.96688500

C 3.20271800 0.54410200 -0.82384500

H 3.84080300 1.00184100 -1.58217000

C 2.30398500 -0.63593600 -2.92161900

H 1.34385500 -0.92044000 -3.36524600

H 2.65920100 0.26134000 -3.44019000

H 3.01630000 -1.44621700 -3.11998100

C 3.94100600 0.37110300 0.50350300

H 3.29790600 -0.16430500 1.20837700

C 4.28399900 1.73537700 1.12837400

H 3.37433200 2.21756500 1.49033700

H 4.96669100 1.62301600 1.97870700

H 4.74949300 2.41410000 0.40381500

C 5.18362200 -0.54102900 0.34549700

H 5.61327900 -0.69330500 1.34962100

O 4.73354300 -1.77350700 -0.18927600

H -1.35566100 1.39522100 0.41040700

C -2.11942300 -2.13795100 -0.13838500

C -3.50171400 -1.53208500 0.11767500

C -4.29720400 -1.13363700 -1.14785100

C -2.48170800 0.40821500 -1.95017600

C -3.41744200 -0.75569200 -2.34082400

H -2.21112300 -2.80969400 -0.99920200

H -1.85518100 -2.76853900 0.71387000

H -4.84519700 -0.22769600 -0.86304400

O -0.70749200 2.49433200 -1.23957800

C -1.06306600 3.69168900 -0.50510700

H -0.70407000 4.50997300 -1.13552100

H -0.50683400 3.72373000 0.44175900

C -2.54904700 3.80972800 -0.26235900

C -3.13598600 3.31953800 0.91254800

C -3.37550800 4.36717400 -1.24922700

C -4.52030700 3.36396400 1.09122500

H -2.52140700 2.88694800 1.69777100

C -4.75628500 4.43101300 -1.06683000

H -2.93093100 4.75194900 -2.16461300

C -5.33102300 3.92419000 0.10280800

H -4.94987900 2.94217700 1.99415100

H -5.38417600 4.87351200 -1.83584100

H -6.40812700 3.96695800 0.24179400

C -5.38130300 -2.23521600 -1.28120100

C -4.92635700 -3.50931500 -2.01147200

H -4.71110200 -3.30307100 -3.06606600

H -4.02915200 -3.95069600 -1.56594000

H -5.72251200 -4.25968000 -1.96576300

C -6.67783100 -1.71002700 -1.89638000

H -6.52067800 -1.40994200 -2.93859000

H -7.45123800 -2.48512000 -1.87721200

H -7.04406500 -0.84595300 -1.33325500

O -5.67717400 -2.53042000 0.10517500

C -4.46166000 -2.52308200 0.84781000

H -4.03743400 -3.53451300 0.88789700

C -4.65320500 -1.91911200 2.23408800

H -5.69673800 -1.92364100 2.55607500

H -4.04779900 -2.43198800 2.98817500

C -4.12191400 -0.49644200 2.12517700

O -3.40526800 -0.36617100 0.97412900

O -4.27066000 0.42155800 2.89136200

H -2.85221800 -1.61379900 -2.72508700

H -4.06044800 -0.42359100 -3.16617900

H -2.24777900 0.99829000 -2.84180900

H -3.04698500 1.08079200 -1.29718400

H 5.52545000 -2.31717800 -0.33964500

C 6.33950700 -0.01822900 -0.55447500

H 5.94284300 0.26266700 -1.53797100

C 7.24832100 1.03700100 0.01334600

H 6.91358100 2.03473100 0.26665300

C 8.49397900 0.56252000 0.14168900

C 9.74206100 1.20329100 0.65463700

H 10.52069500 1.20053700 -0.11710900

H 9.56394000 2.23439900 0.97204700

H 10.14299300 0.63762800 1.50385100

C 8.49717900 -0.84129200 -0.34779300

O 7.21896600 -1.14690500 -0.76844000

O 9.40553500 -1.63073700 -0.40727700

B -0.37486400 -1.10958500 2.25895400

F -1.51312200 -1.73942300 2.73690600

F 0.76763000 -1.85836200 2.49383300

F -0.22199400 0.19220900 2.94170000

**CP5**

C -0.98117900 -1.17170800 -0.23045700

C 0.20960000 -2.08809200 -0.68182900

C 0.85149300 1.15386900 0.09912000

C 1.65733700 -1.60452800 -0.41817000

C 1.85149900 1.03570700 -0.93260200

C 2.21437000 -0.47910300 -1.28774100

H 0.06965900 -2.35168700 -1.73897700

H 1.74338400 -1.37640700 0.64400700

H 0.11728700 -3.02095100 -0.11559200

H 2.32819200 -2.45636400 -0.57962800

H 1.61832900 1.62235500 -1.81478500

C -1.09116100 0.10328000 -1.18511300

H -0.38716100 -0.05771000 -2.00724900

C -0.58935700 1.33604900 -0.36840200

O -0.72496600 -0.63265000 1.03699000

O 1.21768200 1.11815400 1.32481300

C 3.25696500 0.47854700 -0.78190600

H 3.88410200 0.86261500 -1.58814200

C 2.25867400 -0.78753500 -2.77531300

H 1.27853800 -1.06011400 -3.17870400

H 2.63998100 0.05872700 -3.35666000

H 2.93439900 -1.63748900 -2.93283300

C 4.03990400 0.34837200 0.52476800

H 3.42837700 -0.18193600 1.25871900

C 4.38055100 1.72826700 1.11226400

H 3.47312100 2.20451200 1.49016500

H 5.08565000 1.64273400 1.94715100

H 4.81672300 2.39976600 0.36320100

C 5.28164200 -0.55797500 0.33446000

H 5.73386600 -0.71666700 1.32749900

O 4.81559700 -1.78679600 -0.19771000

H -1.24647600 1.45539800 0.49077400

C -2.22284500 -2.09467200 -0.14627600

C -3.61456900 -1.48216400 0.04157800

C -4.32308500 -1.02267800 -1.25430800

C -2.43153600 0.51377600 -1.85983600

C -3.36308400 -0.60782800 -2.37076400

H -2.26605500 -2.74051800 -1.03079200

H -2.02396900 -2.75704900 0.70198800

H -4.87531900 -0.12054900 -0.96663300

O -0.58152900 2.51239700 -1.17855600

C -0.92375600 3.72357500 -0.45940400

H -0.55795400 4.52914200 -1.10204800

H -0.36464900 3.76222800 0.48597200

C -2.40796800 3.85867900 -0.21455700

C -2.99775800 3.37563100 0.96202000

C -3.23202300 4.41503600 -1.20376300

C -4.38245100 3.41764400 1.13798600

H -2.38191700 2.95042700 1.75051400

C -4.61301700 4.48116500 -1.02288700

H -2.78604400 4.79515300 -2.12042100

C -5.19052400 3.97532700 0.14576400

H -4.81597500 2.99072900 2.03667000

H -5.23937300 4.92252900 -1.79380200

H -6.26809700 4.01584300 0.28123100

C -5.41382400 -2.09935400 -1.49573000

C -4.93592500 -3.35284500 -2.24726700

H -4.66547500 -3.11354500 -3.28214800

H -4.06686800 -3.82057600 -1.77359800

H -5.74150600 -4.09424600 -2.26758300

C -6.66402600 -1.52907600 -2.16435100

H -6.44123900 -1.18987200 -3.18240400

H -7.44807200 -2.29150200 -2.22254800

H -7.05114300 -0.68245500 -1.58875700

O -5.79635300 -2.44315600 -0.14140000

C -4.62360300 -2.49592800 0.66511100

H -4.21582200 -3.51499500 0.67244500

C -4.87605500 -1.96311500 2.07027300

H -5.92892200 -1.99751900 2.35750000

H -4.28530600 -2.50460600 2.81658200

C -4.35756000 -0.53153800 2.04825500

O -3.57534700 -0.35250500 0.94895400

O -4.56572800 0.35504000 2.83727100

H -2.79117800 -1.45924100 -2.76037200

H -3.94545900 -0.22215600 -3.21759300

H -2.15955500 1.16724900 -2.69400500

H -3.00763600 1.14070700 -1.17244500

H 5.60205100 -2.33193300 -0.37037400

C 6.41683000 -0.02898900 -0.58683500

H 6.00099700 0.25917700 -1.56035200

C 7.33503700 1.02373800 -0.02929700

H 7.00377000 2.01914900 0.23758200

C 8.58356900 0.55010100 0.07045900

C 9.84105300 1.18904800 0.56211200

H 10.60278000 1.19468600 -0.22629500

H 9.66780500 2.21696000 0.89224200

H 10.26126200 0.61671400 1.39738500

C 8.57869000 -0.85055500 -0.42886700

O 7.29199700 -1.15510500 -0.82590700

O 9.48615500 -1.63841400 -0.51200000

B -0.56335000 -1.22601800 2.35328800

F -1.75025400 -1.67305500 2.91375100

F 0.41747300 -2.21099300 2.39445200

F -0.07836900 -0.04369300 3.09158800

H 0.51647800 0.77655400 2.01792000

**CP6**

C -1.26356000 -1.14228600 0.46731300

C 0.02365000 -2.04747200 0.54449900

C 0.91168400 1.16228600 -0.15582000

C 1.47307200 -1.51161700 0.54911600

C 1.74663700 0.47803500 -1.14435800

C 2.00825800 -1.01411500 -0.79545900

H -0.05460900 -2.79750400 -0.25104800

H 1.59313600 -0.75667300 1.33139600

H -0.09149900 -2.60149600 1.48364000

H 2.12084200 -2.33894700 0.85900100

H 1.45168700 0.67232800 -2.17128700

C -1.28189900 -0.13590400 -0.75918800

H -0.73535900 -0.60274200 -1.58933200

C -0.58547000 1.21172200 -0.40846100

O -1.47913500 -0.44440200 1.68642100

O 1.46148000 1.70662500 0.83028600

C 3.15114700 -0.02896200 -0.86305800

H 3.70494100 -0.11418100 -1.79903600

C 1.91859200 -2.01169500 -1.93890000

H 0.89067200 -2.31953800 -2.15665900

H 2.33993000 -1.59648800 -2.86085200

H 2.49354800 -2.91001600 -1.68168500

C 4.06721700 0.37451200 0.29412000

H 3.50912400 0.33084000 1.23225300

C 4.57854700 1.81701900 0.13494500

H 3.77138700 2.51963400 0.35145200

H 5.39704700 2.02831600 0.83271500

H 4.93499000 2.01898200 -0.88238200

C 5.20657400 -0.65533500 0.49042400

H 5.77743100 -0.35158800 1.38351000

O 4.59603200 -1.91868900 0.70682900

H -1.10044600 1.65457600 0.44342800

C -2.39458000 -2.22303500 0.42628500

C -3.89859000 -1.90803300 0.42547600

C -4.51831400 -1.44844900 -0.91779500

C -2.68136500 0.25212400 -1.29140800

C -3.51047000 -0.88660800 -1.91882000

H -2.21998100 -2.87926300 -0.43464800

H -2.20824000 -2.83521300 1.31659800

H -5.19788400 -0.63161200 -0.65017700

O -0.60198900 2.10072400 -1.54153700

C -0.79982400 3.50698900 -1.23074200

H -0.36959000 4.02609000 -2.09215300

H -0.22470200 3.77257600 -0.33855200

C -2.25500500 3.87021900 -1.05701900

C -2.81648200 4.00154000 0.22045800

C -3.07014100 4.06145000 -2.18242900

C -4.17188400 4.30650800 0.36782300

H -2.19136800 3.86638800 1.09789200

C -4.42200300 4.36911000 -2.03608900

H -2.63904400 3.97056300 -3.17734400

C -4.97580500 4.48946400 -0.75811300

H -4.59674600 4.40075100 1.36319900

H -5.04246300 4.52044500 -2.91557700

H -6.02921100 4.73009200 -0.64203100

C -5.42125400 -2.63319300 -1.36497100

C -4.70460100 -3.69648000 -2.21390000

H -4.44146700 -3.30562200 -3.20281500

H -3.78417700 -4.05610400 -1.74101700

H -5.36826000 -4.55636200 -2.35117000

C -6.69840800 -2.16879400 -2.06348900

H -6.46407200 -1.65995600 -3.00535600

H -7.34504500 -3.02349700 -2.28922300

H -7.25394400 -1.47873800 -1.42077600

O -5.83141700 -3.21118900 -0.10480700

C -4.72527100 -3.18933000 0.78402500

H -4.12214900 -4.10094100 0.67264900

C -5.18997000 -2.96098900 2.21283000

H -6.23671500 -3.23655900 2.35987300

H -4.58828100 -3.51488800 2.94235700

C -4.98994000 -1.46744900 2.45364000

O -4.23560400 -0.94179300 1.44815900

O -5.40009700 -0.80425700 3.36913800

H -2.85005300 -1.67187200 -2.30906100

H -4.05855100 -0.49952600 -2.78731000

H -2.52283500 1.02730500 -2.04249800

H -3.25329700 0.72219500 -0.48212100

H 5.31759400 -2.56822600 0.76272300

C 6.23961700 -0.80802300 -0.65984800

H 5.71779100 -1.00873200 -1.60361100

C 7.28190200 0.26583800 -0.80352500

H 7.04575200 1.28920600 -1.06594500

C 8.50233800 -0.23287100 -0.56870800

C 9.84760700 0.41640900 -0.57674800

H 10.50786200 -0.07598400 -1.30020600

H 9.78010600 1.47870400 -0.82685100

H 10.32864000 0.31563200 0.40328600

C 8.35082200 -1.68111700 -0.26770100

O 7.00879900 -1.99374100 -0.35074100

O 9.19172300 -2.50114900 0.00077800

B 0.89757400 2.23656100 2.26052700

F -0.17002500 3.05891800 1.99741300

F 0.54698100 1.05376900 2.89690000

F 1.97884000 2.85445600 2.79055700

H -0.65783100 -0.06526600 2.04565300

**TS6**

C -1.14821300 -1.15534500 -0.36097000

C 0.10374600 -1.56903600 -1.27043200

C 0.68940700 1.25239800 -0.08402000

C 1.47136500 -1.66101700 -0.51624600

C 1.61673700 1.14411400 -1.07688500

C 2.58914200 -0.96961600 -1.25652700

H 0.17167900 -0.86772300 -2.10323500

H 1.34680100 -1.25686400 0.48769100

H -0.09980100 -2.54716800 -1.71898700

H 1.75151000 -2.71483000 -0.41313800

H 1.28790200 1.36145700 -2.08671400

C -1.65287400 0.27998800 -0.77543300

H -1.65686400 0.26772500 -1.87330000

C -0.76968600 1.54054500 -0.42323500

O -0.78126800 -1.30171100 0.99048800

O 0.99478100 1.00789400 1.17698100

C 2.95545400 0.45745500 -0.93442100

H 3.63212100 0.82540700 -1.71920300

C 2.89500300 -1.47534500 -2.63925500

H 2.16376500 -1.03845500 -3.33144800

H 3.88842000 -1.16966900 -2.98109500

H 2.79347800 -2.56246500 -2.70687800

C 3.70452200 0.52334200 0.42307100

H 3.01340600 0.22243700 1.21042600

C 4.19394800 1.93229700 0.76169900

H 3.32776900 2.58852300 0.87702900

H 4.74084700 1.94321800 1.71144400

H 4.83850800 2.35629200 -0.01911100

C 4.79769900 -0.55517600 0.34832300

H 5.12468000 -0.85499800 1.35016200

O 4.13392200 -1.70835300 -0.23493700

H -1.22110900 2.02986500 0.43992800

C -2.20731300 -2.26535800 -0.67385200

C -3.54276700 -2.37574700 0.08036000

C -4.66381600 -1.35573100 -0.24358500

C -3.08706000 0.65726600 -0.33399900

C -4.23152300 -0.11179600 -1.01787800

H -2.43545700 -2.25736000 -1.74890100

H -1.67897400 -3.20896200 -0.48313100

H -5.02416500 -1.01760300 0.73427900

O -0.87542000 2.39223000 -1.56397800

C -0.66096400 3.78468500 -1.26537400

H -0.33404600 4.22557800 -2.21308300

H 0.15465000 3.88950600 -0.53935900

C -1.91095400 4.47921800 -0.76821500

C -2.10722300 4.73035700 0.59679600

C -2.90504100 4.86447000 -1.67994800

C -3.27687000 5.35619500 1.03727400

H -1.35264400 4.41975200 1.31380400

C -4.07127900 5.48988300 -1.24098300

H -2.75862200 4.67228500 -2.74093400

C -4.25845000 5.73836500 0.12211800

H -3.41829300 5.54308400 2.09851500

H -4.83088300 5.78851200 -1.95916400

H -5.16538300 6.22863100 0.46680800

C -5.80971500 -2.21180100 -0.86553800

C -5.74503300 -2.33842600 -2.39693600

H -5.98071000 -1.38786700 -2.88662300

H -4.75686300 -2.65798900 -2.74677800

H -6.47551200 -3.08450000 -2.72663700

C -7.19593800 -1.74178300 -0.42792400

H -7.39018800 -0.72430400 -0.78518900

H -7.97212400 -2.40038700 -0.83242700

H -7.27206200 -1.75030500 0.66382000

O -5.60860300 -3.51265800 -0.26895300

C -4.21471900 -3.76059400 -0.19775300

H -3.84775100 -4.20563000 -1.13349600

C -3.89256400 -4.59760200 1.02759700

H -4.73804600 -5.20675700 1.35457900

H -3.03658300 -5.26276000 0.86454400

C -3.51980300 -3.57550100 2.09795500

O -3.31910300 -2.36200300 1.51410900

O -3.39890800 -3.75515500 3.28103100

H -3.94287500 -0.36154700 -2.04721700

H -5.10207400 0.55153100 -1.10553900

H -3.20340600 1.71825500 -0.57734100

H -3.17130300 0.58233600 0.75787000

H 4.80674700 -2.21298200 -0.74039000

C 6.04485500 -0.20756500 -0.50469200

H 5.76179100 0.40598700 -1.36838900

C 7.18913700 0.39920200 0.25860700

H 7.11255700 1.35409700 0.76506500

C 8.26035800 -0.40265200 0.20440900

C 9.62456300 -0.26062000 0.79590100

H 10.38779600 -0.28329900 0.00947900

H 9.72301000 0.67246100 1.35648600

H 9.84075200 -1.09994900 1.46697200

C 7.89924700 -1.59913000 -0.60216100

O 6.57892400 -1.44324100 -1.02141700

O 8.55100100 -2.56429800 -0.89473600

B 0.27114800 1.57145500 2.38518600

F 0.13466800 2.95065100 2.20525200

F -1.01503500 0.96971600 2.45900400

F 1.04572900 1.24605300 3.46699500

H -1.02930500 -0.52888600 1.53583700

**CP7**

C -1.10623200 -1.12667600 -0.29691500

C 0.19190700 -1.51337400 -1.15432300

C 0.67956600 1.32858100 -0.14179200

C 1.51315700 -1.60569900 -0.34129500

C 1.57253100 1.20623500 -1.15173300

C 2.72232200 -1.02262400 -1.07044900

H 0.29329800 -0.79792300 -1.97058000

H 1.37752400 -1.11113100 0.62039100

H 0.01795800 -2.48558100 -1.62938100

H 1.73493200 -2.65803300 -0.12720400

H 1.21509800 1.40150400 -2.15662300

C -1.64391900 0.27893700 -0.75917300

H -1.63743000 0.23555800 -1.85661300

C -0.79799600 1.57089900 -0.43261400

O -0.80926800 -1.23371900 1.07667600

O 1.01982900 1.08646500 1.12346000

C 2.90776900 0.51950800 -1.02137600

H 3.53069400 0.79498500 -1.88660100

C 3.01859000 -1.66264100 -2.41506700

H 2.25211900 -1.33520200 -3.12452100

H 3.99076900 -1.35624800 -2.81656000

H 2.97237500 -2.75638200 -2.36601900

C 3.72683300 0.82217700 0.26851400

H 3.05856800 0.70801900 1.12339400

C 4.29232000 2.24286300 0.30561400

H 3.45628000 2.94688900 0.28681700

H 4.85605800 2.42587400 1.22731500

H 4.93769700 2.46700900 -0.55365700

C 4.76053000 -0.29698000 0.33859900

H 5.06646600 -0.54877200 1.35672200

O 3.97916600 -1.48190800 -0.11870700

H -1.24180200 2.04495500 0.44366800

C -2.11471200 -2.27836500 -0.61976000

C -3.46842900 -2.43386100 0.09371800

C -4.60968900 -1.44073200 -0.24668300

C -3.09036300 0.62079200 -0.33257400

C -4.20362500 -0.18811300 -1.02201200

H -2.31025400 -2.29197900 -1.70110600

H -1.55900800 -3.20062800 -0.40243600

H -4.98899800 -1.10825600 0.72588100

O -0.97048900 2.41492800 -1.57158800

C -0.78579500 3.81250700 -1.28396500

H -0.49890600 4.25986200 -2.24183700

H 0.04595500 3.94227900 -0.58105900

C -2.04064700 4.47391800 -0.75358100

C -2.19956300 4.73658200 0.61404800

C -3.07694500 4.81450500 -1.63561900

C -3.37357900 5.33059800 1.08604000

H -1.41124100 4.45926900 1.30803400

C -4.24786900 5.40755100 -1.16525800

H -2.96004700 4.61211800 -2.69840300

C -4.39742200 5.66839600 0.20020400

H -3.48574500 5.52653000 2.14920300

H -5.04075800 5.67111100 -1.86094800

H -5.30818500 6.13321400 0.56936400

C -5.72759500 -2.32515200 -0.88240900

C -5.65376300 -2.43357800 -2.41483200

H -5.91382800 -1.48509700 -2.89606400

H -4.65565500 -2.72185000 -2.76369100

H -6.36194900 -3.19646000 -2.75474700

C -7.12884300 -1.90120400 -0.44536900

H -7.35113800 -0.88531000 -0.79064100

H -7.88402400 -2.57699000 -0.86130300

H -7.20870100 -1.92559800 0.64586200

O -5.49190500 -3.62723300 -0.30172700

C -4.09180400 -3.83321400 -0.22091700

H -3.70135200 -4.24886300 -1.16081800

C -3.76040000 -4.68351900 0.99214800

H -4.58365900 -5.33827500 1.28589400

H -2.87065200 -5.30435300 0.83461400

C -3.46062900 -3.66728500 2.09066900

O -3.29077100 -2.43800900 1.53479600

O -3.36869900 -3.86543700 3.27404100

H -3.89488900 -0.43464800 -2.04629500

H -5.09346000 0.44719900 -1.12417400

H -3.23679200 1.67611000 -0.58292900

H -3.18063100 0.54728700 0.75917300

H 4.60754800 -2.09683100 -0.56904100

C 6.00168800 -0.18472700 -0.57453800

H 5.74725700 0.33312000 -1.50641300

C 7.21494300 0.40625100 0.08801700

H 7.24125800 1.42559100 0.45518900

C 8.20375000 -0.49611100 0.13389500

C 9.58938000 -0.40536900 0.68381900

H 10.32705700 -0.60030000 -0.10307900

H 9.78485700 0.57950500 1.11511300

H 9.74521500 -1.16697100 1.45649400

C 7.71470600 -1.75287500 -0.49374100

O 6.40021600 -1.52609700 -0.92049700

O 8.25903000 -2.80882400 -0.65431000

B 0.36943800 1.71179200 2.32887900

F 0.20817600 3.08301700 2.09669900

F -0.90463900 1.10532300 2.50793200

F 1.20844400 1.44917800 3.38545100

H -0.93282800 -0.39346900 1.56157300

**CP8**

C -0.99973400 -0.71145900 -0.27537800

C 0.38368500 -1.20844600 -0.88708200

C 0.80818800 1.66858200 0.11974100

C 1.63449400 -1.38749400 0.02082500

C 1.86187000 1.42396300 -0.66208200

C 2.97808200 -0.84598000 -0.53991700

H 0.62011500 -0.53551900 -1.71180500

H 1.48794200 -0.96629400 1.01376500

H 0.18975800 -2.17550100 -1.36107400

H 1.79155600 -2.45607300 0.19920200

H 1.67158400 1.61985400 -1.71589800

C -1.39822100 0.69322200 -0.86999800

H -1.15371700 0.61360000 -1.93646800

C -0.58470900 1.96049800 -0.40304100

O -0.96828800 -0.61475500 1.19068900

O 0.82093700 1.40804900 1.49746100

C 3.14482000 0.70617100 -0.35823700

H 3.90449500 1.03356800 -1.07675300

C 3.25145000 -1.31504800 -1.97092400

H 2.51275200 -0.91967900 -2.67644800

H 4.24142000 -0.98665200 -2.29972100

H 3.21741200 -2.40908100 -2.02256600

C 3.75307400 0.82690800 1.05579900

H 2.95735500 0.70181800 1.79287200

C 4.44620500 2.16371600 1.32827000

H 3.71578300 2.97912100 1.28037000

H 4.89481200 2.18726000 2.32922900

H 5.23082900 2.37212300 0.59341900

C 4.63034600 -0.44465700 1.14098700

H 4.62701700 -0.83573700 2.16969200

O 4.02587300 -1.40850000 0.28961900

H -1.15448000 2.44333100 0.40995700

C -2.01856500 -1.82424700 -0.64415400

C -3.50609700 -1.82914100 -0.24441000

C -4.47576500 -0.90119200 -1.02323700

C -2.89180200 1.07542100 -0.77787200

C -3.82498800 0.28974300 -1.72237000

H -1.98895700 -1.90857300 -1.73707100

H -1.58435200 -2.74822200 -0.25744100

H -5.14007600 -0.49666500 -0.25152000

O -0.57402700 2.81228600 -1.54018800

C -0.15863600 4.16337600 -1.29183200

H -0.09256000 4.60468400 -2.29130100

H 0.84720100 4.17224500 -0.85177500

C -1.11825900 4.94810800 -0.42235000

C -0.70832100 5.47169800 0.80935900

C -2.43944100 5.16367000 -0.84210200

C -1.59516800 6.19928400 1.60804700

H 0.31753900 5.31998700 1.14030000

C -3.32846600 5.88425100 -0.04611500

H -2.76569500 4.76471500 -1.79950900

C -2.90754100 6.40432300 1.18187900

H -1.26099200 6.60276700 2.56008500

H -4.34851600 6.04641300 -0.38386600

H -3.60015900 6.96806700 1.80069700

C -5.34848400 -1.86567200 -1.87602700

C -4.76236700 -2.20858600 -3.25602900

H -4.78972000 -1.34464400 -3.92894700

H -3.72369000 -2.55216700 -3.19610300

H -5.35333400 -3.01188400 -3.70778300

C -6.79141200 -1.38313400 -2.01690100

H -6.83388500 -0.44016900 -2.57362300

H -7.39301500 -2.12390000 -2.55421800

H -7.23845100 -1.22902200 -1.02987700

O -5.39232400 -3.05482900 -1.05643800

C -4.11226700 -3.25512800 -0.47586000

H -3.47865200 -3.86231800 -1.13667000

C -4.24503300 -3.83935700 0.92007200

H -5.15464600 -4.42768000 1.05273800

H -3.38012200 -4.45859600 1.18331100

C -4.23341500 -2.62533500 1.83875100

O -3.67844300 -1.56556500 1.16633100

O -4.61926800 -2.53807700 2.97138000

H -3.26745500 -0.02467100 -2.61374300

H -4.61914100 0.95407100 -2.08646700

H -2.95358200 2.13774100 -1.03296600

H -3.23475400 0.97944500 0.26011100

C 6.11775300 -0.27536600 0.76580000

H 6.56943400 0.44921200 1.45756900

C 6.88950100 -1.56428300 0.74567100

H 6.98382500 -2.20312800 1.61752900

C 7.38751700 -1.78470800 -0.47580400

C 8.20053300 -2.91269100 -1.02195000

H 9.14480400 -2.54330600 -1.43903800

H 8.42319700 -3.65659500 -0.25190800

H 7.66799700 -3.40687800 -1.84305800

C 6.96912800 -0.64889000 -1.33956700

O 6.25901600 0.24294900 -0.56685700

O 7.17064000 -0.46779400 -2.51609300

B -0.87253400 -1.81593000 2.48611800

F -1.79831300 -1.36280000 3.34816800

F -1.12585800 -3.01079900 1.87965400

F 0.41937000 -1.63237000 2.88769400

H -0.27979500 0.04279100 1.45063400

H 0.27815400 2.08068000 1.9468980

**TS7**

C -0.92805700 -1.02972500 0.02839700

C 0.34363600 -1.97628300 0.15982900

C 0.67070300 1.31323700 -0.82399300

C 1.68519300 -1.44130400 0.75462500

C 1.77335900 0.72515600 -1.26834300

C 2.94765400 -1.13179300 -0.10653600

H 0.50702500 -2.47457000 -0.80288000

H 1.47761800 -0.59306800 1.40854600

H 0.04383400 -2.76853700 0.85057800

H 2.04485100 -2.22107800 1.43320100

H 1.61603400 0.25526700 -2.23905600

C -1.03360200 -0.41737000 -1.45685100

H -0.23095500 -0.89591300 -2.02784300

C -0.71796900 1.10199300 -1.39137100

O -0.84129000 0.04935800 0.95093700

O 0.69141200 2.00061300 0.46833400

C 3.06248700 0.34878200 -0.62399200

H 3.85035200 0.35599400 -1.38538600

C 3.18915800 -2.16319400 -1.20742600

H 2.41198700 -2.11941500 -1.97794400

H 4.15850000 -1.99199600 -1.68532700

H 3.18605200 -3.17428300 -0.78631000

C 3.59163500 1.10447800 0.60812800

H 2.78214600 1.23293400 1.33164500

C 4.18197200 2.48389300 0.30622800

H 3.40214900 3.15126200 -0.07754100

H 4.59054400 2.95046400 1.21084600

H 4.97697100 2.42968900 -0.44412700

C 4.55533700 0.05089900 1.21551200

H 4.52634400 0.10615900 2.31428300

O 4.07900600 -1.22304600 0.80095400

H -1.44176000 1.53310700 -0.69747300

C -2.13805100 -1.90768100 0.44740100

C -3.57047400 -1.40928200 0.20731900

C -4.16624700 -1.79424600 -1.16674800

C -2.31082200 -0.58762200 -2.33651800

C -3.11434600 -1.90440000 -2.27058100

H -2.04913300 -2.88598300 -0.03595300

H -1.99784600 -2.09597100 1.51471200

H -4.81647300 -0.95410900 -1.43897700

O -0.87984300 1.69089400 -2.67958500

C -0.68365600 3.11134300 -2.72567000

H -1.15372400 3.42088700 -3.66475900

H 0.39071900 3.33615300 -2.79640100

C -1.27350700 3.85445200 -1.54112400

C -0.45034500 4.62691500 -0.70373100

C -2.61813700 3.68042500 -1.18493900

C -0.96396700 5.20888200 0.46507100

H 0.58849000 4.79888900 -0.98378700

C -3.12445100 4.23077300 -0.00708500

H -3.25815900 3.06150100 -1.80794700

C -2.29745700 4.99664400 0.82020600

H -0.31928800 5.81507600 1.09601700

H -4.14642700 4.01721100 0.28821400

H -2.68876800 5.41492500 1.74267800

C -5.13752300 -2.96034600 -0.85033000

C -4.48547200 -4.34934900 -0.75899100

H -4.09413200 -4.66698800 -1.73225200

H -3.66177900 -4.37826000 -0.03905700

H -5.23306500 -5.08156400 -0.43650900

C -6.33279000 -3.00141700 -1.80190700

H -6.01032600 -3.23923400 -2.82197700

H -7.04905100 -3.76613600 -1.48328500

H -6.84618700 -2.03474200 -1.81254800

O -5.65684000 -2.56498100 0.44282400

C -4.59895000 -2.00698700 1.21895800

H -4.14052200 -2.78339400 1.84378300

C -5.07273700 -0.81036300 2.03788800

H -6.15880900 -0.78303800 2.15023800

H -4.61039800 -0.78724800 3.02910800

C -4.57125700 0.41397700 1.28434900

O -3.66450100 0.03096500 0.34518200

O -4.88724600 1.56833600 1.43130200

H -2.45733700 -2.77196900 -2.13498000

H -3.61842000 -2.05596400 -3.23387300

H -1.97388900 -0.42461500 -3.36429900

H -3.00336300 0.23322300 -2.12339600

C 6.04130800 0.18453200 0.82104900

H 6.41170300 1.15708700 1.17286600

C 6.90130600 -0.94362600 1.31676300

H 7.01349800 -1.17382400 2.37101000

C 7.44672500 -1.59533400 0.28408000

C 8.34832500 -2.78523400 0.23208500

H 9.28184300 -2.54177700 -0.28887500

H 8.58958300 -3.15018800 1.23425700

H 7.87720800 -3.59772800 -0.33337300

C 6.97612500 -0.93251400 -0.96127600

O 6.19328600 0.14512300 -0.60703700

O 7.19240500 -1.22275300 -2.11248800

B -0.94091900 -0.02779700 2.43418000

F -2.24778700 0.10963600 2.88213900

F -0.36333800 -1.18427700 2.94369800

F -0.16488500 1.13108000 2.78910100

H 0.15602100 1.38168900 1.12761200

H 0.20329700 2.86514100 0.38496400

**29**

C -0.82561800 -0.47195400 -0.86570700

C 0.27391600 -0.34253500 -1.97339600

C 0.08484100 1.17971500 0.30563600

C 1.65117300 -0.91714600 -1.54837700

C 1.32351700 1.67097600 0.31968100

C 2.78873700 0.03695100 -1.06818400

H 0.37344300 0.70947200 -2.25645500

H 1.50980300 -1.67742400 -0.77439500

H -0.08288900 -0.85893400 -2.87340000

H 2.08938100 -1.44767200 -2.40220000

H 1.43266600 2.74770500 0.22822100

C -1.86475300 0.69267100 -0.99545000

H -1.89848100 1.02423600 -2.03930200

C -1.20464900 1.83678000 -0.15072100

O -0.16016500 -0.17738800 0.39059400

C 2.58284500 0.84397800 0.25476500

H 3.42854200 1.53900300 0.29610400

C 3.26896200 0.93167800 -2.21142900

H 2.48880000 1.64301200 -2.50474400

H 4.15438000 1.49811600 -1.90658600

H 3.53191400 0.32980600 -3.08847000

C 2.84761100 -0.22108900 1.33981200

H 1.96729100 -0.86777300 1.40513800

C 3.12183500 0.34630600 2.73394100

H 2.23640100 0.88158800 3.09328400

H 3.33624400 -0.44941500 3.45839400

H 3.96345700 1.04693100 2.73014600

C 3.98810100 -1.04472000 0.68310200

H 3.86047200 -2.11597900 0.90831200

O 3.89226900 -0.84431800 -0.72637400

H -1.85595100 2.06715700 0.70309800

C -1.31109700 -1.93124700 -0.83863500

C -2.42813600 -2.41079000 0.09586900

C -3.88264200 -1.99836800 -0.25747300

C -3.30672400 0.45482600 -0.51467200

C -4.03134600 -0.75399400 -1.13694200

H -1.61355400 -2.19654000 -1.85836800

H -0.42446300 -2.54161600 -0.62462200

H -4.34473900 -1.76529200 0.70881900

O -1.00671700 3.00764500 -0.92978200

C -1.12212800 4.23248900 -0.20555900

H -0.68448800 4.98580300 -0.87066600

H -0.51204800 4.20810300 0.70897000

C -2.55399600 4.60059900 0.13620800

C -2.89884100 5.03918000 1.41878500

C -3.55167200 4.53025000 -0.84703500

C -4.21298000 5.41036800 1.71639600

H -2.13471100 5.09090000 2.19158400

C -4.86461000 4.89479400 -0.55180600

H -3.28891200 4.18283200 -1.84266500

C -5.19852500 5.33824700 0.73181800

H -4.46554700 5.74748600 2.71820100

H -5.62849400 4.83811900 -1.32312400

H -6.22170300 5.62343300 0.96145500

C -4.55503200 -3.31544600 -0.73990000

C -4.37443900 -3.61341100 -2.23746600

H -4.93829100 -2.90599200 -2.85480700

H -3.32548500 -3.56639400 -2.54911800

H -4.74179800 -4.62235200 -2.45136900

C -6.03177800 -3.39707600 -0.35645900

H -6.61007200 -2.61801000 -0.86550900

H -6.44715000 -4.36992000 -0.64003400

H -6.15257000 -3.27421100 0.72443800

O -3.87270200 -4.31969100 0.04510700

C -2.49898700 -3.97483500 0.13019200

H -1.93825400 -4.41695000 -0.70483200

C -1.93099700 -4.36087900 1.48778500

H -2.55518200 -5.09700200 1.99995200

H -0.91558500 -4.76755000 1.42294000

C -1.88331700 -3.06028000 2.28433300

O -2.14007800 -2.00675900 1.45737000

O -1.64845000 -2.91827200 3.45521700

H -3.66445100 -0.93508300 -2.15549600

H -5.10113800 -0.53077800 -1.23853100

H -3.86114700 1.37650400 -0.73070900

H -3.31346900 0.34857100 0.57731600

C 5.42518400 -0.69525600 1.12461100

H 5.51179000 -0.86364700 2.20653200

C 6.48417300 -1.44250600 0.36368700

H 6.55088200 -2.52552100 0.36486200

C 7.26243300 -0.58520600 -0.30497200

C 8.42394700 -0.80955000 -1.21707300

H 9.31497800 -0.28943000 -0.84592600

H 8.65501400 -1.87367400 -1.31947700

H 8.21411500 -0.39875700 -2.21169500

C 6.76533400 0.78691700 -0.01579400

O 5.71739600 0.68801900 0.86987300

O 7.16081500 1.84632800 -0.43859100

BF_3_·H_2_O

O 1.54375500 -0.03785700 0.00000000

H 1.83733100 0.46811300 0.77765600

B -0.31962100 -0.02961900 0.00000000

F -0.53431800 1.30521500 0.00000000

F -0.53431800 -0.67956700 -1.15486500

F -0.53431800 -0.67956700 1.15486500

H 1.83733100 0.46811300 -0.77765600

DBU

C -2.72505500 0.84734000 0.18185700

C -2.79526300 -0.60691700 -0.28435300

C -1.59380500 -1.35742800 0.27762000

H -3.72483000 -1.09152000 0.03779000

H -2.97299900 0.91290100 1.25319200

H -3.47771200 1.45390400 -0.33822600

H -1.68912100 -1.45442100 1.37346300

H -1.55014900 -2.37581600 -0.12848800

H -2.76459600 -0.64540200 -1.38047600

C 0.92091900 1.48140000 -0.41504900

C -0.37018100 0.72108300 -0.13411000

C 2.10044100 1.18766000 0.53141800

C 0.84279500 -1.47417000 0.27371700

C 2.95896200 -0.00473400 0.07884400

C 2.12692400 -1.15926700 -0.50422100

H 1.22916200 1.30895700 -1.45625600

H 1.04213400 -1.43349200 1.35973100

H 1.71187400 1.01276300 1.54338400

H 3.55255400 -0.36917900 0.92780300

H 0.63023900 2.53115300 -0.34568800

H 2.73317900 2.08021400 0.60638000

H 0.56437600 -2.51204200 0.05855900

H 3.68066100 0.32503000 -0.68018300

H 2.74243100 -2.06751300 -0.52436000

H 1.85328600 -0.95484600 -1.54603800

N -1.42230600 1.45903200 -0.03961500

N -0.34268200 -0.67767400 -0.07394600

**TS8**

C 2.44621600 0.59567900 -0.80301500

C 1.34759500 0.42451500 -1.90548300

C 1.96419500 -1.38793400 0.07238700

C -0.10177000 0.59581300 -1.37549600

C 0.86202400 -2.13416100 0.00355700

C -0.97981100 -0.66169400 -1.07933900

H 1.46410200 -0.55813300 -2.37179000

H -0.09722000 1.21306700 -0.47243100

H 1.54316300 1.15403400 -2.70184200

H -0.68203400 1.16048000 -2.11583400

H 0.98884200 -3.17849500 -0.26813200

C 3.69940300 -0.28001500 -1.14909800

H 3.74163300 -0.42706500 -2.23426900

C 3.34565700 -1.65893100 -0.49252800

O 1.91878400 -0.04290300 0.38717700

C -0.54857100 -1.60714300 0.08793700

H -1.21923800 -2.47348500 0.01177900

C -1.25757800 -1.43486700 -2.37094800

H -0.34014400 -1.87719000 -2.77449600

H -1.97143300 -2.24399300 -2.18372600

H -1.68204700 -0.77272200 -3.13509300

C -1.00036900 -0.82197700 1.33401700

H -0.28246400 -0.01273200 1.50468500

C -1.09745600 -1.65429700 2.61243100

H -0.11109000 -2.05304200 2.87674600

H -1.45478300 -1.04854900 3.45104100

H -1.79085000 -2.49310600 2.49253600

C -2.33987700 -0.18874000 0.84110800

H -2.40111400 0.84252500 1.22033700

O -2.24628000 -0.14947300 -0.61134400

H 4.06871100 -1.86201200 0.30900300

C 2.61388900 2.09942700 -0.52148100

C 3.66423300 2.64652900 0.45360600

C 5.14713900 2.62735100 -0.00752800

C 5.08380400 0.18711000 -0.66955600

C 5.49466000 1.60952500 -1.09657900

H 2.78356900 2.59908700 -1.48253200

H 1.63389200 2.45018100 -0.17384400

H 5.70894100 2.33970300 0.88873600

O 3.38383600 -2.71086100 -1.44688000

C 3.77588900 -3.97703500 -0.91770400

H 3.48400200 -4.69926100 -1.68908500

H 3.20504100 -4.21798800 -0.00950800

C 5.26369500 -4.08178600 -0.63774600

C 5.73451000 -4.61855600 0.56491300

C 6.19179600 -3.66362600 -1.60259800

C 7.10624500 -4.74577200 0.80066300

H 5.02387500 -4.93873500 1.32395200

C 7.56086100 -3.78434100 -1.36831100

H 5.82938000 -3.23815700 -2.53467400

C 8.02198600 -4.32823500 -0.16521600

H 7.45646000 -5.16265300 1.74120400

H 8.27033100 -3.45816000 -2.12463400

H 9.08916700 -4.42262800 0.01715200

C 5.49684600 4.12047800 -0.26380500

C 5.16986500 4.61956000 -1.68095200

H 5.83144100 4.16293600 -2.42497500

H 4.13720100 4.40138800 -1.97425400

H 5.30605000 5.70518200 -1.72248300

C 6.94411100 4.45445700 0.09508300

H 7.64162100 3.91741100 -0.55731400

H 7.12871600 5.52797700 -0.01851800

H 7.15158500 4.17854300 1.13364600

O 4.66845500 4.80682800 0.70169700

C 3.40671900 4.15956800 0.76320600

H 2.71159200 4.60210000 0.03643800

C 2.86185300 4.17837600 2.18316400

H 3.33494300 4.94801100 2.79738500

H 1.77822000 4.33799600 2.21729500

C 3.16335600 2.79078300 2.74194900

O 3.56105500 1.96735000 1.72873600

O 3.07291800 2.41514000 3.88013700

H 5.02634800 1.86979500 -2.05466100

H 6.57776100 1.64620000 -1.27064100

H 5.80789000 -0.54413800 -1.04983300

H 5.13160600 0.11771100 0.42420300

C -3.63954500 -0.87682500 1.16785200

H -4.88220600 0.06076100 0.58920500

C -4.04259400 -2.18831300 0.81893000

H -3.79240700 -2.66757600 -0.12298500

C -4.85971900 -2.70895200 1.80527400

C -5.64191100 -3.98683400 1.83226600

H -5.37946700 -4.60432300 2.70154100

H -6.72446600 -3.80524600 1.90392200

H -5.46041700 -4.58443200 0.93110000

C -4.93192700 -1.73209700 2.86369000

O -4.10249400 -0.66010500 2.49575600

O -5.60886000 -1.67758800 3.88741900

C -6.56204500 0.93592900 1.73317700

C -7.70491900 1.90263100 1.45451500

C -8.49416700 1.41373000 0.24380200

H -8.36609700 1.97223300 2.32409500

H -6.91426200 0.00767500 2.19636600

H -5.82543900 1.35277300 2.42383200

H -8.99879100 0.46340700 0.46984100

H -9.26602000 2.13834800 -0.03151600

H -7.31644000 2.90785600 1.25119800

C -5.39134400 0.46224800 -1.85240500

C -6.34961200 0.78140500 -0.71859300

C -5.98791800 -0.34529500 -3.01786100

C -8.31446000 1.28649100 -2.22648300

C -6.67424700 0.53822600 -4.07090100

C -7.47999000 1.68866600 -3.44650100

H -4.97259500 1.40808000 -2.22159600

H -8.81716100 0.32417400 -2.40794300

H -6.69376000 -1.08700100 -2.62242500

H -7.33305700 -0.08033800 -4.69357300

H -4.54508900 -0.06844200 -1.40772500

H -5.18459800 -0.92218200 -3.48881500

H -9.10740200 2.02838700 -2.09267800

H -5.92165900 0.96419600 -4.74649000

H -8.17005200 2.09513300 -4.19573700

H -6.82334200 2.51935400 -3.16171100

N -5.86137700 0.61754500 0.48664900

N -7.61127100 1.23839100 -0.92685700

DBUH^+^

C 2.80913500 -0.78025000 0.05578900

C 2.75615900 0.69466600 -0.31729800

C 1.58086000 1.35920300 0.39251400

H 3.68577900 1.19072900 -0.02539100

H 3.15751700 -0.92286100 1.08545700

H 3.47520100 -1.33911700 -0.60689200

H 1.73436900 1.38666700 1.47868100

H 1.46460000 2.38872800 0.04921300

H 2.64632600 0.80650800 -1.40142700

C -0.93541700 -1.50624600 -0.28684200

C 0.30895400 -0.66466700 -0.06970400

C -2.15369600 -1.13893900 0.58157500

C -0.91167200 1.51468700 0.23216300

C -2.98677500 0.00498000 -0.01488500

C -2.11955100 1.11909100 -0.62023800

H -1.19654800 -1.46383500 -1.35245700

H -1.18024100 1.58243700 1.29403400

H -1.81276300 -0.88602400 1.59319000

H -3.63471000 0.41917200 0.76589700

H -0.64878800 -2.54395200 -0.08859000

H -2.77469100 -2.03340200 0.69135100

H -0.57588200 2.50740700 -0.07453800

H -3.65184200 -0.38561200 -0.79380000

H -2.72687600 2.02170300 -0.74918600

H -1.77236900 0.85288500 -1.62621600

N 1.45949300 -1.34582700 -0.07714800

N 0.30275600 0.65276700 0.11378700

H 1.38719700 -2.34516000 -0.21424000

**CP9**

C -1.00247700 -0.53616700 -0.98024300

C -0.03842900 -0.55757500 -2.21340300

C 0.14446500 1.09905200 -0.01152100

C 1.34179900 -1.20166000 -1.91891900

C 1.39846500 1.50460700 -0.21150700

C 2.58788800 -0.28877700 -1.67702100

H 0.09800500 0.46690000 -2.57116200

H 1.26490600 -1.87979500 -1.06451200

H -0.54298700 -1.09019600 -3.03116600

H 1.62896500 -1.83037400 -2.77070900

H 1.54520300 2.57075300 -0.36921000

C -1.96366100 0.70247800 -1.07017400

H -2.04026100 1.01216900 -2.11889900

C -1.16191600 1.80960300 -0.30521200

O -0.18320000 -0.23459200 0.17484700

C 2.59766900 0.61079800 -0.39591800

H 3.47401200 1.26156800 -0.51808400

C 2.90893600 0.51685500 -2.94164400

H 2.11776000 1.24068100 -3.17345400

H 3.84743700 1.06276100 -2.80672300

H 3.02760200 -0.15266700 -3.80178600

C 2.99529600 -0.39393200 0.69921100

H 2.14180000 -1.05292600 0.90173300

C 3.45053600 0.23757700 2.01311300

H 2.62239500 0.78205600 2.48484200

H 3.80580500 -0.52697300 2.71167100

H 4.28238100 0.92858400 1.84539800

C 4.09414600 -1.21495000 -0.05347900

H 4.05131700 -2.25857000 0.29884400

O 3.67961500 -1.18968600 -1.45054500

H -1.69786200 2.05175900 0.62381300

C -1.57958100 -1.95200700 -0.79956000

C -2.67433800 -2.28232100 0.22131900

C -4.11660800 -1.79944400 -0.09588400

C -3.38684200 0.58577700 -0.49978700

C -4.22330000 -0.59422700 -1.03352500

H -1.95334000 -2.28714900 -1.77418100

H -0.71820000 -2.59302600 -0.57487600

H -4.51829900 -1.48293000 0.87407100

O -1.03525700 2.98654300 -1.09537200

C -0.89267700 4.18338200 -0.33824400

H -0.47832100 4.91438100 -1.04344400

H -0.15743600 4.05586300 0.46846500

C -2.20339000 4.69932900 0.23080400

C -2.26972000 5.22292100 1.52639500

C -3.36432000 4.69181800 -0.55552700

C -3.46807700 5.73826100 2.02738500

H -1.37837600 5.22306300 2.15022800

C -4.56308300 5.20001300 -0.05653700

H -3.31710000 4.27495600 -1.55775500

C -4.61816600 5.72773900 1.23707800

H -3.50362900 6.13697800 3.03804700

H -5.45614700 5.18667000 -0.67679800

H -5.55285600 6.12293800 1.62679300

C -4.89060900 -3.09413500 -0.46624500

C -4.79924700 -3.49325000 -1.94851800

H -5.33554200 -2.78127400 -2.58554900

H -3.76456900 -3.54443100 -2.30315300

H -5.24856800 -4.48254000 -2.08595000

C -6.35151700 -3.05253200 -0.01856500

H -6.90085600 -2.26987000 -0.55387000

H -6.84165500 -4.01175200 -0.21813500

H -6.41380200 -2.85280500 1.05579300

O -4.24286500 -4.09322500 0.35276400

C -2.84479600 -3.83074000 0.37577600

H -2.34170300 -4.37027200 -0.43758300

C -2.26014200 -4.15001400 1.74309300

H -2.89096500 -4.83215700 2.31740400

H -1.25764100 -4.58787200 1.67683300

C -2.14752000 -2.80124600 2.44753700

O -2.30622100 -1.79897500 1.53758000

O -1.94255500 -2.59028900 3.61370000

H -3.91222700 -0.84990500 -2.05473800

H -5.27975500 -0.30267000 -1.10348700

H -3.89256000 1.53491000 -0.71827200

H -3.33529500 0.51698700 0.59370000

C 5.49220900 -0.72382800 0.06738100

C 6.29265100 0.07908600 -0.69968000

H 6.01320600 0.50763800 -1.65698600

C 7.55386800 0.20355900 -0.04606200

C 8.77693300 0.94650900 -0.48578000

H 9.19398800 0.59400400 -1.44728000

H 8.62013200 2.03456500 -0.60467700

H 9.55737600 0.81312600 0.27368900

C 7.50007500 -0.55049700 1.14820800

O 6.18113200 -1.13262800 1.19408400

O 8.29316900 -0.78534100 2.07232400

**TS9**

C -2.40657100 -0.46688000 -0.85481700

C -1.27185700 -0.25619100 -1.91232200

C -1.71858900 1.28185100 0.33429300

C 0.14919900 -0.65000200 -1.42396100

C -0.53270300 1.88524000 0.42173500

C 1.15350600 0.43914500 -0.90677900

H -1.27994800 0.78877500 -2.23490800

H 0.07743300 -1.42577000 -0.65502900

H -1.53033500 -0.83937100 -2.80614600

H 0.68464100 -1.11729100 -2.25971600

H -0.51831400 2.96743600 0.32420900

C -3.53960400 0.59614800 -1.06384500

H -3.54204100 0.91033900 -2.11365100

C -3.03800200 1.80522400 -0.20091800

O -1.84265200 -0.09199600 0.42640700

C 0.79350000 1.16247100 0.44156000

H 1.57723200 1.92398900 0.55982900

C 1.39570700 1.47878700 -2.01713200

H 0.48483000 2.03582400 -2.26331200

H 2.14988300 2.20993400 -1.70418700

H 1.75567400 0.98668700 -2.92853100

C 1.02156200 0.13275300 1.57550600

H 0.14877800 -0.52530800 1.59412900

C 1.12326900 0.80118800 2.95483200

H 0.20261700 1.35742400 3.16486300

H 1.25322000 0.05170000 3.74282300

H 1.97177000 1.48895700 3.00786200

C 2.22735400 -0.75152600 1.22423500

H 1.96292700 -1.78610400 1.01892800

O 2.35539000 -0.23315100 -0.61470800

H -3.75502300 1.97625200 0.61354200

C -2.75650100 -1.96481500 -0.81582100

C -3.87977400 -2.52911900 0.06291700

C -5.34190600 -2.25777700 -0.38406100

C -4.97925600 0.23014100 -0.66429400

C -5.54639200 -1.05164900 -1.30412500

H -2.96984800 -2.28150600 -1.84353000

H -1.83037300 -2.48125000 -0.53211300

H -5.87850500 -2.04143200 0.54711500

O -2.91646600 2.98683800 -0.98275400

C -3.17196300 4.19513500 -0.26966100

H -2.76706400 4.98551100 -0.91273200

H -2.61016600 4.22128600 0.67514200

C -4.64592600 4.44049800 -0.00266900

C -5.08731500 4.88113300 1.24925700

C -5.58676900 4.25452200 -1.02579800

C -6.44138200 5.14143400 1.47725600

H -4.36768100 5.01976800 2.05342000

C -6.93913500 4.50805000 -0.79984200

H -5.24837700 3.90314000 -1.99684300

C -7.37016900 4.95470900 0.45323500

H -6.76898600 5.48045800 2.45647700

H -7.65856200 4.36089700 -1.60144200

H -8.42420500 5.15242700 0.62918900

C -5.86756000 -3.64003000 -0.86437500

C -5.58720000 -3.95190400 -2.34394200

H -6.17974000 -3.30971400 -3.00446100

H -4.53234400 -3.81908200 -2.60806900

H -5.85371700 -4.99365300 -2.55000400

C -7.34893200 -3.84536200 -0.55099300

H -7.96738700 -3.13204400 -1.10724000

H -7.66299300 -4.85742400 -0.82782200

H -7.53328500 -3.70989200 0.51926900

O -5.14029000 -4.56330000 -0.02278100

C -3.81016900 -4.09139200 0.12849700

H -3.16591000 -4.49489200 -0.66475200

C -3.28681700 -4.39936500 1.52279400

H -3.85554700 -5.19368300 2.01172200

H -2.23060300 -4.69134100 1.52200000

C -3.42653600 -3.09120700 2.29616000

O -3.71667400 -2.07793700 1.43037800

O -3.30038600 -2.91341300 3.47858700

H -5.09479000 -1.21686800 -2.29085600

H -6.62296100 -0.93215800 -1.48148700

H -5.60522900 1.09122900 -0.92960900

H -5.04118300 0.13820600 0.42722300

C 3.49512100 -0.68206700 1.80669700

C 4.45042100 -1.69279700 1.99650400

H 4.26347400 -2.74623400 1.81138900

C 5.62520200 -1.13192200 2.47287200

C 6.90819200 -1.78262300 2.89186000

H 7.00700300 -1.84327800 3.98534000

H 6.98881500 -2.80397500 2.50069500

H 7.77356100 -1.20766800 2.53755500

C 5.40937700 0.28953300 2.55076200

O 4.08621800 0.53598600 2.15227300

O 6.15646200 1.22236900 2.84714700

H 3.73964100 0.61608300 -0.70509500

C 4.80533400 2.46654300 -0.31911500

C 6.25631900 2.79176100 0.00953600

C 7.15951800 2.27309100 -1.10425900

H 6.38670700 3.87448600 0.10843600

H 4.39818200 3.14612200 -1.07979700

H 4.18527400 2.53407100 0.57766000

H 6.99290400 2.82193000 -2.04306000

H 8.21000700 2.40211800 -0.83132300

H 6.52089000 2.32912800 0.96687500

C 5.31410400 -1.09587400 -1.54956500

C 5.68868100 0.33919400 -1.22609200

C 5.97750600 -1.69397800 -2.80030400

C 8.11495400 0.10481500 -1.86943400

C 7.37116500 -2.27461300 -2.51580900

C 8.19047700 -1.39351800 -1.56101600

H 5.53516400 -1.70215400 -0.66189100

H 8.20011000 0.28044700 -2.95246300

H 6.03562800 -0.93009700 -3.58703000

H 7.91381600 -2.40506700 -3.46097800

H 4.22428300 -1.10717800 -1.63858500

H 5.32550600 -2.47956900 -3.19799900

H 8.97695800 0.59343500 -1.40703600

H 7.27357400 -3.27427600 -2.07412600

H 9.24644600 -1.68448300 -1.61392600

H 7.88597800 -1.55033600 -0.51984800

N 4.68976000 1.09177700 -0.81115700

N 6.93777500 0.83098500 -1.34596300

**CP10**

C 2.27968300 0.76617500 -0.56151400

C 1.10563400 0.92447300 -1.58203600

C 1.61730300 -1.29222900 -0.05155800

C -0.29876500 1.08849700 -0.94289500

C 0.42860400 -1.89194200 -0.05733000

C -1.26133500 -0.13441200 -0.74762200

H 1.10547400 0.07019600 -2.26547200

H -0.22557700 1.60742500 0.01851100

H 1.32206300 1.80020100 -2.20802000

H -0.87378700 1.76329300 -1.59002400

H 0.35521300 -2.87243300 -0.51914400

C 3.39811200 -0.13947400 -1.17181400

H 3.41727500 -0.00894000 -2.25950000

C 2.86862300 -1.58177700 -0.86369400

O 1.77107800 -0.04638400 0.52361700

C -0.86311000 -1.20362500 0.34332100

H -1.65710300 -1.95722200 0.27851800

C -1.49083900 -0.85229800 -2.09122400

H -0.58718100 -1.34875900 -2.45842400

H -2.27755700 -1.60659000 -1.97938000

H -1.82239100 -0.13684500 -2.85320500

C -0.92576000 -0.65869100 1.82200800

H -0.44226000 0.32163400 1.83165000

C -0.13307700 -1.53977200 2.81210400

H 0.94026300 -1.42159000 2.64907700

H -0.35366200 -1.24417800 3.84304700

H -0.36880800 -2.60475800 2.70881100

C -2.35973800 -0.43203500 2.25520800

H -2.72549500 0.58715200 2.20571000

O -2.48899400 0.41650600 -0.28501400

H 3.62068700 -2.12089300 -0.27246000

C 2.63910800 2.15666100 -0.01208200

C 3.75176600 2.36742300 1.02310700

C 5.22090300 2.23215800 0.53904700

C 4.83005600 0.01695300 -0.63339500

C 5.43296000 1.43069200 -0.74806800

H 2.87258900 2.80014100 -0.86874200

H 1.71360400 2.55988200 0.41908300

H 5.72589300 1.68462300 1.34318300

O 2.60663100 -2.30271000 -2.06346600

C 2.83740600 -3.70688800 -1.97993600

H 2.34806300 -4.11866700 -2.87048000

H 2.33469100 -4.13893900 -1.10234500

C 4.30821700 -4.08331500 -1.96943000

C 4.78649000 -5.08432000 -1.11731000

C 5.20438200 -3.45279700 -2.84409800

C 6.13235600 -5.45928400 -1.14218700

H 4.10211200 -5.57416200 -0.42765600

C 6.54939200 -3.82032600 -2.86668500

H 4.83719100 -2.66906000 -3.50090500

C 7.01693500 -4.82717800 -2.01649600

H 6.48916000 -6.23736400 -0.47256300

H 7.23418200 -3.32444800 -3.54994300

H 8.06505000 -5.11367800 -2.03462800

C 5.78488700 3.68146100 0.59200700

C 5.56140300 4.49949600 -0.69070200

H 6.17024400 4.11723100 -1.51701600

H 4.51519200 4.49112800 -1.01582900

H 5.84633100 5.54093400 -0.50918600

C 7.25686600 3.72314600 0.99940900

H 7.88331100 3.23239200 0.24618000

H 7.59699900 4.75898400 1.10327900

H 7.40016100 3.21700000 1.95920300

O 5.04191800 4.26991200 1.68322200

C 3.69943900 3.81512800 1.61570400

H 3.09644600 4.48293600 0.98516000

C 3.12171400 3.64343900 3.01165700

H 3.67397600 4.21553300 3.76079900

H 2.06788900 3.93797300 3.07160100

C 3.22524800 2.14890200 3.30034900

O 3.55104600 1.48416200 2.15493500

O 3.04766900 1.58166400 4.34574900

H 5.01413400 1.95168200 -1.61903600

H 6.51240900 1.35665500 -0.93246000

H 5.45161900 -0.69987600 -1.18449600

H 4.86001700 -0.29998800 0.41687200

C -3.26722100 -1.34724100 2.63845100

C -3.24824600 -2.78799000 2.80003400

H -2.37315600 -3.40393100 2.64212400

C -4.47868400 -3.21052800 3.16995200

C -4.99852800 -4.57923900 3.46268800

H -5.84134500 -4.82424200 2.80484400

H -4.22219800 -5.33923400 3.33393800

H -5.37837300 -4.64067600 4.48999700

C -5.34486000 -2.01534600 3.23998000

O -4.56665800 -0.91554900 2.93318500

O -6.52700800 -1.92129100 3.48836000

H -3.02434200 0.80466800 -1.02846200

C -4.00980100 2.40406800 -3.17794700

C -5.09667500 2.50323900 -4.24463200

C -6.43080300 2.75121000 -3.55292600

H -4.88933200 3.30902100 -4.95836900

H -3.82354400 3.39550400 -2.73574000

H -3.05933700 2.08596000 -3.62526700

H -6.45076200 3.76540800 -3.11960500

H -7.25388900 2.69829900 -4.27587800

H -5.14699600 1.56405900 -4.80927500

C -5.82342700 0.18825100 -0.73227800

C -5.59274900 1.20744200 -1.84037000

C -6.77803000 0.63075200 0.39525500

C -8.05597800 1.81392600 -1.98585600

C -8.24999500 0.30465500 0.09758300

C -8.62829100 0.52917100 -1.37544400

H -6.18741200 -0.74721400 -1.18026800

H -8.14932500 2.64544500 -1.26694300

H -6.65519300 1.70874000 0.56585900

H -8.89584500 0.91507800 0.74209400

H -4.83659200 -0.03056500 -0.32319000

H -6.47893700 0.14590600 1.32952500

H -8.67616700 2.08630500 -2.84685100

H -8.45628600 -0.74027100 0.36176900

H -9.72105500 0.57681400 -1.46433600

H -8.31198300 -0.31746900 -1.99646400

N -4.35070600 1.46178700 -2.11893400

N -6.68090300 1.74587500 -2.51306500

**CP11**

C 1.75107100 -0.06165000 -0.74482800

C 0.81286000 -1.14604600 -1.36824900

C 2.07750700 -1.32822400 1.05399600

C -0.57828000 -1.26608600 -0.68341300

C 1.34193800 -2.26283400 1.65653000

C -0.83369400 -2.37737400 0.38812700

H 1.32157200 -2.11340200 -1.35584000

H -0.87900100 -0.30683500 -0.24846100

H 0.67391200 -0.90167000 -2.42941000

H -1.31267100 -1.47742000 -1.47020600

H 1.83055700 -3.20019600 1.90649800

C 3.24884400 -0.48603400 -0.86210100

H 3.40640600 -1.04519600 -1.79098900

C 3.41619800 -1.47015600 0.34493800

O 1.53692000 -0.10640000 0.68586000

C -0.16795400 -2.17667100 1.80367800

H -0.50059900 -3.05939400 2.36648200

C -0.42117300 -3.75978000 -0.16482100

H 0.65869300 -3.86793000 -0.30506600

H -0.76014900 -4.53960100 0.52575400

H -0.91078100 -3.93427000 -1.13123100

C -0.60141800 -0.94016100 2.66202200

H -0.11187200 -0.05571400 2.25008000

C -0.07921400 -1.13349600 4.10727100

H 1.00286100 -1.30412700 4.10875100

H -0.28956800 -0.24731900 4.71473800

H -0.56269500 -1.99318300 4.58848000

C -2.08629900 -0.69834500 2.71328700

H -2.74394900 -1.55335600 2.81595700

O -2.22976800 -2.41331500 0.65475400

H 4.25209600 -1.13327200 0.97170300

C 1.31056900 1.30243200 -1.30567900

C 1.93172000 2.63192200 -0.86574900

C 3.39011400 2.93891400 -1.29910200

C 4.30853800 0.62563900 -0.75861300

C 4.21158500 1.74946900 -1.80559600

H 1.41763500 1.24694900 -2.39604700

H 0.23100100 1.37637100 -1.11973400

H 3.88142500 3.30735800 -0.39133100

O 3.66893500 -2.80081900 -0.09782500

C 4.56834300 -3.54501200 0.71952700

H 4.43093200 -4.58563400 0.40279400

H 4.27995000 -3.48650300 1.77928600

C 6.02144000 -3.13614300 0.55511500

C 6.87398800 -3.05573900 1.66134300

C 6.53908400 -2.86960400 -0.72021400

C 8.22228600 -2.72626600 1.49987600

H 6.48157100 -3.25198500 2.65708200

C 7.88277800 -2.53401300 -0.88371800

H 5.87684300 -2.92331600 -1.57982900

C 8.72941200 -2.46385400 0.22677900

H 8.87184800 -2.66656900 2.36915400

H 8.27231800 -2.33056200 -1.87798300

H 9.77665300 -2.20319800 0.09913400

C 3.26411800 4.16321900 -2.25733500

C 3.08276200 3.78981500 -3.73804900

H 3.99680600 3.35320700 -4.15392400

H 2.27043900 3.07054300 -3.89125200

H 2.84321500 4.69203100 -4.31003200

C 4.41344400 5.15622700 -2.09383600

H 5.36853500 4.69447600 -2.36813900

H 4.26027000 6.03036000 -2.73545500

H 4.47468400 5.49834900 -1.05601900

O 2.07498200 4.82932100 -1.77752200

C 1.11912200 3.84167500 -1.43243500

H 0.52346700 3.54895700 -2.30807900

C 0.26497400 4.31554500 -0.26885600

H 0.25954700 5.40392100 -0.17300200

H -0.77492100 3.97731200 -0.34101700

C 0.89754800 3.67464900 0.95959700

O 1.82906000 2.76378800 0.57940900

O 0.63568600 3.88478600 2.11918300

H 3.80542400 1.35343500 -2.74518400

H 5.21984200 2.11215800 -2.04437900

H 5.28593500 0.13295500 -0.83583600

H 4.26988600 1.06786400 0.24555300

C -2.64480200 0.52408400 2.68223100

C -2.06898800 1.85205700 2.61656500

H -1.00681500 2.06291100 2.59827900

C -3.06877100 2.76443300 2.62848800

C -3.01475800 4.25731100 2.61045000

H -3.46481400 4.67460800 3.52003500

H -1.98116000 4.60736600 2.53952100

H -3.59198100 4.66013400 1.76854600

C -4.34149900 2.01522300 2.67228200

O -4.03459700 0.66550600 2.72959000

O -5.48654200 2.41406600 2.64566400

H -2.76089400 -2.47114400 -0.18348700

C -4.14614700 -3.64709700 -2.38150200

C -5.57533000 -4.14864300 -2.57147900

C -6.46117100 -2.96378200 -2.93794600

H -5.63269000 -4.91508600 -3.35315100

H -3.70636000 -3.38576900 -3.35707700

H -3.51214600 -4.43866500 -1.96294300

H -6.21402600 -2.60257300 -3.95042100

H -7.51516300 -3.26540700 -2.95646300

H -5.93712100 -4.59765400 -1.63848700

C -4.89329300 -0.56730400 -0.35295300

C -5.09227100 -1.70727500 -1.34123300

C -5.31157000 0.83106200 -0.84437700

C -7.37472100 -0.85627000 -2.07381600

C -6.78621100 1.14623400 -0.55329100

C -7.70514000 -0.06042600 -0.80412100

H -5.41452600 -0.79847900 0.58466600

H -7.14925900 -0.16525800 -2.90377800

H -5.10366200 0.91788100 -1.91990800

H -7.10951600 1.99217300 -1.17477300

H -3.83262400 -0.58385800 -0.10520400

H -4.68209100 1.58181900 -0.35415600

H -8.27300800 -1.40658700 -2.37392100

H -6.88554100 1.46645100 0.49045300

H -8.74223400 0.28739200 -0.89017200

H -7.68790400 -0.74893600 0.04877700

N -4.06802300 -2.49176900 -1.49488600

N -6.32014700 -1.87995900 -1.95868300

**TS10**

C 2.28488200 0.59551000 -0.49112900

C 1.59049200 -0.43601900 -1.43923700

C 2.38062200 -0.92396800 1.12357700

C 0.09352500 -0.67657900 -1.09158400

C 1.63177200 -1.99726600 1.37886500

C -0.37417000 -1.92750700 -0.26686600

H 2.14479500 -1.37857100 -1.42079100

H -0.32116700 0.20371700 -0.59093600

H 1.67588400 -0.06243700 -2.46812400

H -0.45891900 -0.76827600 -2.03662000

H 2.14679500 -2.93303200 1.57634500

C 3.81172200 0.30533800 -0.35273100

H 4.21051100 -0.07970700 -1.29793100

C 3.84195400 -0.84841900 0.70652200

O 1.80458700 0.30468400 0.84455500

C 0.13233100 -2.04270000 1.22255700

H -0.19546000 -3.04369000 1.53796700

C -0.02139100 -3.21677800 -1.03143500

H -0.42021100 -3.17724700 -2.05232800

H 1.06173700 -3.37929000 -1.09246100

H -0.47025100 -4.08022500 -0.52846000

C -0.69525600 -1.04597100 2.06301000

H -0.33445300 -0.03350200 1.86553800

C -0.57412600 -1.31319400 3.57231000

H 0.46965500 -1.22812100 3.89416000

H -1.17006500 -0.59280600 4.14175800

H -0.92766600 -2.32156700 3.82517600

C -2.13220100 -1.16599500 1.60521100

O -1.77188800 -1.83519600 -0.13757200

H 4.48905600 -0.55447800 1.54684500

C 1.83459300 1.99982700 -0.93044400

C 2.19770500 3.27240900 -0.15821100

C 3.67019900 3.76015100 -0.20851000

C 4.70339000 1.45956300 0.14194300

C 4.70117900 2.74259100 -0.70888900

H 2.18354700 2.13757100 -1.96164800

H 0.73959000 1.96834800 -0.99256000

H 3.92513700 3.99399100 0.83135800

O 4.34700500 -2.04578900 0.13357200

C 5.02367200 -2.90812100 1.03777900

H 4.33054200 -3.33492600 1.77796500

H 5.77125500 -2.32496100 1.60446000

C 5.70556000 -4.02037600 0.27331900

C 5.85242400 -5.28326600 0.85832800

C 6.23825000 -3.79770000 -1.00215400

C 6.52891800 -6.30427400 0.18850600

H 5.43221800 -5.47153200 1.84424500

C 6.90680700 -4.81979700 -1.67668500

H 6.11103400 -2.82459400 -1.46516000

C 7.05785400 -6.07514000 -1.08282000

H 6.63343000 -7.27986900 0.65603100

H 7.31039500 -4.63584700 -2.66917600

H 7.57909900 -6.87016800 -1.60943800

C 3.60404900 5.12443600 -0.96237100

C 3.77545100 5.01195500 -2.48663100

H 4.80054300 4.73448100 -2.75380400

H 3.10303300 4.26812900 -2.92847700

H 3.55214700 5.98042700 -2.94577000

C 4.57316200 6.15845900 -0.39153100

H 5.61135500 5.83308000 -0.52199100

H 4.45105400 7.12085100 -0.89975500

H 4.38353400 6.30743600 0.67606500

O 2.27367400 5.60020700 -0.65962200

C 1.38644900 4.49558700 -0.69569000

H 1.02524900 4.31796800 -1.71832200

C 0.25751700 4.68240900 0.30438100

H 0.11978500 5.73039700 0.58167900

H -0.70265500 4.30195900 -0.06117900

C 0.67619400 3.86474400 1.52236600

O 1.78869900 3.13666100 1.22725300

O 0.13914600 3.81833800 2.59895900

H 4.53985600 2.49404500 -1.76549100

H 5.69209500 3.21231200 -0.65980400

H 5.72459000 1.06072100 0.19991300

H 4.41824800 1.71547500 1.17077100

C -2.96972800 -0.04730500 1.60658400

C -2.75539600 1.33756200 1.67533900

H -1.79841800 1.81190200 1.86485300

C -3.97030700 1.99412500 1.52581100

C -4.26982300 3.46091800 1.59567800

H -4.73981500 3.83128700 0.67358200

H -4.96795800 3.69405500 2.41077800

H -3.35474900 4.03859700 1.76721900

C -4.97611400 0.98584300 1.32792600

O -4.34099200 -0.25420400 1.40069700

O -6.18941400 1.04976300 1.09321500

H -2.62209800 -2.10125500 1.86615100

H -2.91017400 -1.25576100 -1.17447800

C -3.65228200 0.54020500 -2.08425600

C -4.88868900 0.90304900 -2.89522300

C -6.13639100 0.56745300 -2.08572600

H -4.89039400 1.97042100 -3.13838000

H -3.53874000 1.20507800 -1.21835200

H -2.74023300 0.60898800 -2.68565200

H -6.25177900 1.22709900 -1.21769400

H -7.02856100 0.66296500 -2.71338500

H -4.89101600 0.34605100 -3.84021100

C -4.81101200 -2.82368900 -0.76360100

C -4.93019600 -1.41783000 -1.32546500

C -5.69326800 -3.13852000 0.45897100

C -7.40632900 -1.25154000 -1.01242300

C -7.12165500 -3.56643000 0.09167200

C -7.71152100 -2.75180100 -1.07089400

H -5.02085600 -3.52909200 -1.57992500

H -7.46175900 -0.84863800 0.00634100

H -5.70180400 -2.26335400 1.11480500

H -7.76314600 -3.46266200 0.97567300

H -3.75676000 -2.95217100 -0.50379300

H -5.20800400 -3.94037200 1.02757000

H -8.15656900 -0.72302400 -1.60708200

H -7.13989400 -4.63000800 -0.18094900

H -8.80233700 -2.86656900 -1.07464100

H -7.36709700 -3.14293000 -2.03686800

N -3.77435100 -0.83757100 -1.61637300

N -6.10487600 -0.82729700 -1.58804900

**CP12**

C 0.26558800 0.59692200 -0.65844900

C 0.40809400 -0.38442900 -1.86676700

C 1.41665700 -0.75456900 0.68053300

C -0.50800500 -1.63002700 -1.78195300

C 1.63410100 -2.06782700 0.59512400

C 0.06494700 -2.99681000 -1.27835000

H 1.45592500 -0.69085000 -1.95114000

H -1.39913500 -1.41504600 -1.18833500

H 0.19338900 0.18182300 -2.78354000

H -0.88740800 -1.85729300 -2.78541800

H 2.66064800 -2.41177900 0.69698600

C 1.62425600 1.35315000 -0.43784100

H 2.18833900 1.33347700 -1.37739500

C 2.36946300 0.42099600 0.57821000

O 0.15163400 -0.22187500 0.53258900

C 0.58860200 -3.08460000 0.20172300

H 1.06159200 -4.07491900 0.26007200

C 1.10276500 -3.53726200 -2.26889100

H 0.67027600 -3.59912400 -3.27403700

H 1.99315800 -2.89761200 -2.31413200

H 1.41506700 -4.54501300 -1.97395100

C -0.71849100 -3.16519300 1.02256700

H -1.05783400 -2.14946900 1.22675200

C -0.57329700 -3.89716200 2.35816400

H 0.12179600 -3.37307200 3.02660000

H -1.54520700 -3.95906100 2.86105600

H -0.20078800 -4.92062700 2.21416000

C -1.72005900 -3.85316200 0.06017700

O -1.01836300 -3.91415100 -1.26321300

H 2.44207600 0.94244000 1.54624200

C -1.05347800 1.37397200 -0.80839200

C -1.42471000 2.53970100 0.11474100

C -0.67388900 3.88300200 -0.09970500

C 1.60548400 2.80495800 0.06930200

C 0.71489300 3.77479200 -0.73465600

H -1.10681700 1.75609500 -1.83429300

H -1.84827900 0.62287100 -0.72740900

H -0.52817400 4.28029000 0.91197800

O 3.67944300 0.11883100 0.11974100

C 4.59663400 -0.20976100 1.14592100

H 4.31802000 -1.15188100 1.64242700

H 4.57419300 0.57283600 1.92603600

C 5.99618200 -0.32240400 0.58042700

C 6.33654200 0.25735800 -0.64617700

C 6.98688500 -0.98800900 1.31368900

C 7.64462800 0.17392500 -1.12819800

H 5.56593800 0.75978600 -1.22023600

C 8.29541100 -1.06570300 0.83659500

H 6.73049500 -1.45264200 2.26403600

C 8.62904600 -0.48399000 -0.38897200

H 7.89354000 0.62312500 -2.08663900

H 9.05178500 -1.58794600 1.41731400

H 9.64647500 -0.54898300 -0.76635700

C -1.72638800 4.81377900 -0.76146400

C -1.81065800 4.69992200 -2.29308500

H -0.90455400 5.08801100 -2.77200000

H -1.94876800 3.66632200 -2.62559000

H -2.66560100 5.28234400 -2.65260100

C -1.55775000 6.27656900 -0.34957700

H -0.60552400 6.67836900 -0.71459900

H -2.36773700 6.88650700 -0.76447300

H -1.58353300 6.36942800 0.74085800

O -2.95942500 4.36277500 -0.16353300

C -2.92741700 2.94028700 -0.06076400

H -3.36226800 2.48480400 -0.95908500

C -3.60485300 2.47182700 1.21806400

H -4.29049100 3.21837200 1.62533400

H -4.16439600 1.54037700 1.07188000

C -2.46404400 2.20237000 2.19047600

O -1.28304700 2.15528800 1.50479600

O -2.51795800 2.03876000 3.38138400

H 0.65516200 3.45472300 -1.78285200

H 1.16991300 4.77428400 -0.74906800

H 2.64660200 3.15479100 0.05880700

H 1.28728200 2.82281100 1.11879000

C -3.04608800 -3.20987500 0.03314000

C -3.53221300 -1.99209800 0.45925100

H -2.95219800 -1.22477300 0.96498000

C -4.91728700 -1.93160000 0.16638100

C -5.90210000 -0.83945100 0.44252400

H -5.95438000 -0.54580400 1.50602500

H -5.71537100 0.09282900 -0.12692200

H -6.90071500 -1.18775800 0.15142500

C -5.27739100 -3.15741000 -0.45302700

O -4.08508500 -3.93937200 -0.51841900

O -6.33864700 -3.61858000 -0.89408100

H -1.83325900 -4.91960700 0.29975900

**TS11**

C 0.26670800 0.52220700 -0.62105500

C 0.61977700 -0.41984900 -1.81541000

C 1.52655700 -0.67787900 0.75777900

C -0.16969300 -1.74826900 -1.79092800

C 1.87706700 -1.96456200 0.71741400

C 0.44144000 -3.05026700 -1.18314900

H 1.70013000 -0.60187200 -1.82661600

H -1.13723700 -1.59720700 -1.31644900

H 0.39959100 0.12360800 -2.74415500

H -0.41966500 -2.02649700 -2.82118600

H 2.92295600 -2.22063000 0.86633600

C 1.47243500 1.47100400 -0.31485800

H 2.04867800 1.62693700 -1.23425800

C 2.34474600 0.59994900 0.65705900

O 0.21545900 -0.31166300 0.56229100

C 0.92515800 -3.06807000 0.31240300

H 1.47645500 -4.01443200 0.40114100

C 1.52067500 -3.60778400 -2.11654100

H 1.11306800 -3.75186000 -3.12317900

H 2.37337600 -2.92103600 -2.18474400

H 1.88282600 -4.57638300 -1.75416200

C -0.38663000 -3.24683300 1.12037500

H -0.78691800 -2.26860800 1.38686000

C -0.19364900 -4.06816300 2.39815600

H 0.52729100 -3.58694100 3.07184600

H -1.13823600 -4.17309000 2.94532600

H 0.17656100 -5.07786300 2.17207100

C -1.35905800 -3.90579300 0.08245500

O -0.61612500 -4.01723000 -1.15152100

H 2.40092400 1.10474100 1.63481500

C -1.13741700 1.09544600 -0.84672900

C -1.77292400 2.08139200 0.13607200

C -1.25112700 3.54279700 0.11337400

C 1.17726100 2.84561300 0.31335300

C 0.16307900 3.73071300 -0.44563400

H -1.15369000 1.57127700 -1.83465200

H -1.81203400 0.23580800 -0.92197000

H -1.21444500 3.83844700 1.16833000

O 3.66387400 0.44253100 0.14860200

C 4.65029000 0.18988500 1.12966000

H 4.52507400 -0.80815500 1.57718100

H 4.54553500 0.91730600 1.95529700

C 6.03241500 0.30806600 0.52293300

C 6.25182700 1.03428800 -0.65237100

C 7.12572000 -0.28541500 1.16652900

C 7.54142300 1.16579500 -1.17155000

H 5.40277500 1.48056800 -1.15819600

C 8.41565600 -0.14833700 0.65306800

H 6.96529000 -0.86297700 2.07509100

C 8.62800400 0.57889600 -0.52054800

H 7.69605300 1.72754600 -2.08963300

H 9.25301200 -0.61701400 1.16430500

H 9.63119200 0.68129600 -0.92672600

C -2.42414000 4.35542900 -0.50527400

C -2.40746400 4.42682700 -2.04239700

H -1.56581100 5.02883200 -2.40437200

H -2.33495700 3.43684600 -2.50372500

H -3.33666700 4.88908200 -2.39248200

C -2.54081700 5.76045300 0.08654700

H -1.65696800 6.36213800 -0.15627800

H -3.42354700 6.27199400 -0.31300000

H -2.64079100 5.70505100 1.17529500

O -3.58292100 3.62596200 -0.05438800

C -3.29926800 2.22795600 -0.15532200

H -3.56162500 1.85728100 -1.15332000

C -3.96546100 1.42735400 0.94951100

H -4.89241800 1.87178300 1.31655800

H -4.17633800 0.39674400 0.61115800

C -2.91699800 1.33824200 2.04063700

O -1.68427800 1.59849400 1.49884900

O -3.04942900 1.07150100 3.20778900

H 0.20492500 3.51640000 -1.52144700

H 0.44457800 4.78708100 -0.33754200

H 2.14018600 3.36851100 0.39535500

H 0.81738500 2.70288100 1.33979900

C -2.66523500 -3.15501100 -0.05131500

C -3.56651200 -2.76176700 0.89575600

H -3.47258100 -2.92616300 1.96577300

C -4.65111300 -2.08935400 0.23686600

C -5.96655500 -1.65255800 0.81156200

H -6.59087500 -2.49224100 1.16942900

H -5.87401900 -0.96109100 1.66656200

H -6.54196700 -1.13974900 0.03045500

C -4.37867400 -2.10313400 -1.15053700

O -3.11332700 -2.78769400 -1.30373900

O -4.95072600 -1.67441900 -2.16436600

H -1.54229500 -4.95381500 0.37362300

**CP13**

C 0.22229300 0.56051100 -0.64120100

C 0.49752700 -0.38476800 -1.85615200

C 1.48154800 -0.69185700 0.69919700

C -0.30676400 -1.70517200 -1.82274000

C 1.79790600 -1.98636300 0.63115600

C 0.32480600 -3.02361900 -1.26410700

H 1.57260100 -0.58980400 -1.91215800

H -1.24930200 -1.54773100 -1.29966500

H 0.25629600 0.17491100 -2.77029500

H -0.58889800 -1.96467800 -2.85126900

H 2.84120100 -2.26820900 0.75088900

C 1.49549900 1.44469400 -0.38970700

H 2.06476800 1.51322100 -1.32411200

C 2.32856000 0.56471200 0.60511500

O 0.18055000 -0.28115500 0.53853800

C 0.81468500 -3.06302000 0.22856500

H 1.33683200 -4.02706900 0.31442500

C 1.40991600 -3.53670100 -2.21617700

H 1.00322100 -3.65523300 -3.22709500

H 2.25652900 -2.84050300 -2.26522600

H 1.78247300 -4.51225400 -1.88398100

C -0.50699600 -3.20015600 1.01604600

H -0.92457800 -2.20695200 1.18212500

C -0.36280700 -3.90073800 2.36739600

H 0.27587300 -3.32377300 3.04777100

H -1.34374100 -4.01502700 2.84412800

H 0.07736800 -4.90206900 2.25540500

C -1.44697400 -3.96485400 0.03815700

O -0.72069500 -3.99917300 -1.25534700

H 2.36859100 1.07548800 1.58043500

C -1.17297400 1.19196300 -0.79107200

C -1.66653100 2.29414600 0.15526100

C -1.05983800 3.71233200 -0.02897800

C 1.31467300 2.87058500 0.15584600

C 0.33937000 3.75725700 -0.64815300

H -1.25958500 1.58489500 -1.81098400

H -1.89358200 0.36848400 -0.72091900

H -0.96735900 4.10648000 0.99017800

O 3.65596700 0.37788600 0.12948400

C 4.60478000 0.11109400 1.14158100

H 4.39074700 -0.84379400 1.64691400

H 4.54943300 0.89396200 1.91990600

C 6.00108300 0.07685400 0.55701700

C 6.26827400 0.55179200 -0.73077600

C 7.06003400 -0.41613300 1.33100500

C 7.57180800 0.53536400 -1.23308300

H 5.44643900 0.92192300 -1.33323800

C 8.36236300 -0.42794400 0.83243300

H 6.86196200 -0.79660100 2.33159300

C 8.62316500 0.04896300 -0.45487500

H 7.76407600 0.90274500 -2.23833500

H 9.17219400 -0.81528900 1.44597000

H 9.63662400 0.03615800 -0.84818700

C -2.20146000 4.53544200 -0.68560200

C -2.26402400 4.43167000 -2.22043300

H -1.39547700 4.90736700 -2.69107800

H -2.30556100 3.39252500 -2.56051200

H -3.16832100 4.93510200 -2.57939200

C -2.18468800 6.00467000 -0.25970700

H -1.28249300 6.50997300 -0.62452800

H -3.05767800 6.52896300 -0.66456500

H -2.21536200 6.08307700 0.83190900

O -3.37959300 3.95490600 -0.10085000

C -3.20204500 2.53395000 -0.01765600

H -3.59270800 2.04396000 -0.91568500

C -3.82203400 1.95228600 1.24037900

H -4.61662000 2.57505500 1.65816500

H -4.22622600 0.95159500 1.00154500

C -2.67370600 1.80645200 2.21454800

O -1.48400300 1.90064000 1.53712300

O -2.70153200 1.62272600 3.40622400

H 0.32210400 3.44216700 -1.69974000

H 0.69276600 4.79749000 -0.64822100

H 2.31231900 3.33048000 0.18015800

H 0.97035400 2.82186800 1.19591700

C -2.81519000 -3.41077900 -0.03615600

C -4.05801900 -3.98078600 -0.11226800

H -4.25306300 -5.04920800 -0.08724600

C -5.02973200 -2.94503200 -0.23196100

C -6.52057100 -3.05218900 -0.33270700

H -6.86090600 -3.61488300 -1.21892200

H -6.98421100 -3.54153700 0.54081100

H -6.94508600 -2.04383000 -0.40481900

C -4.33515100 -1.72105100 -0.23913000

O -2.95919500 -2.03039500 -0.10365800

O -4.66963500 -0.51521300 -0.33453600

H -1.53163400 -5.02267300 0.31972200

**TS12**

C -2.23916100 0.81237800 0.63748200

C -2.32316900 -0.11886100 1.89112400

C -2.48678800 -0.93133200 -0.71568200

C -1.01886100 -0.91964100 2.13980300

C -2.21688400 -2.22582800 -0.54225100

C -0.89494400 -2.40974600 1.69319800

H -3.18003700 -0.79200300 1.78673300

H -0.16940700 -0.38670100 1.71062400

H -2.54464100 0.50864500 2.76393700

H -0.82680500 -0.94962800 3.21924600

H -2.99319800 -2.94183800 -0.79679200

C -3.65962400 1.02552700 0.02028400

H -4.41420400 0.96954500 0.81283600

C -3.82546500 -0.23444500 -0.89829600

O -1.56486700 0.04313200 -0.39376600

C -0.99055500 -2.74851500 0.16600600

H -1.03830100 -3.84634700 0.12894000

C -1.82375900 -3.29852200 2.52389800

H -1.63959300 -3.14939200 3.59353400

H -2.87630700 -3.06853500 2.32087200

H -1.65067700 -4.35528600 2.29407900

C 0.40321900 -2.35469700 -0.36337600

H 0.41789200 -1.27641200 -0.53616300

C 0.79879400 -3.07265900 -1.65495600

H 0.11662200 -2.80844100 -2.47132100

H 1.81419700 -2.80221900 -1.96910900

H 0.76793400 -4.16340700 -1.52841600

C 1.32318400 -2.68422600 0.83844200

O 0.45667700 -2.79768600 2.00430600

H -3.96196100 0.09739900 -1.93887800

C -1.37315600 2.02797600 1.00762600

C -1.05605500 3.15332600 0.01813300

C -2.20048600 4.12773700 -0.36922600

C -3.90485200 2.30096300 -0.80484200

C -3.62305800 3.63553400 -0.08802600

H -1.83316500 2.49917400 1.88489700

H -0.41160000 1.63045200 1.35319300

H -2.11093700 4.24663400 -1.45517800

O -4.94916500 -1.00783500 -0.50772500

C -5.57127100 -1.72240400 -1.56272500

H -4.88466700 -2.46544400 -1.99727100

H -5.83129500 -1.02215100 -2.37661700

C -6.82082700 -2.41363000 -1.06182500

C -7.45017800 -2.01981500 0.12331000

C -7.38421700 -3.44872500 -1.81899900

C -8.62390800 -2.65056500 0.54201900

H -7.00775200 -1.22704700 0.71622400

C -8.56049600 -4.07374100 -1.40545200

H -6.89749500 -3.76985200 -2.73814900

C -9.18475100 -3.67613500 -0.22059200

H -9.09992600 -2.33899600 1.46836600

H -8.98475800 -4.87610700 -2.00354000

H -10.09791800 -4.16596000 0.10702200

C -1.77278600 5.49285600 0.24853900

C -2.26543000 5.71578100 1.68855900

H -3.35138800 5.85570500 1.72026100

H -2.01708500 4.87838600 2.34955200

H -1.79214600 6.61494500 2.09648300

C -2.15641100 6.68083700 -0.63299100

H -3.24531400 6.76060300 -0.72836300

H -1.78244100 7.61540400 -0.20117800

H -1.72343500 6.56718000 -1.63187000

O -0.33226600 5.41579200 0.22770300

C 0.05033300 4.09504900 0.59194100

H 0.13177200 4.00234000 1.68252000

C 1.32164100 3.67007200 -0.12148500

H 1.94144800 4.51825200 -0.42224700

H 1.92379000 2.99282300 0.50260000

C 0.83637400 2.89885500 -1.33478700

O -0.48404500 2.60405500 -1.20056900

O 1.46897900 2.54432600 -2.30312100

H -3.80634600 3.53400600 0.98928600

H -4.32838500 4.39843300 -0.44277400

H -4.95370600 2.26675100 -1.12818000

H -3.30193200 2.25869800 -1.72119800

C 2.47043200 -1.72883300 1.03063600

C 3.38071000 -1.83103500 2.12953400

H 3.77728300 -2.77657300 2.48954700

C 3.70278700 -0.58641600 2.61152800

C 4.67101300 -0.17657900 3.67904400

H 4.16308400 0.33033100 4.50979700

H 5.20113600 -1.04274600 4.09084100

H 5.41820000 0.53312200 3.29778100

C 2.90579200 0.36932200 1.87039700

O 2.10495300 -0.33474300 0.99646400

O 2.89621200 1.60311500 1.89280800

H 1.77010300 -3.68273300 0.70895700

H 3.42246100 -1.61937700 -0.24787700

C 3.48763600 0.00107900 -1.78895200

C 4.35417100 1.24338600 -1.63822100

C 5.76781000 0.91135200 -2.09319100

H 3.94222700 2.06263400 -2.23202100

H 3.36860900 -0.26900400 -2.84643600

H 2.49525000 0.15819100 -1.36964300

H 5.81218100 0.80513200 -3.18687500

H 6.46085400 1.71316200 -1.81539200

H 4.35051600 1.55840100 -0.58830600

C 5.88226000 -2.46398200 -0.18898700

C 5.40608800 -1.24343600 -0.96025000

C 6.99080900 -3.28836200 -0.87167800

C 7.71918600 -0.55541200 -1.68516200

C 8.40307900 -2.77071500 -0.55716100

C 8.48738500 -1.23618000 -0.54575200

H 6.20288000 -2.14676300 0.81094900

H 7.87134800 -1.10026300 -2.62925800

H 6.82021200 -3.29870600 -1.95585500

H 9.10882900 -3.17576200 -1.29355000

H 4.99793000 -3.08560700 -0.04182100

H 6.90307900 -4.33114700 -0.54703100

H 8.13857300 0.44280800 -1.83597300

H 8.72694800 -3.14814700 0.42094100

H 9.53824000 -0.93290600 -0.62606300

H 8.12885000 -0.82969300 0.40720400

N 4.10291800 -1.12360800 -1.07671100

N 6.27406800 -0.33364300 -1.46935900

**30**

C 0.25018200 0.71608700 -0.49081100

C 0.53708000 -0.35061200 -1.59803000

C 1.45421400 -0.44842000 0.96705300

C -0.31992300 -1.63096500 -1.41930600

C 1.74624200 -1.74751700 1.04425400

C 0.26742200 -2.91278400 -0.75270200

H 1.60469800 -0.59097600 -1.60728400

H -1.23016700 -1.38152700 -0.87346200

H 0.32400500 0.10241200 -2.57420000

H -0.65240200 -1.97146600 -2.40756100

H 2.78230900 -2.03129200 1.20123200

C 1.50281500 1.61612600 -0.24889300

H 2.05567600 1.73796300 -1.18716200

C 2.37064500 0.74022700 0.71952900

O 0.16029300 -0.02527700 0.75543700

C 0.76000100 -2.84622900 0.73254000

H 1.27864300 -3.80174200 0.89753600

C 1.30943100 -3.56550900 -1.66192100

H 0.89346600 -3.74072600 -2.66000100

H 2.19123300 -2.92303100 -1.76582900

H 1.63115400 -4.52902700 -1.25298400

C -0.55313800 -2.92073100 1.54002500

H -1.02455800 -1.93552900 1.53551700

C -0.37674800 -3.38491300 2.98582800

H 0.24507200 -2.67460400 3.54112100

H -1.33883700 -3.45293600 3.50801800

H 0.10599700 -4.37004100 3.03476100

C -1.37967200 -3.88751800 0.66462700

O -0.85662700 -3.83439300 -0.66096900

H 2.55453900 1.30214800 1.64745300

C -1.10438000 1.37260200 -0.80112000

C -1.74379000 2.40946700 0.12803800

C -1.11552300 3.82820000 0.16817200

C 1.27873200 3.00400500 0.38045300

C 0.31553300 3.94702200 -0.36593200

H -1.01713200 1.83693900 -1.79126100

H -1.83283500 0.56196400 -0.91856000

H -1.08193200 4.08813300 1.23233200

O 3.61386800 0.40261600 0.12523800

C 4.68843600 0.23046500 1.03855800

H 4.52395600 -0.64349100 1.68726600

H 4.74436800 1.11053900 1.70345400

C 5.98777700 0.06982300 0.28158600

C 6.17856400 0.67491100 -0.96606100

C 7.03972900 -0.65703300 0.85142800

C 7.40086700 0.55514800 -1.62896300

H 5.35965000 1.22578600 -1.41648300

C 8.26558400 -0.76950300 0.19385100

H 6.89917800 -1.14111200 1.81591600

C 8.44929200 -0.16361700 -1.05060800

H 7.53433300 1.02451700 -2.60034200

H 9.07242400 -1.33783900 0.64911800

H 9.40055100 -0.25530700 -1.56804400

C -2.19973400 4.75081700 -0.46570000

C -2.08817500 4.90050600 -1.99253400

H -1.19883700 5.47514500 -2.27332300

H -2.03600700 3.93308100 -2.50382700

H -2.97038100 5.42927600 -2.36791300

C -2.26330300 6.12475300 0.19996000

H -1.32733900 6.67476200 0.04989600

H -3.07968300 6.71894000 -0.22425100

H -2.43956800 6.01757100 1.27487700

O -3.42992700 4.07510200 -0.12919000

C -3.22784200 2.67679000 -0.28276600

H -3.41985500 2.36809700 -1.31864600

C -4.05046700 1.88846900 0.72146200

H -4.93279800 2.43181200 1.06695700

H -4.36933800 0.92556900 0.30490900

C -3.09490900 1.61998600 1.87560300

O -1.81465600 1.89268500 1.48011800

O -3.34839000 1.21746200 2.98086600

H 0.36102500 3.75788000 -1.44619800

H 0.64671000 4.98477300 -0.22993900

H 2.26887900 3.47266500 0.45600400

H 0.92195200 2.87895500 1.41089700

C -2.89401700 -3.60270400 0.67552300

C -3.69075400 -4.36228600 -0.34600100

H -3.69818600 -5.44597700 -0.39395300

C -4.33585800 -3.51315500 -1.15522600

C -5.23490000 -3.75424200 -2.32393200

H -4.81753800 -3.30107000 -3.23084300

H -5.38025200 -4.82314500 -2.50338500

H -6.21394800 -3.28804400 -2.16218500

C -3.99862600 -2.13696800 -0.70795400

O -3.17072700 -2.22174600 0.37648600

O -4.35329000 -1.07289900 -1.16925500

H -1.26712300 -4.92100200 1.03541500

H -3.26017200 -3.79164800 1.69491100

**TS13**

C 1.87234600 0.83450600 -0.55544400

C 1.54922500 -0.14111300 -1.73700000

C 2.60465700 -0.82814000 0.72001700

C 0.28285700 -1.00670900 -1.50335100

C 2.38518900 -2.13944900 0.63708900

C 0.42966400 -2.49605900 -1.05449500

H 2.41656200 -0.78399800 -1.91286600

H -0.38052800 -0.51306000 -0.78842500

H 1.43539300 0.45443000 -2.65192800

H -0.28363400 -1.06135100 -2.44095200

H 3.25695600 -2.78724800 0.65848200

C 3.42197100 1.07574400 -0.46649100

H 3.86209500 0.86177800 -1.44682400

C 3.88903000 -0.05171400 0.51564100

O 1.57834500 0.09613400 0.66155400

C 1.05124800 -2.78186400 0.35286200

H 1.21631800 -3.86861800 0.36663300

C 1.10945800 -3.32204000 -2.15080100

H 0.58424200 -3.20017200 -3.10488200

H 2.15374800 -3.02044800 -2.29265100

H 1.09104700 -4.38476100 -1.88875400

C -0.13178600 -2.52734400 1.30965500

H -0.26597400 -1.44608200 1.42659400

C 0.05171300 -3.16445600 2.68817500

H 0.91240700 -2.72513900 3.20492300

H -0.83134300 -3.01830700 3.31852500

H 0.22319000 -4.24516600 2.60118200

C -1.29892300 -3.11751100 0.49623000

O -0.91593700 -2.99336600 -0.91002300

H 4.20844600 0.41453300 1.46058800

C 0.91139000 2.03311300 -0.64044200

C 1.02479200 3.24507900 0.29124400

C 2.18512900 4.24205700 0.02463400

C 3.95451700 2.43979600 0.00626600

C 3.39325800 3.67397800 -0.72475600

H 0.94987700 2.41916400 -1.66554200

H -0.09666700 1.61686800 -0.51751600

H 2.53579900 4.52880300 1.02304900

O 4.96357600 -0.79324800 -0.03037000

C 5.83766900 -1.36063000 0.93920600

H 5.31115500 -2.09987000 1.56070200

H 6.18640300 -0.56457200 1.62047100

C 7.01633900 -2.00868900 0.25027400

C 7.55528500 -1.46279200 -0.92114700

C 7.61541900 -3.14388200 0.80774000

C 8.67407800 -2.04209800 -1.52040600

H 7.08241100 -0.59257800 -1.36484600

C 8.74057200 -3.71917600 0.21439800

H 7.19814800 -3.58357200 1.71132700

C 9.27284900 -3.16953900 -0.95304700

H 9.07945800 -1.61244400 -2.43296900

H 9.19394800 -4.60120100 0.65901600

H 10.14459600 -3.61938400 -1.42075500

C 1.49013500 5.50576500 -0.55534000

C 1.28457000 5.48037600 -2.07861000

H 2.23997500 5.55252600 -2.60919000

H 0.78257800 4.56789100 -2.41785500

H 0.66411100 6.33263300 -2.37427900

C 2.18102600 6.80165200 -0.13362000

H 3.19278800 6.85762500 -0.55039100

H 1.61709800 7.67001100 -0.49031900

H 2.24721400 6.86022500 0.95729100

O 0.20756800 5.49086600 0.11370800

C -0.24905100 4.15030900 0.19820500

H -0.85486100 3.89433700 -0.68217500

C -0.98985500 3.91512100 1.50643400

H -1.30475600 4.85066600 1.97483500

H -1.87715600 3.28383000 1.38336700

C 0.00367800 3.18084800 2.40317900

O 1.08979800 2.80449700 1.67018300

O -0.10329400 2.93166300 3.57450100

H 3.14358100 3.42170300 -1.76351700

H 4.16333700 4.45395900 -0.78068100

H 5.04535200 2.39985900 -0.10891600

H 3.76767100 2.55381900 1.08123100

H -1.36178200 -4.19993900 0.69258300

C -2.65020400 -2.50621400 0.74557200

C -3.08592700 -1.93899400 1.98294900

H -2.47302400 -1.28835500 2.59984600

C -4.40043700 -2.26554100 2.22188100

C -5.32544900 -1.81628800 3.31103000

H -5.73265100 -2.66901800 3.86945300

H -4.81542900 -1.15948200 4.02462800

H -6.19600900 -1.27381100 2.91211200

C -4.82354300 -3.14459700 1.14764400

O -3.72053500 -3.36906400 0.33810200

O -5.92487100 -3.60683700 0.86325300

H -3.02253200 -1.40438700 -0.36497000

C -3.72648100 -1.96225500 -2.35911000

C -5.14350700 -2.50237500 -2.50627900

C -6.09507500 -1.32522000 -2.67157100

H -5.22708500 -3.15703100 -3.38064600

H -3.40267500 -1.45306800 -3.27811800

H -2.99891600 -2.74318300 -2.13440000

H -5.99718000 -0.87398600 -3.67033600

H -7.13300700 -1.65694700 -2.56093800

H -5.40770100 -3.08208100 -1.61541500

C -4.49481100 0.72431100 0.20190800

C -4.69836000 -0.22824300 -0.96497200

C -4.86254100 2.19446500 -0.07968200

C -6.91749800 0.76069500 -1.63462500

C -6.34689900 2.49614000 0.18320200

C -7.27606100 1.35878800 -0.26934700

H -5.05291400 0.35212300 1.06766100

H -6.65620100 1.55970000 -2.34580400

H -4.59720400 2.44723100 -1.11474400

H -6.62355900 3.42754200 -0.32725900

H -3.43856200 0.64612000 0.46544000

H -4.24405800 2.84134600 0.55365100

H -7.80637700 0.26888200 -2.03818500

H -6.50343900 2.67204000 1.25470000

H -8.30294600 1.73827200 -0.33381700

H -7.29918900 0.54959700 0.46943000

N -3.66933400 -0.99921200 -1.25414000

N -5.86576200 -0.27682600 -1.65207800

**TS14**

C -2.45025000 0.76962500 0.61293000

C -2.43462800 -0.21351200 1.83129100

C -2.52861100 -0.94528400 -0.79255200

C -1.04879100 -0.88290200 2.04417000

C -2.12293100 -2.20874800 -0.66739800

C -0.75681400 -2.33639500 1.54411100

H -3.21618100 -0.96797200 1.70043100

H -0.26813500 -0.24332600 1.62923900

H -2.71491200 0.35154700 2.72961600

H -0.84060900 -0.92624300 3.11964000

H -2.82449000 -2.99051100 -0.94386000

C -3.87562500 0.87486200 -0.01303600

H -4.63637100 0.78305300 0.77004400

C -3.94369800 -0.40104400 -0.91924000

O -1.70563800 0.11251300 -0.44374500

C -0.84367200 -2.63110300 0.00780100

H -0.78524300 -3.72615100 -0.06701000

C -1.57323300 -3.34620900 2.35583300

H -1.38248500 -3.21698600 3.42678500

H -2.64841300 -3.22262700 2.17915500

H -1.29342700 -4.36944100 2.08437600

C 0.49834400 -2.09545200 -0.52414100

H 0.42698300 -1.00816900 -0.62925400

C 0.90757700 -2.69557500 -1.87051000

H 0.18924900 -2.42195400 -2.65197900

H 1.89626000 -2.34330300 -2.18490500

H 0.94705900 -3.79161400 -1.81605000

C 1.46128900 -2.44450500 0.63243900

O 0.63260100 -2.57371100 1.83006400

H -4.17235600 -0.10125100 -1.95325600

C -1.73160200 2.06012500 1.04581200

C -1.43793900 3.21387400 0.08091300

C -2.63321300 4.07852000 -0.40090400

C -4.18409900 2.11512200 -0.87255400

C -4.02807200 3.48263100 -0.18354400

H -2.30473700 2.48020000 1.88153300

H -0.77125600 1.75197900 1.47699000

H -2.49010300 4.18043400 -1.48266100

O -4.95348100 -1.28769900 -0.46227200

C -5.56247100 -2.08319500 -1.47027700

H -4.85641100 -2.82224300 -1.87778200

H -5.86182300 -1.43376900 -2.31211600

C -6.77356600 -2.78971800 -0.90409600

C -7.56815800 -2.18463800 0.07702500

C -7.14135000 -4.04827800 -1.39342600

C -8.70998800 -2.82728000 0.55639200

H -7.27567100 -1.21679000 0.47097100

C -8.28868800 -4.68837800 -0.92160500

H -6.52432700 -4.53389000 -2.14679300

C -9.07658700 -4.07905600 0.05643500

H -9.31464500 -2.34989500 1.32331100

H -8.55994900 -5.66629900 -1.31059600

H -9.96627200 -4.57835500 0.43083600

C -2.36763900 5.48854700 0.21044200

C -2.98718100 5.70076500 1.60139800

H -4.07964600 5.75390700 1.54775300

H -2.72650500 4.89945800 2.30159000

H -2.61910900 6.64334100 2.01923500

C -2.77019200 6.62149400 -0.73198500

H -3.85003100 6.60775200 -0.91764800

H -2.51134900 7.59276700 -0.29728400

H -2.24835200 6.52340900 -1.68910100

O -0.92643600 5.53064000 0.30987300

C -0.47020900 4.25613500 0.73015700

H -0.47482400 4.17984900 1.82600900

C 0.89081800 3.95459700 0.12483000

H 1.41816500 4.86195100 -0.17947100

H 1.54338600 3.39819000 0.80713700

C 0.59093500 3.08114300 -1.08771700

O -0.71593400 2.71436800 -1.07986800

O 1.35783000 2.72423100 -1.94876800

H -4.26610300 3.39563600 0.88410900

H -4.76357500 4.18420400 -0.59806000

H -5.21748400 1.99881000 -1.22456300

H -3.55418300 2.09554400 -1.77184900

C 2.60282100 -1.48089900 0.78223100

C 2.49043300 -0.11751900 1.20548600

H 1.70589000 0.55319700 0.86863100

C 3.55683800 0.22355200 1.99989900

C 3.95082100 1.53992400 2.59661300

H 3.31272400 2.35580000 2.23537200

H 3.88217300 1.52636400 3.69271900

H 4.99332900 1.79023500 2.35822000

C 4.35887700 -0.97453300 2.16659800

O 3.70121500 -2.00838700 1.53122500

O 5.46904600 -1.14062400 2.67322800

H 1.89534400 -3.44059800 0.46294000

H 3.46013500 -1.33840400 -0.56135600

C 3.73766100 0.27640200 -1.98571100

C 4.57676200 1.48462800 -1.60162900

C 6.03631400 1.16864800 -1.89493700

H 4.25837800 2.37251200 -2.15511400

H 3.74543500 0.12843800 -3.07366300

H 2.69607700 0.40816200 -1.68385900

H 6.22165700 1.14795000 -2.97909500

H 6.68654500 1.93965800 -1.46976900

H 4.44880100 1.68668400 -0.53277600

C 5.90914900 -2.42308400 -0.43229300

C 5.52946900 -1.08943600 -1.05418200

C 7.17927700 -3.08521100 -0.99936100

C 7.91536700 -0.27094300 -1.17157000

C 8.44746100 -2.61352900 -0.27246500

C 8.41316700 -1.10541900 0.01489700

H 6.00131600 -2.29632900 0.65329700

H 8.33487600 -0.65128900 -2.11530400

H 7.25888300 -2.89191100 -2.07752500

H 9.33243500 -2.86296200 -0.87295400

H 5.04697700 -3.07245000 -0.58674600

H 7.07907700 -4.17162400 -0.89812900

H 8.29091100 0.74973300 -1.05824700

H 8.54964200 -3.15070800 0.67809200

H 9.42347300 -0.75683000 0.26189700

H 7.78874000 -0.88868900 0.88941800

N 4.24406300 -0.93654100 -1.34032000

N 6.44828000 -0.12508200 -1.30880900

**CP14**

C -0.76018400 0.79597600 0.68489400

C -0.41765000 -0.10648700 1.91788200

C -0.94570900 -0.96988600 -0.65192000

C 1.01842900 -0.69105500 1.88004600

C -0.46517900 -2.20760200 -0.53689000

C 1.24230300 -2.16796300 1.43039100

H -1.15052600 -0.91626200 1.98000300

H 1.65730600 -0.05530700 1.26084000

H -0.55659400 0.48741600 2.82985100

H 1.44562600 -0.64746000 2.88922400

H -1.16180900 -3.02841500 -0.67865000

C -2.29636500 0.71375600 0.37436800

H -2.81972100 0.41483600 1.28931000

C -2.38186200 -0.48597600 -0.63005600

O -0.15088700 0.14205500 -0.46196100

C 0.92059100 -2.55218100 -0.05126200

H 1.00067700 -3.64839100 -0.07766500

C 0.60461700 -3.13957500 2.42484300

H 0.94289900 -2.92606400 3.44478700

H -0.48841100 -3.06726600 2.40256200

H 0.88528500 -4.16952200 2.18265700

C 2.15227600 -2.02026900 -0.81780700

H 2.06269500 -0.93298500 -0.91995900

C 2.34729400 -2.63294900 -2.20489300

H 1.49946200 -2.38782900 -2.85378500

H 3.25942000 -2.25347600 -2.67436500

H 2.42467700 -3.72658700 -2.14613400

C 3.27006900 -2.35512900 0.18901300

O 2.67903900 -2.37410800 1.49611300

H -2.67178800 -0.09947200 -1.61914400

C -0.08564900 2.16494400 0.87767400

C -0.31424200 3.33430900 -0.08830300

C -1.67664200 4.07281300 0.00221100

C -3.01791000 1.94226500 -0.20581800

C -2.83756000 3.25746100 0.57698000

H -0.34647900 2.52598900 1.87886300

H 0.99351200 1.96776400 0.90451200

H -1.93324700 4.30322500 -1.03840800

O -3.32942800 -1.44439300 -0.19934800

C -3.94607700 -2.17888100 -1.25168900

H -3.20627200 -2.78238000 -1.79838400

H -4.37854300 -1.47121500 -1.98057600

C -5.02704100 -3.07255000 -0.68845100

C -5.78602000 -2.67624800 0.41936200

C -5.31300400 -4.29616500 -1.30454100

C -6.81256800 -3.49025300 0.89925700

H -5.55640600 -1.73503300 0.90820100

C -6.34600000 -5.10709100 -0.83132000

H -4.72272200 -4.61892700 -2.15971500

C -7.09858600 -4.70616700 0.27388600

H -7.39078000 -3.17433500 1.76383500

H -6.55543900 -6.05492500 -1.32009800

H -7.89891200 -5.33869800 0.64854100

C -1.33750900 5.43914300 0.66112000

C -1.33561600 5.42746000 2.19844000

H -2.34755300 5.29407500 2.59579100

H -0.70674200 4.63123900 2.61100000

H -0.94716500 6.38259900 2.56635900

C -2.21592100 6.57610300 0.14073600

H -3.26274100 6.42193500 0.42534000

H -1.88725100 7.53413800 0.55712900

H -2.15381600 6.63725700 -0.95030500

O -0.00239900 5.69688800 0.16910500

C 0.72644700 4.47900400 0.15522800

H 1.25637900 4.33809500 1.10730800

C 1.65785800 4.42045400 -1.04705600

H 1.82853600 5.40790600 -1.48235700

H 2.63439200 3.98546900 -0.80685500

C 0.95780400 3.51128200 -2.05271400

O -0.10464900 2.89744000 -1.45227400

O 1.25013700 3.31080300 -3.20090800

H -2.69512600 3.04895200 1.64534300

H -3.75253900 3.85917500 0.50695100

H -4.08209200 1.67816200 -0.25597600

H -2.69434700 2.10099800 -1.24183300

C 4.44327800 -1.36063600 0.23353500

C 5.48715500 -1.69888800 1.25931500

H 5.25327300 -1.77434700 2.31457100

C 6.66646000 -1.90130800 0.65982000

C 8.00570300 -2.26743700 1.21196500

H 7.97334600 -2.38838400 2.29842900

H 8.36476300 -3.20153400 0.76403900

H 8.74772500 -1.49885500 0.96490800

C 6.46926700 -1.70131600 -0.80301900

O 5.14775000 -1.37657700 -1.01809300

O 7.27249400 -1.79136100 -1.69849900

H 3.68553800 -3.35207800 -0.02381000

H 4.04151400 -0.34919300 0.38323300
